# Little evidence that nonmonogamous family structures are detrimental to children’s well-being in Mpimbwe, Tanzania

**DOI:** 10.1073/pnas.2407785121

**Published:** 2024-12-20

**Authors:** Riana Minocher, Monique Borgerhoff Mulder, Cody T. Ross

**Affiliations:** ^a^Department of Human Behavior, Ecology and Culture, Max-Planck-Institute for Evolutionary Anthropology, 04103 Leipzig, Germany; ^b^Berlin Institute for Health at Charité, 10178 Berlin, Germany; ^c^Department of Anthropology, University of California, Davis, CA 95616

**Keywords:** polygyny, monogamy, step-parent, survival, well-being

## Abstract

Nuclear family structures are often thought to be essential for the well-being of children. Divorce, the loss of either biological parent, the presence of step-parents, and the practice of polygynous marriage have all been claimed to negatively impact child well-being. However, empirical research on these topics has been limited by the routine use of cross-regional and cross-sectional databases. Cross-regional data render research vulnerable to the ecological inference fallacy, and cross-sectional data prevent assessment of age-specific impacts of time-varying family-structure variables. When longitudinal data are available, they tend to be drawn from Western/urban contexts. Detailed data on family structure and children’s well-being are rarely collected in more marginalized communities. In many rural and traditional communities, nonnuclear family structures are indeed prevalent and viewed as socially permissible—and, as such, may have different impacts on children’s well-being than in Western contexts. Here, we draw on a detailed, longitudinal dataset from a 20-y prospective study in rural Tanzania, where polygyny and serial monogamy are common. We analyze survival outcomes for 3,693 children born between 1931 and 2014, growth outcomes for 881 children born between 1976 and 2014, and educational outcomes for 1,370 children born between 1976 and 2014. Our analyses indicate that monogamous marriage is not consistently associated with better outcomes for children—contrary to some popular and public health perspectives on human family structure.

## Significance

The relationship between family structure and child well-being is rarely studied in marginalized communities. Here, we present data from a region in which nonnuclear family structures are common and culturally permissible. We pair these data with nuanced statistical methods to study the long-term associations between dynamically changing family structures and children’s outcomes. Our findings challenge prevailing assumptions about monogamy, and offer insights into the diverse ways in which children’s welfare can be supported across sociocultural settings.

In some contexts, children are raised by biological parents in a nuclear setting. In other contexts, children might be raised by a single parent, a step-parent, in a polygynous family, or by other caregivers (1). Such nonnuclear family structures are sometimes thought to be detrimental to children’s well-being, due to the economic, social, or emotional challenges they impose (2, 3). Indeed, the death or absence of parents (4, 5), the divorce and remarriage of biological parents to step-parents (6–9), and the practice of polygyny (10) have all been shown to be associated with adverse outcomes for children. Reported adversities range in severity from poorer educational attainment and diminished physical development to substantially increased mortality rates. Despite these associations, polygyny remains prevalent in many non-Western parts of the world (11), and divorce and single-parenthood are increasingly common globally, including in the West (12–14).

The apparent inconsistency between the prevalence of nonnuclear family structures and their reported negative consequences for children presents a challenge for evolutionary and public health scholars (for discussion, see: refs. 15 and 16). A primary concern is that much existing research relies on cross-regional or cross-sectional datasets, which are vulnerable to inferential confounds (e.g., the ecological inference fallacy: ref. 17) and are unable to account for the time-varying nature of family structure and child development. When longitudinal data are available, they come primarily from Western or urban contexts where nuclear families are normative (see: ref. 4). Longitudinal data from non-Western, rural, and indigenous populations are relatively rare in comparison (but see: refs. 5, 18, and 19), which limits our understanding of the impacts of diverse family structures in these contexts.

In marginalized communities, where families tend to lack economic security, face frequent and unpredictable environmental shocks, and have limited access to state services or robust infrastructure, extended family structures are not only common and culturally permissible, but seem to play a crucial role in child-rearing (1, 20, 21). Some have argued that nonnuclear arrangements increase costs for women and children by intensifying intrafamilial conflict or competition over resources (22)—e.g., as has been described among the Dogon of Mali (18)—and these effects may be more pronounced for poor families in harsh environments. At the same time, however, in cases where women have little economic buffering from the state, little rival wealth to promote intrafamilial conflict, and must contend with highly variable environments, the accumulation of a network of potential providers—through repeated divorce and remarriage—may be seen as an adaptive strategy (23). Such patterns have been described not only in anthropological contexts (24) but also in marginalized communities in Western contexts (25–27).

Here, we examine whether nonnuclear family structures are associated with favorable or unfavorable child outcomes. We draw on a longitudinal dataset from a 20-y study in a rural Tanzanian village, which is primarily inhabited by indigenous Pimbwe people. The village’s poor infrastructure, unpredictable rainfall, unreliable crop yields, and limited administrative services create a challenging environment for its inhabitants. There is substantial variation in family structure between Pimbwe households, and—even within households—many children are exposed to a variety of family structures across their childhoods. Among Pimbwe, nonnuclear family structures, such as polygyny or extended family arrangements are normative, and may not carry the same negative consequences as are often reported in Western contexts. Consequently, our data and analysis aim to provide insights into how different family structures function in diverse environments, potentially challenging prevailing assumptions about the universality of their impacts on child well-being.

## Local Contexts Will Determine Outcomes.

The impacts of family structure on children’s well-being have been shown to vary—in magnitude and direction—across different contexts. For example, the practice of polygyny might be causally associated with reduced child well-being in some cultural contexts (e.g., where women are prevented from freely choosing partners or men coercively take on more wives than they can support; 28, 29). In other contexts, polygyny may be associated with improvements in child well-being (e.g., if polygynous families are wealthier than monogamous families, or if there are benefits to cooperation between co-wives; 30, 31). However, attempts to study such patterns comparatively have typically failed to consider contextual variation in factors (e.g., economic conditions, material infrastructure, state services, or the effects of colonialism), which not only affect both family structure (e.g., marital institutions) and children’s well-being but also mediate how family structure might impact child outcomes.

In one example to the contrary, Lawson et al. (30) show how inferences about the causes of diminished child well-being can be confounded when patterns in regionally aggregated data are assessed. In their data, the practice of polygyny appears to be associated with high childhood food insecurity across multiple villages in Tanzania. However, within villages, children in polygynous households show improved metrics of growth when compared to children in monogamous households in the same community. By partitioning effects using multilevel models, Lawson et al. (30) show that the positive association between child well-being and polygyny within villages is driven by elevated wealth and food security in polygynous families, whereas the negative correlation between the aggregated (i.e., village-level) summaries of these variables is driven by the nonrandom distribution of polygyny across villages. Polygyny is more frequently practiced in marginalized regions, where children in all family structures tend to suffer from poorer nutrition in general (30). If associations are not analyzed within particular cultural or regional contexts, the root causes of deficiencies in measures of childhood well-being (e.g., poverty or gender inequality: 32) might be conflated with aggregate variation in family structure or other variables, and, as such, the specific causal pathways may be misunderstood.

Notably, inferential problems are further exacerbated by the fact that impacts of varying family circumstances on child outcomes can also depend on the frequency of the marital practice in the population—as seen in studies of divorce (33) and polygyny (32). Such findings suggest that impacts on child outcomes can change over time, simply as a consequence of the shifting prevalence of the practice, even if the context (poverty or religion) remains stable.

## Longitudinal Data Afford More Precise Inference.

Despite the importance attributed to detailed and context-specific sociodemographic data, they are seldom collected in marginalized regions where nonnuclear family forms are both common and culturally acceptable (11, 15). Even when available, such data are rarely longitudinal: data about family structure and children’s well-being in non-Western contexts often consist of just a single wave of observations. Yet the effects of family structure on children’s well-being will likely be dynamic over the life-courses of focal children (34). Some events may have greater impacts for younger children. Other events could have constant—or even null—impacts across childhood. Some effects of family structure on early childhood outcomes may reverse at later ages, while others can become amplified at later ages (35). Without considering the entire period of dependency, the cumulative costs or benefits associated with a particular family form may be undetectable, and age-specific effects can be missed (36).

In much of the literature—in both marginalized and high-income populations alike—family-structure variables typically only code whether divorce, remarriage, or polygyny ever occurred during a given individual’s childhood, which can make fine-scale inference difficult. However, family structures often change dynamically during childhood—e.g., a child in a step-parent family is likely to have had an unmarried/divorced parent at some point, prior to the remarriage of their parent. Similarly, being born of a monogamous union does not exclude moving into a polygynous family, or vice versa. Reliance on point-estimates of family structure, when family structure is actually time-varying, can produce misleading inferences about the effects of family structure on outcomes (37).

Moreover, some parents may be more prone than others to belong to specific family structures—a phenomenon known as “selection bias” (38). Specific parental characteristics—like resource holdings, personality, or substance abuse issues—may affect both the probability of entering into a certain marriage state and the well-being of children. The causes of poor child well-being (e.g., parental alcoholism) may then be mistakenly attributed to the marriage state that parents with those characteristics tend to find themselves in (e.g., divorced). In some studies, selection bias can be addressed by directly testing hypotheses about the potential pathways linking family structure and child outcomes (see: ref. 14). Another approach involves the use of longitudinal data from families in which siblings have experienced varying lengths of a particular parental state (e.g., ref. 39); such sibling comparisons, however, assume that the effects of family structure are sibling-invariant.

If children are measured at multiple time points (i.e., when data are longitudinally recorded) and parents also transition between states frequently (e.g., when data are gathered from communities in which marriages may be unstable and/or a variety of family structures are permissible), then selection bias can be controlled for with more precision. In a relevant example, Winking et al. (19) implemented an intraindividual design to study the impact of polygyny on fertility among Tsimane’ parents. More generally, when data are recorded longitudinally (40), parent-specific random effects can be used to statistically control for the effects of time-invariant parental characteristics that are shared by all children of a given parent, while marriage-state variables (e.g., whether the parents of child i were divorced at age a) are allowed to vary both across children and within children across time. Observational studies cannot fully account for selection into marriage states, but our approach permits comparison of outcomes for focal children in different family structures, holding constant—if only statistically—the effects of parent identity. Although no observational study can provide causal inferences that are as robust as those of randomized controlled trials, in cases where randomized trials are unethical or infeasible (e.g., as in the case of intervening on parental marriage status) longitudinal studies with individual-specific random effects provide some of the best estimates possible, since they can attenuate time-invariant confounding related to parental identity (40).

## Pimbwe Childhoods.

To address some of the limitations to past research about children’s well-being and family structure, we present a longitudinal dataset collected over a 20-y period in a single village in Tanzania. In the village, marriages tend, at least for some individuals, to be quite transitory, and serial monogamy and polygyny are both practiced (24, 41). As a direct result of marital changes, households break up, and children may move in with a parent’s new spouse or other family members. Some individuals may even leave the village (either with or without their children), and return later, either with a new spouse or to live alone with relatives. As such, there is substantial variation in the family structure that is experienced by Pimbwe children. Many children experience an absent father, polygynous marriage, or step-parent presence at some point during their childhood. Moreover, there is substantial variation in family structure within the lifetimes of individual children.

To provide a relatively holistic picture of child well-being, we draw on four classic measures—survival, height-for-age (a measure of “stunting”), weight-for-height (a measure of “wasting”), and age-specific educational attainment. Prior research investigating the effects of family structure on children’s well-being has rarely assessed multiple aspects of well-being within the same set of individuals and has instead focused on a single outcome—e.g., survival or growth (but see: ref. 34). However, some variables may be more sensitive to variation in family structure than others. An impact of family structure on one aspect of well-being may affect other aspects of well-being in turn—e.g., deficits or advantages in growth could affect secondary variables, like educational attainment (42). By assessing multiple dimensions of child well-being concurrently, we attempt to provide a more thorough assessment of how different family arrangements might affect child outcomes.

Child height and weight—specifically, when operationalized as measures of stunting, wasting, and “underweight”—are globally utilized indicators of childhood well-being (43). Growth trajectories are affected by stressful living conditions, including poverty, malnutrition, exposure to infection, and psychological stress (43–45). Low height-for-age, a measure of stunting, can result from chronically insufficient nutrition, persistent infection, and/or psychosocial deprivation, and is thus indicative of adverse outcomes in childhood (43). By comparison, low weight-for-height—i.e., a measure of wasting—often results from nutritional shock or shorter-term deprivation (45). Similarly, child education is a commonly utilized indicator of child well-being, and, for Pimbwe children, it is likely an outcome of different forms of parental investment. Schooling is strongly believed to afford economic advantages in adulthood (for children who can obtain work in larger cities), but a stagnant rural economy in western Tanzania limits the potential economic returns to education in terms of local employment opportunities.

Sociodemographic data are often subject to various systematic biases, especially when collected from communities with low literacy rates and inadequate administrative support, where the state does not systematically record life events. Such biases may result from condition-dependent emigration, death, or divorce. Ethnographic methods may be particularly helpful in addressing these biases: during her visits over a 20-y period, M.B.M. sought to triangulate and cross-verify records of births, deaths, and marriages for all residents in the study village. Even so, missing and censored data remain. Typical approaches to dealing with unevenly sampled or missing data—e.g., restricting the sample to exceptionally well-observed individuals—can produce misleading results by undersampling unusual or unstable family configurations. Here, we implement statistical strategies designed to estimate age-specific effects of time-varying family-structure variables that also appropriately account for missing or incomplete data (see: ref. 46). Taken together, our data and analyses provide a methodologically robust test of the dynamic relationship between family structure and children’s well-being in a contemporary, rural, non-Western population.

## Results

**Fig. 1.**
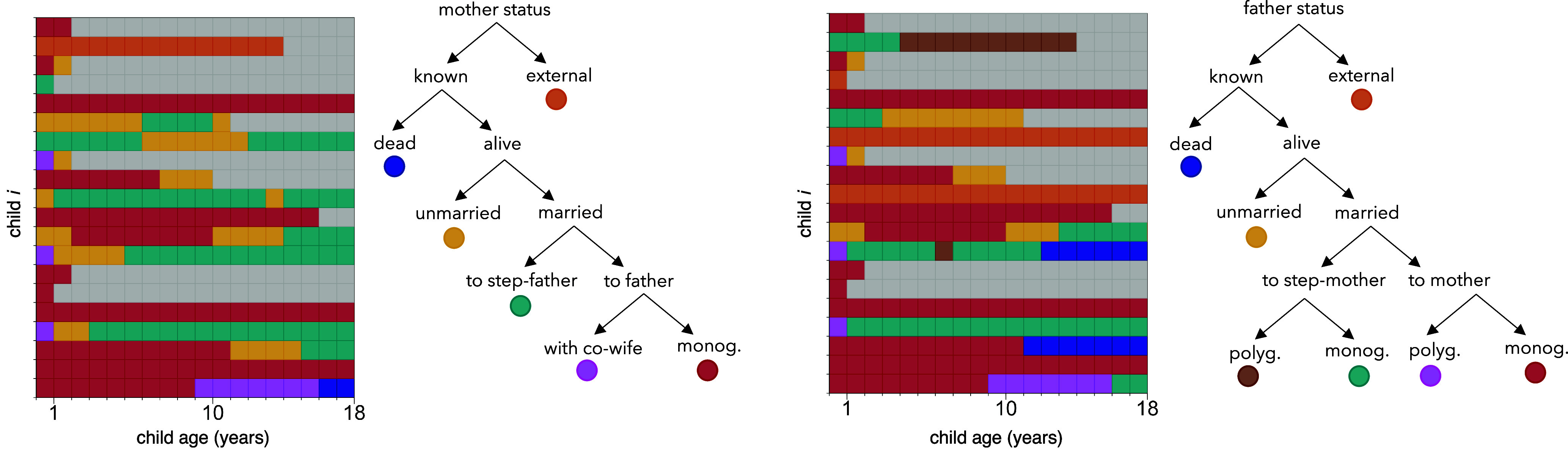
Description of parental marriage status during childhood. In the colored frames, each row represents a particular child’s life, and each square corresponds to a particular year—from ages 0 to 18—of that child’s life. Colors indicate a biological parent’s marital state in each year (mother’s state, *Left* panel; father’s state, *Right* panel). These plots represent only a slice of the complete dataset (i.e., trajectories of the same 20 randomly selected focal children). Gray boxes indicate censoring—i.e., when the child is deceased or is unobserved after a particular age. For example, a child born in 2009 could only be observed until the age of 5, because the last year of data collection was 2014. In another example, a child born in 1975 who died in 1977 could only be “observed” until the age of 2 (i.e, through data reconstructed from reproductive interviews with the parents).

We characterize the relationship between parent marriage status in a given year and child well-being in the same year by modeling both the association between biological mother status and child outcomes, and the association between biological father status and child outcomes. For each year of childhood (until age 18), we classify a child’s parent using a branching tree of marriage states (Fig. 1). During each year of childhood, a focal child’s biological parent may be deceased, unmarried, married to a step-parent (in polygyny or monogamy), or married to the child’s other biological parent. In several cases, we recorded a focal child’s parent as “external” to the dataset. This code is intended to reflect a state in which data about a focal parent’s vital or marital status is unavailable, typically because the parent never lived in the village, or left the village. In the vast majority of cases (1,030 of 1,302 cases), the external parent is a biological father.

**Fig. 2.**
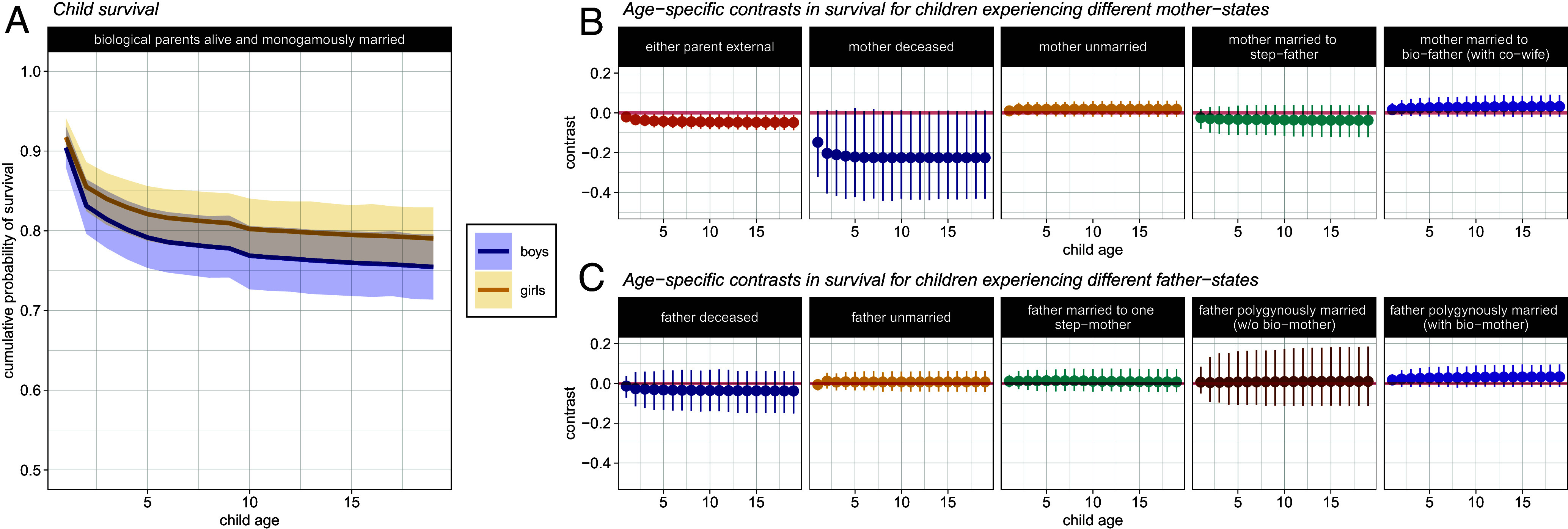
(*A*) Predicted cumulative probability of survival across childhood. The solid line plots the sex-specific means of the posterior prediction intervals for children with living, monogamously married biological parents. The shaded regions show the boundaries of the narrowest interval of the posterior that contains 90% of the probability density. Predictions for female children are shown in yellow and predictions for male children in blue. (*B*) Difference in cumulative survival probability (i.e., the contrast) at each age between a child whose biological parents are alive and monogamously married, and a child whose mother is in different states. (*C*) Difference in cumulative survival probability at each age between a child whose biological parents are alive and monogamously married, and a child whose father is in different states. Refer to Fig. 1 for the full branching tree of possible parental states during childhood.

**Fig. 3.**
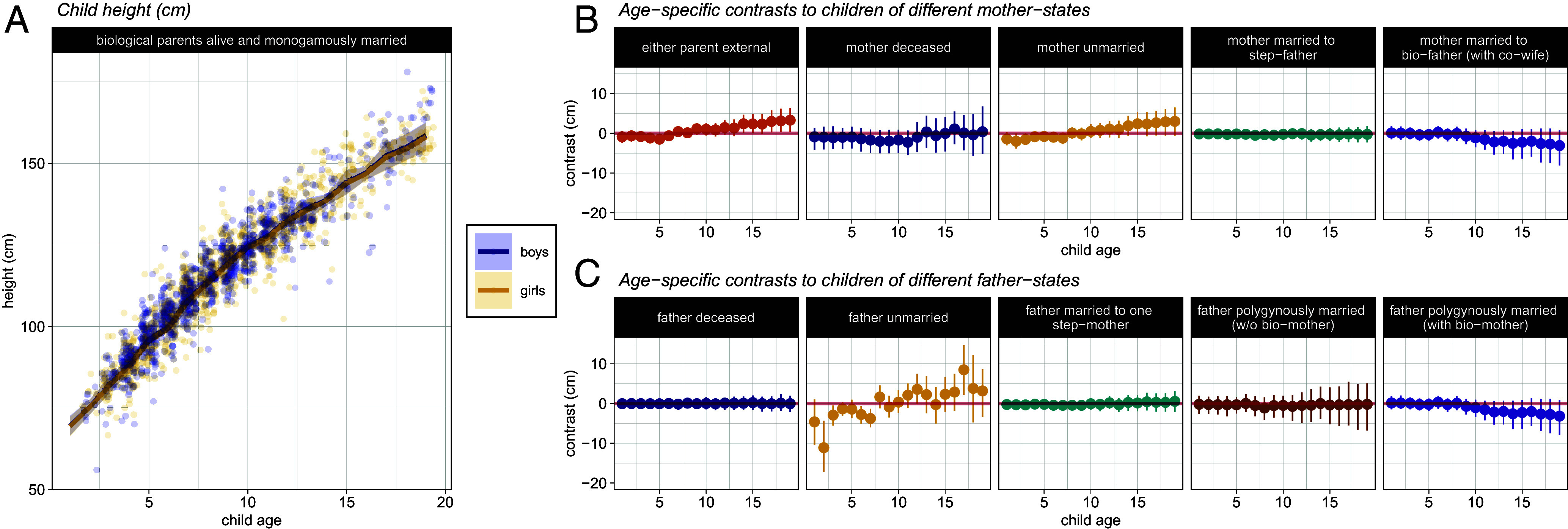
(*A*) Height across childhood, in centimeters. Lines and shaded intervals show the model’s predicted age-trajectories. Circles plot the measured heights at each age in our data (jittered for visualization). Data from girls and boys are plotted in yellow and blue, respectively. (*B*) Age-specific differences in height (cm) between a child whose parents are alive and monogamously married, and children of different mother-states. (*C*) Age-specific differences in height (cm) between a child whose parents are alive and monogamously married, and children of different father-states.

**Fig. 4.**
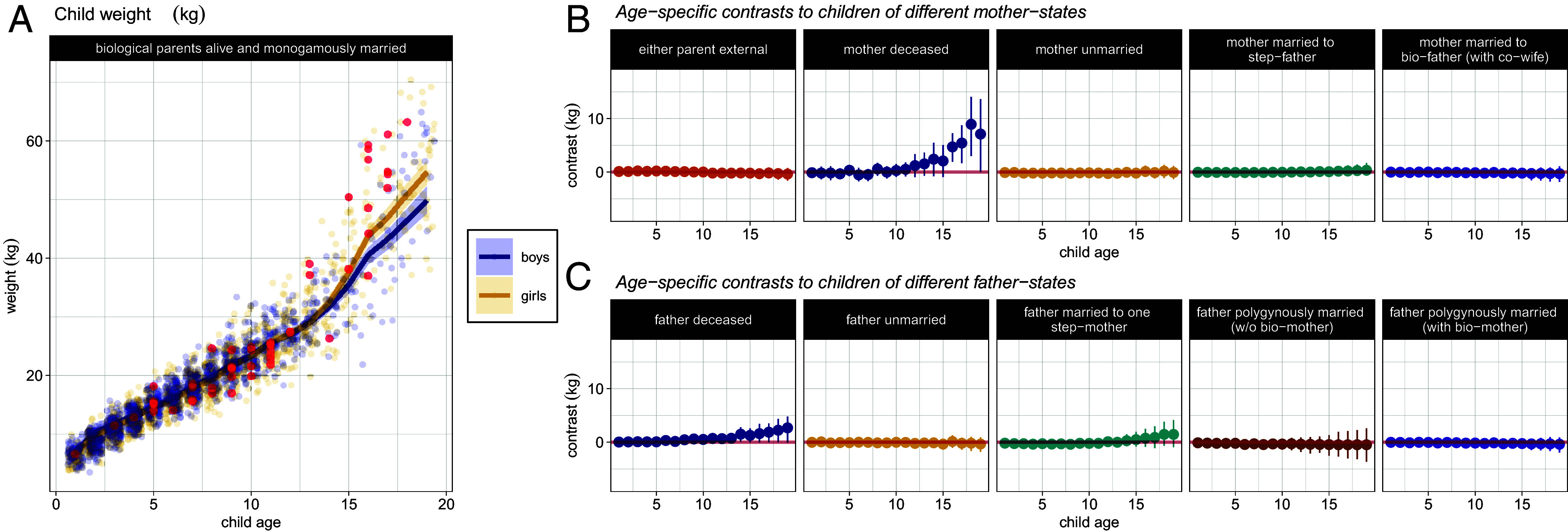
(*A*) Weight across childhood, in kilograms. Lines and shaded intervals show the model’s predicted age-trajectories. Circles plot measured weight at each age in our data (jittered). Data from girls and boys are plotted in yellow and blue, respectively. Points in red highlight weights recorded for children with deceased mothers, and illustrate that the model is responding to true structure in the observed data. (*B*) Age-specific differences in weight between a child whose parents are alive and monogamously married, and children of different mother-states. (*C*) Age-specific differences in weight between a child whose parents are alive and monogamously married, and children of different father-states.

**Fig. 5.**
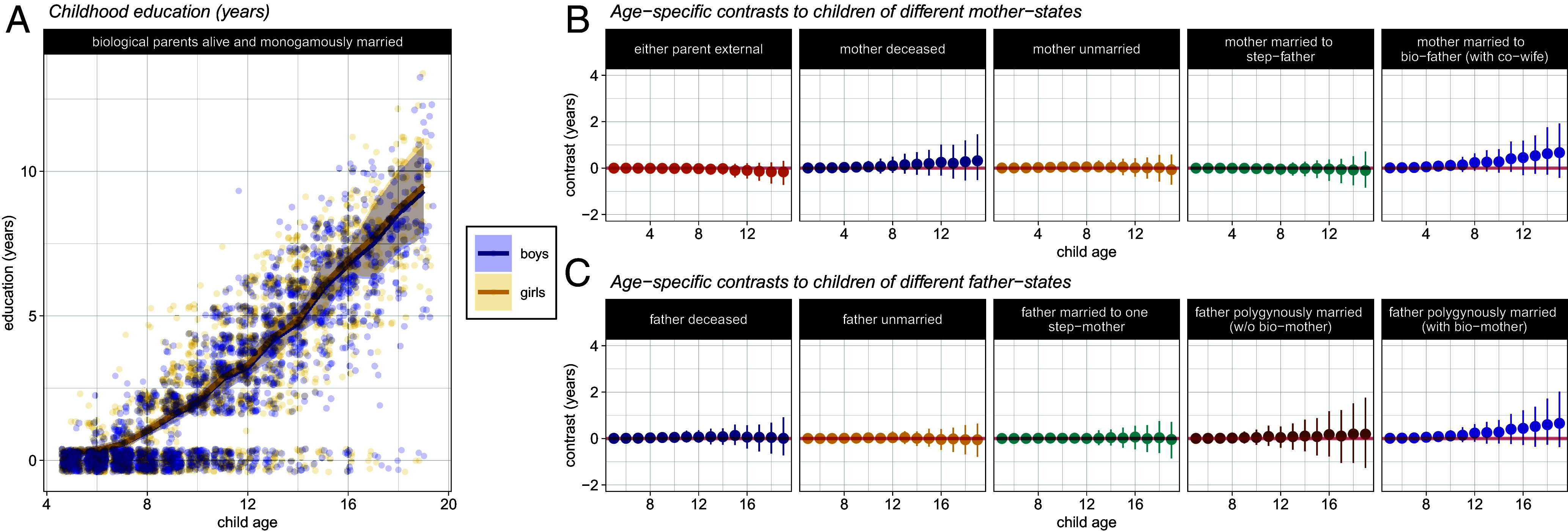
(*A*) Years of education completed during childhood. Lines and shaded intervals show the model’s predicted age-trajectories. Circles plot observed years of education at each age in our data (jittered). Girls are shown in yellow, and boys in blue. (*B*) Age-specific differences in education between a child whose parents are alive and monogamously married, and children of different mother-states. (*C*) Age-specific differences in education between a child whose parents are alive and monogamously married, and children of different father-states.

To visualize the effects of various parental states on child outcomes, we present contrasts in predicted outcomes (in natural units) for children of the same sex, but who experience different family structures (Figs. 2*B*–5*B* and 2*C*–5*C*). Our “mother-perspective” models allow us to contrast age-specific outcomes between a base case of a child with two living, monogamously married parents, and a child whose mother is either: 1) “external,” 2) deceased, 3) unmarried, or 4) married to a step-father. Likewise, our “father-perspective” models show the same contrast, but with a child whose father is either: 1) external, 2) deceased, 3) unmarried, 4) married to a step-mother in polygyny, or 5) married to the child’s biological mother in polygyny. We control for time-invariant, parent-specific characteristics by including random-effects terms for mother and father identity (see Supplementary Information for additional model details).

As statistical summaries, we report means and the boundaries of the 90% highest posterior density interval (HPDI). We present differences between posterior model predictions to assess whether children in different family structures experience different outcomes. For example, Fig. 2*C*: “father deceased” shows the difference in survival probability at each age between a female child whose father is deceased, and a female child whose father is alive. No difference—i.e., a value of zero—would indicate that children in different states do not experience different outcomes. The distance from zero allows us to infer the magnitude of a difference, if it exists, on the observational scale. Thus, we can determine whether a child who experiences a particular family structure appears to suffer disadvantage—and if so, at which point during childhood, and at what magnitude—relative to a child in a monogamous, two-biological parent family.

To gauge the credibility of results, we assess the narrowness of the posterior distributions (i.e., the HPDIs) of the contrast estimates. The width of these posterior intervals indicates the precision with which we are able to constrain the magnitude of potential effects; smaller intervals imply greater precision. In contrast to methods based on null-hypothesis significance testing, our Bayesian methods are informative about null effects; if the estimated effect size is close to zero—i.e., “no effect”—and the HPDIs are tight around zero, this is indicative that the data are highly inconsistent with the existence of substantive effect sizes. To ensure that our study is sufficiently powered to detect substantive effects if they are present, we also conducted a simulation study to assess the range of detectable effect sizes, for each of the parameters of interest, for each of the outcome variables we analyze here (see *SI Appendix* for details).

### Survival.

Our data indicate that children of all family structures in Mpimbwe face an increased risk of mortality at earlier ages (Fig. 2*A*). A child with a mother or father who is external to the family/village is predicted to experience a further small but consistent reduction in survival probability at early ages (Fig. 2*B*). This corresponds to a reduction in cumulative probability of survival to age 19 of about −0.04 (90% HPDI: −0.07, 0.00), compared with a child whose parents are known to be alive and monogamously married.

The death of a mother in the first few years of life is associated with a substantial reduction in probability of survival (Fig. 2*B*). A female infant with a deceased mother is predicted to survive her first year with mean probability 0.78 (90% HPDI: 0.62, 0.94), which is about 0.13 (90% HPDI: −0.01, 0.30) less than the probability that a female infant with two living, monogamously married, biological parents will survive her first year (mean: 0.92; 90% HPDI: 0.89, 0.94). The death of a mother in the first year of a child’s life results in a mean cumulative probability of surviving to age 19 of 0.58 (90% HPDI: 0.36, 0.81) for a female child, which is about 0.21 (90% HPDI: −0.01, 0.42) less than a child whose parents are alive and monogamously married throughout childhood (mean: 0.79; 90% HPDI: 0.75, 0.83). We observe no association between child survival and maternal marital status.

The death of a father is not associated with a substantial change in probability of survival at any age (Fig. 2*C*). In addition, the marital status of a father is not associated with child survival at any age. That is, there is no observable difference in the probability of survival, at any age, between a child with two living, monogamously married, biological parents, and a child with an unmarried father, or a child whose father is married to a step-mother in monogamy, or is polygynously married. With the exception of the category of fathers who became polygynously married to multiple women not including the biological mother of a given child (a category with very few cases of data), the HPDIs are quite tight around zero, indicating that the data exclude the possibility of numerically substantive effect sizes.

### Height.

Children with an external parent are predicted to be slightly taller at the end of the childhood period, corresponding to an increase in height of about 3.3 cm (90% HPDI: 0.35, 6.34), compared with children of monogamously married biological parents (Fig. 3*B*). At the end of childhood, there is some evidence that children whose mothers are unmarried are slightly taller than children of monogamously married biological parents. This corresponds to an increase of about 1.5 cm (90% HPDI: −0.01, 2.93). The marriage of a mother to a step-father with a co-wife (i.e., in polygyny) is associated with a decrease in height of about 3.01 cm (90% HPDI: −0.78, 8.05), though the effect is not reliably nonzero. There are no other clear associations between maternal status and child height.

Children of unmarried fathers are predicted to be shorter—until about age 6—than children whose biological parents are married; above the age of 10 this apparent height difference disappears. The predicted height of a 6-y-old male child with an unmarried father is 105 cm (90% HPDI: 101.3, 107.8), which is about 3.8 cm (90% HPDI: 1.35, 6.01) less than the height of a 6-y-old male child of monogamously married parents (mean: 108.5; 90% HPDI: 105.9, 111.1). Slight reductions in height appear to be associated with a child’s biological father being in a polygynous marriage, but the credible intervals here are wide and overlap zero, which indicates limited evidence of a reliable effect.

### Weight-for-Height.

A mother’s death is associated with higher weight-for-height at ages 15 to 18 (Fig. 4*C*). For example, a 15-y-old female of average height with a deceased mother is predicted to weigh 48.8 kg (90% HPDI: 45.9, 51.5), which is 5 kg (90% HPDI: 2.05, 7.78) heavier than a 15-y-old female with living biological, monogamously married parents.

Children whose fathers are deceased have slightly increased weight compared with children of monogamously married biological parents. This corresponds to an increase at the end of the childhood period of 2.9 kg (90% HPDI: −0.03, 5.3).

In *SI Appendix*, we visually compare Pimbwe growth trajectories with those of a global reference population as a method of model checking, reproducing Fig. 3. However, we contend that between-population differences in growth trajectories alone are not necessarily indicative of between-population difference in deprivation or nutrition, as human allometry is itself variable across populations (47, 48).

### Education.

For some children, the number of years of education begins to increase from about age 7, but some children appear never to attend school (Fig. 5*A*). We observe no differences in years of schooling, at any point during childhood, between children of different parental states (Fig. 5 *B* and *C*). The credible regions here are tightly centered around zero, and so the data are inconsistent with substantive effect sizes.

## Discussion

Across contexts, it is commonly argued that nonnuclear family structures—such as polygyny, single-parent households, and families with step-parents—exacerbate challenges for children (for discussion, see: refs. 16 and 49). In low-income contexts in particular, families may face social and economic challenges that could intensify the negative impacts of such family structures on child well-being. However, in these very same contexts, some researchers have found that deviations from monogamy (e.g., via divorce and remarriage) may also improve prospects for women and their children (22, 50, 51)—particularly if women are able to exert agency over such decisions. Assessing the validity and generalizability of past work is challenging, because much previous research–particularly in low-income contexts–that links family structure and child outcomes has been hampered by methodological issues. These issues include reliance on cross-sectional or aggregated data that fail to account for the dynamic nature of family structures and child development, and potential selection biases that obscure true causal relationships. Here, we have leveraged long-term data from an indigenous population in a rural Tanzanian village and developed statistical methods to address past methodological limitations. Our findings challenge some prevailing assumptions, revealing that, under certain conditions, and with respect to multiple key child outcomes, there is little evidence that nonnuclear family structures uniformly result in adverse outcomes for children.

### An Integrated View of Child Development.

The loss of a mother has repeatedly been shown to have a negative impact on the probability a child survives to adulthood [e.g., in rural Gambia, 1950–1974 (5), and in rural Tanzania, 1996–2012 (52)]. Given the critical importance of breastfeeding to a child’s nutritional status (53), it is unsurprising that the effects of maternal loss on child survival are greatest in the first few years of life (e.g., ref. 5). Our results similarly indicate that maternal loss is associated with reduced child survival, particularly in infancy. Although these results are not necessarily surprising, they demonstrate that our statistical methods are able to accurately recover the age-specific effect of a key variable, unambiguously known to affect child outcomes more acutely at specific ages.

In contrast, we detect no association between paternal death and child mortality, consistent with previous evidence (reviewed in: ref. 20). Pimbwe mothers typically compensate for father absence by securing support from other family or community members, including their own mothers. Paternal grandparents, or other kin of a deceased father, are also observed to provide assistance to mothers. However, children with an “external” parent are observed to have reduced age-specific survival prospects compared to children in monogamous two-parent families. External parents represent those individuals for whom either the identity and/or marital/vital status was not reported during interviews, most typically because these individuals left the village, or never lived there at all. In the vast majority of cases, the missing or external parent is a father. Substituted care is far less likely to be forthcoming if and when the identity of the father is unknown and/or no one in the village recognizes any kinship ties through the father. Similar reasoning may also account for the poor growth of young children whose fathers are unmarried. Unmarried or divorced Pimbwe men are often characterized by other Pimbwe as “lazy” or “heavy-drinking” (54); community members may be less inclined to support children whose fathers are alive, if those fathers themselves seemingly choose not to fully invest in their own children. Sociological accounts attribute the rather diffuse and unpredictable paternal roles among the inhabitants of Tanzania’s Rukwa region (including Mpimbwe) to its designation as a “labor reserve” in the early 20th century, when marriage and fatherhood were deeply disrupted by enforced outmigration of men (55).

While the impact of maternal death on child survival is well established, less work has explored how maternal death affects other aspects of development. Qualitative research in Africa has indicated that children without biological mothers may receive less care and psychological support from their caregivers and may be forced to undertake greater responsibility as children (56). Other studies have shown that maternal loss predicts less schooling-for-age (data from South Africa; 57). The fact that we do not observe much evidence to indicate maternal death negatively impacts metrics of well-being other than survival, whether early or later in childhood, is consistent with the idea that children who lose their mothers early in life—and survive—are well cared for by their more distant kin. Among Pimbwe, children who lose their mother typically live with a maternal aunt or grandmother. Some research in sub-Saharan Africa has suggested that families which adopt or foster children may be more wealthy or food-secure on average (58), and so orphans may not necessarily be disadvantaged in metrics of health or nutrition (59).

Indeed, we surprisingly observe maternal death to be strongly associated with increased weight of Pimbwe children in late teenage years. In some contexts, increased weight can be indicative of a poor diet, that is, a diet which is calorie-rich but nutrient-poor, but in Pimbwe villages, there are no products for sale (i.e., junk food) that might account for such a pattern; even sodas are rarely purchased. Moreover, a female child with a deceased mother is predicted to have a weight at age 16 of 48 kg—below the threshold for “obesity” (albeit by Western standards). An alternative explanation may be that higher weights of children with deceased mothers—and deceased fathers, to some extent—represent weights measured during pregnancies (a covariate we did not measure systemically or include in our models). Supporting this interpretation, high weights are largely recorded for female children (Fig. 4*A*, and see *SI Appendix*). In Western contexts, early pregnancy has been associated with factors such as maternal separation (e.g., in a longitudinal study of British women; 60) or adoption of children from their biological parents (e.g., in data from immigrant families in Denmark; 61). Pimbwe often attribute early pregnancies to “parents not maintaining sufficient control over their daughters,” and this finding may indicate that a mother’s death prompts teenagers to seek other forms of support earlier—e.g., by entering into an early marriage. An alternative possibility is noted by Strassmann (18), who found that Dogon girls who received less alloparental care tended to work more—in gardens, weeding, pounding, and lifting—resulting in accelerated growth rates.

We fail to detect any associations between paternal death and reductions in height, weight, or education. Other studies conducted among marginalized populations have demonstrated links between father presence and subtle indicators of well-being, such as improved marriage prospects (e.g., in a Martu Aboriginal community in Australia; 62), stunted height-for-age and low food security (e.g., among Tanzanian pastoralists; 58), or better educational attainment (e.g., in a Mosuo community in China; 63). Ethnographers nevertheless recognize that the specific reasons for paternal absence—e.g., whether paternal investment is normative and whether alternative sources of support are available—are likely to affect whether father absence has measurable impacts on well-being (20, 64, 65). Among Pimbwe, we suspect that as the child grows and starts schooling, both paternal and maternal kin compensate for a deceased father, although we cannot rule out the possibility that we have failed to identify small, but plausible effects (as, for example, were observed with the Bolivian Tsimane; 66).

Overall, we observe little evidence for differences in schooling between Pimbwe children as a function of parental marital structure, even though some children appear never to attend school. While primary school is nominally free, there are costs for uniforms, shoes, and stationery; secondary schools demand significant costs, particularly if the student is boarding. Furthermore, sending children to school brings some opportunity cost to parents, in the form of lost help with agricultural, childcare, and other household tasks. Although there is substantial variability in education among Pimbwe children (Fig. 5*A*), we speculate that economic considerations, rather than family structure, are the major explanatory factor here—future research, however, is needed to evaluate this idea.

### Adaptive Implications of Serial Monogamy.

Serial monogamy involves cycles of divorce and remarriage, and exposes children to step-parents. The presence of a step-parent has been associated with negative outcomes for children in Western studies—e.g., through a substantially increased risk of abuse or neglect (9, 67), for reasons such as increased family discord or competition for resources. Similarly, divorce has been associated with more subtle negative child outcomes, such as emotional distress, behavioral issues, or educational attainment (6, 68–70), both in Western contexts, and also in some developing regions, such as rural Bolivia (71). Increasingly, however, negative effects of divorce are shown to depend on the circumstances of the separation (e.g., ref. 72), or to be accounted for primarily by the economic disadvantages associated with being raised in a single-parent (typically single-mother) household (73). In some low-income settings, women may actually find themselves economically burdened by unemployed or underemployed husbands, who become financially dependent on them. In such situations, divorce and remarriage can be seen as strategies to alleviate these burdens, allowing women to secure better economic circumstances for themselves and their children (22, 25, 50, 51, 74–77).

Among the Pimbwe, the nature of marital relationships would seem to facilitate such strategic decisions. Pimbwe marriages are often informal, involving simple cohabitation (locally known as “kujipikia,” meaning “to cook for oneself or together”), and do not consistently require the payment of bridewealth. When bridewealth is paid, it is relatively modest (41). This informality reduces the costs associated with marriage and divorce, granting both men and women the freedom to marry, divorce, and remarry according to their needs. Indeed, women often initiate divorce (23), and a wife’s infidelity (much like a husband’s) does not necessarily lead to divorce (41). Divorces may result from a wide variety of disputes between partners, including disagreements over “theft” of shared goods or how labor responsibilities are allocated (23). Additionally, remarriage does not necessarily entail the coresidence of children from previous marriages with a step-parent, due to the residential flexibility observed among Pimbwe families. This system has long been perceived as allowing flexibility, even by early 20th-century missionaries, who noted the “fragility of the institution of marriage” among the Pimbwe (78).

From an evolutionary perspective, these practices might be adaptive, particularly for women. Prior research has suggested that serial remarriage could benefit Pimbwe women by enabling them to respond to unpredictability in the levels of economic support provided by husbands (24) and is consistent with evidence that Pimbwe women achieve fitness gains through remarriage (23). The lack of significant adverse effects on children in step-families that we observe here supports the idea that serial remarriage could be beneficial in this community. Future research might explore the mechanisms by which these benefits are realized, such as the potential expansion of social support networks through remarriage or optimization of trade-offs in spouse or relationship quality (79).

### Little Evidence that Polygyny Is Detrimental.

If data are aggregated such that underlying associations between poverty and marriage practices cannot be appropriately controlled for, polygyny may appear associated with worsened child well-being, even if it plays no causal role in producing such outcomes (see: ref. 30). However, if men vary substantially in their ability to provide resources, and women can choose their partners freely, choosing a polygynous husband may be beneficial for a focal woman, as such a marriage may provide a woman access to greater per capita resources (80). Accordingly, polygyny is expected to arise in cases where it improves reproductive outcomes for both males and females (i.e., under “the polygyny-threshold model”; 81, 82). Polygyny should only to lead to diminished measures of child well-being in cases where the assumptions of the polygyny threshold model are violated—e.g., in cases where women lack agency and men coercively marry more women than they can effectively support (29), or where there is discord among co-wives (for discussion, see: ref. 31).

Among Pimbwe, women are free to choose their husbands, and can easily dissolve a polygynous marriage. Both unmarried and married women can support themselves economically, through activities such as farming or beer-brewing, and women can gain access to land through their parents or the village leaders. Our data suggest that children of polygynously married fathers seem to have mostly equivalent outcomes to children of monogamously married fathers, across childhood. Polygyny is not associated with reductions in survival or education; slight deficits in height during late teenage years are observed, but they are not reliably nonzero. As such, there appear to be no substantial costs to polygyny in this population, implying that women may be making strategic decisions in choosing polygynous marriage as one of many options. This appears to be true even though polygynous marriage is increasingly practiced by men who have access to new sources of wealth—e.g., through business or political appointments—which women have less access to. Our findings here are thus consistent with the polygyny threshold model, which suggests that, at equilibrium, there should be no special advantages to children of either monogamous or polygynous unions in a closed population, since free partner choice normalizes the per capita resources available to children and damps inequality in female reproductive success (83). Alternatively, if male investment in children is generally rather limited, there may be no adaptive story at all (84), and polygyny may simply be the result of cultural transmission from prestigiously viewed neighboring cultural groups—e.g., the Sukuma (85).

### Toward an Improved Empirical Understanding of Diversity in Family Structure and Impacts on Well-Being.

The idea that men might increase their reproductive output by partnering with multiple women—e.g., under a system of a polygynous marriage or mating (83)—is widely recognized. A more subtle consideration is that women too may reap fitness benefits from reproducing with, or marrying, multiple men over their life courses (23). For example, extrapair paternities can yield social benefits for women (86, 87). Likewise, confusion of paternity (or beliefs in partible paternity) might help women garner care from additional providers (88–90). More generally, families can take many forms, from single parent homes, to Western-style nuclear families, to large multigenerational groups, and geographically separated but ever-connected networks of past and present partners.

As such, evolutionary scholars have puzzled over the origins, and current prevalence, of formal monogamous marriage systems and nuclear families (80, 91, 92). Indeed, the prominence of monogamous marriage in Western societies today may be driven in large part by the cultural transmission of norms regulating marriage, such as those propagated by state democracies and religious institutions (11). This may in turn explain why public health scholars have been quick to view this as a favorable marriage form (16).

In many parts of the world—and particularly in rural and indigenous enclaves in developing nations—monogamy may not provide special advantages. Families in such contexts may be bigger, more interdependent, and tend away from nuclear parenting, with many parties involved in supporting the health and well-being of children (1, 93, 94). In some cases, children routinely spend a majority of their time away from biological parents (95). Under such circumstances, cultural norms to ensure care in the absence of biological parents typically develop, thereby generating conditions under which neither divorce, nor remarriage, nor polygyny result in negative outcomes for children.

Our findings from Mpimbwe have particular significance for these debates. Instead of focusing on a single threat to child well-being (divorce, single-parent households, exposure to a step-parent, or to polygyny), we integrate these variables into a unified analysis of children’s first 18 y of life. Given that parents make marital decisions on the basis of their current circumstances—which will include their spouse’s contribution to the marriage, their own marital options outside of the marriage, and their access to kin support if choosing to remain as a single parent—it is perhaps not surprising that we do not observe substantial or consistent costs associated with varying family structures. Moreover, because Pimbwe children tend to drift residentially during childhood, moving between households of one or more biological parents, their grandparents, or a sibling of their parents, many of the challenges of a stereotypically fragmented family are mitigated, constantly, over time.

While we detect no appreciable costs to child well-being under various family arrangements, our research can provide only limited insight into the mechanisms by which transitions in family forms mitigate negative impacts for Pimbwe children. Decades of study indicate that the extent and patterning of parental investment in children depend on a myriad of factors, which may range from extrinsic mortality risk to opportunity costs to parents and the rival nature of resources (3). Interpreting our results here in the context of past work, several hypotheses about the ways in which Pimbwe women may improve their circumstances arise. For example, material wealth differences likely influence parental investment decisions. While cross-sectional analysis hints at effects on child survival (96), future research might explore how economic considerations dynamically affect parents’ marital decisions, and, in turn, child outcomes. Similarly, future work might consider whether aspects of partner quality—such as social status, emotional stability, or capacity to work, correlate with marital events and child outcomes. Given that costs of family transitions may vary for boys and girls, due to variable competition over household resources, or cultural norms, future work might extend our analytical framework to study sex-specific effects of parental states.

In our dataset, family structure is dynamic, but is only resolved annually. Our analyses therefore attempt to identify the age-specific effects of parental marriage states on child outcomes, with an annual level of specificity. However, it is also possible that a child’s poor health could lead to family disruption, and a child’s death could potentially cause a marriage to break up. Indeed, subannual data resolution may improve our understanding of such dynamics—although the results observed here are likely to remain robust, as we observe only 18 of 462 mortality event to have occurred in the same year as a marital dissolution (see *SI Appendix* for an additional robustness check). Similarly, because child outcomes like height and education are cumulative and potentially influenced by prior family structures, future research may expand our models to test for lagged effects of family structure, in order to better capture such dynamics (see *SI Appendix* for a brief exploratory analysis using a one-lag model). Limitations notwithstanding, our research here bolsters the idea that within particular social environments—e.g., where women have economic and social autonomy or are in scarce supply (97)—deviations from monogamous marriage may have smaller impacts on child outcomes than Western academic or public health perspectives would have us expect.

## Materials and Methods

### Ethnographic Details.

Data were collected as part of a long-term anthropological field study in a village in the Rukwa Valley of the Mpanda District of western Tanzania. The ethnographic details described here refer primarily to the time of study—i.e., between 1995 and 2014. The village is home to a population of approximately 2,500 indigenous people of primarily Pimbwe descent. Pimbwe have traditionally relied on the cultivation of cassava for subsistence; at present, households may also grow a variety of other crops, including tobacco, peanuts, maize, beans, sweet potatoes, and other vegetables for cash or subsistence. Crop yields tend to be unreliable, and the region is characterized by high levels of food insecurity (85, 98). Livelihoods are also supplemented through activities like hunting, fishing, carpentry, pottery, beer brewing, and basketry, and some people run small-scale kiosks or shops that sell products—such as batteries, oil, or matches. Infrastructure was (at the time of the study) rather poor relative to the rest of Tanzania, and the incidence of infectious disease high. Children attend primary school in the village, which is nominally free, but with considerable incidental costs (uniforms, shoes, stationery, etc.). Prior to 2007, secondary schooling options with some fees were available about 14 km away from the village. Post-2007, a secondary school opened about 7 km away, which was nominally free, but demanded significant ancillary costs.

Marriage is ubiquitous, but marriages tend to be rather unstable, such that divorce and remarriage are more common than the formation of lifetime partnerships. While marriages may be celebrated with ritual and dance (41), they are often marked solely by the sharing of a household. Pregnancy often precipitates a marriage. Pimbwe women may be thought of as relatively autonomous in making marriage decisions (24). After the dissolution of a marriage, young children (under the age of approximately 8 y) tend to remain with their biological mothers; older children will sometimes live with their biological fathers. Polygynous marriage—in the early 20th century—was reportedly restricted to village chiefs (41). Our data indicate that the rate of polygynous marriage has remained at about 10% of marriages, since the 1950s (*SI Appendix*). Ethnographic observations during fieldwork between 1995 and 2014 suggest that polygyny is now aspired to by men who are ambitious, including shopkeepers, entrepreneurs, and those with political influence (see *SI Appendix* 23). Co-wives almost never coreside, and often resent one another. Women may also enter into a polygynous relationship without moving away from the house in which they were previously residing in (when single or divorced).

Newly married couples are equally likely to live close to the husband’s or wife’s parents and/or their siblings, although about 30% have no close kin in vicinity. Mothers can typically rely on the assistance of one or more coresident older children to care for young children; support from other relatives nearby, such as the mother’s own mother, aunt, or sister can be enormous, but is far from assured (99). Furthermore, there is some indication that children in families with large number of relatives in the village have lower survival (96). Some research reports that social relationships among Pimbwe might be strained due to widespread mistrust and frequent accusations of witchcraft (79). Pimbwe children grow rather slowly, and suffer from poor nutrition relative to other groups in the region. For additional details on the study site, see refs. 41, 79, and 100.

### Data and Sampling Frame.

Fieldwork was conducted in the years: 1995, 1996, 1998, 2000, 2002, 2004, 2006, 2010, 2012, and 2014. All interviewed individuals provided informed consent verbally, following approved IRB protocols (UC Davis Institutional Review Board 436682-4; see *SI Appendix* for further details about the consent process). Data were collected by M.B.M., usually in the company of different research assistants recruited from the village across the years, either in ki-Swahili or ki-Pimbwe, depending on the interviewee’s preference. Generally interviewees eschewed the suggestion that interviews be conducted in privacy, and invited the researchers to sit outside the house in a patch of shade. No attempt was made to gender match interviewer and interviewee; this was not deemed necessary as there is very little gender segregation in the Pimbwe community.

During each round of fieldwork, complete reproductive histories were collected for all reproductive-aged individuals currently residing in the village. At the time of data collection, longitudinal records of deaths, births, marriages, and divorces were recorded for each individual. In addition, the heights, weights, and years-of-schooling of all children in each household were recorded. Across the many rounds of interviews, inaccuracies or inconsistencies were corrected and queries followed up on. Every household within the village was censused in each year, with the exception of 2014, when fieldwork could not be completed due to illness. Summaries of our key data are presented in *SI Appendix*, Tables S3 and S4.

#### Survival data.

During reproductive interviews conducted between 1995 and 2014, individuals reported births/deaths for all of their children (including those who were born and/or passed away before the year 1995). Children who passed away before 1995 were never observed by M.B.M. (n = 515, see *SI Appendix*), but such records are still useful for estimating survival outcomes when paired with marriage histories reported by the same focal respondents. The earliest birth year recorded for a child during a reproductive interview was 1931. Our full survival database contains records on 3,693 children born between the years 1931 and 2014. The sex of the child is unknown in 278 cases, due primarily to respondents not remembering the sex of children who died during infancy many years ago (see *SI Appendix* for further details).

#### Height, weight, and education data.

Data on child height, weight, and education were collected at each round of data collection—in 1995, 1996, 1998, 2000, 2002, 2004, 2006, 2010, 2012, and 2014. Height measurements were recorded in centimeters using a SECA-brand portable height measure, and weight was recorded in kilograms using a SECA-brand digital scale. Children aged 5 and above (or their parents) were asked how many years of school they had attended until that point.

At least one anthropometric measurement was recorded for 881 children; 454 children were measured between 2 and 7 times; we utilize a total of 1,744 height/weight records across all child-years. Education data was recorded at least one time for 1,370 children; 921 children were measured between 2 and 9 times; we utilize a total of 3,693 education records across all child-years.

#### Parental marriage status data.

When information on parent identity is available, we first classify mothers as being alive or dead in a given year. We then classify living mothers into married or unmarried categories. Married mothers are further classified as either being married to a focal child’s biological father or to a step-father. We similarly classify the marriage states of a focal child’s father in each year of childhood—but the branching tree of marriage states is slightly more complex, to accommodate polygynous marriage categories (Fig. 1).

In some cases, limited information was available about the status of a child’s biological mother or biological father (*SI Appendix*, Table S3). Typically only a name and/or a residential location of the missing parent was available; even if the parent appeared in the dataset one or more times during the 20 y study, follow-up information on vital or marital status was not available throughout the focal child’s life, most likely because this parent had left the village (or never lived there at all). We do not remove these observations from our analyses (see: ref. 46). Excluding missing data can introduce substantial bias in a sample—for example, if the data are missing for a reason which also affects the outcomes or variables of interest. Children with a parent who never lived in the village, or left during childhood, but for whom no (or limited) information was made available during reproductive interviews, presumably represent the most marginalized subset of individuals in our sample. These children are less likely to receive support (financial or emotional) from that parent (or indeed from his or her relatives or friends). It is therefore critical to distinguish children in this category from children of deceased parents. We categorize the parent status in these cases as external, to represent that the parent is external to the child/family/household/village. We then model the effect of this category of outcomes as a type of parental marriage-state. We model a single external parent category, rather than separating external parents into mothers and fathers, because of the small number of occurrences of missing mothers (*SI Appendix*, Table S3).

### Statistical Analyses.

To investigate the association between the marital status of mothers and child survival, we model whether child i survives year t, $S_{\left[i,t\right]}\in \left\{0,1\right\}$, conditional on having survived year t−1, as a Bernoulli distributed outcome with probability $\theta_{\left[i,t\right]}$:

[1] $$S_{\left[i,t\right]}∼\mathrm{Bernoulli}(\mathrm{logistic}\left(\theta_{\left[i,t\right]}\right)){|}_{S_{\left[i,t-1\right]}=1},$$

[2] $$\begin{array}{cc} \theta_{\left[i,t\right]} & =\alpha +\gamma_{\left[O_{\left[i\right]}\right]}+\epsilon_{\left[Y_{\left[t\right]}\right]}+\kappa_{\left[Q_{\left[i\right]}\right]}+\eta_{\left[J_{\left[i\right]}\right]}+\beta_{\left[1,A_{\left[i,t\right]}\right]}\\ & \;+\beta_{\left[2,A_{\left[i,t\right]}\right]}M_{\left[i\right]}+\beta_{\left[3,A_{\left[i,t\right]}\right]}T_{\left[i\right]}+\beta_{\left[4,A_{\left[i,t\right]}\right]}F_{\left[i,t\right]}\\ & \;+\beta_{\left[5,A_{\left[i,t\right]}\right]}X_{\left[i,t\right]}+\beta_{\left[6,A_{\left[i,t\right]}\right]}W_{\left[i,t\right]}\\ & \;+\beta_{\left[7,A_{\left[i,t\right]}\right]}Z_{\left[i,t\right]}+\beta_{\left[8,A_{\left[i,t\right]}\right]}P_{\left[i,t\right]}+\beta_{\left[9,A_{\left[i,t\right]}\right]}U_{\left[i,t\right]}, \end{array}$$

The log-odds of survival, $\theta_{\left[i,t\right]}$, is a function of age-specific parameters and time-varying covariates for child i. The parameter α is a global intercept, γ is a random effects vector to measure change in survival as a function of birth-order, O, ϵ is a random effects vector to measure change in survival as a function of year of measurement Y, κ is a random effects vector to measure change in survival as a function of mother identity, Q, and η is a random effects vector to measure change in survival as a function of father identity, J.

Next, we model a set of age-specific parameters, represented by β. The first three terms model *time-invariant* data; $\beta_{\left[1,\right]}$ gives age-specific intercept parameters, $\beta_{\left[2,\right]}$ represents the effect of being male on survival at each age, and $\beta_{\left[3,\right]}$ represents the effect of being a twin on survival at each age. The $\beta_{\left[2,\right]}$ parameters control for the sex of the child, and the $\beta_{\left[3,\right]}$ parameters control for the twin-status of the child. The remaining terms model *time-varying* data. Specifically, $\beta_{\left[4,\right]}$ represents the effect of the absence of a biological father due to death on offspring survival, $\beta_{\left[5,\right]}$ represents the effect of the absence of a biological mother due to death on offspring survival, $\beta_{\left[6,\right]}$ represents the effect of a biological mother being unmarried on offspring survival, $\beta_{\left[7,\right]}$ represents the effect of a biological mother being married to a step-father on offspring survival, and $\beta_{\left[8,\right]}$ represents the effect of a biological mother being in a polygynous union on offspring survival. Finally, the parameter $\beta_{\left[9,\right]}$ represents the effect of having an external parent on child survival. The function $A_{\left[i,t\right]}$ returns the age of individual i in year t.

We model the random effects vectors γ, ϵ, and β in each model using Gaussian process submodels. This model structure allows, for example, the age-specific effects (i.e., effects of being male, being a twin, and all parental states) to take on arbitrary functional forms (see *SI Appendix* for details) with respect to age while still sharing information across neighboring age-categories. More specifically, the Gaussian process approach: 1) allows for partially pooled estimation of parameters, 2) imposes no a priori functional form (e.g. linear, quadratic, etc.) on random effects, and 3) allows for a reduction in parameter complexity of the model by reducing the extent to which neighboring random effects parameters can vary independently.

To investigate the association between the marital status of fathers and child survival, we specify a similar model, but include additional parameters that capture the association between paternal marriage status (i.e., monogamy or polygyny) and child outcomes. Because we do not estimate a sex-specific effect of parental state on the outcomes (i.e., model an interaction between the sex of a child and the effects of family structure), our model assumes that the effect of family structure on child outcomes is the same for boys and girls. For example, our survival model estimates an age-specific mean survival probability that is different for boys and girls, but the offset for mother unmarried is the same for both boys and girls.

To characterize the association between parental status and other child outcomes, we specify the same sets of age-specific regression parameters, but modify the outcome distribution as appropriate. We model height-for-age using a Bayesian Log-Normal regression approach, where the logarithm of the height in centimeters of child i in year t, $H_{\left[i,t\right]}$, is a Normal distributed outcome:

[3] $$\log\left(H_{\left[i,t\right]}\right)∼\mathrm{Normal}(\theta_{\left[i,t\right]},\lambda ).$$

We model the weight of child i in year t, $K_{\left[i,t\right]}$, as a Log-Normal distributed outcome, controlling for height with an additional age-specific parameter:

[4] $$\log\left(K_{\left[i,t\right]}\right)∼\mathrm{Normal}(\theta_{\left[i,t\right]},\lambda ).$$

We model years of schooling, for child i in a year t, $E_{\left[i,t\right]}$, with a zero-inflated Poisson model:

[5] $$E_{\left[i,t\right]}∼ZI\_Poisson(\mathrm{logistic}\left(\theta_{\left[i,t\right]}\right),\exp\left(\eta_{\left[i,t\right]}\right)).$$

In the case of the zero-inflated Poisson model, the linear models on both θ and η are defined as functions of the same set of predictors as in the height, weight, and survival models. The linear model on θ measures the probability that a child, i, does not report a single year of school attendance at year t, and the linear model on η estimates the cumulative number of years of schooling of child i in year t, conditional on child i having begun schooling in or before year t. We specify weakly regularizing priors on all intercept, slope, and variance parameters (see *SI Appendix* and Stan code at https://github.com/rianaminocher/pimbwe-child-outcomes).

We provide our dataset and code so that interested readers can reproduce our reported results—and should they choose to—modify or extend the models, or visualize the results using alternative approaches. See *SI Appendix* for additional modeling details, and https://github.com/rianaminocher/pimbwe-child-outcomes.

## Supplementary Material

Appendix 01 (PDF)

## Data Availability

Anonymized tabular data have been deposited in GitHub (https://github.com/rianaminocher/pimbwe-child-outcomes) (101).
