# Supplementary material for: Little evidence that nonmonogamous family structures are detrimental to children’s well-being in Mpimbwe, Tanzania
Source: Proc Natl Acad Sci U S A. 2024 Dec 20;121(52):e2407785121. doi: 10.1073/pnas.2407785121 (PMC11670189; doi:10.1073/pnas.2407785121)
Supplement: Supplementary file 1 — Appendix 01 (PDF) [file pnas.2407785121.sapp.pdf]

PNAS

Supporting Information for

Little evidence that non-monogamous family structures are detrimental to children's well-being in Mpimbwe, Tanzania

Riana Minocher, Monique Borgerhoff Mulder, and Cody T. Ross

Riana Minocher
E-mail: riana.minocher@gmail.com

This PDF file includes:

Supporting text
Figs. S1 to S90
Tables S1 to S39

Supporting Information Text

1. Data

Consent process. All interviewed individuals provided informed consent verbally, following approved IRB protocols. The details included in the most recent IRB protocol which addressed the consent process are as follows:

"Written consent is not appropriate in this population for several reasons. Most pertinent is the low level of literacy. However much more problematic is fear that villagers have of signing any document. With the expansion of microloan programs in rural Africa, villagers are beginning both to understand (and fear) the power of a signature (with respect to subsequent litigation). In addition, the only times they have sign their names is for issues of state (registering to vote) or legal cases. A researcher presenting a consent form for signing would in many instances be distrusted, or viewed as a government functionary. I therefore argue that this research could not practically be conducted without waiving the signed consent forms. I do however propose an alternative. On arrival at each household we will reiterate the explication of our research goals and methods made at the original village meeting. We then ask whether the household is willing to participate, whether the adults present are willing and have time to answer our questions (we will give some examples of our questions – although from prior experience it seems that households will have learnt about the questions from neighbors). We then explain that it is quite OK to refuse to answer any of the questions, or to make any comments on (or objections to) our protocol to any of the village leaders. In earlier work this discussion has taken, typically, less than a minute – consent was forthcoming. Accordingly I make sure at various junctures in the interview if the subject is comfortable with proceeding. For example "Now I have some questions about children who have died. Are you happy with continuing to answer?" This seems in the past to have worked well, reminding subjects to realize that they can reconsider participation. Interviews and consent discussion are necessarily conducted in Ki-Swahili (the lingua franca of Tanzania) or Ki-Pimbwe. I am near fluent in Swahili and have passable knowledge of Ki-Pimbwe."

Sampling frame and additional details. Our survival database contains records on 3,693 children born to 662 mothers of known marital/vital status and 461 fathers of known marital/vital status. For 272 children, the status of the child's mother was unresolved (coded as "external") throughout the period of study and for 1,030 children, the status of the child's father was unresolved (coded as "external"). 1,709 children were recorded as female, 1,706 as male, and the sex of the child was unknown in 278 cases. 192 children were recorded to be twins. Figure S1 shows the distribution of years of birth for children in our sample.

All 3,693 children were included in our survival models (see: Tables S1—S2). Some children lived before the first year of fieldwork (i.e., the year 1995). Demographic data about these children (year of birth, year of death, or year of censor) were reported during reproductive interviews conducted with their parents. Figure S2 plots the first 18 years of life of all 3,693 children in our survival database, ordered by year of birth.

When modeling height, weight, and education data, we utilized measurements which were made repeatedly during the sampling period (1995-2014). Some children were measured more frequently than others. Figure S3 shows the frequency distribution of height, weight, and education observations available for each child. Sample size information is shown in Tables S3 and S4.

Missing parent information. When data on the status of a child's mother or a child's father was missing, we modelled an additional parameter to describe the association between this form of parental status and child outcomes. In these cases, the identity of the parent may have been truly unknown (e.g., the reporting adult would not say or did not know), or only a name and residential location (outside the village) were reported. Some cases corresponded to individuals who had been recorded at one point in the Pimbwe database, but for whom no subsequent vital/marital status data were available, most likely because of emigration from the village. Children with a parent of such status likely do not receive any support (financial or emotional) from that parent (or indeed from his relatives or friends). Therefore, it is important to distinguish children in this category from children of deceased parents. Children whose parents are deceased—i.e., known to be deceased, and known to their families and to other community members—may receive forms of assistance from the relatives or friends of the deceased. For the purpose of our statistical models, we assigned each missing parent a unique ID. Some missing parents may have mothered/fathered more than one child in our database. Because this information is not available to us, we make a conservative assumption that each missing parent is unique.

Prevalence of polygyny. The frequency of polygynous marriage in the population is about 10% of marriages, across the years 1950-2014 (Figure S4).

2. Extended results

Male, twin, birth-order, and year-specific effects.

Survival. A male child has a slightly lower probability of surviving the first two years of life compared to a female child, but at later ages, there is no clear evidence of sex-specific mortality (Figure S5). We observe a mean reduction in the probability of survival to age 2 for a male child versus a female child of about 0.02 (90% CI: 0.00, 0.04). A twin child has a substantially reduced probability of survival, compared to a non-twin child, until about the age of 5 (Figure S5). For males, this corresponds to reduction in survival probability to age 5 from 0.788 (90% CI: 0.735, 0.835) in the base case, to 0.647 (90% CI: 0.534, 0.746). For females, this corresponds to reduction in survival from 0.818 (90% CI: 0.769, 0.860) in the base case, to 0.691 (90% CI:

0.584, 0.784). We observe no difference in the predicted probability of survival for children of different birth-orders (Figure S6). There is a slight secular trend of increasing survival probability with year of measurement (Figure S6).

Height. We do not observe a height difference between female and male children during childhood (Figure S7). However, when we plot heights measured across childhood and adulthood for individuals in our sample, we observe a visual difference between male and female individuals (Figure S9). The difference appears above the age of 20.

In order to visually compare child height in the Pimbwe population studied here with comparable data from a global reference population, we downloaded relevant records from the World Health Organization (WHO) Child Growth Standards web-page (?). We plot these reference height-for-age curves alongside the estimates produced by our statistical models (Figure S10). The WHO standards were developed using data from the WHO Multicentre Growth Reference Study (?) and cannot be expected to perfectly fit data from every human population (for discussion, see: ?). We compare these data to ensure that our results are reasonable in light of a previously validated set of growth trajectories, but recognize that such measures may not necessarily proxy deprivation or poor nutrition in all world populations (?). When visually comparing Pimbwe growth trajectories with those recorded by WHO standards, it appears that Pimbwe growth trajectories may be delayed. Indeed, delayed growth trajectories have been reported for neighbouring African populations, e.g., the Turkana (?).

We observe no strong association between child height and twin-status, birth-order, or year of measurement (Figure S7—Figure S8).

Weight-for-height. We observed maternal death to be strongly associated with increased weight-for-height of teenage Pimbwe children—this was a result we did not predict. High weights are largely recorded for female children. Early pregnancy has been associated with maternal separation or loss in a number of populations and contexts, so we speculated that our results may reflect a similar pattern. To visually inspect this hypothesis, we paired weight data for girls with data on pregnancy (Figure S13), and highlight whether or not a particular observation was recorded at the time of pregnancy or lactation. At least two observations of pregnancy/lactation co-occur with the loss of a mother and high weight (Figure S13).

However, we do not include data on pregnancy/lactation as a predictor of child weight in our models. This is not a hypothesis we set out to investigate *a priori*. Additionally, a number of factors might affect the relationship between parent status and weight, including the pregnancy status of a child's mother or siblings. A more detailed study of child weight-for-height trajectories might investigate the role of these factors.

Education. We observe no association between years of schooling reported and the sex of a child, twin-status, or birth-order (Figures S14—S15). Education levels have been increasingly slightly since the late 1990s (Figure S15).

3. Additional details on models

All analyses and data-processing procedures were performed in R (version 4.3.2, ?) and Stan (version 2.35.0, ?). The results that we present here are summaries of 4,000 sampling iterations from each of 4 MCMC chains. We assess model convergence and mixing with the 4th version of the $\hat{R}$ convergence diagnostic as implemented in Stan (?), the estimate of the autocorrelation-adjusted number of samples, and visual inspection of the trace plots of all relevant parameters.

Gaussian process priors. We model the random effects vectors γ, ϵ, and β in each model using Gaussian Process functions, which allow for birth-order-, year-of-observation-, and age-specific effects (i.e., the effects of being male, being a twin, and all parental states) to take on arbitrary functional forms, while still partially sharing information across neighboring parameters. We let $d \in \{1, ..., 9\}$ index the nine age-specific coefficient vectors that are used to estimate covariate effects. Then, we model:

$$\beta_{[d]} \sim \text{Multi. Normal Cholesky}((0, \dots, 0)', \eta_{[d]} L_{[d]}) \quad [1]$$

$$\gamma \sim \text{Multi. Normal Cholesky}((0, \dots, 0)', \eta_{[10]} L_{[10]}) \quad [2]$$

$$\epsilon \sim \text{Multi. Normal Cholesky}((0, \dots, 0)', \eta_{[11]} L_{[11]}) \quad [3]$$

where $\eta_{[d]} \in (0, \infty)$ serves to scale variance, and $L_{[d]}$ is a factor given by the Cholesky decomposition of the correlation matrix $\rho_{[d]}$. The correlation matrices are defined using a distance decay function:

$$\rho_{[d,i,j]} = \kappa_{[d]} \exp\left(-\tau_{[d]} \frac{(i-j)^2}{C_{[d]}^2}\right) \quad [4]$$

where $\kappa_{[d]} \in (0, 1)$ controls the maximum correlation, $\tau_{[d]} \in (0, \infty)$ controls the decay rate, and $C_{[d]}$ is a constant which normalizes the maximum distance between categories to 1.

Using a Gaussian Process to generate random effects offers several benefits: 1) it allows for partially-pooled estimation of birth-order effects, year-specific trends, and age-specific effects (of each covariate), 2) it imposes no *a priori* functional form (e.g. linear, quadratic, etc.) on these random effects, and 3) it allows for a reduction in the parameter complexity of the model by reducing the extent to which neighboring random effects parameters can vary independently.

We use weakly regularizing priors on the parameters that control the Gaussian Process:

$$\eta_{[d]} \sim \text{Exponential}(1) \quad [5]$$

$$\tau_{[d]} \sim \text{Exponential}(1) \quad [6]$$

$$\kappa_{[d]} \sim \text{Beta}(12, 2) \quad [7]$$

Other model priors. We define weak priors over the other top-level parameters. In the survival model, we define a vague prior on the intercept which, when passed through the logistic link function, implies that mortality is fairly rare:

$$\alpha \sim \text{Normal}(2, 2) \quad [8]$$

while the wide standard deviation of 2, however, ensures that the prior is not informative.

In the other models, we also use a vague normal prior on the intercept:

$$\alpha \sim \text{Normal}(2, 2) \quad [9]$$

In the height and weight models, this intercept term represents the log height or weight value of an individual at birth. As such, this prior is very flat across the feasible range of such values in humans.

We also give a weak prior suggesting that children of unknown sex are male with about 50% probability:

$$P_{male} \sim \text{Beta}(2, 2) \quad [10]$$

Parameter traceplots and summaries. We fit eight models—describing the association between each of four child outcomes (survival, height, weight, education) and the status of each biological parent (mother or father). Traceplots for all relevant parameters of each model fit are shown in Figures S16—S23. Numerical summaries of estimated parameter values are shown in Tables S5—S14.

4. Supplementary robustness checks

Parent vital/marital status at "t-1". Our analysis measures family state in the same year as child outcomes are measured. Thus, our models quantify the association between a parent state in year t and child outcomes in the same year t. It is possible that past family structures have lagged effects on child well-being. For some child well-being measures in particular, such as psychosocial health measures—which we do not study here—early childhood experiences may be critical determinants of behaviour in adulthood. Our analysis does not attempt to systematically quantify lagged effects of parental states on child outcomes. However, we conducted a brief robustness check to assess whether there is a correlation between child outcomes and past parental states. We measure parent status in the year $t - 1$, i.e., a year before the child outcome is measured. The results of this robustness check indicate that the effects observed are restricted to analysis of annual resolved child outcomes and parent states in the same year, t. It is important to note that the results of this robustness check do not exclude the possibility that parent states have lagged effects on child outcomes at all; to address this question thoroughly would demand a more complex analytical approach, which is beyond the scope of the analysis presented in the current paper.

Comparison of results. The results of survival analyses of the "t-1" robustness check are presented in Figure S24. Full fitted model summaries and traceplots are shown in Tables S15-S16 and Figures S25-S26. We observe no association between maternal vital/marital state and child survival. The loss of a father in the year prior to measurement of child survival appears associated with an increased survival probability for infants. This may imply that the loss of a father is compensated by care from other relatives in the years following his death, as ethnographic evidence also suggests. Polygyny in the year prior is associated with increased probability of survival for children, at an early age. It is possible that benefits of polygyny are only realized for children after living in the arrangement for a period of time. The association between "either parent external" and child outcomes observed is identical as in the analysis presented in the main paper, which is expected, given that this parental state is not time-varying.

The results of height analyses of the "t-1" robustness check are presented in Figure S27. Full fitted model summaries and traceplots are shown in Tables S17-S18 and Figures S28-S29. No associations between parent vital/marital status at $t - 1$ and child height at t can be observed.

The results of weight analyses of the "t-1" robustness check are presented in Figure S30. Full fitted model summaries and traceplots are shown in Tables S19-S20 and Figures S31-S32. The same associations observed in the analysis of parent vital/marital states at t (i.e., results in the main text) are observed here.

The results of education analyses of the "t-1" robustness check are presented in Figure S33. Full fitted model summaries and traceplots are shown in Tables S21-S24 and Figures S34-S35. No association between parent vital/marital status and child education can be observed.

Parent vital/marital status at "t+1". Because our data is resolved to the year of observation, rather than month or day, it is difficult to exclude the possibility of "reverse causality". It is possible, for example, for the death of a child to influence changes in family structure. Our data do not distinguish whether, within a specific year, a child's death or a parent's death occurs first. Of 462 child deaths observed to occur after the first year of life, we observe only 18 instances to co-occur with a change in marital state (4%). Thus, we do not view the possibility of "reverse causality" will substantially affect the results we observe. However, as an additional robustness check, and to assess the potential for parental states to be affected by child outcomes, we analyzed the association between parental state in the year $t + 1$ and child outcomes in the year t.

Comparison of results. The results of survival analyses of the "t+1" robustness check are presented in Figure S36. Full fitted model summaries and traceplots are shown in Tables S25-S26 and Figures S37-S38. No associations between parent state in $t+1$ and child survival at time t are observed. The association between "either parent external" and child outcomes are identical, as in the results presented in the main text—as expected—because this parental state is not time-varying.

The results of height analyses of the "t+1" robustness check are presented in Figure S39. Full fitted model summaries and traceplots are shown in Tables S27-S28 and Figures S40-S41. No associations between parent state in $t+1$ and child height at time t are observed.

The results of weight analyses of the "t+1" robustness check are presented in Figure S42. Full fitted model summaries and traceplots are shown in Tables S29-S30 and Figures S43-S44. No associations between parent state in $t+1$ and child weight at time t are observed.

The results of education analyses of the "t+1" robustness check are presented in Figure S45. Full fitted model summaries and traceplots are shown in Tables S31-S34 and Figures S46-S47. No associations between parent state in $t+1$ and child education at time t are observed.

Excluding children of unknown sex. We have records in our survival database of 278 children whose sex was not reported. For most cases, this was due to parents not remembering the sex of an infant who died a long time ago. In some cases, a parent did not remember or know the sex of a child who lived at the time in a different village with a missing parent. Figure S48 describes more details about the potential reason for missing information on the sex of each of these children.

To consider whether our results are robust to the decision to include these children in our database, we assess survival outcomes using a dataset which excludes information on the 278 children of unknown sex.

Comparison of results. The results of survival analyses for the sample restricted to children of known sex are presented in Figure S49. Full fitted model summaries and traceplots are shown in Tables S35-S36 and Figures S50-S51.

The results of this analysis are similar to those reported in the main text. The loss of a mother is associated with reduced survival in the first few years of life. In the main text, we reported a decline in survival probability for a female infant with a deceased mother of about 0.14 (90% HPDI: -0.01, 0.30) compared with a female infant with two living parents. The corresponding prediction for the dataset excluding children of unknown sex is a decline in survival probability of 0.1 (90% HPDI: -0.02, 0.24). This analysis reproduces the result that having a parent of unknown status is associated with reduced probability of survival. In the main text, we reported a predicted reduction in survival of 0.05 at age 19 (90% HPDI: 0.02, 0.08) for a child with a parent of unknown status. Excluding children of unknown sex, we predict a corresponding reduction in survival at age 19 of 0.04 (90% HPDI: 0.00, 0.07). There is no association observed between child survival and any other maternal state, or any paternal state. The patterns in age-specific, male-, twin-, birth-order and year-specific effects are also reproduced here.

Excluding early data (individuals born pre-1976). We draw on data from full reproductive interviews conducted during the period of fieldwork between 1995 and 2014. During these reproductive interviews, individuals also reported births and deaths of children who lived before the period of fieldwork. Consequentially, these children were not observed by MBM during the period of fieldwork. To ensure that the inclusion of these data do not substantially bias our results, we assess survival outcomes using a dataset which excludes information on children born before the year 1976. This means we include all data that corresponds to all individuals who would have been alive and under the age of 18 during the period of fieldwork (which began in 1995).

Comparison of results. We analyze a dataset of survival outcomes for 2,965 children (1,387 boys, 1,397 girls, 181 of unknown sex) born after the year 1975. The results of these analyses are shown in Figure S52. Full fitted model summaries and traceplots are shown in Tables S37-S38, and Figures S52-S54.

The associations between parental states and survival outcomes observed are similar to those reported in the main text. The death of a mother is associated with substantially reduced probability of survival in the first few years of life (Figure S52, Panel C). For the full sample of 3,693 children, we observe a decline in survival probability for a female infant with a deceased mother of about 0.14 (90% HPDI: -0.01, 0.30) compared with a female infant with two living parents. The corresponding prediction for the dataset of 2,965 children born after 1976 is a decline in survival probability of 0.20 (90% HPDI: 0.00, 0.40). In addition, a child with a parent of unknown status is predicted to experience reduced survival probability compared with a child of two known, living parents. This decline for the full sample of 3,693 children is predicted to be about 0.05 at age 19 (90% HPDI: 0.02, 0.08). We observe the same pattern in the set of children born post-1976, corresponding to a predicted survival reduction to age 19 of about 0.07 (0.03, 0.11). No associations are observed between father death or parent marriage status and child survival outcomes. Similar patterns as for the full sample are observed in age-specific twin- and male- effects. In summary, the observed trends in the full sample are reproduced in the sample of 2,965 children.

The predicted estimates for maternal loss or a parent of unknown status observed for the restricted sample seem to be larger than in the full sample, suggesting that we may be under-estimating the associations between parent states and child survival when we include data for children born time a long time ago. Indeed, some indication that data for children born pre-1976 is more prone to reporting error is observed in the pattern of estimated age-specific effects. For the full sample of 3,693 children, we observe a clear decline in the probability of surviving age 10 (Figure S5). Excluding children born before 1976, we do not observe a similar decline in probability of survival at age 10. The reduction in survival probability at age 10 for the full sample likely reflects a tendency to round the age of a child who died in childhood, a long time ago, to a rough value of 10.

5. Power assessment

Our analysis aims to assess whether there is any association between family structure and child outcomes at different ages during childhood. We found no substantial associations between various parental marital states and multiple child outcomes. We interpret this finding as evidence supporting the hypothesis that, in particular contexts, family structures and marital events that deviate from monogamy might have negligible impacts on child well-being. Such a strong claim of an “absence of an effect” can only be made if our data are sufficiently powered to detect potential positive or negative effects, should they exist. Therefore, we conducted power assessment to determine the range of *effect sizes* that can be reliably detected given our overall sample size—and, more specifically, the effective sample sizes for each of the particular parental marital state variables.

Procedure. We simulated hypothetical outcome data (survival events, height measurements, weight measurements, and years of schooling) for a range of plausible parameter values, for each set of age-specific parameters we studied (Table S39). The effective sample sizes of each predictor variable are fixed by using our empirical set of covariates to simulate hypothetical outcome data. Across simulations, we vary the “true” causal effect of each predictor variable. We then fit our statistical models to the simulated outcomes, and test if the dataset is sufficiently powered to detect true effects of varying magnitudes. We then plot the generative “true” parameter values alongside the 90% posterior intervals of the parameter values estimated by the statistical models. An overlap between the estimated interval and the simulated value indicates that we are able to detect an effect.

Results summary. For most substantive effect sizes, for most variables, we find that our models permit excellent parameter recovery. The only truly under-powered predictor variable is “father married to stepmother (polygyny)”; we have very few cases of child-years in which a child was exposed to parents in this marital state. As such, we cannot detect small effects of this variable reliably. This is not a concern for our inferences in the main text, however, because the results presented in the main text reflect this lack of power—i.e., the posterior credible intervals are wide, and do not exclude the existence of small to moderate effects sizes, nor do we interpret wide credible regions around zero as evidence for the absence of an effect. Such inferences are only possible when the credible region is tight enough around zero to exclude effects of substantive size.

Survival results. Our survival power assessment confirms that we can recover simulated effects for a range of parameter values for each of the covariates we are interested in. Figures S55-S63 show the results of these analyses. Each figure corresponds to a specific covariate set (listed, for reference, in Table S39). In general, we are able to recover most effects well, however, there are some exceptions arising for well-understood reasons. Our analysis indicates that we are unable to precisely detect very large positive effects (+2 on the log-odds scale) for some covariates (twin, mother deceased, father deceased, father unmarried). This is because the overall rate of survival is rather high, and large-positive effects on the log-odds scale are unlikely to change the probabilities of survival on the natural scale by much. A large positive effect of being twin, having a deceased parent, or unmarried parent, at any age is also biologically and sociologically unlikely and therefore unprecedented. Thus, our inability to detect such effects should not detract in any meaningful way from our analysis. We also have limited power to detect effects for the “father married to stepmother (polygyny)” covariate (Figure S61); as stated above, we observe few instances of this family structure overall. Still, our power analysis indicates that we are able to detect a strong negative effect (Figure S61, first panel), and thus our conclusion that no substantial costs of polygyny or step-parent presence—also indicated by other covariates—remains robust.

Height results. Our height power analysis confirms that we can recover simulated effects for a range of parameter values for each of the covariates we are interested in. Figures S64-S72 show the results of these analyses. For some covariates (“mother deceased”, “father married to stepmother (polygyny)”), we are unable to detect very small positive or negative effects (-0.2 to 0.2) with precision. For the same covariates, it appears that we may under-estimate some effects at young ages.

Weight results. Our weight power analysis confirms that we can recover simulated effects for a range of parameter values for each of the covariates we are interested in. Figures S73-S81 show the results of these analyses. As for height data, we observe limited power to detect small positive or negative effects of specific covariates—“mother deceased” and “father married to stepmother (polygyny)”. Similarly, we observe lower precision to detect effects for these covariates and may therefore under-estimate these effects for very young children (Figures S75 and S79).

Education results. Our education power analysis confirms that we can recover simulated effects for large parameter values for most of the covariates we are interested in. Figures S73-S81 show the results of these analyses. We have limited power to detect small effect sizes for several covariates. This means that we are only able to exclude the possibility that large associations (larger than 0.4) between covariates and years of schooling exist. Small impacts of parental states on child education would be undetected by us.

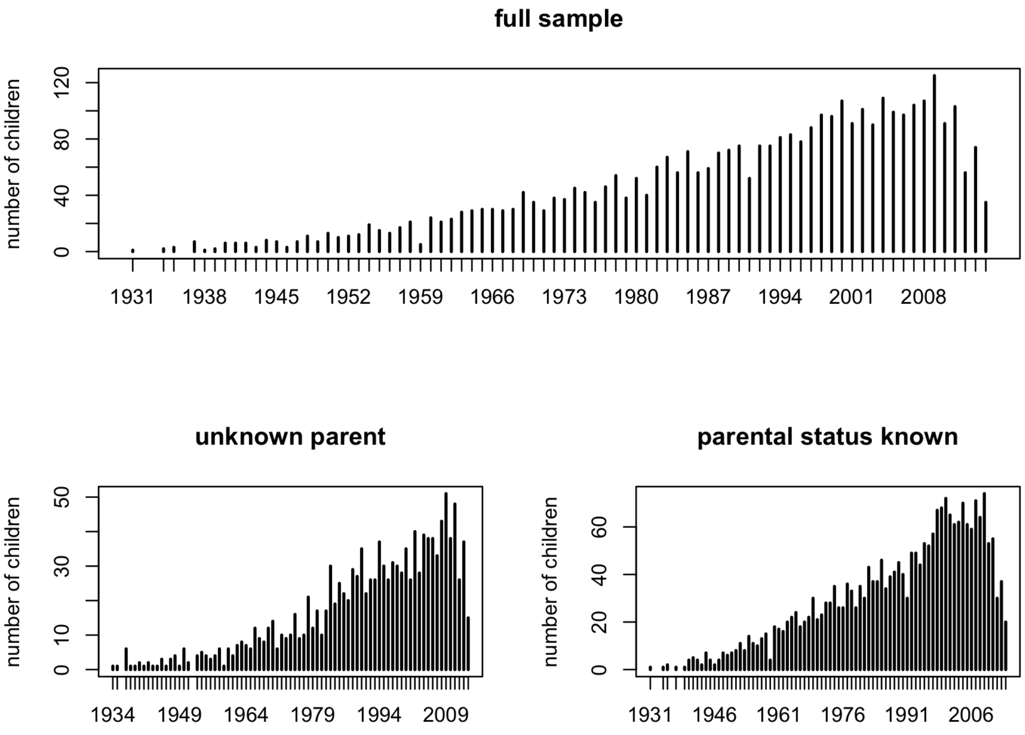

Fig. S1. Frequency distribution of birth-years in the survival database. The top panel shows all children in the sample, while the panels below separate children born to an "external" parent (n = 1,302) from children born to two parents of known vital/marital status (n = 2,391).

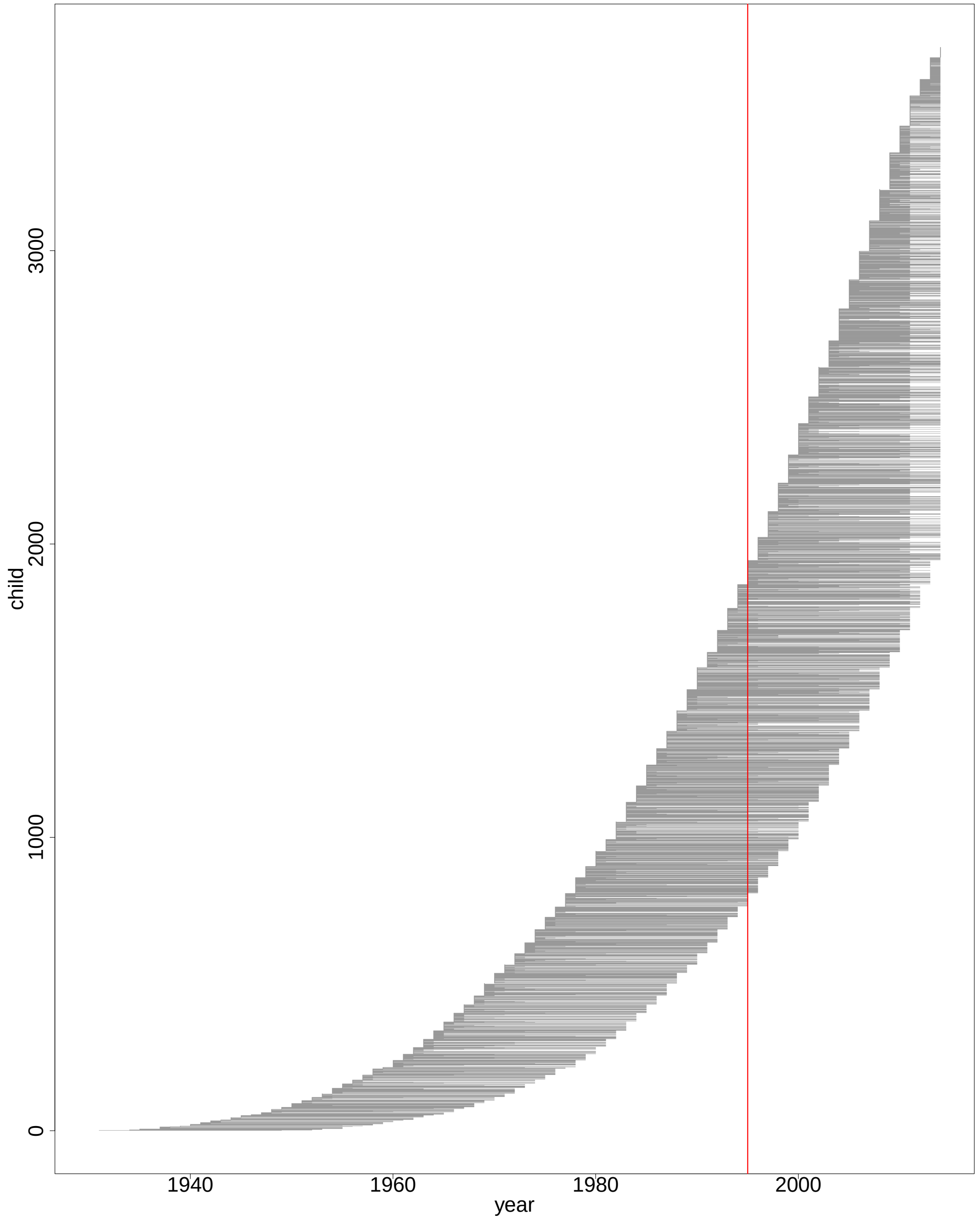

Fig. S2. Birth–death data of children in the survival database. Each grey line represents a child. The line shows a child's year of birth until year of death or censoring from the database. The red dotted line shows the beginning of the fieldwork period (1995). Some children lived entirely before the period of fieldwork, and the periods of their childhoods were constructed from reproductive interviews with their parents.

Table S1. Data on predictor variables. Number of child-years recorded in each mother-state category.

child age	deceased	unmarried	married to step-father	married to father (monogamous)	married to father (with cowife)
0–5	113	2891	2763	7352	698
6–10	302	1917	2377	4886	511
11–15	419	1551	1895	3453	337
16–18	410	1002	1224	2064	205

Table S2. Data on predictor variables. Number of child-years recorded in each father-state category.

child age	deceased	unmarried	married to step-mother (monogamous)	married to step-mothers (polygynous)	married to mother (monogamous)	married to mother (polygynous)
0–5	126	1352	1213	124	7432	688
6–10	384	961	1108	156	4957	509
11–15	639	751	882	171	3503	346
16–18	549	472	547	99	2096	223

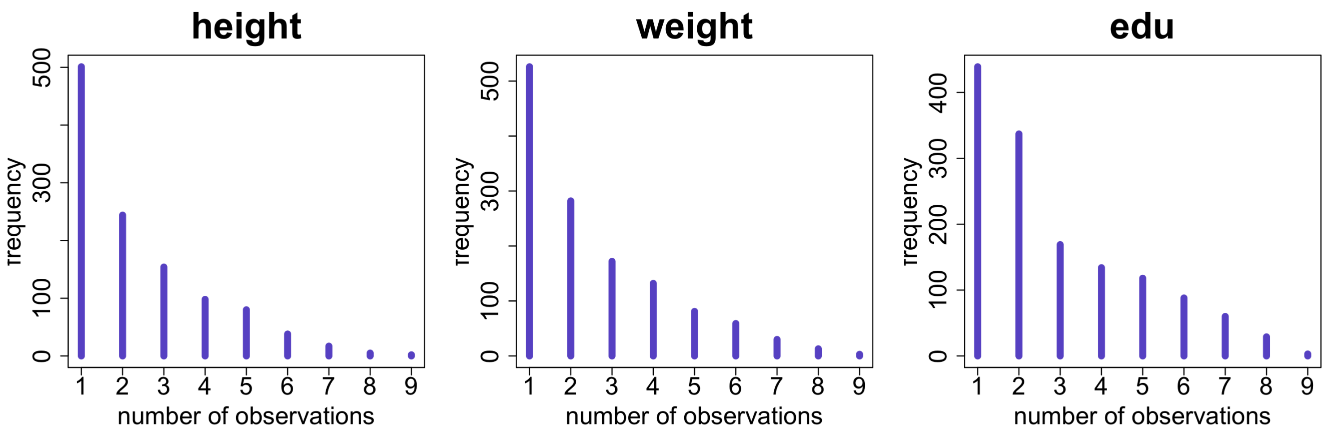

Fig. S3. Frequency of height, weight, and education observations per child.

Table S3. Number of observations, children, and parents tabulated by outcome.

N	survival	height/weight	education
children	3,693	881	1,370
observations	-	1,744	3,693
female | male | sex unknown	1,709 | 1,706 | 278	469 | 412 | 0	703 | 667 | 0
mothers | fathers	662 | 461	324 | 245	423 | 314
"external" mothers | "external" fathers	272 | 1030	13 | 140	53 | 279

Table S4. Number of observations/measurements recorded, tabulated by outcome and child age category.

age	outcome		
	survival	height/weight	education
0–1	3,693	0	0
1–2	3,253	15	0
2–3	2,911	63	0
3–4	2,746	157	0
4–5	2,587	173	265
5–6	2,468	182	335
6–7	2,355	186	346
7–8	2,244	163	310
8–9	2,139	166	309
9–10	2,052	132	284
10–11	1,926	114	299
11–12	1,822	100	288
12–13	1,736	79	264
13–14	1,659	49	249
14–15	1,580	39	221
15–16	1,495	32	203
16–17	1,428	31	145
17–18	1,351	29	101
18–19	1,288	34	74

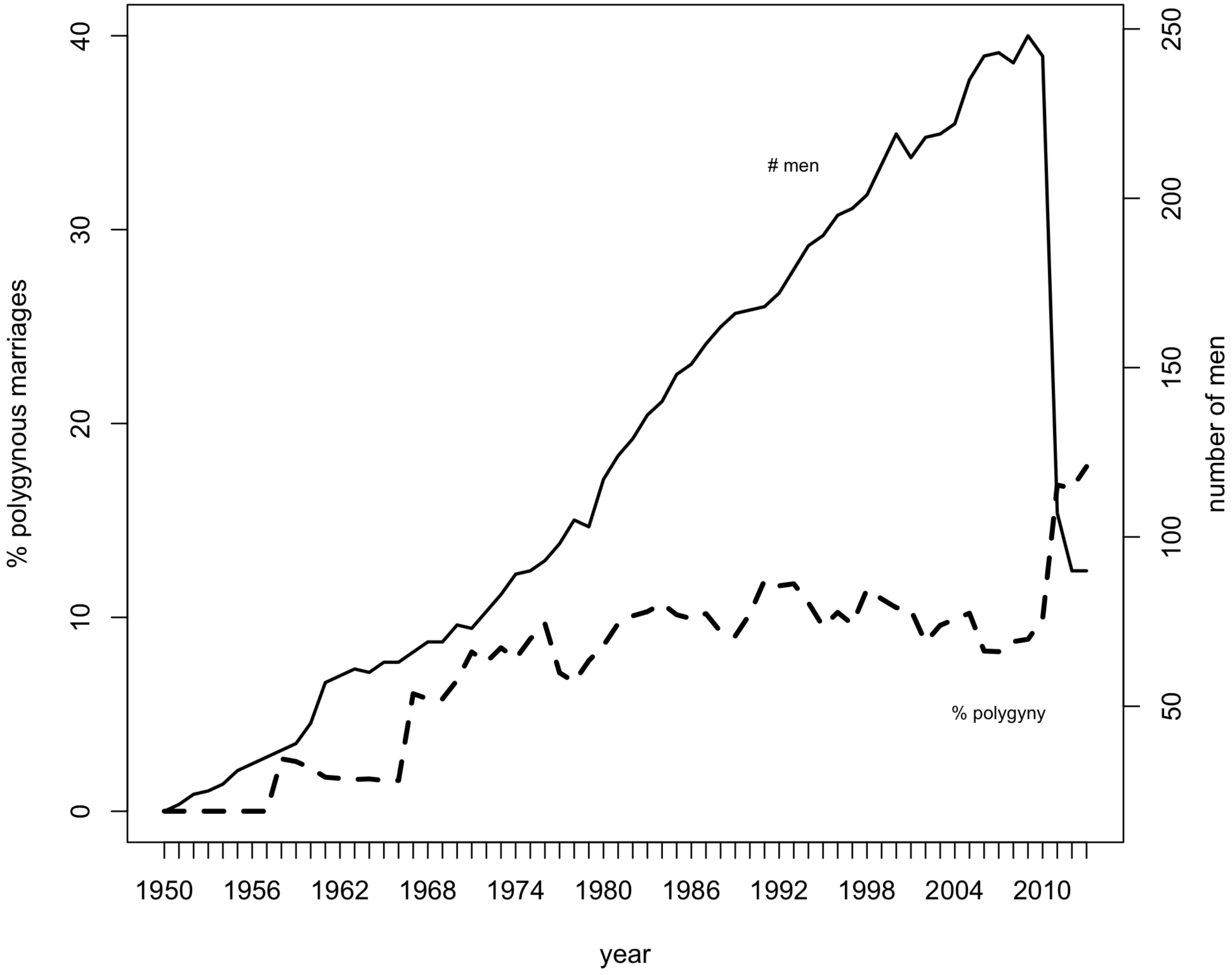

Fig. S4. Left axis, dotted line: percentage of marriages that are polygynous. Right axis, solid line: number of men sampled. Note: the precipitous drop in sampling intensity at the last year of observation, reflects that M.B.M. was ill and could not complete surveys with all respondents that year.

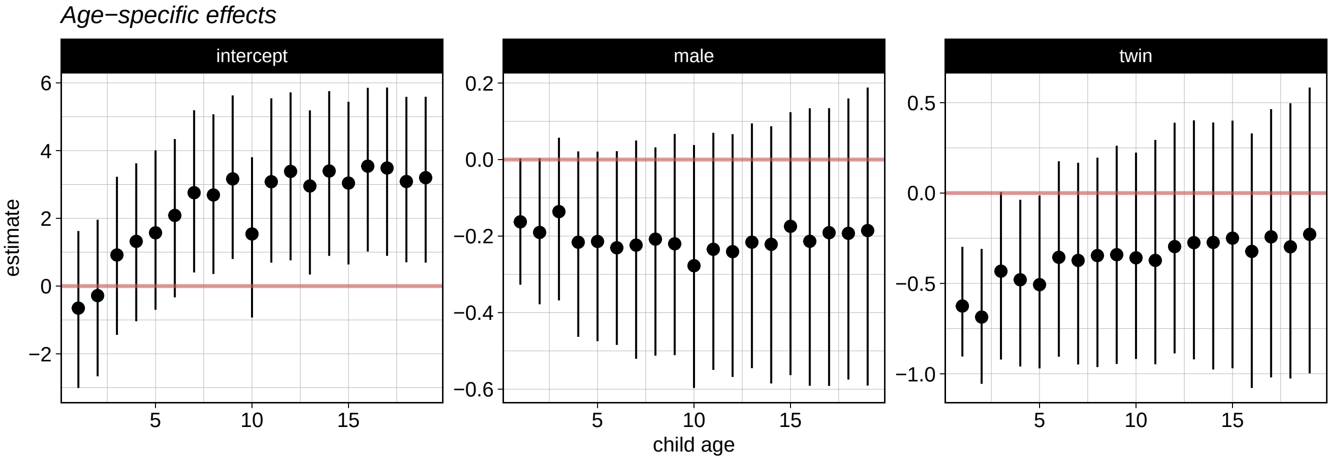

Fig. S5. Age-specific effects on survival. The panel on the left shows the age-specific intercepts. The panel in the middle shows the effect of being male on survival. The panel on the right shows the effect of being a twin on survival.

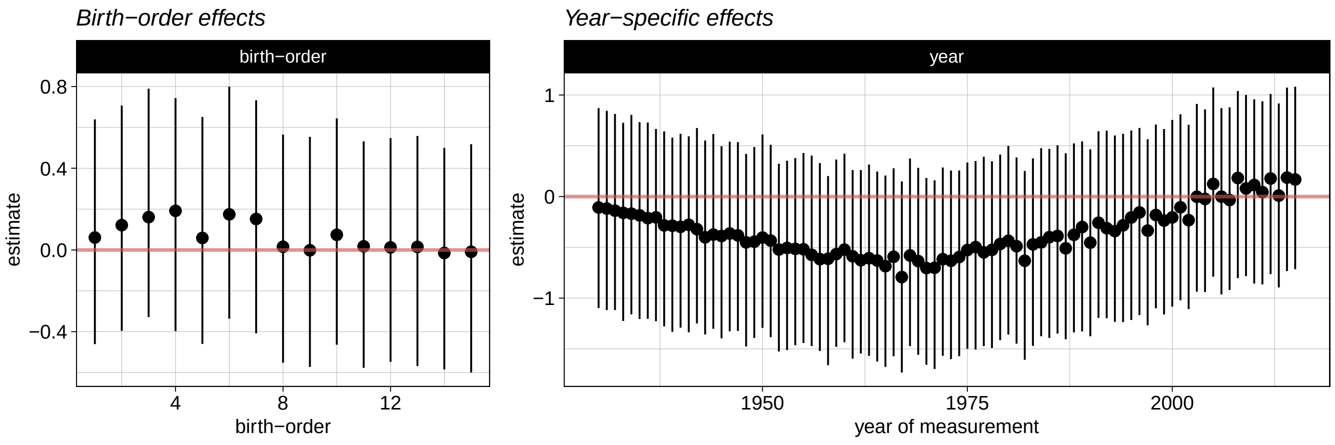

Fig. S6. Birth-order and year-specific effects on survival.

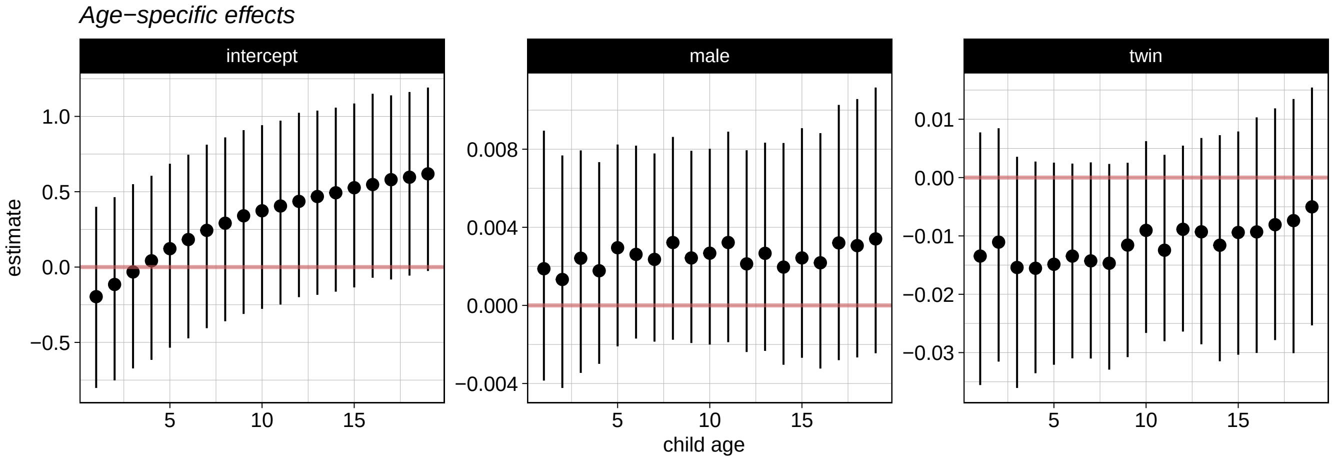

Fig. S7. Age-specific effects on child height. The panel on the left shows the age-specific intercepts. The panel in the middle shows the effect of being male on height. The panel on the right shows the effect of being a twin on height.

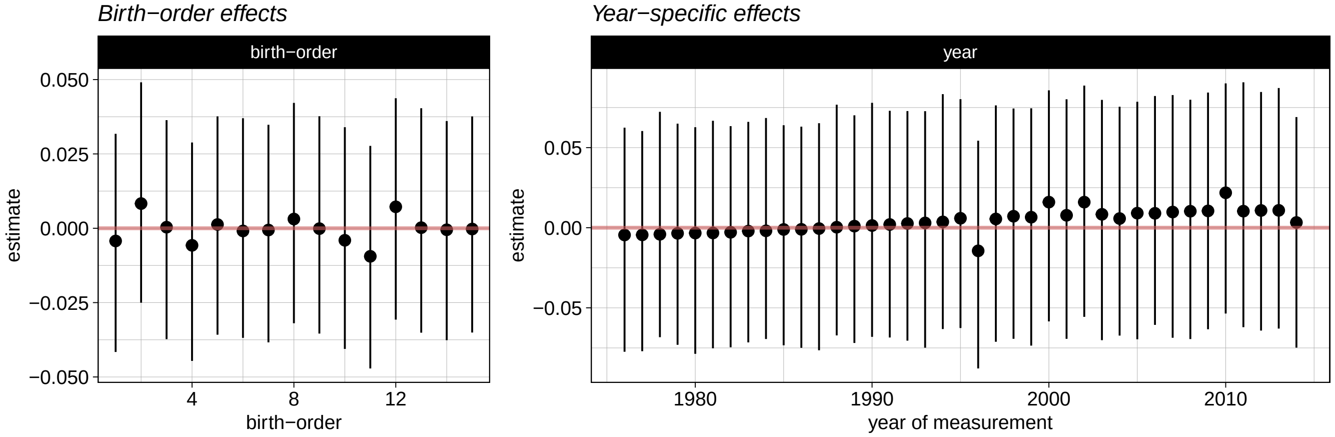

Fig. S8. Birth-order and year-specific effects on height.

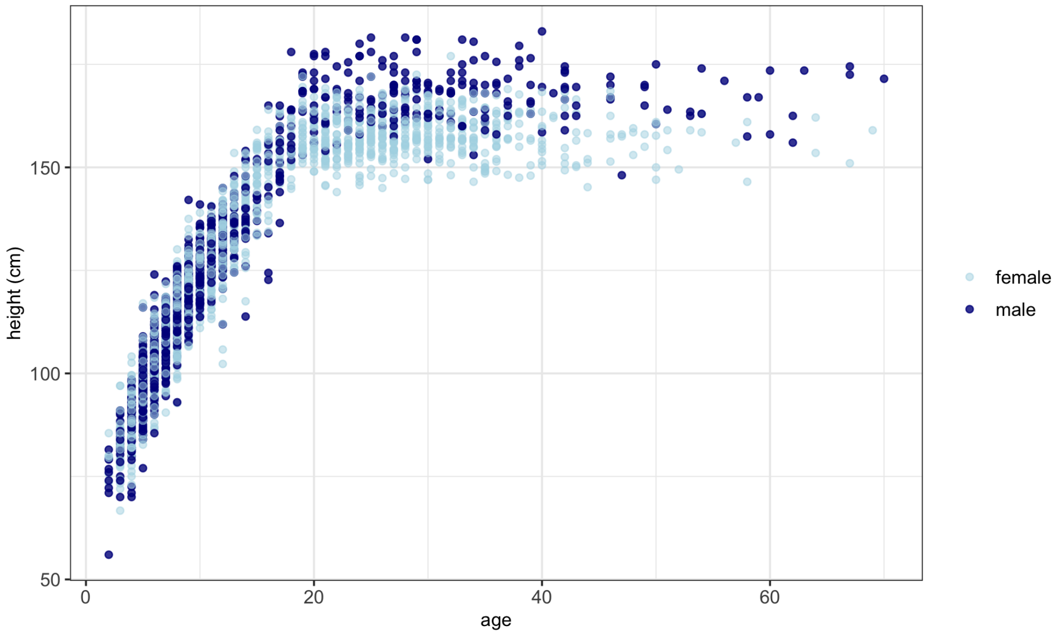

Fig. S9. Height-for-age observed for individuals in our sample from ages 0-70. Observations for female individuals are shown in light blue, and observations for male individuals in dark blue.

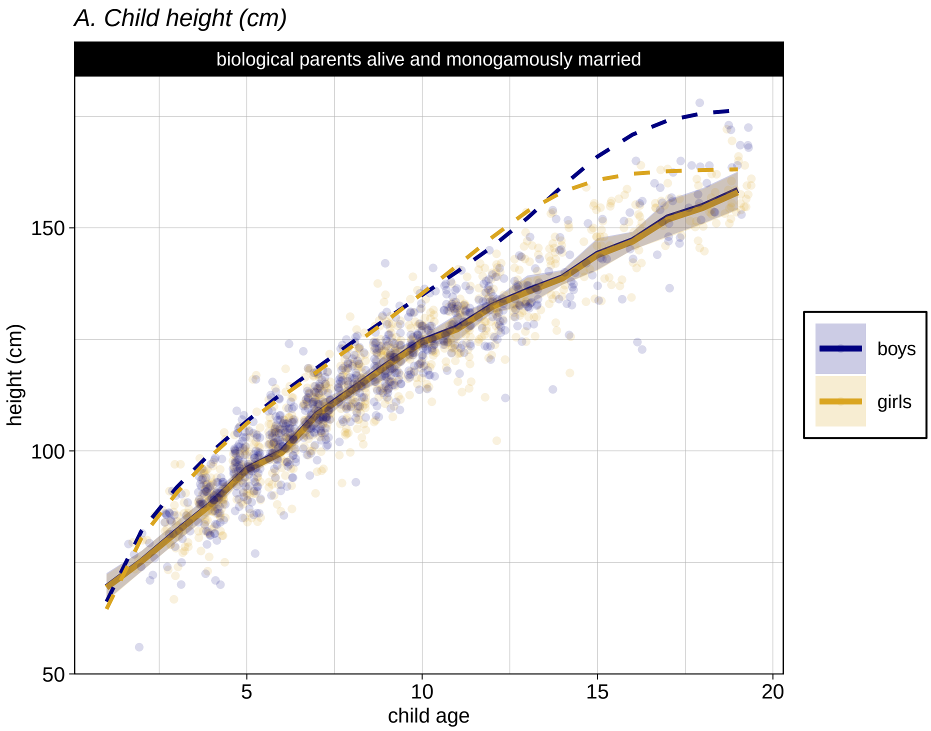

Fig. S10. Visual comparison of sex-specific height trajectories of Pimbwe children with the global reference population standards from the WHO. WHO growth curves are plotted in dotted lines, while Pimbwe measurements are shown by the dots (observed data) and solid lines (statistical model predictions).

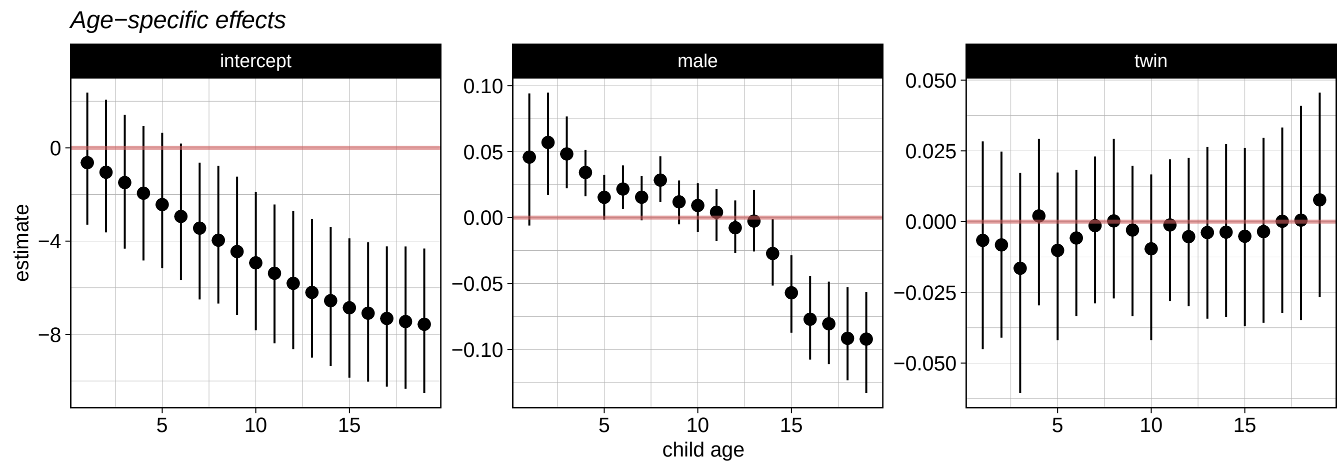

Fig. S11. Age-specific effects on weight-for-height. The panel on the left shows the age-specific intercepts. The panel in the middle shows the effect of being male on weight-for-height. The panel on the right shows the effect of being a twin on weight-for-height.

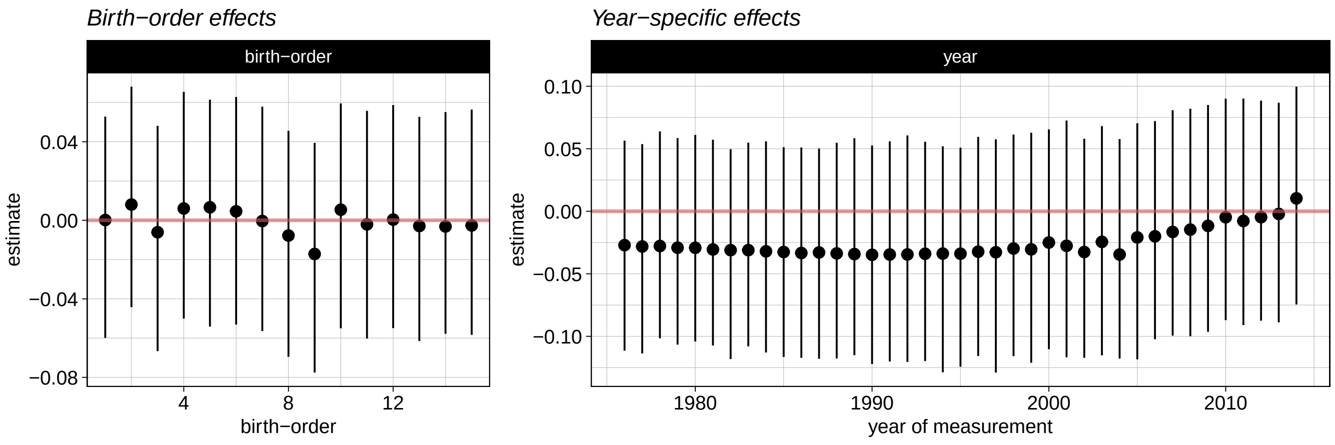

Fig. S12. Birth-order and year-specific effects on weight-for-height.

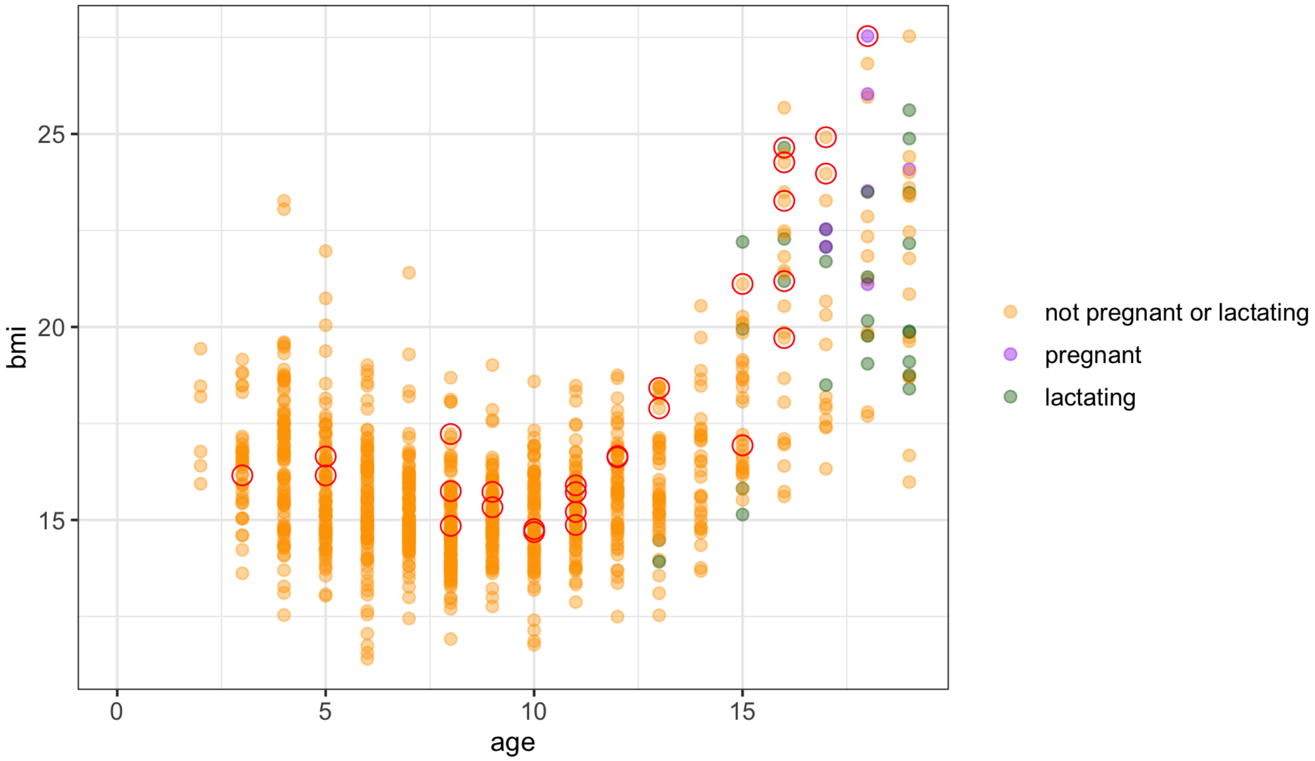

Fig. S13. Observed weight-for-height (female individuals only). Weight observations made in the same year as a pregnancy/time of lactation are shown in purple (pregnancy) and green (lactation). Points circled in red are observations of girls with a deceased mother.

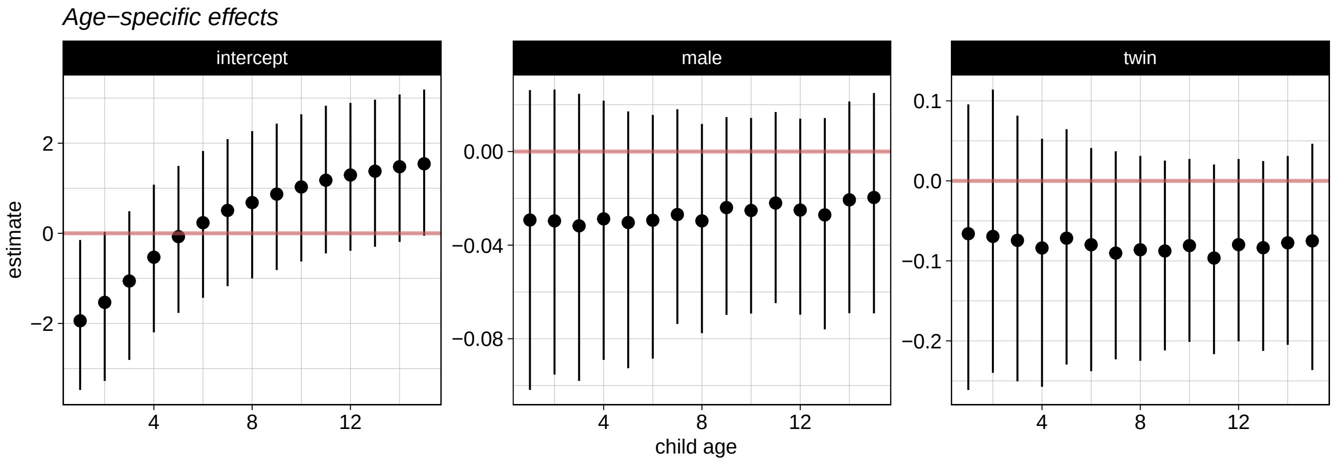

Fig. S14. Age-specific effects on years of schooling reported. The panel on the left shows the age-specific intercepts. The panel in the middle shows the effect of being male on years of schooling. The panel on the right shows the effect of being a twin on years of schooling.

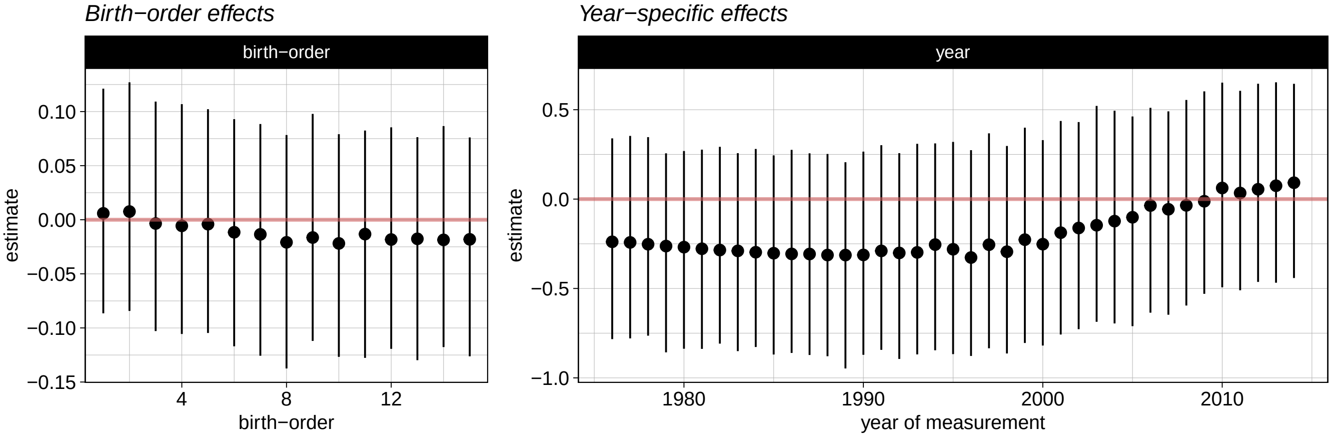

Fig. S15. Birth-order and year-specific effects on years of schooling.

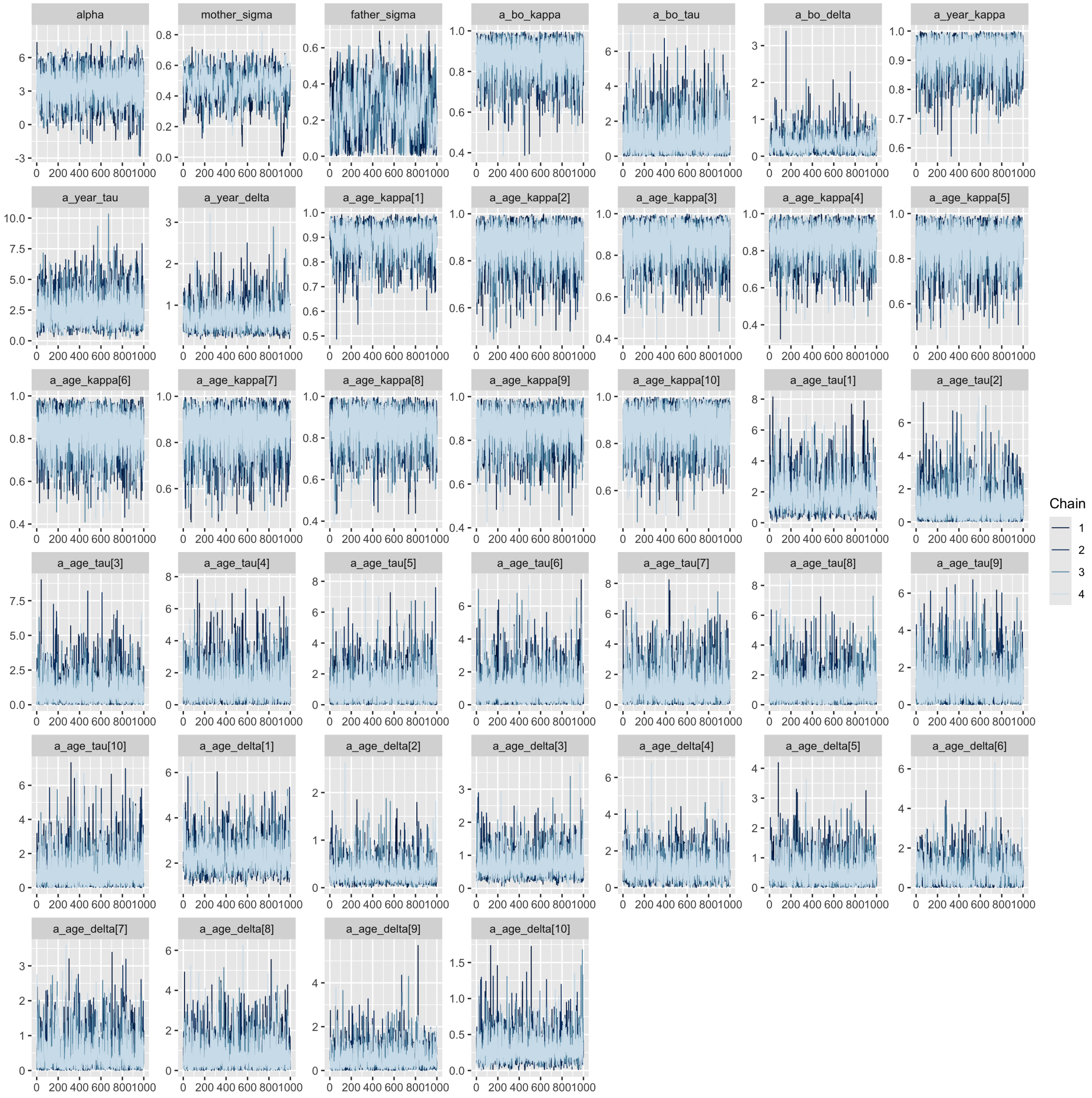

Fig. S16. Traceplots showing good mixing and convergence of four chains to the same posterior region for main model parameters—for the model of father status and child survival.

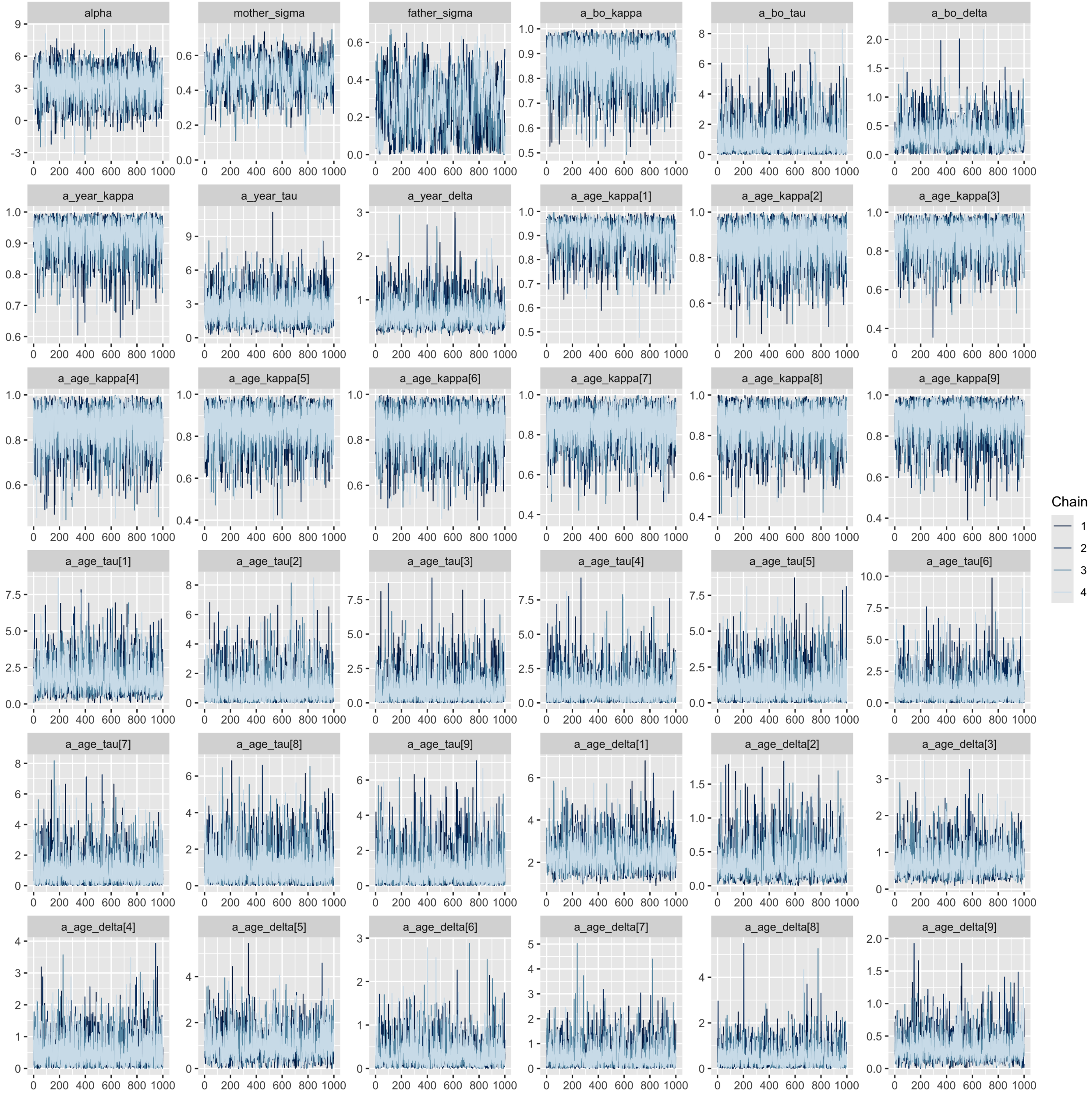

Fig. S17. Traceplots showing good mixing and convergence of four chains to the same posterior region for main model parameters—for the model of mother status and child survival.

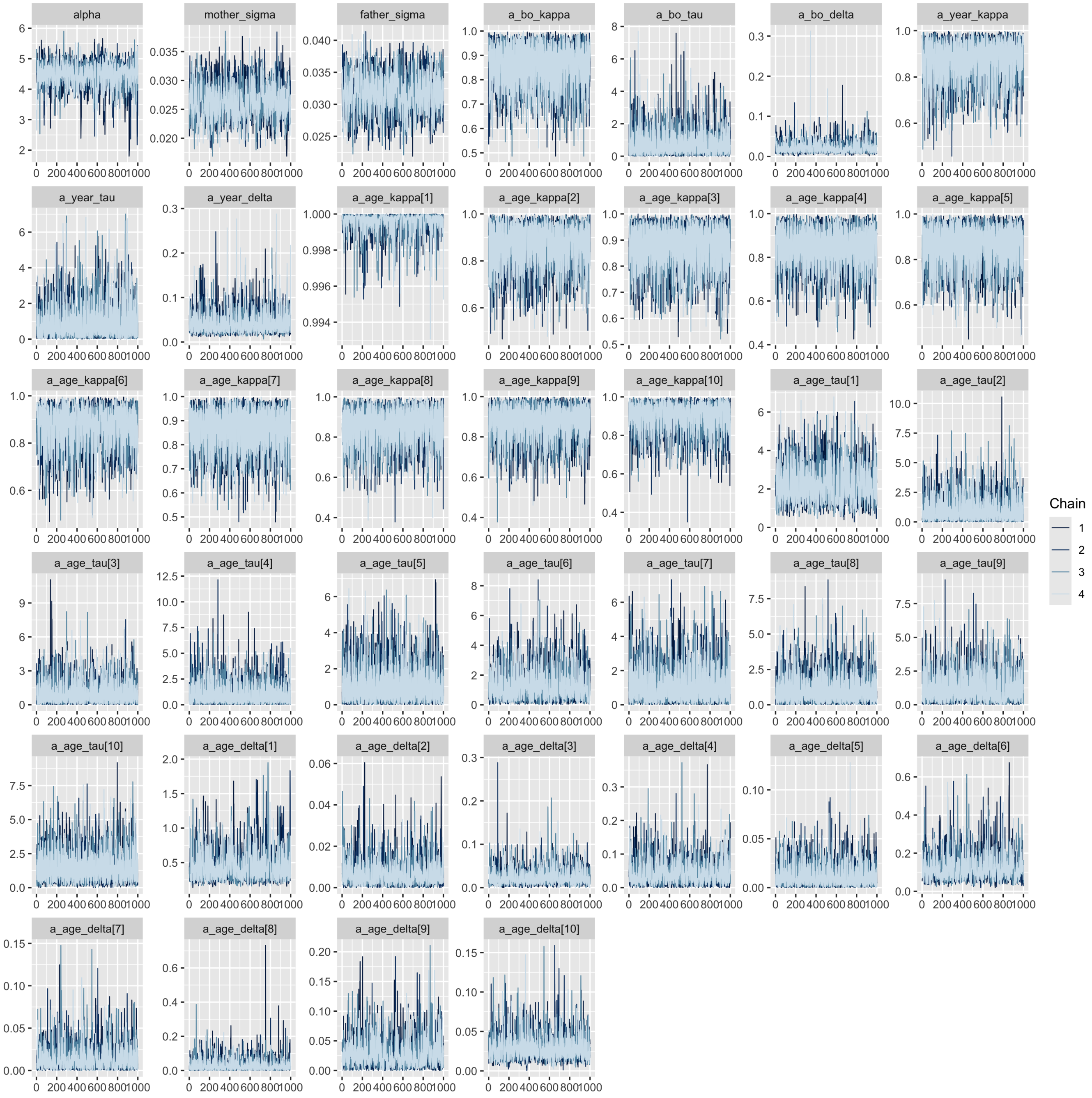

Fig. S18. Traceplots showing good mixing and convergence of four chains to the same posterior region for main model parameters—for the model of father status and child height.

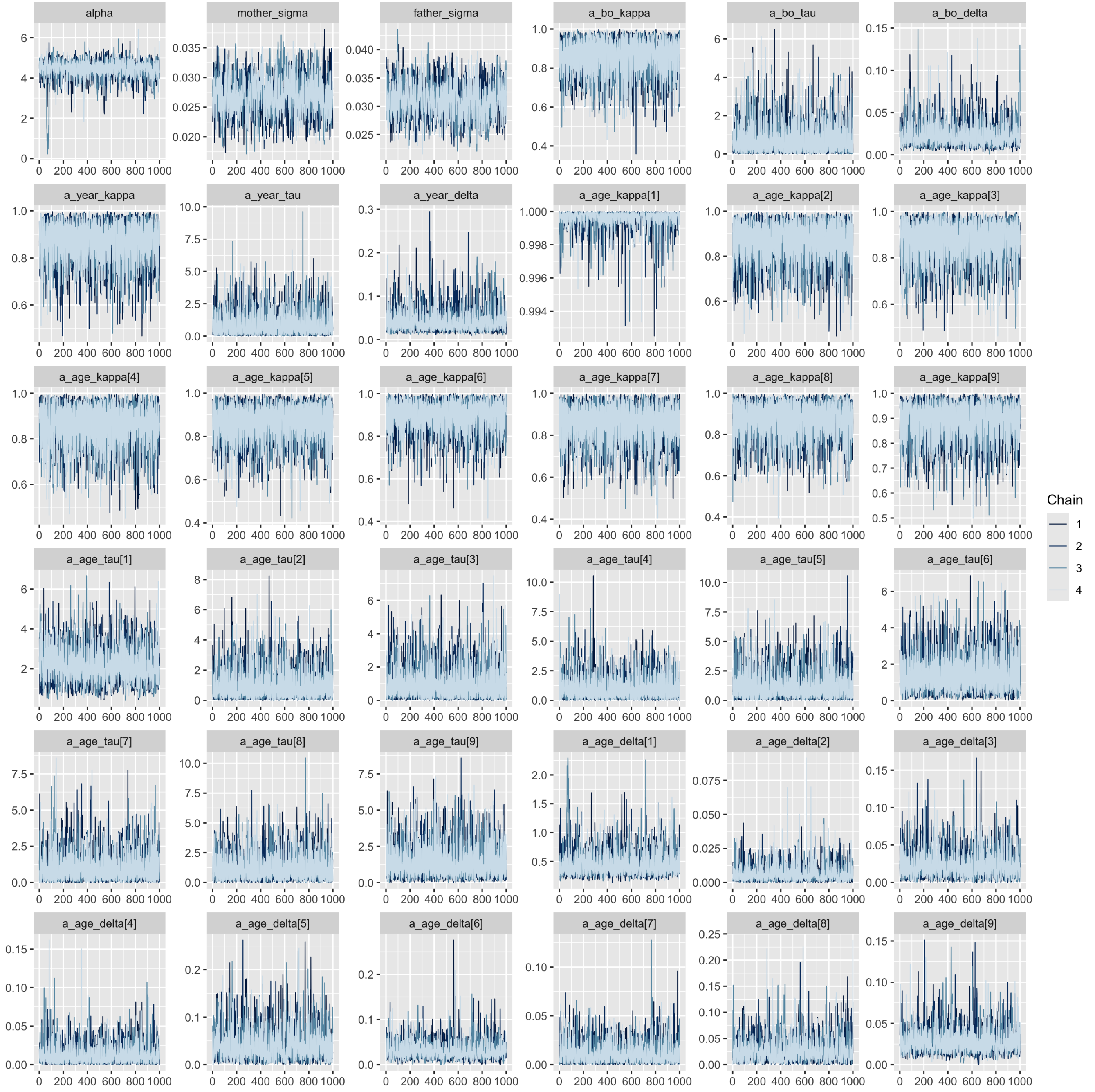

Fig. S19. Traceplots showing good mixing and convergence of four chains to the same posterior region for main model parameters—for the model of mother status and child height.

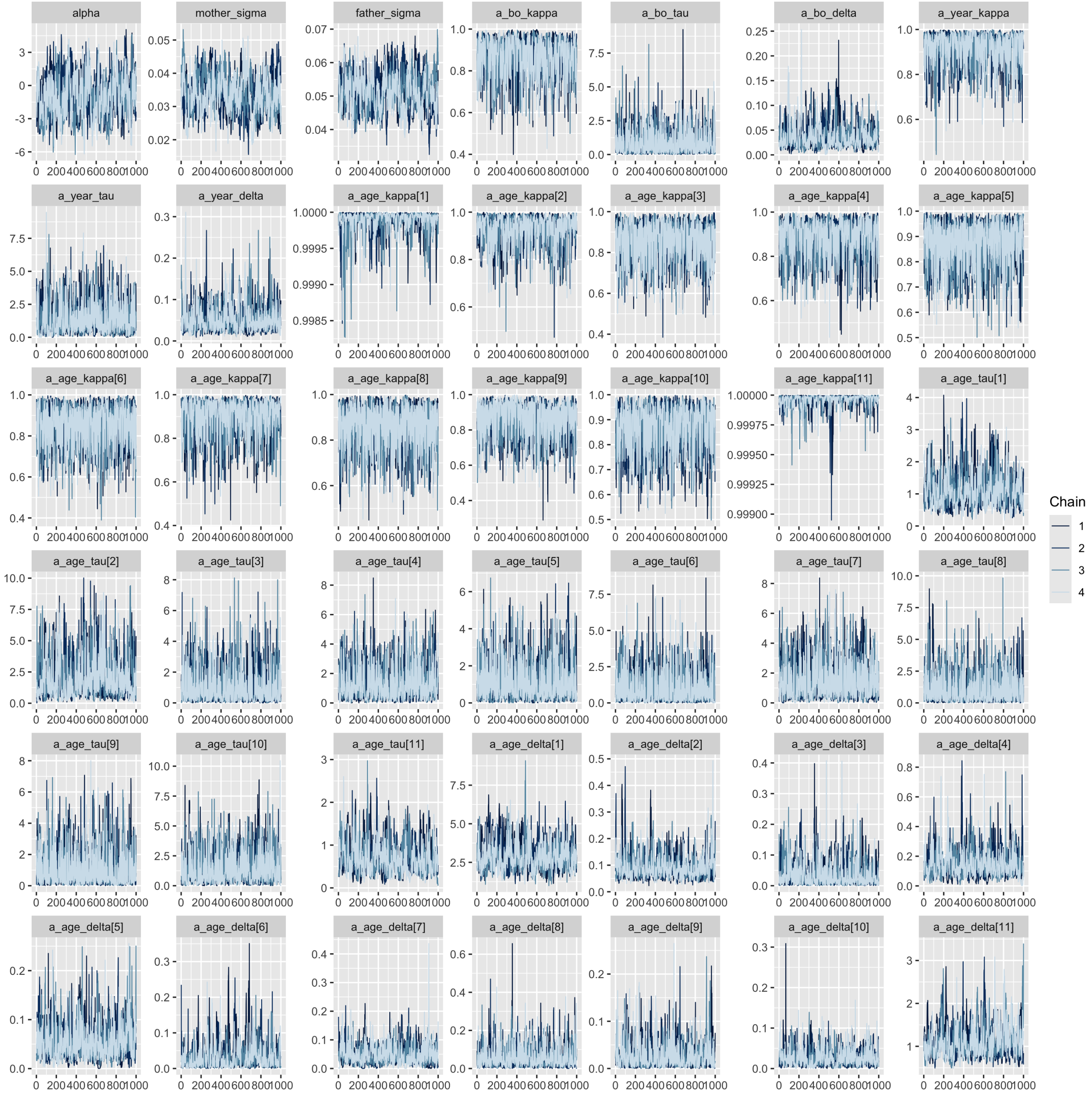

Fig. S20. Traceplots showing good mixing and convergence of four chains to the same posterior region for main model parameters—for the model of father status and child weight.

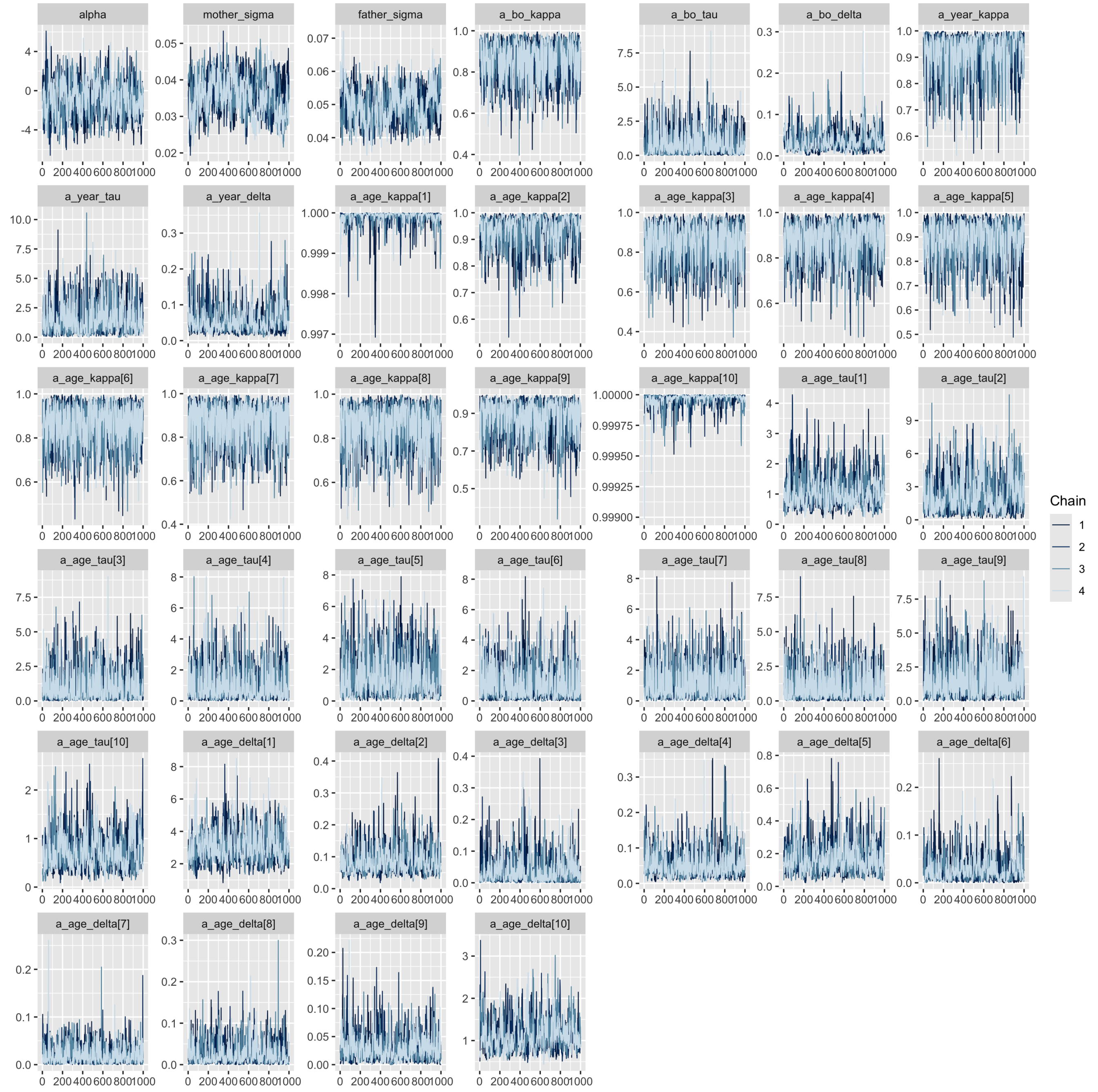

Fig. S21. Traceplots showing good mixing and convergence of four chains to the same posterior region for main model parameters—for the model of mother status and child weight.

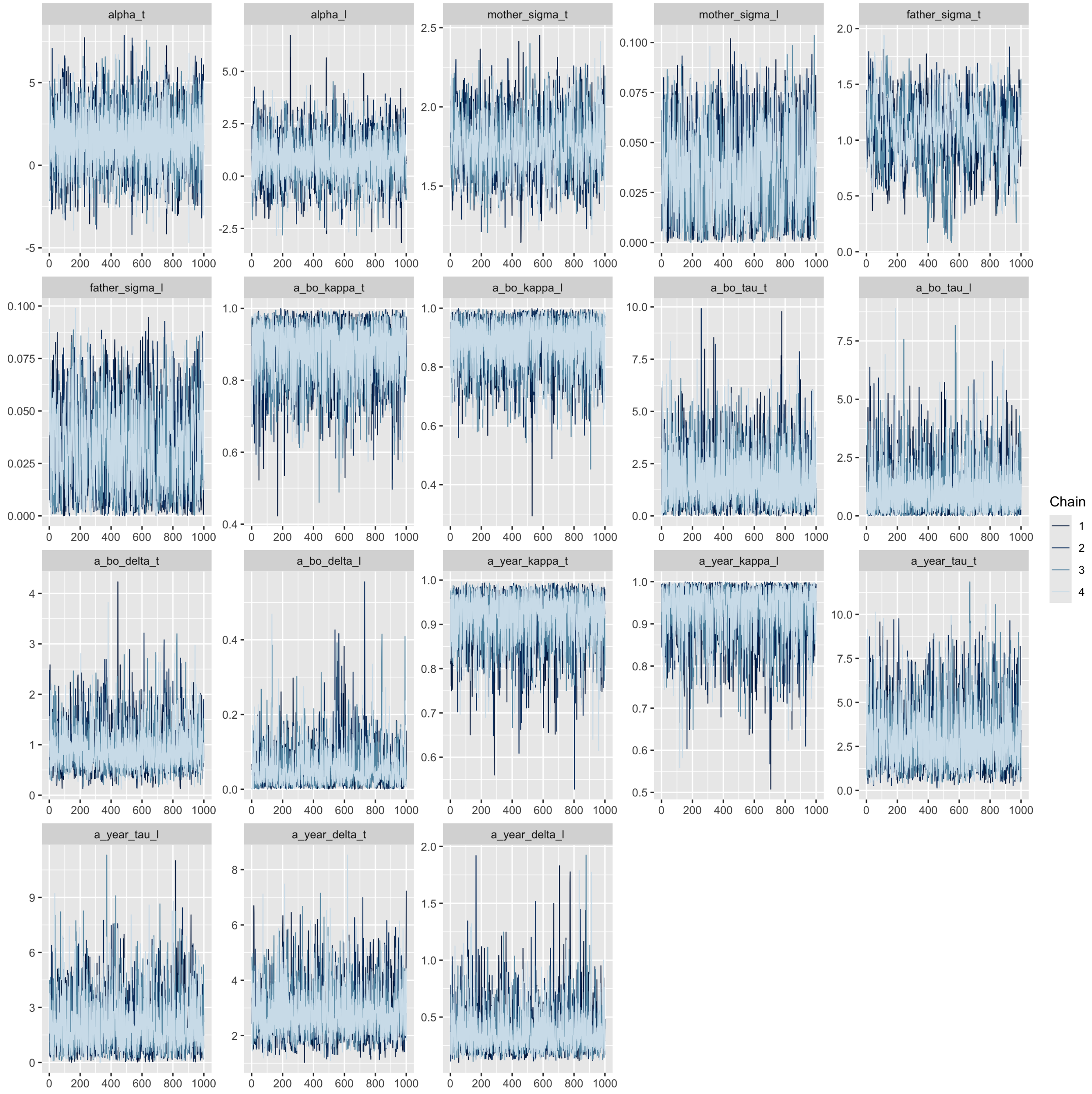

Fig. S22. Traceplots showing good mixing and convergence of four chains to the same posterior region for main model parameters—for the model of father status and child education.

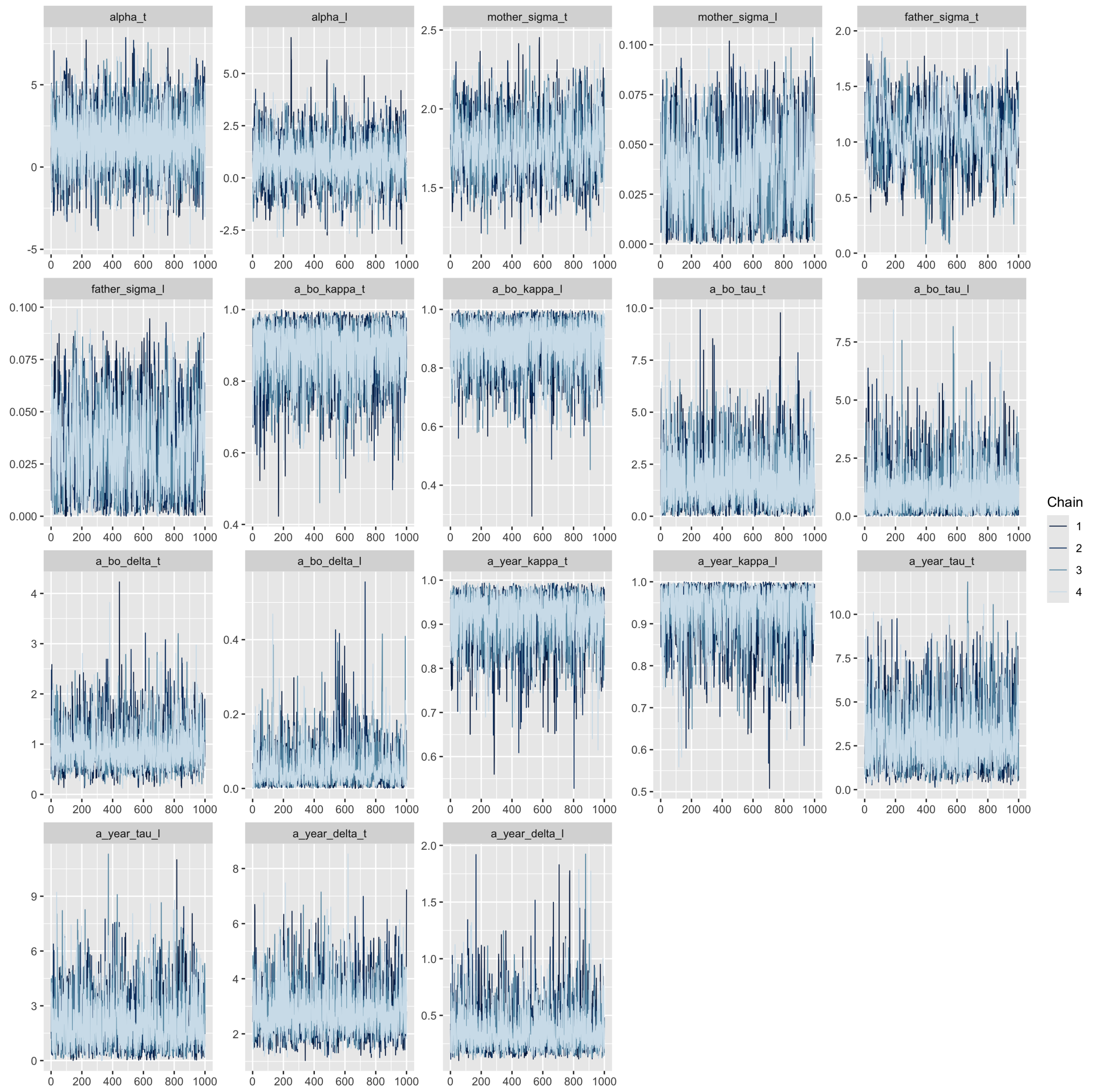

Fig. S23. Traceplots showing good mixing and convergence of four chains to the same posterior region for main model parameters—for the model of mother status and child education.

	mean	sd	5.5%	94.5%	rhat	ess_bulk
α	3.35	1.45	1.00	5.58	1.00	2587.94
γ_τ	0.95	0.96	0.06	2.83	1.00	4619.17
γ_κ	0.87	0.09	0.71	0.97	1.00	5174.63
γ_δ	0.32	0.24	0.04	0.73	1.00	1133.60
ϵ_τ	2.45	1.27	0.87	4.87	1.00	3046.89
ϵ_κ	0.92	0.06	0.81	0.99	1.00	2727.83
ϵ_δ	0.68	0.30	0.34	1.21	1.00	2600.28
β_{τ_1}	1.69	1.07	0.50	3.73	1.00	1751.24
β_{τ_2}	0.94	0.93	0.05	2.75	1.00	5360.50
β_{τ_3}	0.95	0.99	0.05	2.81	1.00	4977.58
β_{τ_4}	1.13	1.07	0.07	3.16	1.00	4750.30
β_{τ_5}	0.96	0.97	0.05	2.78	1.00	4544.83
β_{τ_6}	0.90	0.94	0.05	2.69	1.00	4548.63
β_{τ_7}	1.10	1.07	0.06	3.16	1.00	5405.95
β_{τ_8}	0.98	0.99	0.05	2.84	1.00	5104.19
β_{τ_9}	0.97	0.97	0.05	2.85	1.00	5408.75
$\beta_{\tau_{10}}$	0.88	0.92	0.04	2.60	1.00	4685.45
β_{κ_1}	2.27	0.67	1.42	3.49	1.00	1972.39
β_{κ_2}	0.32	0.23	0.08	0.75	1.00	2047.96
β_{κ_3}	0.71	0.38	0.28	1.40	1.00	2315.07
β_{κ_4}	1.03	0.68	0.17	2.26	1.00	1700.57
β_{κ_5}	0.48	0.45	0.03	1.30	1.00	2664.81
β_{κ_6}	0.71	0.61	0.04	1.80	1.00	1034.16
β_{κ_7}	0.47	0.46	0.03	1.36	1.00	1912.58
β_{κ_8}	0.72	0.74	0.04	2.07	1.00	3542.41
β_{κ_9}	0.51	0.49	0.04	1.40	1.00	1765.13
$\beta_{\kappa_{10}}$	0.29	0.19	0.08	0.64	1.00	2557.73
β_{δ_1}	0.90	0.06	0.79	0.97	1.00	1422.99
β_{δ_2}	0.87	0.08	0.72	0.98	1.00	6766.54
β_{δ_3}	0.87	0.09	0.71	0.98	1.00	5835.43
β_{δ_4}	0.86	0.09	0.69	0.97	1.00	5593.23
β_{δ_5}	0.86	0.09	0.70	0.97	1.00	7906.93
β_{δ_6}	0.83	0.10	0.65	0.96	1.00	5050.43
β_{δ_7}	0.86	0.09	0.69	0.97	1.00	7841.22
β_{δ_8}	0.86	0.09	0.68	0.97	1.00	7866.09
β_{δ_9}	0.86	0.09	0.70	0.97	1.00	7654.57
$\beta_{\delta_{10}}$	0.88	0.08	0.72	0.98	1.00	7707.68
κ_σ	0.26	0.14	0.04	0.49	1.01	241.47
η_σ	0.47	0.10	0.31	0.60	1.01	420.74
π_σ	0.73	0.13	0.52	0.94	1.02	313.60

Table S5. Mean, standard deviation, 5.5% and 94.5% intervals, number of effective samples and Rhat values, for model parameters—modeling association between father status and child survival.

	mean	sd	5.5%	94.5%	rhat	ess_bulk
α	3.31	1.46	0.88	5.54	1.00	2431.40
γ_τ	0.95	0.98	0.05	2.83	1.00	5104.86
γ_κ	0.87	0.09	0.71	0.97	1.00	5138.83
γ_δ	0.33	0.23	0.04	0.73	1.00	1414.00
ϵ_τ	2.47	1.29	0.88	4.79	1.00	3805.64
ϵ_κ	0.93	0.05	0.82	0.99	1.00	2443.74
ϵ_δ	0.68	0.30	0.34	1.20	1.00	2474.28
β_{τ_1}	1.73	1.08	0.51	3.77	1.00	1929.45
β_{τ_2}	0.94	0.98	0.04	2.73	1.00	5033.87
β_{τ_3}	0.95	0.99	0.05	2.82	1.00	4553.63
β_{τ_4}	0.95	1.00	0.04	2.89	1.00	4764.11
β_{τ_5}	1.13	1.12	0.06	3.30	1.00	4725.66
β_{τ_6}	0.99	1.02	0.05	2.89	1.00	5947.94
β_{τ_7}	0.94	0.95	0.06	2.70	1.00	5120.22
β_{τ_8}	0.96	0.94	0.05	2.85	1.00	5047.40
β_{τ_9}	0.87	0.91	0.04	2.66	1.00	5116.27
β_{κ_1}	2.28	0.69	1.41	3.48	1.00	2299.13
β_{κ_2}	0.32	0.23	0.07	0.74	1.00	1467.61
β_{κ_3}	0.70	0.37	0.28	1.38	1.00	2517.69
β_{κ_4}	0.49	0.45	0.03	1.36	1.00	2842.64
β_{κ_5}	1.00	0.63	0.17	2.19	1.00	1437.14
β_{κ_6}	0.31	0.30	0.02	0.87	1.00	1915.11
β_{κ_7}	0.49	0.47	0.03	1.36	1.00	1661.78
β_{κ_8}	0.49	0.46	0.04	1.33	1.00	1747.80
β_{κ_9}	0.30	0.19	0.08	0.64	1.00	2268.30
β_{δ_1}	0.90	0.06	0.79	0.97	1.00	1644.92
β_{δ_2}	0.87	0.08	0.71	0.98	1.00	5787.77
β_{δ_3}	0.87	0.09	0.71	0.97	1.00	5694.67
β_{δ_4}	0.86	0.09	0.69	0.97	1.00	5673.82
β_{δ_5}	0.86	0.09	0.69	0.97	1.00	6219.07
β_{δ_6}	0.86	0.09	0.69	0.97	1.00	7371.21
β_{δ_7}	0.85	0.09	0.69	0.97	1.00	7233.11
β_{δ_8}	0.86	0.09	0.69	0.97	1.00	6158.01
β_{δ_9}	0.88	0.08	0.73	0.98	1.00	5848.97
κ_σ	0.48	0.09	0.33	0.61	1.01	543.17
η_σ	0.25	0.14	0.03	0.48	1.02	308.30
π_σ	0.73	0.13	0.52	0.94	1.01	568.89

Table S6. Mean, standard deviation, 5.5% and 94.5% intervals, number of effective samples and Rhat values, for model parameters—modeling association between mother status and child survival.

	mean	sd	5.5%	94.5%	n_eff	Rhat4
α	4.47	0.39	3.81	4.95	944.65	1.00
γ_τ	0.75	0.80	0.03	2.28	4779.27	1.00
γ_κ	0.87	0.08	0.72	0.98	3890.70	1.00
γ_δ	0.02	0.02	0.01	0.04	529.41	1.01
ϵ_τ	0.77	0.81	0.03	2.27	3768.51	1.00
ϵ_κ	0.87	0.09	0.70	0.97	3940.12	1.00
ϵ_δ	0.03	0.02	0.01	0.07	1792.55	1.00
β_{τ_1}	2.23	0.89	1.00	3.82	1504.86	1.00
β_{τ_2}	0.97	0.98	0.05	2.78	6033.60	1.00
β_{τ_3}	0.82	0.87	0.04	2.40	4879.62	1.00
β_{τ_4}	1.01	1.03	0.06	2.96	4938.85	1.00
β_{τ_5}	0.95	0.92	0.06	2.76	6061.92	1.00
β_{τ_6}	1.29	0.98	0.21	3.12	3311.34	1.00
β_{τ_7}	1.00	1.00	0.05	2.87	5952.11	1.00
β_{τ_8}	0.97	1.01	0.05	2.96	5309.95	1.00
β_{τ_9}	1.08	1.03	0.08	3.08	6071.16	1.00
$\beta_{\tau_{10}}$	1.03	0.91	0.11	2.73	4088.68	1.00
β_{κ_1}	0.44	0.20	0.23	0.80	1012.51	1.00
β_{κ_2}	0.01	0.01	0.00	0.02	1524.37	1.00
β_{κ_3}	0.05	0.04	0.01	0.11	1086.93	1.00
β_{κ_4}	0.04	0.03	0.00	0.10	1624.10	1.00
β_{κ_5}	0.01	0.01	0.00	0.03	2611.04	1.00
β_{κ_6}	0.06	0.03	0.02	0.12	1160.17	1.01
β_{κ_7}	0.01	0.01	0.00	0.04	1854.83	1.00
β_{κ_8}	0.04	0.04	0.00	0.12	1673.78	1.00
β_{κ_9}	0.03	0.02	0.00	0.07	1230.07	1.00
$\beta_{\kappa_{10}}$	0.03	0.02	0.01	0.06	1699.07	1.00
β_{δ_1}	1.00	0.00	1.00	1.00	1441.37	1.00
β_{δ_2}	0.86	0.09	0.71	0.97	6433.87	1.00
β_{δ_3}	0.85	0.09	0.69	0.97	4844.77	1.00
β_{δ_4}	0.86	0.09	0.69	0.97	5333.86	1.00
β_{δ_5}	0.87	0.09	0.70	0.97	5587.01	1.00
β_{δ_6}	0.89	0.08	0.75	0.98	3437.18	1.00
β_{δ_7}	0.87	0.09	0.70	0.97	5984.43	1.00
β_{δ_8}	0.86	0.09	0.70	0.97	5925.08	1.00
β_{δ_9}	0.88	0.09	0.71	0.98	5119.29	1.00
$\beta_{\delta_{10}}$	0.87	0.09	0.71	0.97	3663.99	1.00
κ_σ	0.02	0.00	0.01	0.03	594.08	1.01
η_σ	0.03	0.00	0.02	0.03	852.06	1.01
π_σ						

Table S7. Mean, standard deviation, 5.5% and 94.5% intervals, number of effective samples and Rhat values, for model parameters—modeling association between father status and child height.

	mean	sd	5.5%	94.5%	rhat	ess_bulk
α	4.44	0.46	3.76	4.98	1.00	1283.09
γ_τ	0.69	0.76	0.03	2.09	1.00	4252.54
γ_κ	0.86	0.09	0.70	0.97	1.00	4046.89
γ_δ	0.02	0.01	0.01	0.05	1.00	1558.39
ϵ_τ	0.86	0.90	0.04	2.56	1.00	2696.86
ϵ_κ	0.87	0.08	0.72	0.98	1.00	3769.87
ϵ_δ	0.04	0.02	0.02	0.09	1.00	2192.92
β_{τ_1}	2.16	0.86	0.94	3.63	1.00	1604.13
β_{τ_2}	0.92	0.93	0.05	2.80	1.00	4503.99
β_{τ_3}	0.91	0.93	0.05	2.73	1.00	4819.29
β_{τ_4}	0.93	0.96	0.05	2.75	1.00	3579.65
β_{τ_5}	1.11	1.12	0.07	3.22	1.00	3858.58
β_{τ_6}	1.35	0.97	0.24	3.17	1.00	3835.14
β_{τ_7}	0.94	0.96	0.05	2.68	1.00	4592.95
β_{τ_8}	1.13	1.07	0.08	3.17	1.00	3138.21
β_{τ_9}	1.36	1.05	0.21	3.30	1.00	3639.24
β_{κ_1}	0.46	0.23	0.24	0.86	1.00	1554.04
β_{κ_2}	0.01	0.01	0.00	0.02	1.00	2057.89
β_{κ_3}	0.02	0.02	0.00	0.05	1.00	1818.02
β_{κ_4}	0.01	0.01	0.00	0.03	1.00	2640.70
β_{κ_5}	0.04	0.03	0.00	0.10	1.00	1520.10
β_{κ_6}	0.03	0.02	0.01	0.06	1.00	1389.31
β_{κ_7}	0.01	0.01	0.00	0.03	1.00	2114.58
β_{κ_8}	0.03	0.02	0.00	0.06	1.00	1347.49
β_{κ_9}	0.03	0.01	0.01	0.05	1.00	1730.44
β_{δ_1}	1.00	0.00	1.00	1.00	1.00	1352.61
β_{δ_2}	0.87	0.09	0.71	0.98	1.00	5770.42
β_{δ_3}	0.88	0.08	0.73	0.98	1.00	5420.67
β_{δ_4}	0.87	0.09	0.70	0.97	1.00	6037.25
β_{δ_5}	0.87	0.09	0.71	0.97	1.00	5152.71
β_{δ_6}	0.90	0.07	0.77	0.98	1.00	2933.67
β_{δ_7}	0.86	0.09	0.70	0.97	1.00	5010.25
β_{δ_8}	0.88	0.08	0.72	0.98	1.00	4278.78
β_{δ_9}	0.89	0.07	0.75	0.98	1.00	2928.18
κ_σ	0.03	0.00	0.02	0.03	1.00	656.72
η_σ	0.03	0.00	0.03	0.04	1.01	708.47
π_σ	0.06	0.00	0.05	0.06	1.00	1325.68

Table S8. Mean, standard deviation, 5.5% and 94.5% intervals, number of effective samples and Rhat values, for model parameters—modeling association between mother status and child height.

	mean	sd	5.5%	94.5%	rhat	ess_bulk
α	-0.97	1.77	-3.71	1.94	1.01	301.77
γ_τ	0.76	0.80	0.05	2.24	1.00	777.69
γ_κ	0.87	0.09	0.72	0.98	1.01	579.51
γ_δ	0.04	0.02	0.01	0.08	1.01	395.79
ϵ_τ	1.39	1.10	0.18	3.53	1.01	429.65
ϵ_κ	0.92	0.07	0.80	0.99	1.00	451.58
ϵ_δ	0.05	0.03	0.02	0.10	1.01	429.05
β_{τ_1}	1.11	0.48	0.50	1.97	1.01	293.85
β_{τ_2}	1.95	1.47	0.39	4.86	1.00	372.16
β_{τ_3}	0.92	0.95	0.05	2.73	1.00	793.44
β_{τ_4}	1.22	1.03	0.13	3.19	1.01	606.80
β_{τ_5}	1.00	0.91	0.08	2.70	1.01	747.44
β_{τ_6}	0.90	0.95	0.04	2.62	1.01	859.99
β_{τ_7}	1.41	1.10	0.19	3.53	1.00	763.76
β_{τ_8}	0.95	0.99	0.05	2.74	1.00	979.94
β_{τ_9}	0.92	0.96	0.06	2.69	1.00	859.55
$\beta_{\tau_{10}}$	1.34	1.16	0.11	3.52	1.01	732.11
$\beta_{\tau_{11}}$	0.69	0.34	0.26	1.30	1.02	326.86
β_{κ_1}	2.90	0.87	1.73	4.48	1.03	335.16
β_{κ_2}	0.09	0.04	0.05	0.17	1.01	500.60
β_{κ_3}	0.03	0.04	0.00	0.10	1.01	404.75
β_{κ_4}	0.14	0.08	0.05	0.28	1.01	344.06
β_{κ_5}	0.05	0.03	0.01	0.11	1.01	393.43
β_{κ_6}	0.03	0.03	0.00	0.09	1.02	363.97
β_{κ_7}	0.04	0.03	0.01	0.10	1.01	215.43
β_{κ_8}	0.05	0.05	0.01	0.14	1.01	553.26
β_{κ_9}	0.02	0.03	0.00	0.07	1.00	485.37
$\beta_{\kappa_{10}}$	0.02	0.02	0.00	0.06	1.01	350.46
$\beta_{\kappa_{11}}$	1.07	0.34	0.68	1.66	1.02	302.37
β_{δ_1}	1.00	0.00	1.00	1.00	1.02	290.23
β_{δ_2}	0.93	0.06	0.83	0.99	1.01	343.09
β_{δ_3}	0.86	0.09	0.70	0.97	1.00	894.38
β_{δ_4}	0.89	0.08	0.74	0.98	1.00	597.04
β_{δ_5}	0.88	0.08	0.73	0.98	1.00	983.82
β_{δ_6}	0.86	0.09	0.69	0.97	1.00	906.26
β_{δ_7}	0.90	0.08	0.76	0.98	1.00	749.02
β_{δ_8}	0.87	0.09	0.70	0.97	1.00	759.81
β_{δ_9}	0.86	0.09	0.70	0.97	1.01	966.81
$\beta_{\delta_{10}}$	0.88	0.08	0.72	0.98	1.00	814.75
$\beta_{\delta_{11}}$	1.00	0.00	1.00	1.00	1.01	326.82
κ_σ	0.05	0.01	0.04	0.06	1.02	185.62
η_σ	0.03	0.01	0.03	0.04	1.03	141.10
π_σ	0.09	0.01	0.08	0.09	1.00	460.82

Table S9. Mean, standard deviation, 5.5% and 94.5% intervals, number of effective samples and Rhat values, for model parameters—modeling association between father status and child weight.

	mean	sd	5.5%	94.5%	rhat	ess_bulk
α	-0.98	1.75	-3.72	1.97	1.01	415.06
γ_τ	0.73	0.83	0.03	2.30	1.02	504.30
γ_κ	0.87	0.09	0.71	0.97	1.00	620.40
γ_δ	0.03	0.02	0.01	0.07	1.02	239.11
ϵ_τ	1.50	1.20	0.20	3.83	1.02	411.41
ϵ_κ	0.92	0.07	0.79	0.99	1.01	570.24
ϵ_δ	0.05	0.03	0.02	0.11	1.01	458.47
β_{τ_1}	1.07	0.48	0.46	1.90	1.01	372.51
β_{τ_2}	2.02	1.43	0.43	4.73	1.02	342.88
β_{τ_3}	0.90	0.94	0.05	2.71	1.00	859.27
β_{τ_4}	0.91	0.90	0.06	2.62	1.01	799.74
β_{τ_5}	1.39	1.11	0.18	3.50	1.01	709.92
β_{τ_6}	0.97	0.95	0.06	2.77	1.00	724.39
β_{τ_7}	1.05	0.96	0.07	2.84	1.00	1118.35
β_{τ_8}	0.90	0.94	0.04	2.75	1.00	835.98
β_{τ_9}	1.37	1.21	0.10	3.67	1.00	728.24
$\beta_{\tau_1 0}$	0.70	0.33	0.29	1.27	1.00	364.29
β_{κ_1}	3.01	0.91	1.81	4.65	1.01	365.27
β_{κ_2}	0.09	0.04	0.04	0.16	1.01	490.44
β_{κ_3}	0.03	0.04	0.00	0.10	1.01	394.20
β_{κ_4}	0.05	0.03	0.02	0.11	1.00	597.80
β_{κ_5}	0.16	0.08	0.06	0.31	1.01	477.55
β_{κ_6}	0.03	0.02	0.00	0.07	1.01	287.92
β_{κ_7}	0.02	0.02	0.00	0.05	1.00	563.72
β_{κ_8}	0.02	0.02	0.00	0.07	1.01	520.33
β_{κ_9}	0.03	0.02	0.00	0.06	1.00	411.31
$\beta_{\kappa_1 0}$	1.10	0.34	0.68	1.71	1.01	349.47
β_{δ_1}	1.00	0.00	1.00	1.00	1.01	325.06
β_{δ_2}	0.94	0.05	0.84	0.99	1.02	356.40
β_{δ_3}	0.85	0.09	0.69	0.97	1.00	875.24
β_{δ_4}	0.89	0.08	0.75	0.98	1.00	795.67
β_{δ_5}	0.89	0.08	0.76	0.98	1.00	768.92
β_{δ_6}	0.87	0.09	0.71	0.97	1.00	1073.93
β_{δ_7}	0.87	0.08	0.71	0.97	1.00	1213.60
β_{δ_8}	0.86	0.09	0.70	0.97	1.00	966.18
β_{δ_9}	0.88	0.08	0.73	0.98	1.00	672.48
$\beta_{\delta_1 0}$	1.00	0.00	1.00	1.00	1.01	290.76
κ_σ	0.04	0.00	0.03	0.04	1.03	159.92
η_σ	0.05	0.00	0.04	0.06	1.02	185.71
π_σ	0.09	0.01	0.08	0.09	1.01	636.77

Table S10. Mean, standard deviation, 5.5% and 94.5% intervals, number of effective samples and Rhat values, for model parameters—modeling association between mother status and child weight.

	mean	sd	5.5%	94.5%	rhat	ess_bulk
α	0.81	1.07	-0.84	2.50	1.00	3149.28
γ_τ	1.01	1.01	0.05	2.93	1.00	5078.16
γ_κ	0.89	0.08	0.74	0.98	1.00	5597.06
γ_δ	0.06	0.05	0.01	0.15	1.00	1549.32
ϵ_τ	2.00	1.42	0.35	4.64	1.00	2171.57
ϵ_κ	0.94	0.06	0.82	0.99	1.00	2273.51
ϵ_δ	0.37	0.19	0.17	0.71	1.00	3291.41
β_{τ_1}	2.75	1.31	1.04	5.11	1.01	1048.62
β_{τ_2}	0.91	0.92	0.05	2.67	1.00	5248.63
β_{τ_3}	0.91	0.92	0.05	2.67	1.00	4921.00
β_{τ_4}	0.96	0.98	0.05	2.89	1.00	5768.82
β_{τ_5}	1.00	0.99	0.05	2.90	1.00	5117.88
β_{τ_6}	0.98	0.99	0.05	2.87	1.00	5623.87
β_{τ_7}	0.97	0.96	0.06	2.70	1.00	5550.98
β_{τ_8}	0.98	0.99	0.05	2.90	1.00	4237.19
β_{τ_9}	0.91	0.91	0.05	2.64	1.00	4810.49
β_{τ_10}	0.97	0.98	0.05	2.90	1.00	5053.24
β_{κ_1}	1.56	0.57	0.88	2.63	1.00	2451.30
β_{κ_2}	0.05	0.05	0.00	0.14	1.00	2030.18
β_{κ_3}	0.15	0.12	0.02	0.35	1.00	2269.12
β_{κ_4}	0.10	0.10	0.01	0.28	1.00	2528.71
β_{κ_5}	0.08	0.09	0.00	0.24	1.00	2602.55
β_{κ_6}	0.07	0.07	0.00	0.21	1.00	2725.06
β_{κ_7}	0.08	0.08	0.01	0.22	1.00	2490.09
β_{κ_8}	0.14	0.14	0.01	0.39	1.00	2794.79
β_{κ_9}	0.12	0.10	0.02	0.29	1.00	2760.18
β_{κ_10}	0.05	0.05	0.00	0.15	1.00	2728.36
β_{δ_1}	0.99	0.01	0.97	1.00	1.01	501.51
β_{δ_2}	0.87	0.09	0.71	0.98	1.00	8303.70
β_{δ_3}	0.87	0.08	0.72	0.98	1.00	8001.04
β_{δ_4}	0.87	0.09	0.71	0.97	1.00	7399.80
β_{δ_5}	0.87	0.09	0.71	0.97	1.00	6880.14
β_{δ_6}	0.86	0.09	0.69	0.97	1.00	7191.57
β_{δ_7}	0.86	0.09	0.71	0.97	1.00	7593.91
β_{δ_8}	0.86	0.09	0.70	0.97	1.00	7202.75
β_{δ_9}	0.87	0.08	0.72	0.97	1.00	6333.44
β_{δ_10}	0.87	0.09	0.71	0.97	1.00	6915.87
κ_σ	0.03	0.02	0.00	0.07	1.00	954.67
η_σ	0.03	0.02	0.00	0.07	1.01	944.92

Table S11. Mean, standard deviation, 5.5% and 94.5% intervals, number of effective samples and Rhat values, for model parameters—modeling association between father status and child education: parameters for the model of θ i.e., modeling the probability a child does not go to school.

	mean	sd	5.5%	94.5%	rhat	ess_bulk
α	1.62	1.78	-1.21	4.49	1.00	5966.41
γ_τ	1.65	1.30	0.17	4.03	1.00	3317.07
γ_κ	0.89	0.08	0.74	0.98	1.00	3178.61
γ_δ	0.91	0.42	0.41	1.67	1.00	2384.23
ϵ_τ	2.95	1.70	0.84	6.07	1.00	2630.17
ϵ_κ	0.92	0.05	0.82	0.98	1.00	2867.90
ϵ_δ	2.83	0.88	1.72	4.42	1.00	3189.86
β_{τ_1}	2.09	0.86	1.05	3.69	1.00	3144.36
β_{τ_2}	1.05	1.02	0.07	2.96	1.00	5375.37
β_{τ_3}	0.95	0.99	0.04	2.87	1.00	4832.75
β_{τ_4}	1.09	1.02	0.08	2.99	1.00	5070.48
β_{τ_5}	1.04	1.04	0.07	2.96	1.00	5200.51
β_{τ_6}	0.94	0.99	0.05	2.84	1.00	4945.94
β_{τ_7}	1.01	1.04	0.05	3.03	1.00	5238.28
β_{τ_8}	0.99	1.03	0.05	2.92	1.00	4779.99
β_{τ_9}	0.96	0.96	0.05	2.80	1.00	4779.55
β_{τ_10}	1.45	1.19	0.15	3.76	1.00	2969.02
β_{κ_1}	4.24	1.05	2.84	6.12	1.00	3601.01
β_{κ_2}	0.32	0.29	0.03	0.85	1.00	1707.18
β_{κ_3}	0.65	0.60	0.05	1.78	1.00	2388.06
β_{κ_4}	1.18	0.85	0.11	2.75	1.00	1964.03
β_{κ_5}	0.93	0.73	0.07	2.28	1.00	1441.19
β_{κ_6}	1.12	0.93	0.06	2.86	1.01	799.63
β_{κ_7}	0.66	0.63	0.04	1.86	1.00	1988.85
β_{κ_8}	0.86	0.74	0.08	2.17	1.00	2727.15
β_{κ_9}	1.16	0.73	0.22	2.51	1.00	2117.94
β_{κ_10}	1.15	0.59	0.33	2.14	1.01	1298.65
β_{δ_1}	0.99	0.01	0.97	1.00	1.00	1541.22
β_{δ_2}	0.87	0.09	0.71	0.97	1.00	6616.30
β_{δ_3}	0.85	0.09	0.68	0.97	1.00	6580.22
β_{δ_4}	0.85	0.09	0.69	0.97	1.00	5405.69
β_{δ_5}	0.86	0.09	0.69	0.97	1.00	5144.66
β_{δ_6}	0.82	0.10	0.64	0.96	1.00	3511.13
β_{δ_7}	0.85	0.09	0.67	0.97	1.00	6613.95
β_{δ_8}	0.86	0.09	0.69	0.97	1.00	7424.71
β_{δ_9}	0.86	0.09	0.69	0.97	1.00	5666.23
β_{δ_10}	0.89	0.08	0.74	0.98	1.00	3762.10
κ_σ	1.06	0.27	0.61	1.46	1.01	331.22
η_σ	1.76	0.18	1.47	2.05	1.00	1145.33

Table S12. Mean, standard deviation, 5.5% and 94.5% intervals, number of effective samples and Rhat values, for model parameters—modeling association between father status and child education: parameters for the model of η, i.e., modeling years of schooling, conditional on attending school.

	mean	sd	5.5%	94.5%	rhat	ess_bulk
α	0.80	1.02	-0.84	2.45	1.00	2680.70
γ_{τ}	1.01	0.99	0.06	2.87	1.00	4055.18
γ_{κ}	0.89	0.08	0.73	0.98	1.00	4629.05
γ_{δ}	0.06	0.05	0.00	0.15	1.00	1500.79
ϵ_{τ}	1.98	1.41	0.35	4.60	1.00	1999.83
ϵ_{κ}	0.94	0.06	0.82	0.99	1.00	2034.54
ϵ_{δ}	0.37	0.18	0.17	0.70	1.00	2638.72
β_{τ_1}	2.85	1.33	1.10	5.17	1.00	965.59
β_{τ_2}	0.91	0.95	0.05	2.70	1.00	3899.35
β_{τ_3}	0.93	0.93	0.06	2.65	1.00	3649.50
β_{τ_4}	0.98	0.96	0.06	2.84	1.00	3808.60
β_{τ_5}	0.93	0.93	0.05	2.68	1.00	4193.76
β_{τ_6}	1.02	1.04	0.06	3.02	1.00	4185.51
β_{τ_7}	0.95	0.99	0.05	2.82	1.00	4049.43
β_{τ_8}	0.92	0.97	0.05	2.72	1.00	3913.58
β_{τ_9}	0.95	0.97	0.05	2.78	1.00	3928.50
β_{κ_1}	1.54	0.57	0.86	2.58	1.00	2374.98
β_{κ_2}	0.05	0.05	0.01	0.14	1.00	1865.13
β_{κ_3}	0.14	0.12	0.02	0.35	1.00	1486.96
β_{κ_4}	0.08	0.08	0.01	0.24	1.00	2414.75
β_{κ_5}	0.10	0.10	0.01	0.29	1.00	2404.16
β_{κ_6}	0.07	0.07	0.00	0.19	1.00	2059.98
β_{κ_7}	0.07	0.08	0.00	0.21	1.00	1978.33
β_{κ_8}	0.12	0.10	0.01	0.30	1.00	2005.64
β_{κ_9}	0.05	0.05	0.00	0.15	1.00	2294.38
β_{δ_1}	0.99	0.01	0.98	1.00	1.01	586.99
β_{δ_2}	0.88	0.09	0.72	0.98	1.00	4365.36
β_{δ_3}	0.87	0.09	0.71	0.98	1.00	5082.91
β_{δ_4}	0.87	0.09	0.70	0.97	1.00	5911.94
β_{δ_5}	0.87	0.09	0.70	0.97	1.00	5538.75
β_{δ_6}	0.87	0.09	0.70	0.97	1.00	5127.62
β_{δ_7}	0.86	0.09	0.70	0.97	1.00	5337.13
β_{δ_8}	0.87	0.08	0.72	0.98	1.00	4534.14
β_{δ_9}	0.87	0.09	0.71	0.98	1.00	4542.33
κ_{σ}	0.03	0.02	0.00	0.07	1.00	761.80
η_{σ}	0.03	0.02	0.00	0.07	1.01	669.28

Table S13. Mean, standard deviation, 5.5% and 94.5% intervals, number of effective samples and Rhat values, for model parameters—modeling association between mother status and child education: parameters for the model of θ, i.e., modeling the probability a child does not go to school.

	mean	sd	5.5%	94.5%	rhat	ess_bulk
α	1.63	1.89	-1.37	4.75	1.00	4149.39
γ_τ	1.63	1.27	0.17	3.97	1.00	2640.17
γ_κ	0.88	0.08	0.73	0.98	1.00	2865.83
γ_δ	0.88	0.38	0.41	1.58	1.00	2033.22
ϵ_τ	2.96	1.68	0.90	5.99	1.00	1965.14
ϵ_κ	0.92	0.05	0.82	0.98	1.00	2242.91
ϵ_δ	2.83	0.89	1.69	4.39	1.00	2662.37
β_{τ_1}	2.08	0.87	1.04	3.66	1.00	2232.69
β_{τ_2}	1.07	1.07	0.07	3.11	1.00	4355.41
β_{τ_3}	0.95	0.97	0.05	2.84	1.00	3608.43
β_{τ_4}	0.97	0.95	0.06	2.79	1.00	3701.19
β_{τ_5}	1.15	1.09	0.08	3.25	1.00	4463.45
β_{τ_6}	0.97	0.93	0.07	2.73	1.00	4578.90
β_{τ_7}	1.03	0.91	0.08	2.70	1.00	2961.91
β_{τ_8}	0.97	1.02	0.05	2.82	1.00	4213.56
β_{τ_9}	1.47	1.13	0.18	3.64	1.00	2965.63
β_{κ_1}	4.26	1.03	2.83	6.09	1.00	2790.22
β_{κ_2}	0.33	0.30	0.03	0.89	1.00	1729.57
β_{κ_3}	0.68	0.62	0.05	1.83	1.00	1927.84
β_{κ_4}	0.82	0.72	0.06	2.16	1.00	1576.66
β_{κ_5}	1.15	0.89	0.08	2.74	1.00	1517.68
β_{κ_6}	0.37	0.36	0.02	1.03	1.00	2365.67
β_{κ_7}	1.43	0.72	0.47	2.71	1.00	1232.06
β_{κ_8}	1.12	0.75	0.16	2.45	1.00	1436.38
β_{κ_9}	1.29	0.59	0.49	2.33	1.00	1517.61
β_{δ_1}	0.99	0.01	0.97	1.00	1.00	1484.04
β_{δ_2}	0.87	0.09	0.71	0.97	1.00	4704.28
β_{δ_3}	0.86	0.09	0.69	0.97	1.00	4506.75
β_{δ_4}	0.85	0.09	0.68	0.97	1.00	4857.12
β_{δ_5}	0.85	0.09	0.68	0.97	1.00	4496.57
β_{δ_6}	0.86	0.09	0.69	0.97	1.00	5957.39
β_{δ_7}	0.86	0.09	0.70	0.97	1.00	4062.69
β_{δ_8}	0.86	0.09	0.69	0.97	1.00	4436.53
β_{δ_9}	0.89	0.07	0.75	0.98	1.00	3137.06
κ_σ	1.09	0.28	0.63	1.49	1.00	329.51
η_σ	1.73	0.18	1.45	2.04	1.00	860.47

Table S14. Mean, standard deviation, 5.5% and 94.5% intervals, number of effective samples and Rhat values, for model parameters—modeling association between mother status and child education: parameters for the model of η, i.e., modeling years of schooling, conditional on attending school.

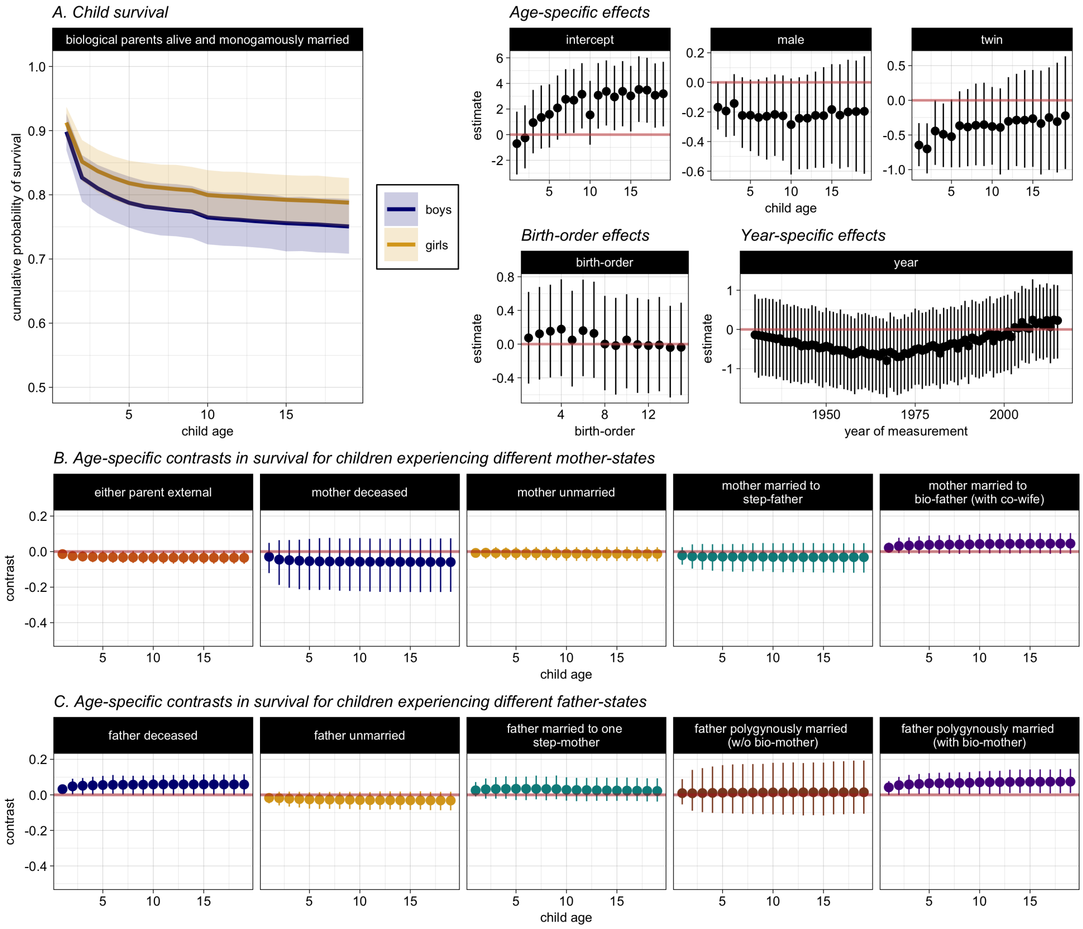

Fig. S24. Association between parent marital/vital states in year $t-1$ and child survival in year t.

	mean	sd	5.5%	94.5%	rhat	ess_bulk
α	3.26	1.48	0.78	5.50	1.00	3701.58
γ_τ	1.01	1.02	0.06	2.86	1.00	5074.93
γ_κ	0.87	0.08	0.71	0.97	1.00	6072.67
γ_δ	0.32	0.24	0.04	0.74	1.00	1531.80
ϵ_τ	2.45	1.26	0.89	4.78	1.00	3311.72
ϵ_κ	0.93	0.05	0.83	0.99	1.00	2839.02
ϵ_δ	0.69	0.30	0.36	1.24	1.00	2752.95
β_{τ_1}	1.78	1.14	0.52	3.93	1.00	2100.48
β_{τ_2}	0.94	1.00	0.05	2.84	1.00	4815.06
β_{τ_3}	0.97	0.98	0.05	2.86	1.00	5344.48
β_{τ_4}	0.99	0.99	0.06	2.86	1.00	5189.78
β_{τ_5}	0.99	0.98	0.06	2.84	1.00	5344.28
β_{τ_6}	0.98	1.00	0.06	2.85	1.00	6142.41
β_{τ_7}	0.98	1.00	0.06	2.84	1.00	6352.91
β_{τ_8}	0.95	0.94	0.06	2.75	1.00	5705.40
β_{τ_9}	0.95	0.95	0.05	2.81	1.00	4758.09
β_{κ_1}	2.33	0.71	1.42	3.58	1.00	2610.07
β_{κ_2}	0.32	0.23	0.08	0.74	1.00	2286.38
β_{κ_3}	0.72	0.41	0.29	1.43	1.00	2152.78
β_{κ_4}	0.63	0.47	0.15	1.52	1.00	2553.50
β_{κ_5}	0.58	0.55	0.04	1.60	1.00	3229.17
β_{κ_6}	0.31	0.31	0.02	0.90	1.00	1349.75
β_{κ_7}	0.46	0.46	0.03	1.35	1.00	2200.36
β_{κ_8}	0.54	0.48	0.05	1.43	1.00	2003.31
β_{κ_9}	0.26	0.19	0.04	0.59	1.00	2188.14
β_{δ_1}	0.91	0.06	0.80	0.97	1.00	1922.54
β_{δ_2}	0.87	0.09	0.72	0.98	1.00	6754.20
β_{δ_3}	0.87	0.09	0.71	0.97	1.00	6509.77
β_{δ_4}	0.86	0.09	0.70	0.97	1.00	7930.54
β_{δ_5}	0.86	0.09	0.68	0.97	1.00	8703.36
β_{δ_6}	0.86	0.09	0.69	0.97	1.00	6913.88
β_{δ_7}	0.85	0.09	0.68	0.97	1.00	7698.75
β_{δ_8}	0.87	0.09	0.70	0.97	1.00	7360.43
β_{δ_9}	0.87	0.09	0.72	0.98	1.00	6246.34
κ_σ	0.48	0.09	0.32	0.61	1.01	591.06
η_σ	0.29	0.15	0.04	0.52	1.03	225.79
π_σ	0.77	0.13	0.55	0.97	1.01	351.62

Table S15. Mean, standard deviation, 5.5% and 94.5% intervals, number of effective samples and Rhat values, for model parameters—modeling association between mother status at $t-1$ and child survival at t.

	mean	sd	5.5%	94.5%	rhat	ess_bulk
α	3.31	1.45	0.87	5.54	1.00	3267.48
γ_τ	0.99	1.01	0.06	2.92	1.00	4453.75
γ_κ	0.87	0.09	0.71	0.98	1.00	5616.13
γ_δ	0.31	0.23	0.04	0.72	1.00	1164.98
ϵ_τ	2.40	1.22	0.88	4.61	1.00	3383.02
ϵ_κ	0.93	0.05	0.83	0.99	1.00	3037.45
ϵ_δ	0.70	0.30	0.36	1.25	1.00	2974.80
β_{τ_1}	1.74	1.10	0.54	3.75	1.00	1741.82
β_{τ_2}	0.94	0.92	0.06	2.71	1.00	4268.79
β_{τ_3}	0.97	1.00	0.05	2.84	1.00	4792.56
β_{τ_4}	0.98	1.02	0.05	2.84	1.00	4867.38
β_{τ_5}	0.98	0.96	0.06	2.82	1.00	5379.44
β_{τ_6}	0.93	0.97	0.05	2.76	1.00	5567.74
β_{τ_7}	1.21	1.05	0.10	3.15	1.00	4082.94
β_{τ_8}	1.00	1.00	0.06	2.97	1.00	5855.57
β_{τ_9}	0.90	0.93	0.05	2.62	1.00	4912.86
$\beta_{\tau_{10}}$	0.94	0.93	0.05	2.67	1.00	5769.53
β_{κ_1}	2.30	0.71	1.42	3.58	1.00	2326.29
β_{κ_2}	0.31	0.21	0.06	0.69	1.00	1455.06
β_{κ_3}	0.72	0.39	0.29	1.40	1.00	2810.39
β_{κ_4}	0.52	0.50	0.04	1.47	1.00	2341.22
β_{κ_5}	0.65	0.48	0.16	1.55	1.00	2332.23
β_{κ_6}	0.42	0.38	0.03	1.15	1.00	1703.48
β_{κ_7}	0.65	0.53	0.05	1.63	1.00	1468.86
β_{κ_8}	0.72	0.74	0.04	2.12	1.00	3608.67
β_{κ_9}	0.84	0.63	0.15	1.99	1.00	2313.27
$\beta_{\kappa_{10}}$	0.26	0.20	0.04	0.62	1.00	2317.06
β_{δ_1}	0.90	0.06	0.79	0.97	1.00	1860.21
β_{δ_2}	0.87	0.09	0.71	0.98	1.00	6907.32
β_{δ_3}	0.87	0.09	0.70	0.97	1.00	6392.97
β_{δ_4}	0.86	0.09	0.68	0.97	1.00	6419.62
β_{δ_5}	0.86	0.09	0.69	0.97	1.00	6956.20
β_{δ_6}	0.86	0.09	0.69	0.97	1.00	6074.56
β_{δ_7}	0.86	0.09	0.70	0.97	1.00	5800.53
β_{δ_8}	0.86	0.09	0.69	0.97	1.00	7358.39
β_{δ_9}	0.86	0.09	0.69	0.97	1.00	5624.82
$\beta_{\delta_{10}}$	0.87	0.09	0.71	0.97	1.00	5997.70
κ_σ	0.27	0.15	0.03	0.50	1.05	101.84
η_σ	0.48	0.09	0.34	0.61	1.01	464.52
π_σ	0.75	0.13	0.53	0.97	1.04	257.19

Table S16. Mean, standard deviation, 5.5% and 94.5% intervals, number of effective samples and Rhat values, for model parameters—modeling association between father status at $t-1$ and child survival at t.

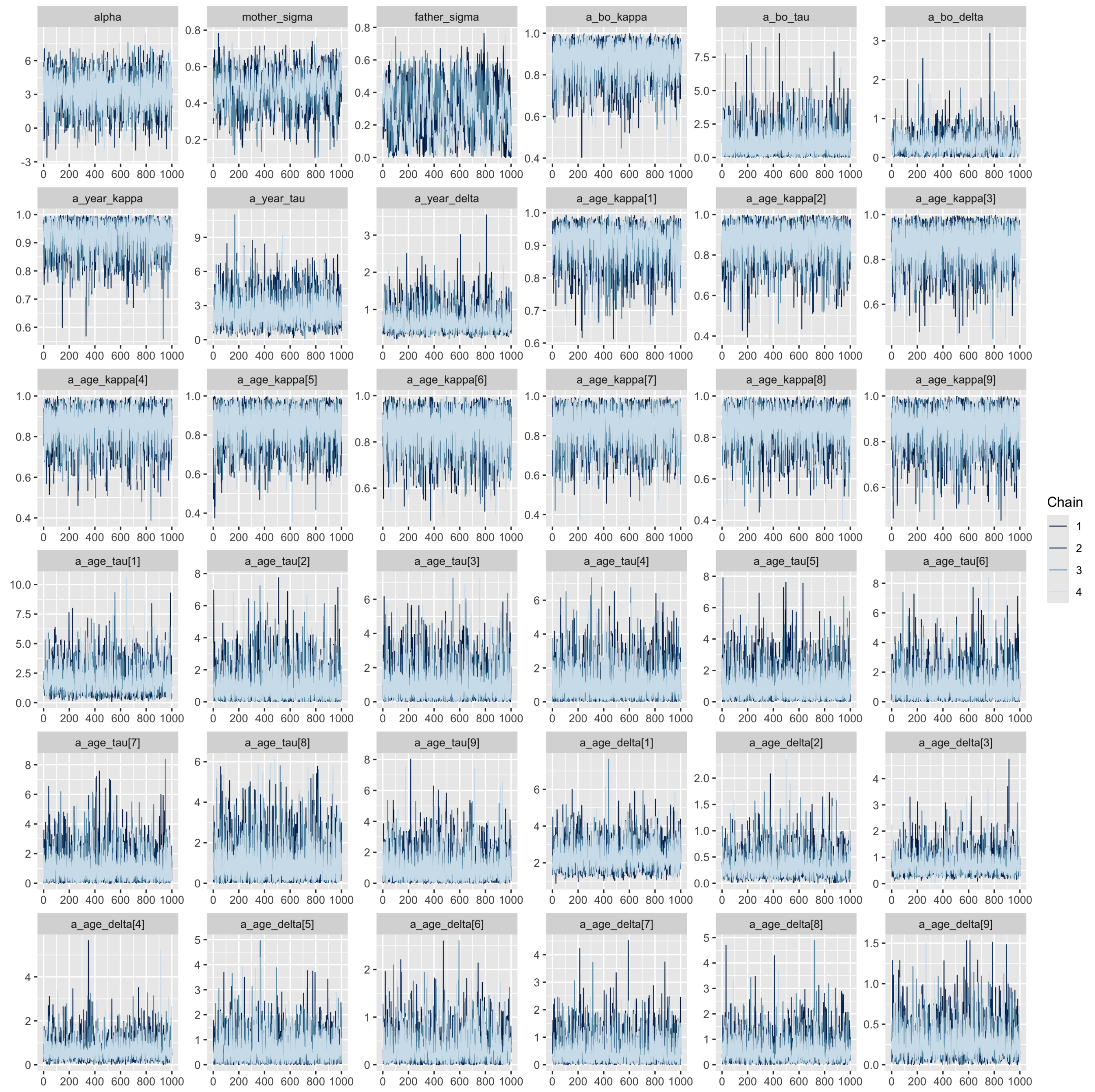

Fig. S25. Traceplots showing good mixing and convergence of four chains to the same posterior region for main model parameters—for the model of mother status at $t-1$ and child survival at t.

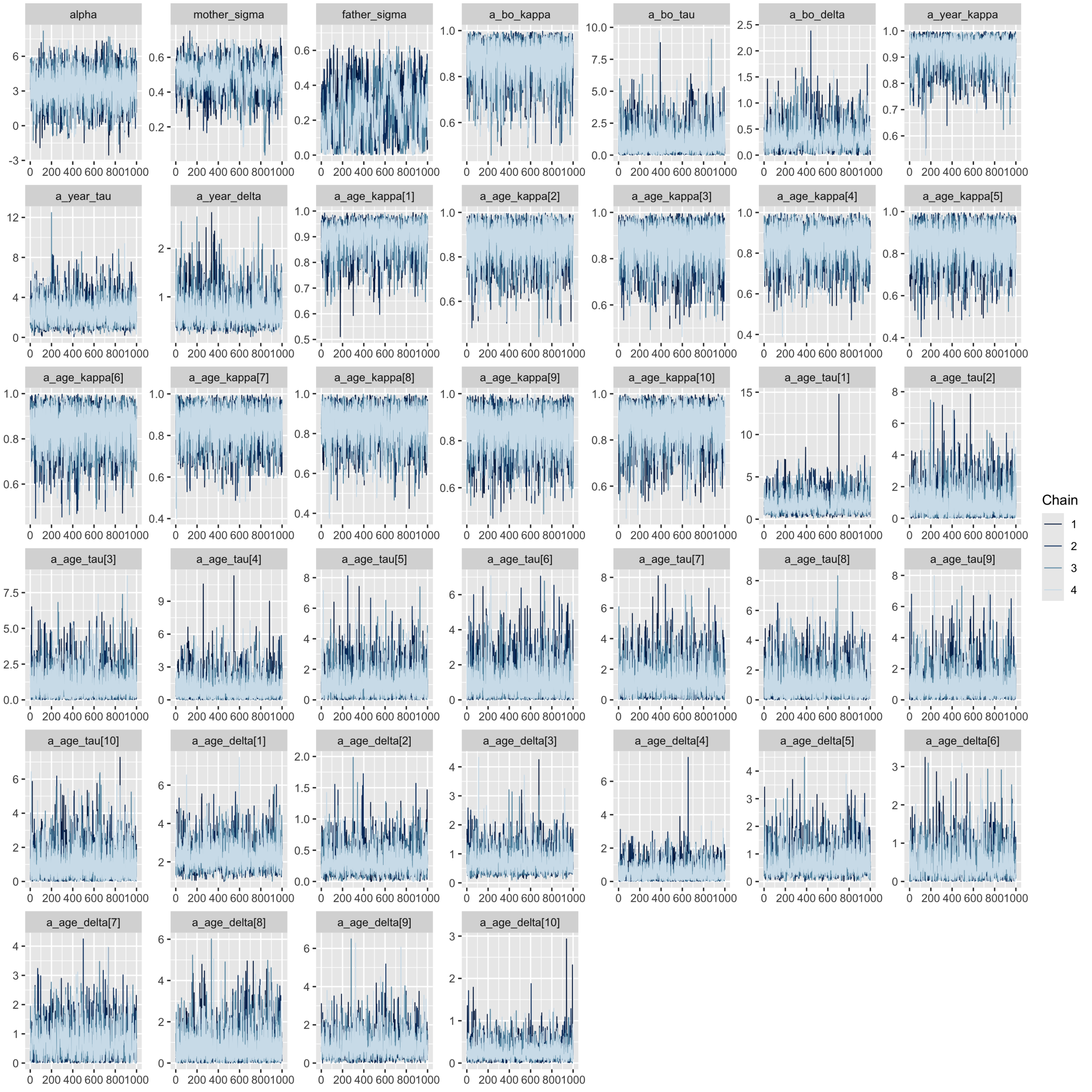

Fig. S26. Traceplots showing good mixing and convergence of four chains to the same posterior region for main model parameters—for the model of father status at $t-1$ and child survival at t.

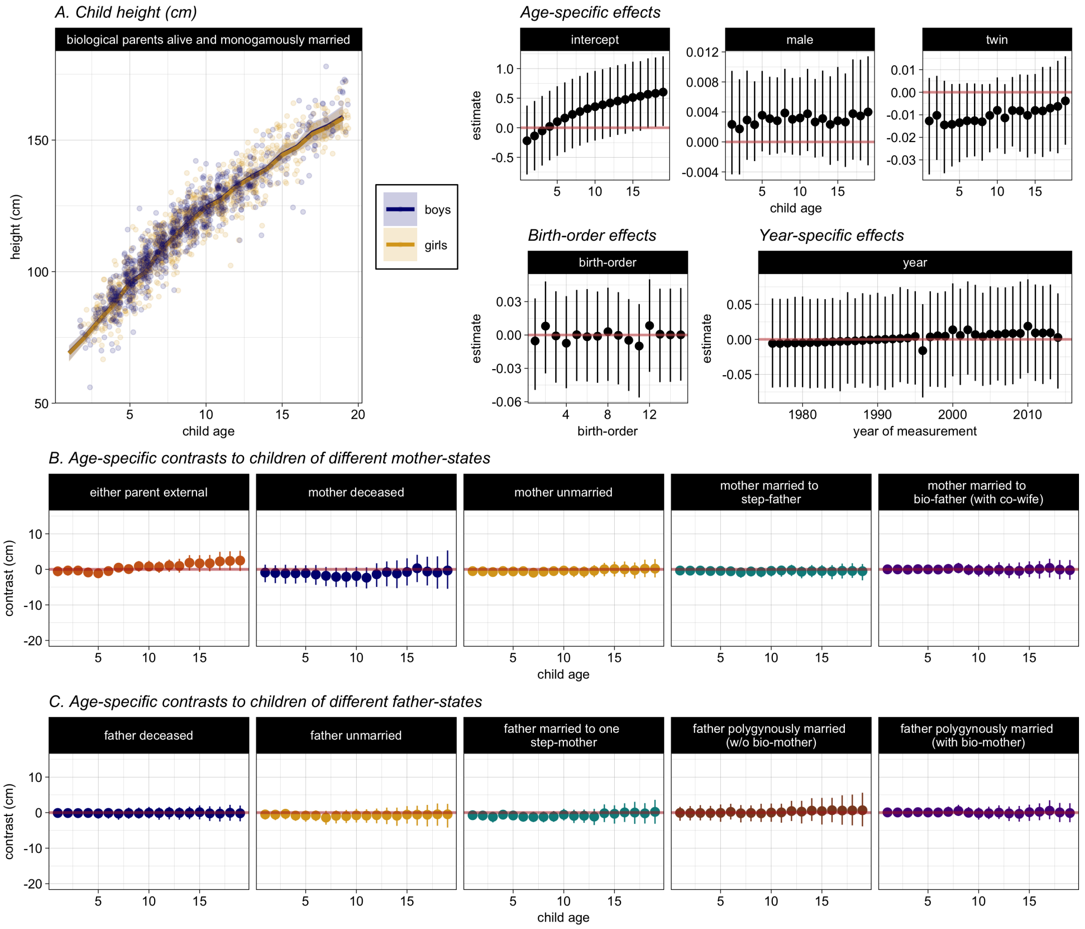

Fig. S27. Association between parent marital/vital states in year $t-1$ and child height in year t.

	mean	sd	5.5%	94.5%	rhat	ess_bulk
α	4.46	0.38	3.80	4.98	1.00	1533.18
γ_τ	0.69	0.78	0.03	2.19	1.00	3706.74
γ_κ	0.87	0.09	0.70	0.97	1.00	2533.55
γ_δ	0.03	0.02	0.01	0.05	1.00	1526.01
ϵ_τ	0.86	0.89	0.05	2.52	1.00	2369.15
ϵ_κ	0.88	0.08	0.72	0.98	1.00	2605.62
ϵ_δ	0.04	0.03	0.02	0.09	1.00	1680.49
β_{τ_1}	2.25	0.90	1.03	3.76	1.00	1336.83
β_{τ_2}	0.90	0.93	0.05	2.66	1.00	3549.72
β_{τ_3}	0.94	0.93	0.06	2.72	1.00	4844.55
β_{τ_4}	0.94	0.95	0.05	2.76	1.00	4363.24
β_{τ_5}	1.09	1.08	0.07	3.07	1.00	3765.29
β_{τ_6}	1.01	0.98	0.06	2.86	1.00	4468.72
β_{τ_7}	0.91	0.91	0.05	2.66	1.00	3713.25
β_{τ_8}	0.94	1.03	0.04	2.94	1.00	4395.16
β_{τ_9}	1.23	1.06	0.15	3.22	1.00	3222.30
β_{κ_1}	0.46	0.21	0.24	0.82	1.00	1484.49
β_{κ_2}	0.01	0.01	0.00	0.02	1.00	1562.83
β_{κ_3}	0.02	0.02	0.00	0.05	1.00	1767.62
β_{κ_4}	0.01	0.01	0.00	0.03	1.00	2370.78
β_{κ_5}	0.03	0.03	0.00	0.08	1.00	1283.38
β_{κ_6}	0.01	0.01	0.00	0.04	1.00	1518.44
β_{κ_7}	0.01	0.01	0.00	0.04	1.00	1554.02
β_{κ_8}	0.01	0.02	0.00	0.04	1.00	1651.02
β_{κ_9}	0.02	0.02	0.01	0.05	1.00	1365.77
β_{δ_1}	1.00	0.00	1.00	1.00	1.00	1126.73
β_{δ_2}	0.87	0.08	0.72	0.98	1.00	4940.39
β_{δ_3}	0.87	0.08	0.73	0.98	1.00	4635.88
β_{δ_4}	0.87	0.09	0.71	0.98	1.00	5726.73
β_{δ_5}	0.87	0.09	0.71	0.97	1.00	4519.81
β_{δ_6}	0.87	0.08	0.72	0.97	1.00	5027.47
β_{δ_7}	0.87	0.09	0.71	0.97	1.00	4739.53
β_{δ_8}	0.86	0.09	0.70	0.97	1.00	5101.41
β_{δ_9}	0.88	0.08	0.73	0.98	1.00	2962.63
κ_σ	0.03	0.00	0.02	0.03	1.00	748.44
η_σ	0.03	0.00	0.03	0.04	1.00	903.59
π_σ	0.06	0.00	0.05	0.06	1.00	1450.65

Table S17. Mean, standard deviation, 5.5% and 94.5% intervals, number of effective samples and Rhat values, for model parameters—modeling association between mother status at $t-1$ and child height at t.

	mean	sd	5.5%	94.5%	rhat	ess_bulk
α	4.45	0.41	3.76	4.96	1.00	1764.00
γ_τ	0.69	0.80	0.03	2.12	1.00	4002.45
γ_κ	0.86	0.09	0.70	0.97	1.00	3287.75
γ_δ	0.02	0.01	0.01	0.05	1.00	1462.55
ϵ_τ	0.85	0.90	0.04	2.53	1.00	2414.93
ϵ_κ	0.88	0.08	0.72	0.98	1.00	2759.54
ϵ_δ	0.04	0.02	0.02	0.08	1.00	2135.32
β_{τ_1}	2.22	0.91	0.99	3.84	1.00	1426.76
β_{τ_2}	0.88	0.91	0.05	2.69	1.00	4467.08
β_{τ_3}	0.92	0.91	0.06	2.64	1.00	4478.62
β_{τ_4}	1.02	1.03	0.05	2.94	1.00	3686.82
β_{τ_5}	0.93	0.93	0.05	2.65	1.00	4907.92
β_{τ_6}	0.98	1.00	0.05	2.77	1.00	4208.40
β_{τ_7}	1.03	1.01	0.07	2.91	1.00	4705.84
β_{τ_8}	0.97	0.96	0.05	2.80	1.00	4812.22
β_{τ_9}	0.92	0.94	0.05	2.77	1.00	5001.15
$\beta_{\tau_{10}}$	1.24	1.05	0.14	3.24	1.00	3576.69
β_{κ_1}	0.46	0.22	0.23	0.84	1.00	1596.47
β_{κ_2}	0.01	0.01	0.00	0.02	1.00	1648.06
β_{κ_3}	0.02	0.02	0.00	0.05	1.00	1777.52
β_{κ_4}	0.03	0.03	0.00	0.08	1.00	1677.32
β_{κ_5}	0.01	0.01	0.00	0.03	1.00	2040.38
β_{κ_6}	0.02	0.02	0.00	0.05	1.00	1552.31
β_{κ_7}	0.02	0.02	0.00	0.05	1.00	1208.21
β_{κ_8}	0.02	0.02	0.00	0.07	1.00	2102.48
β_{κ_9}	0.01	0.02	0.00	0.04	1.00	1541.58
$\beta_{\kappa_{10}}$	0.02	0.01	0.01	0.05	1.00	1583.20
β_{δ_1}	1.00	0.00	1.00	1.00	1.00	1309.67
β_{δ_2}	0.87	0.09	0.70	0.98	1.00	5093.90
β_{δ_3}	0.88	0.08	0.73	0.98	1.00	5551.90
β_{δ_4}	0.86	0.09	0.70	0.97	1.00	4996.77
β_{δ_5}	0.87	0.09	0.71	0.97	1.00	6744.32
β_{δ_6}	0.87	0.09	0.71	0.98	1.00	5057.20
β_{δ_7}	0.87	0.09	0.71	0.98	1.00	5402.72
β_{δ_8}	0.87	0.09	0.70	0.97	1.00	5622.31
β_{δ_9}	0.86	0.09	0.69	0.97	1.00	6436.22
$\beta_{\delta_{10}}$	0.88	0.08	0.73	0.98	1.00	3150.44
κ_σ	0.03	0.00	0.03	0.04	1.00	879.23
η_σ	0.03	0.00	0.02	0.03	1.01	690.07
π_σ	0.06	0.00	0.05	0.06	1.00	1397.81

Table S18. Mean, standard deviation, 5.5% and 94.5% intervals, number of effective samples and Rhat values, for model parameters—modeling association between father status at $t-1$ and child height at t.

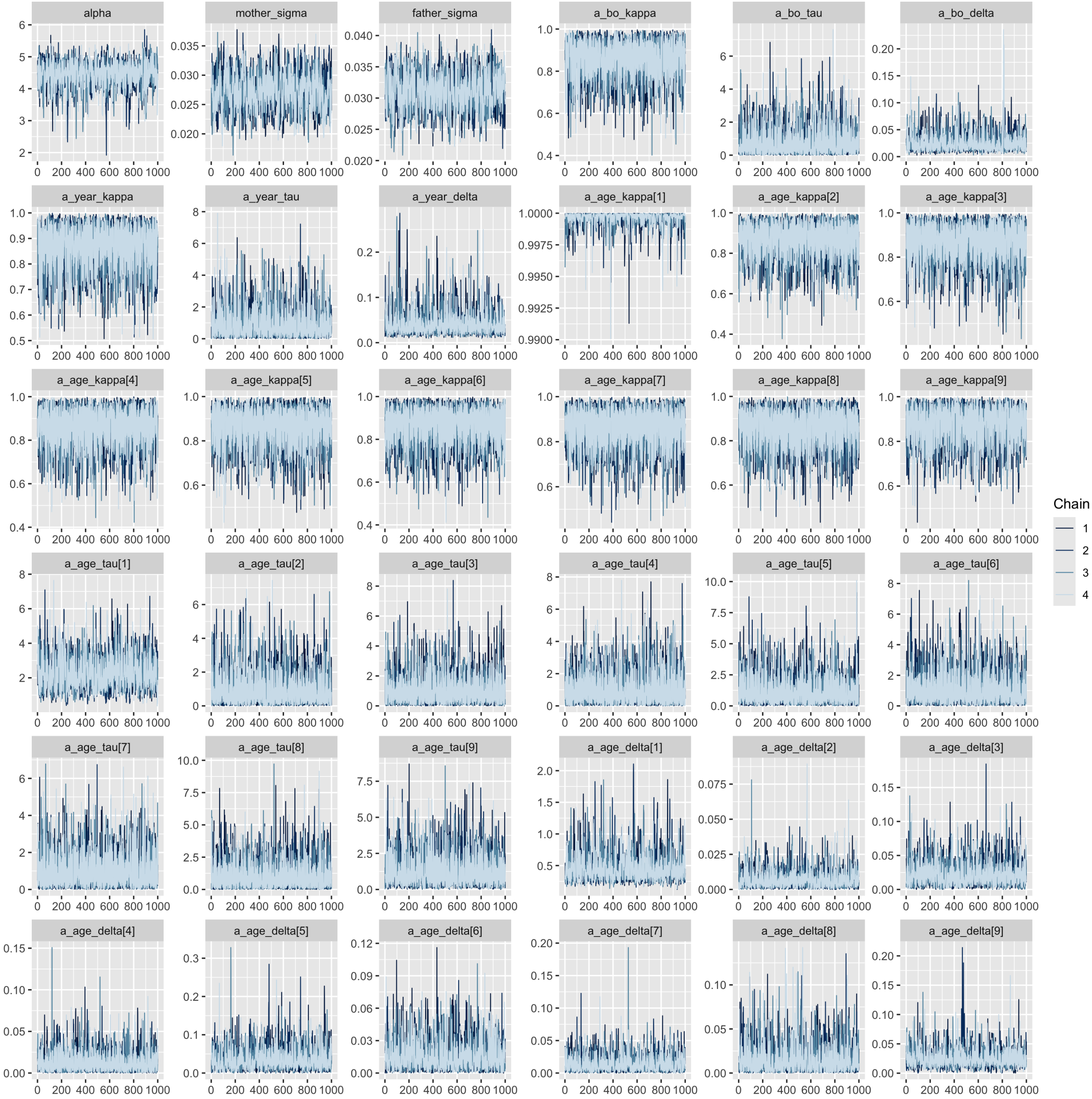

Fig. S28. Traceplots showing good mixing and convergence of four chains to the same posterior region for main model parameters—for the model of mother status at $t-1$ and child height at t.

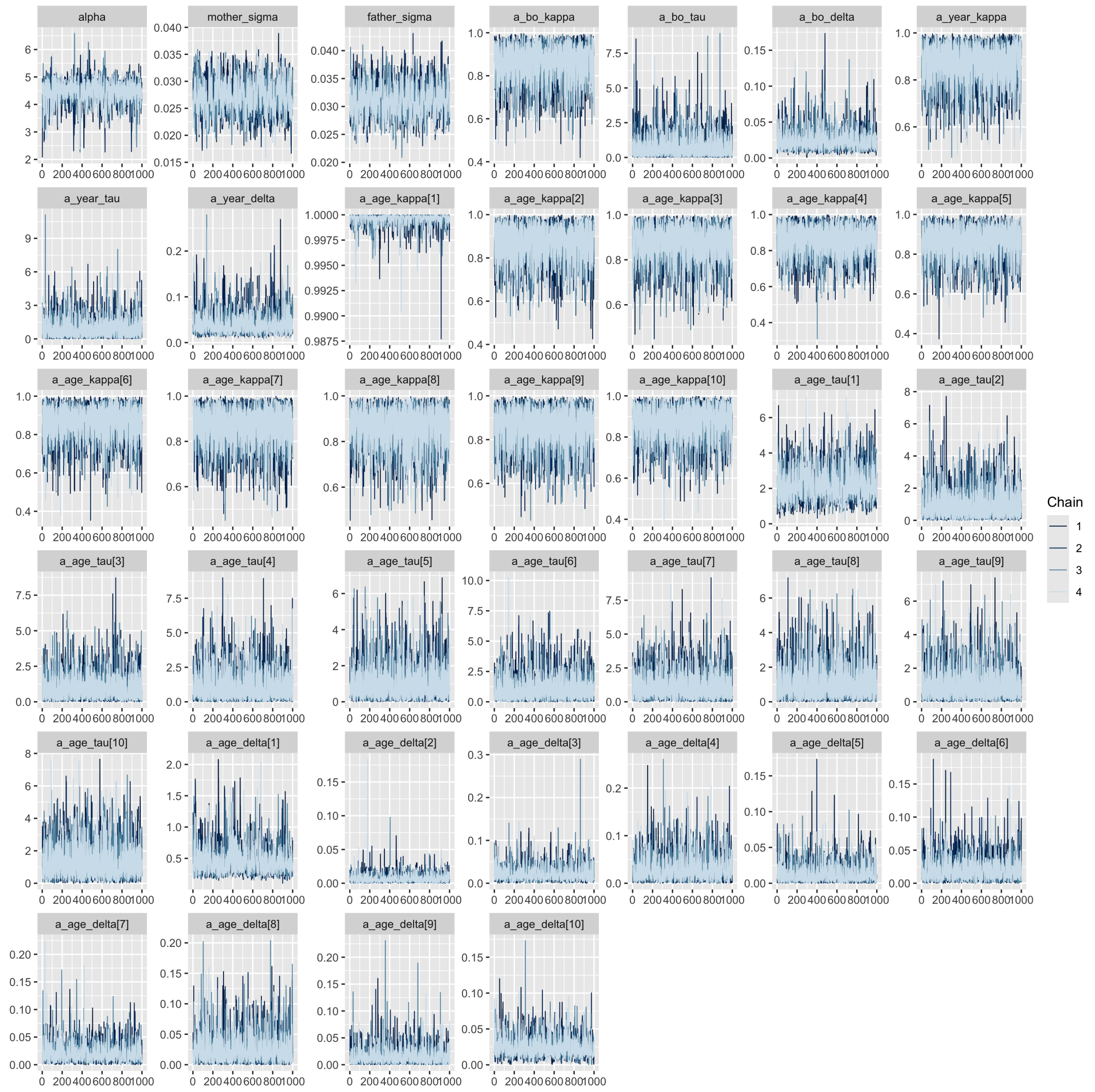

Fig. S29. Traceplots showing good mixing and convergence of four chains to the same posterior region for main model parameters—for the model of father status at $t-1$ and child height at t.

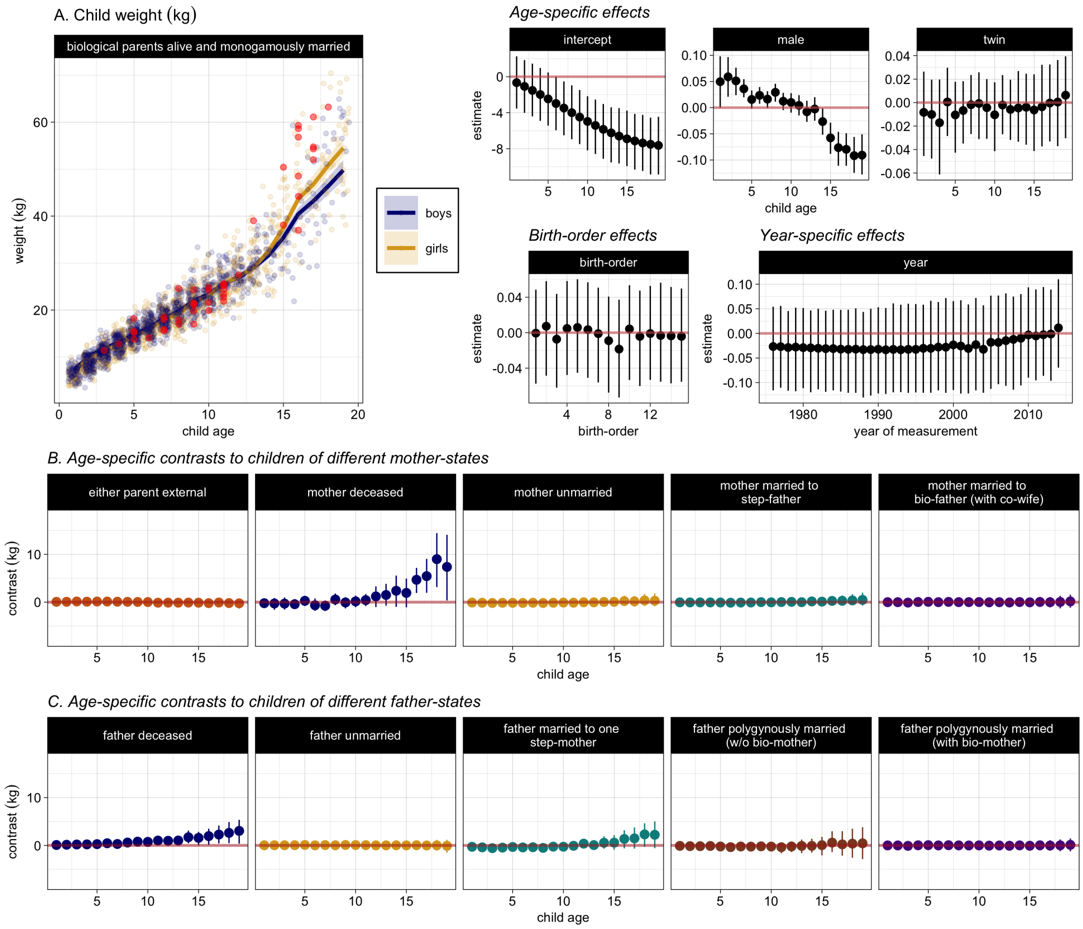

Fig. S30. Association between parent marital/vital states in year $t-1$ and child weight in year t.

	mean	sd	5.5%	94.5%	rhat	ess_bulk
α	-0.94	1.79	-3.67	2.10	1.01	337.45
γ_τ	0.79	0.81	0.05	2.33	1.00	875.98
γ_κ	0.87	0.08	0.71	0.97	1.01	711.74
γ_δ	0.03	0.02	0.01	0.07	1.01	408.33
ϵ_τ	1.47	1.18	0.16	3.68	1.01	491.84
ϵ_κ	0.92	0.07	0.79	0.99	1.01	563.05
ϵ_δ	0.05	0.04	0.02	0.12	1.01	537.73
β_{τ_1}	1.04	0.44	0.49	1.82	1.02	452.21
β_{τ_2}	1.97	1.44	0.43	4.72	1.01	449.60
β_{τ_3}	0.88	0.93	0.05	2.63	1.01	1045.11
β_{τ_4}	0.91	0.93	0.06	2.65	1.00	1030.84
β_{τ_5}	1.39	1.05	0.22	3.38	1.01	1000.91
β_{τ_6}	1.10	0.97	0.11	2.99	1.00	1172.03
β_{τ_7}	1.09	1.06	0.08	3.03	1.00	918.29
β_{τ_8}	0.90	0.96	0.04	2.79	1.00	813.05
β_{τ_9}	1.25	1.15	0.10	3.42	1.00	859.59
$\beta_{\tau_1 0}$	0.67	0.31	0.26	1.23	1.00	394.33
β_{κ_1}	2.98	0.89	1.77	4.54	1.01	383.37
β_{κ_2}	0.09	0.04	0.04	0.16	1.01	411.67
β_{κ_3}	0.04	0.04	0.00	0.11	1.00	461.04
β_{κ_4}	0.05	0.03	0.02	0.12	1.01	636.92
β_{κ_5}	0.17	0.09	0.07	0.32	1.00	555.25
β_{κ_6}	0.02	0.02	0.00	0.06	1.01	375.43
β_{κ_7}	0.02	0.02	0.00	0.06	1.01	319.39
β_{κ_8}	0.02	0.02	0.00	0.07	1.01	477.85
β_{κ_9}	0.02	0.02	0.00	0.05	1.01	456.00
$\beta_{\kappa_1 0}$	1.11	0.35	0.70	1.77	1.01	283.23
β_{δ_1}	1.00	0.00	1.00	1.00	1.01	339.37
β_{δ_2}	0.94	0.05	0.84	0.99	1.00	417.72
β_{δ_3}	0.86	0.09	0.69	0.97	1.00	962.02
β_{δ_4}	0.89	0.08	0.74	0.98	1.00	1025.71
β_{δ_5}	0.89	0.08	0.75	0.98	1.00	734.17
β_{δ_6}	0.88	0.08	0.72	0.98	1.00	1110.50
β_{δ_7}	0.87	0.09	0.72	0.98	1.00	1053.39
β_{δ_8}	0.86	0.09	0.69	0.97	1.00	956.66
β_{δ_9}	0.87	0.09	0.71	0.98	1.01	972.65
$\beta_{\delta_1 0}$	1.00	0.00	1.00	1.00	1.01	368.10
κ_σ	0.04	0.00	0.03	0.04	1.00	285.37
η_σ	0.05	0.00	0.04	0.06	1.01	350.93
π_σ	0.09	0.01	0.08	0.09	1.00	523.77

Table S19. Mean, standard deviation, 5.5% and 94.5% intervals, number of effective samples and Rhat values, for model parameters—modeling association between mother status at $t-1$ and child weight at t.

	mean	sd	5.5%	94.5%	rhat	ess_bulk
α	-1.19	1.81	-3.94	1.89	1.01	387.41
γ_τ	0.71	0.78	0.03	2.19	1.00	781.88
γ_κ	0.87	0.08	0.71	0.97	1.00	530.56
γ_δ	0.03	0.02	0.01	0.07	1.02	294.88
ϵ_τ	1.53	1.26	0.19	3.93	1.01	492.88
ϵ_κ	0.92	0.07	0.79	0.99	1.01	539.87
ϵ_δ	0.05	0.03	0.02	0.10	1.01	425.52
β_{τ_1}	1.15	0.49	0.52	2.01	1.02	301.31
β_{τ_2}	2.12	1.55	0.41	5.10	1.00	362.11
β_{τ_3}	0.93	0.96	0.04	2.73	1.00	949.78
β_{τ_4}	1.21	1.04	0.14	3.21	1.00	771.45
β_{τ_5}	0.94	0.89	0.06	2.60	1.00	559.48
β_{τ_6}	0.93	0.94	0.04	2.71	1.00	605.97
β_{τ_7}	1.30	1.02	0.22	3.14	1.00	763.42
β_{τ_8}	1.07	1.04	0.07	3.03	1.00	955.41
β_{τ_9}	0.96	0.97	0.06	2.73	1.01	1087.10
$\beta_{\tau_{10}}$	1.25	1.15	0.10	3.48	1.01	873.31
$\beta_{\tau_{11}}$	0.72	0.35	0.29	1.35	1.01	367.44
β_{κ_1}	2.89	0.87	1.76	4.45	1.01	412.75
β_{κ_2}	0.09	0.04	0.04	0.16	1.01	459.48
β_{κ_3}	0.03	0.04	0.00	0.09	1.00	527.59
β_{κ_4}	0.14	0.09	0.03	0.29	1.01	251.21
β_{κ_5}	0.05	0.03	0.02	0.11	1.02	408.27
β_{κ_6}	0.02	0.02	0.00	0.06	1.01	714.45
β_{κ_7}	0.07	0.04	0.02	0.14	1.01	382.42
β_{κ_8}	0.05	0.05	0.00	0.15	1.00	414.40
β_{κ_9}	0.02	0.02	0.00	0.07	1.01	522.25
$\beta_{\kappa_{10}}$	0.02	0.02	0.00	0.05	1.00	493.44
$\beta_{\kappa_{11}}$	1.08	0.33	0.68	1.66	1.01	293.30
β_{δ_1}	1.00	0.00	1.00	1.00	1.00	404.28
β_{δ_2}	0.94	0.05	0.85	0.99	1.01	463.42
β_{δ_3}	0.86	0.09	0.70	0.97	1.00	898.32
β_{δ_4}	0.88	0.08	0.73	0.98	1.00	651.73
β_{δ_5}	0.89	0.08	0.75	0.98	1.01	869.85
β_{δ_6}	0.87	0.09	0.70	0.98	1.00	1078.60
β_{δ_7}	0.91	0.07	0.77	0.99	1.00	545.35
β_{δ_8}	0.86	0.09	0.70	0.97	1.00	877.78
β_{δ_9}	0.86	0.09	0.70	0.97	1.00	1163.16
$\beta_{\delta_{10}}$	0.88	0.08	0.72	0.98	1.01	864.04
$\beta_{\delta_{11}}$	1.00	0.00	1.00	1.00	1.02	207.62
κ_σ	0.05	0.00	0.04	0.06	1.01	231.37
η_σ	0.03	0.01	0.02	0.04	1.03	153.96
π_σ	0.08	0.01	0.08	0.09	1.01	367.69

Table S20. Mean, standard deviation, 5.5% and 94.5% intervals, number of effective samples and Rhat values, for model parameters—modeling association between father status at $t-1$ and child weight at t.

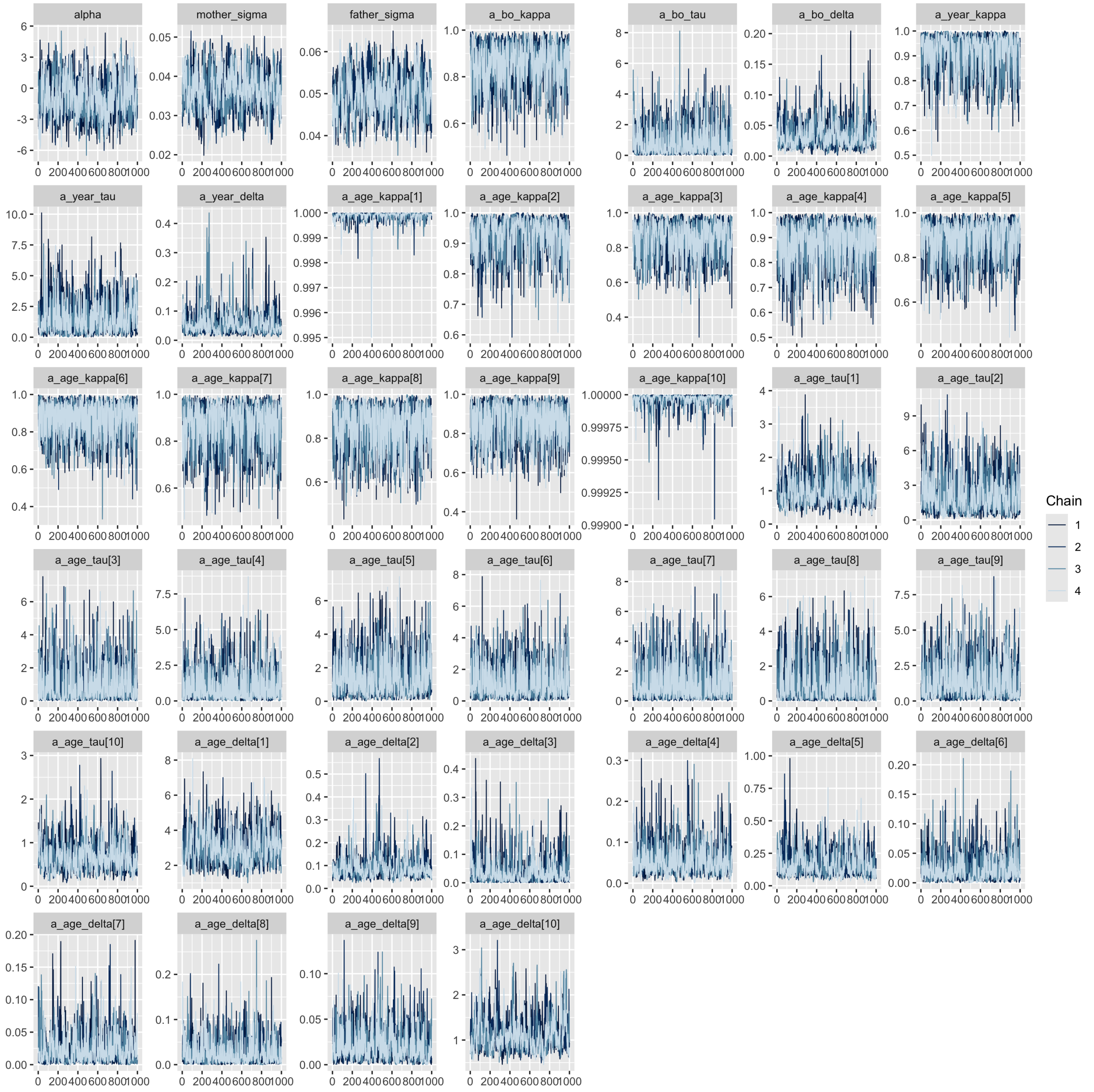

Fig. S31. Traceplots showing good mixing and convergence of four chains to the same posterior region for main model parameters—for the model of mother status at $t-1$ and child weight at t.

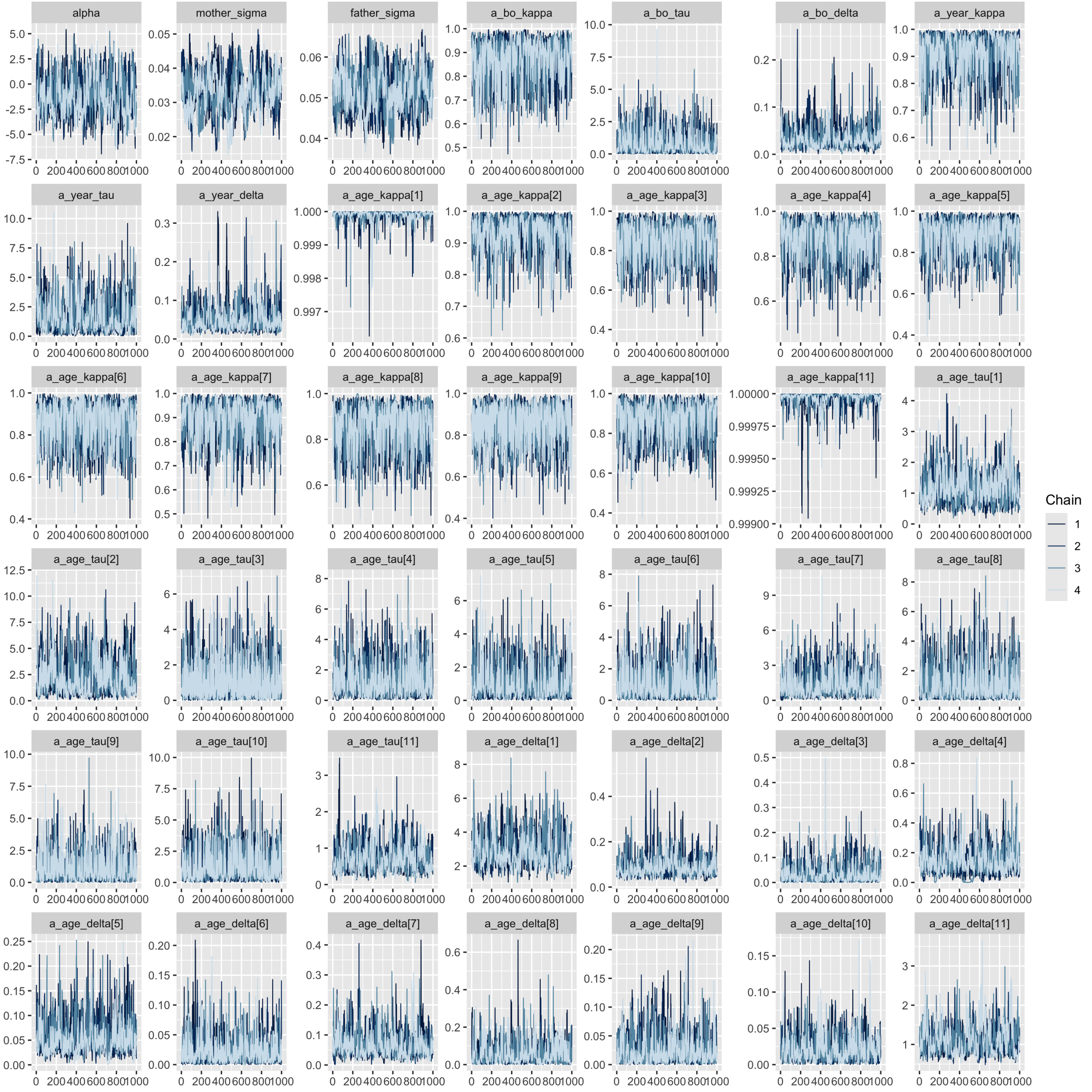

Fig. S32. Traceplots showing good mixing and convergence of four chains to the same posterior region for main model parameters—for the model of father status at $t-1$ and child weight at t.

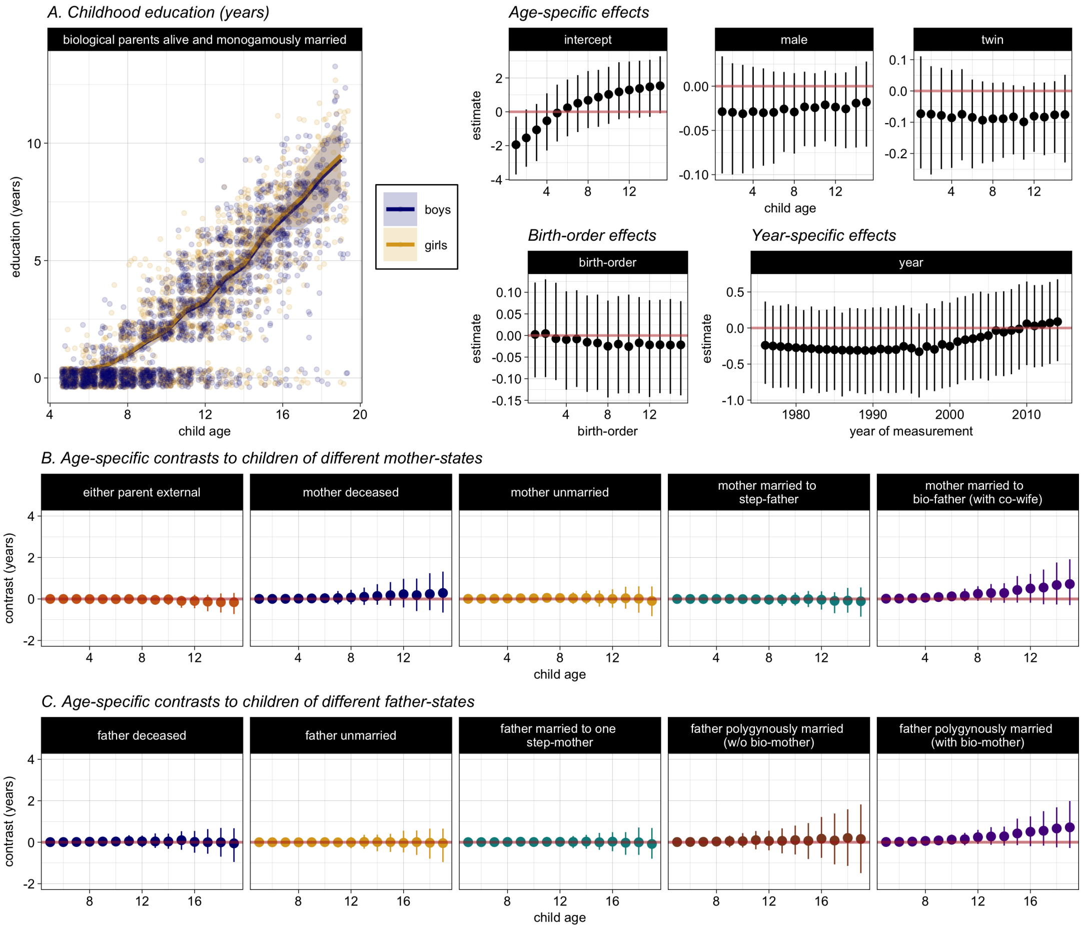

Fig. S33. Association between parent marital/vital states in year $t-1$ and child education in year t.

	mean	sd	5.5%	94.5%	rhat	ess_bulk
α	0.81	1.07	-0.92	2.48	1.00	2111.71
γ_{τ}	0.99	0.98	0.06	2.89	1.00	4586.31
γ_{κ}	0.89	0.08	0.74	0.98	1.00	3520.70
γ_{δ}	0.06	0.06	0.00	0.16	1.00	1619.62
ϵ_{τ}	1.95	1.40	0.31	4.53	1.00	1585.79
ϵ_{κ}	0.93	0.06	0.82	0.99	1.00	2011.50
ϵ_{δ}	0.38	0.20	0.17	0.73	1.00	2144.54
β_{τ_1}	2.81	1.31	1.05	5.14	1.01	787.34
β_{τ_2}	0.92	0.95	0.04	2.75	1.00	4616.65
β_{τ_3}	0.92	0.96	0.05	2.63	1.00	3928.33
β_{τ_4}	0.98	0.93	0.06	2.77	1.00	5043.32
β_{τ_5}	0.98	0.96	0.06	2.82	1.00	4556.91
β_{τ_6}	0.97	0.95	0.06	2.82	1.00	4065.41
β_{τ_7}	0.95	0.95	0.05	2.83	1.00	4193.17
β_{τ_8}	0.91	0.94	0.05	2.79	1.00	4379.94
β_{τ_9}	0.97	1.00	0.05	2.82	1.00	3773.26
β_{κ_1}	1.56	0.58	0.88	2.61	1.00	1805.35
β_{κ_2}	0.05	0.05	0.01	0.13	1.00	1733.84
β_{κ_3}	0.15	0.12	0.02	0.36	1.00	2171.58
β_{κ_4}	0.09	0.09	0.01	0.25	1.00	1859.32
β_{κ_5}	0.10	0.10	0.01	0.28	1.00	2490.73
β_{κ_6}	0.07	0.08	0.00	0.21	1.00	2158.60
β_{κ_7}	0.07	0.07	0.00	0.20	1.00	2825.72
β_{κ_8}	0.12	0.09	0.02	0.28	1.00	1978.30
β_{κ_9}	0.05	0.06	0.00	0.15	1.00	2004.49
β_{δ_1}	0.99	0.01	0.98	1.00	1.01	473.74
β_{δ_2}	0.88	0.08	0.73	0.98	1.00	5282.12
β_{δ_3}	0.88	0.08	0.72	0.98	1.00	5106.43
β_{δ_4}	0.87	0.09	0.70	0.97	1.00	5791.71
β_{δ_5}	0.87	0.09	0.70	0.97	1.00	5650.27
β_{δ_6}	0.87	0.09	0.70	0.97	1.00	5341.16
β_{δ_7}	0.87	0.09	0.70	0.97	1.00	5606.10
β_{δ_8}	0.87	0.08	0.71	0.97	1.00	5036.88
β_{δ_9}	0.87	0.09	0.71	0.97	1.00	6456.60
κ_{σ}	0.03	0.02	0.00	0.06	1.00	564.29
η_{σ}	0.03	0.02	0.00	0.07	1.00	750.31

Table S21. Mean, standard deviation, 5.5% and 94.5% intervals, number of effective samples and Rhat values, for model parameters—modeling association between mother status at $t-1$ and child education at t: parameters for the model of θ, i.e., modeling the probability a child does not go to school.

	mean	sd	5.5%	94.5%	rhat	ess_bulk
α	1.66	1.82	-1.19	4.62	1.00	4862.47
γ_τ	1.66	1.30	0.19	4.02	1.00	2745.47
γ_κ	0.89	0.08	0.73	0.98	1.00	2544.80
γ_δ	0.89	0.39	0.41	1.61	1.00	2330.45
ϵ_τ	2.88	1.62	0.88	5.92	1.00	1548.88
ϵ_κ	0.92	0.06	0.81	0.98	1.00	1793.02
ϵ_δ	2.83	0.86	1.75	4.39	1.00	2449.33
β_{τ_1}	2.06	0.86	1.01	3.58	1.00	1551.81
β_{τ_2}	1.06	1.06	0.06	3.03	1.00	4214.33
β_{τ_3}	0.95	0.97	0.06	2.71	1.00	4329.89
β_{τ_4}	1.00	0.98	0.06	2.86	1.00	4237.61
β_{τ_5}	1.03	0.97	0.07	2.90	1.00	3282.72
β_{τ_6}	1.02	1.05	0.05	3.04	1.00	2913.65
β_{τ_7}	1.00	0.96	0.06	2.81	1.00	4114.59
β_{τ_8}	0.79	0.82	0.04	2.43	1.00	4747.43
β_{τ_9}	1.43	1.15	0.16	3.62	1.00	2623.38
β_{κ_1}	4.26	1.04	2.83	6.08	1.00	2051.75
β_{κ_2}	0.32	0.28	0.02	0.85	1.00	1673.14
β_{κ_3}	0.66	0.60	0.04	1.86	1.00	1571.81
β_{κ_4}	0.73	0.66	0.05	1.94	1.00	1923.20
β_{κ_5}	1.06	0.85	0.08	2.65	1.00	1570.63
β_{κ_6}	0.42	0.41	0.03	1.18	1.00	2645.10
β_{κ_7}	0.90	0.56	0.19	1.89	1.00	1550.02
β_{κ_8}	1.40	0.62	0.64	2.50	1.00	2598.47
β_{κ_9}	1.07	0.57	0.29	2.07	1.00	1358.35
β_{δ_1}	0.99	0.01	0.97	1.00	1.00	1134.36
β_{δ_2}	0.86	0.09	0.70	0.97	1.00	4952.02
β_{δ_3}	0.85	0.09	0.69	0.97	1.00	4454.90
β_{δ_4}	0.85	0.09	0.68	0.97	1.00	5051.49
β_{δ_5}	0.85	0.10	0.67	0.97	1.00	5634.35
β_{δ_6}	0.86	0.09	0.69	0.97	1.00	5396.51
β_{δ_7}	0.87	0.08	0.71	0.97	1.00	4126.81
β_{δ_8}	0.89	0.07	0.75	0.98	1.00	4410.36
β_{δ_9}	0.89	0.08	0.74	0.98	1.00	3670.67
κ_σ	1.12	0.25	0.71	1.51	1.00	404.66
η_σ	1.70	0.18	1.43	1.99	1.00	813.61

Table S22. Mean, standard deviation, 5.5% and 94.5% intervals, number of effective samples and Rhat values, for model parameters—modeling association between mother status at $t-1$ and child education at t: parameters for the model of η, i.e., modeling years of schooling, conditional on attending school.

	mean	sd	5.5%	94.5%	rhat	ess_bulk
α	0.75	1.06	-0.96	2.49	1.00	3113.66
γ_τ	1.02	1.01	0.06	2.95	1.00	4548.80
γ_κ	0.89	0.08	0.73	0.98	1.00	5380.67
γ_δ	0.06	0.05	0.01	0.15	1.00	1683.42
ϵ_τ	2.02	1.40	0.34	4.59	1.00	2270.85
ϵ_κ	0.94	0.06	0.82	0.99	1.00	2764.25
ϵ_δ	0.37	0.19	0.17	0.72	1.00	3371.78
β_{τ_1}	2.74	1.34	1.02	5.13	1.00	964.40
β_{τ_2}	0.94	0.92	0.06	2.71	1.00	5355.41
β_{τ_3}	0.91	0.90	0.05	2.61	1.00	3928.99
β_{τ_4}	0.96	0.97	0.05	2.80	1.00	4565.91
β_{τ_5}	0.96	0.95	0.05	2.81	1.00	3972.13
β_{τ_6}	0.99	1.00	0.06	2.88	1.00	5110.12
β_{τ_7}	0.98	1.00	0.06	2.88	1.00	5351.46
β_{τ_8}	0.98	1.02	0.05	2.84	1.00	4581.06
β_{τ_9}	0.90	0.93	0.05	2.62	1.00	4376.72
β_{τ_10}	0.95	0.98	0.04	2.77	1.00	4297.40
β_{κ_1}	1.55	0.55	0.89	2.56	1.00	2484.67
β_{κ_2}	0.05	0.05	0.01	0.14	1.00	2173.36
β_{κ_3}	0.15	0.11	0.02	0.35	1.00	2393.02
β_{κ_4}	0.10	0.10	0.01	0.28	1.00	2843.02
β_{κ_5}	0.09	0.09	0.01	0.25	1.00	2266.54
β_{κ_6}	0.08	0.09	0.01	0.24	1.00	2787.40
β_{κ_7}	0.07	0.07	0.01	0.21	1.00	2904.92
β_{κ_8}	0.15	0.15	0.01	0.43	1.00	2633.97
β_{κ_9}	0.12	0.09	0.02	0.29	1.00	2199.42
β_{κ_10}	0.05	0.05	0.00	0.14	1.00	2267.20
β_{δ_1}	0.99	0.01	0.98	1.00	1.00	625.74
β_{δ_2}	0.87	0.08	0.72	0.98	1.00	5553.60
β_{δ_3}	0.87	0.08	0.72	0.97	1.00	5433.02
β_{δ_4}	0.87	0.09	0.70	0.98	1.00	5915.73
β_{δ_5}	0.87	0.09	0.70	0.97	1.00	6886.99
β_{δ_6}	0.87	0.09	0.70	0.97	1.00	6240.46
β_{δ_7}	0.86	0.09	0.70	0.97	1.00	7673.63
β_{δ_8}	0.86	0.09	0.71	0.97	1.00	8260.31
β_{δ_9}	0.87	0.08	0.72	0.98	1.00	6683.92
β_{δ_10}	0.87	0.09	0.70	0.98	1.00	5630.74
κ_σ	0.03	0.02	0.00	0.07	1.00	903.61
η_σ	0.03	0.02	0.00	0.07	1.00	1024.94

Table S23. Mean, standard deviation, 5.5% and 94.5% intervals, number of effective samples and Rhat values, for model parameters—modeling association between father status at $t-1$ and child education at t: parameters for the model of θ, i.e., modeling the probability a child does not go to school.

	mean	sd	5.5%	94.5%	rhat	ess_bulk
α	1.66	1.77	-1.19	4.49	1.00	6045.31
γ_τ	1.65	1.25	0.19	4.00	1.00	3021.54
γ_κ	0.89	0.08	0.74	0.98	1.00	2968.55
γ_δ	0.89	0.39	0.42	1.58	1.00	2617.53
ϵ_τ	2.94	1.63	0.95	5.98	1.00	2699.78
ϵ_κ	0.92	0.06	0.81	0.98	1.00	2582.17
ϵ_δ	2.84	0.86	1.73	4.42	1.00	3529.48
β_{τ_1}	2.10	0.88	1.01	3.71	1.00	3110.77
β_{τ_2}	1.04	1.04	0.07	3.01	1.00	4510.82
β_{τ_3}	0.97	1.00	0.06	2.85	1.00	4748.14
β_{τ_4}	1.03	0.98	0.06	2.90	1.00	4534.57
β_{τ_5}	1.01	0.98	0.07	2.83	1.00	4599.25
β_{τ_6}	0.99	0.99	0.06	2.89	1.00	4584.17
β_{τ_7}	1.09	1.11	0.06	3.21	1.00	4649.83
β_{τ_8}	0.95	0.95	0.05	2.79	1.00	4285.14
β_{τ_9}	0.79	0.81	0.04	2.24	1.00	4155.85
$\beta_{\tau_1 0}$	1.44	1.17	0.14	3.65	1.00	2612.54
β_{κ_1}	4.23	1.05	2.81	6.12	1.00	3461.69
β_{κ_2}	0.31	0.27	0.02	0.81	1.00	2118.00
β_{κ_3}	0.64	0.61	0.05	1.78	1.00	2416.24
β_{κ_4}	0.99	0.83	0.08	2.52	1.00	2150.67
β_{κ_5}	0.79	0.68	0.05	2.07	1.00	1625.51
β_{κ_6}	0.75	0.70	0.04	2.12	1.00	1397.98
β_{κ_7}	0.87	0.74	0.06	2.25	1.00	1315.17
β_{κ_8}	1.05	0.79	0.10	2.48	1.00	2299.91
β_{κ_9}	1.39	0.62	0.62	2.45	1.00	3485.61
$\beta_{\kappa_1 0}$	1.02	0.57	0.21	2.00	1.00	965.31
β_{δ_1}	0.99	0.01	0.97	1.00	1.00	1475.90
β_{δ_2}	0.86	0.09	0.70	0.97	1.00	6536.20
β_{δ_3}	0.86	0.09	0.69	0.97	1.00	5814.42
β_{δ_4}	0.85	0.09	0.68	0.97	1.00	5765.20
β_{δ_5}	0.86	0.09	0.69	0.97	1.00	5477.91
β_{δ_6}	0.84	0.10	0.66	0.97	1.00	5976.77
β_{δ_7}	0.84	0.09	0.67	0.96	1.00	5345.93
β_{δ_8}	0.86	0.09	0.70	0.97	1.00	7040.91
β_{δ_9}	0.89	0.07	0.75	0.98	1.00	5009.68
$\beta_{\delta_1 0}$	0.89	0.08	0.74	0.98	1.00	4621.49
κ_σ	1.08	0.28	0.62	1.51	1.01	276.26
η_σ	1.73	0.18	1.45	2.03	1.00	822.38

Table S24. Mean, standard deviation, 5.5% and 94.5% intervals, number of effective samples and Rhat values, for model parameters—modeling association between father status at $t-1$ and child education at t: parameters for the model of η, i.e., modeling years of schooling, conditional on attending school.

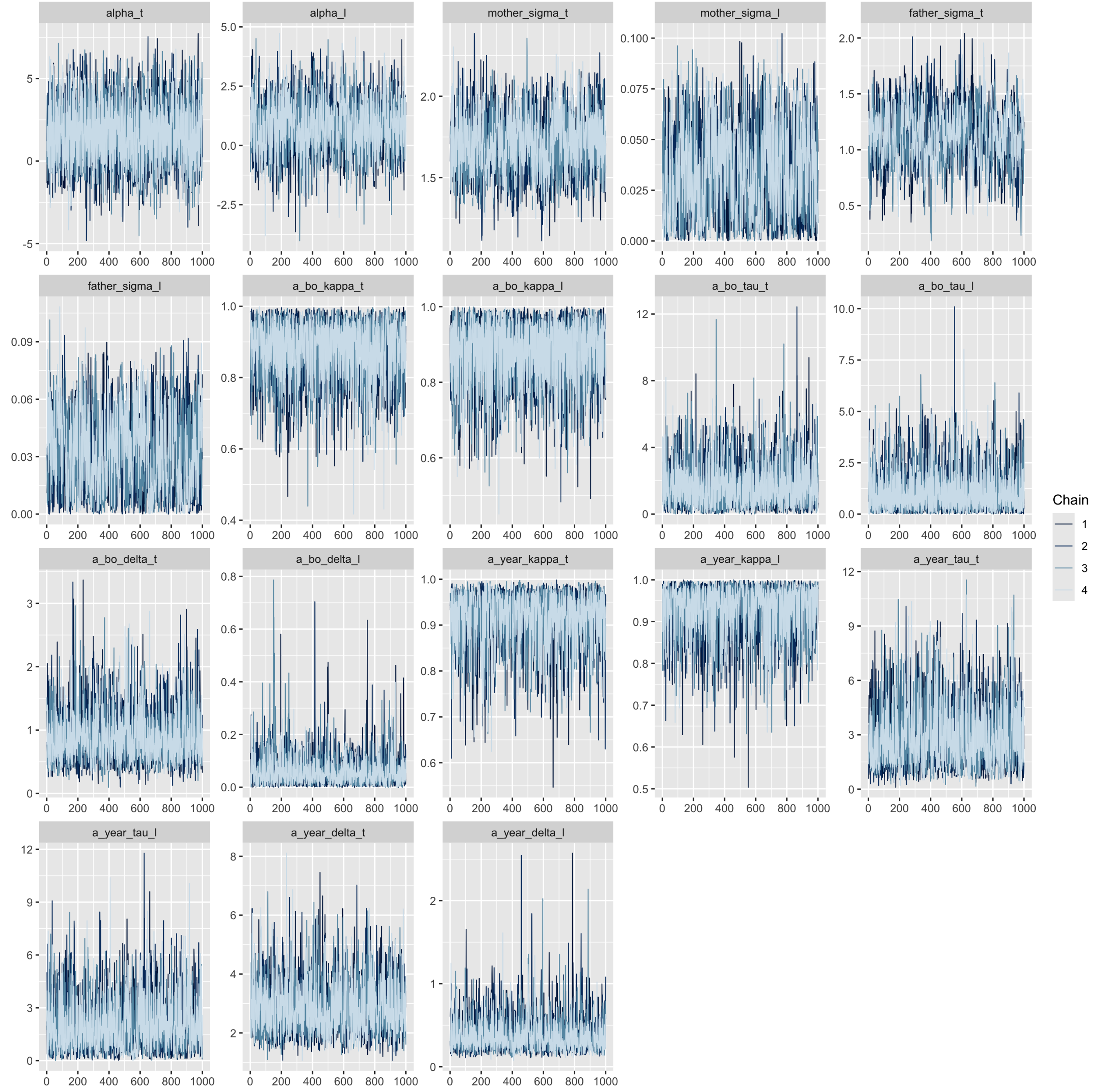

Fig. S34. Traceplots showing good mixing and convergence of four chains to the same posterior region for main model parameters—for the model of mother status at $t-1$ and child education at t.

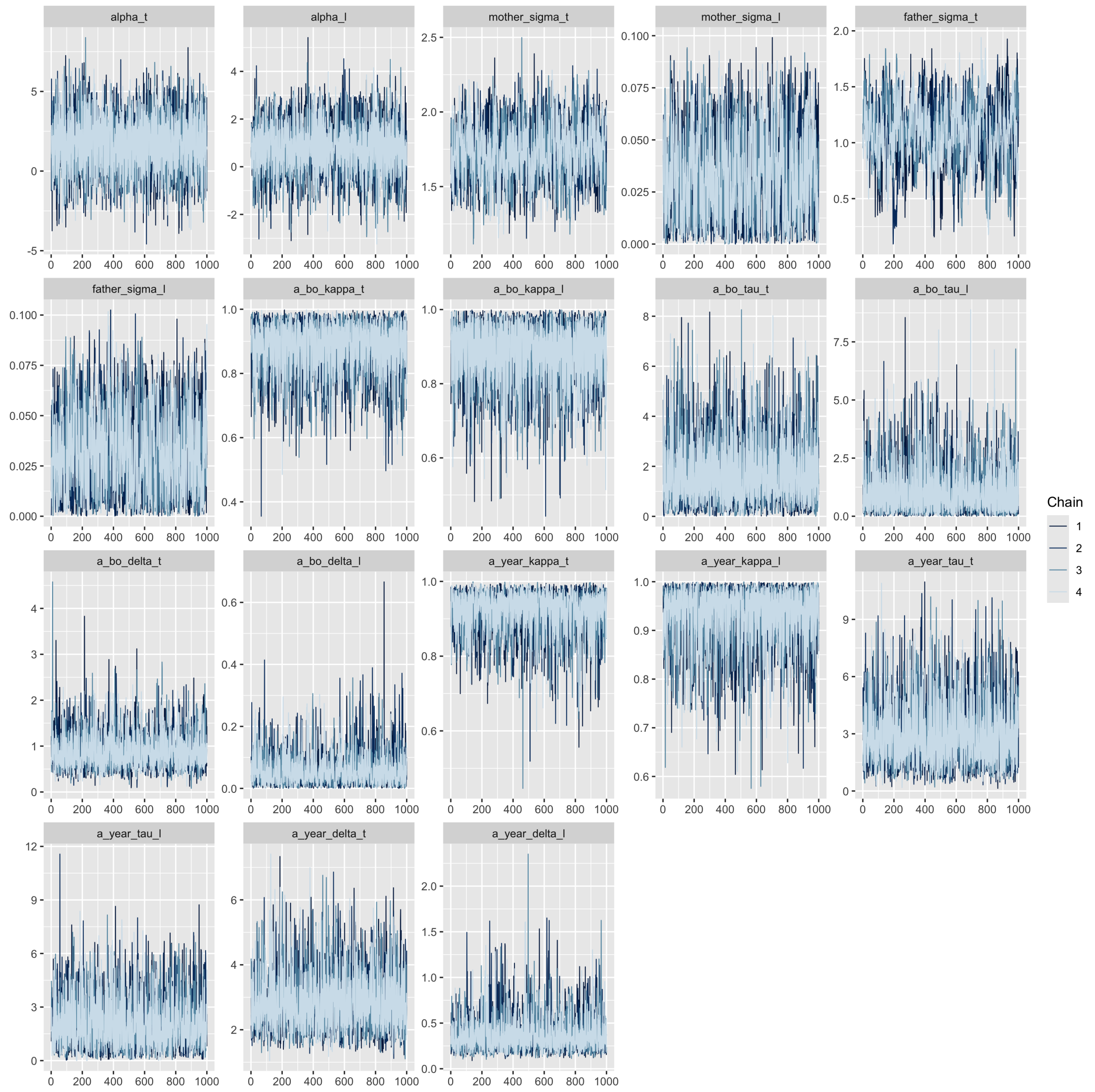

Fig. S35. Traceplots showing good mixing and convergence of four chains to the same posterior region for main model parameters—for the model of father status at $t-1$ and child education at t.

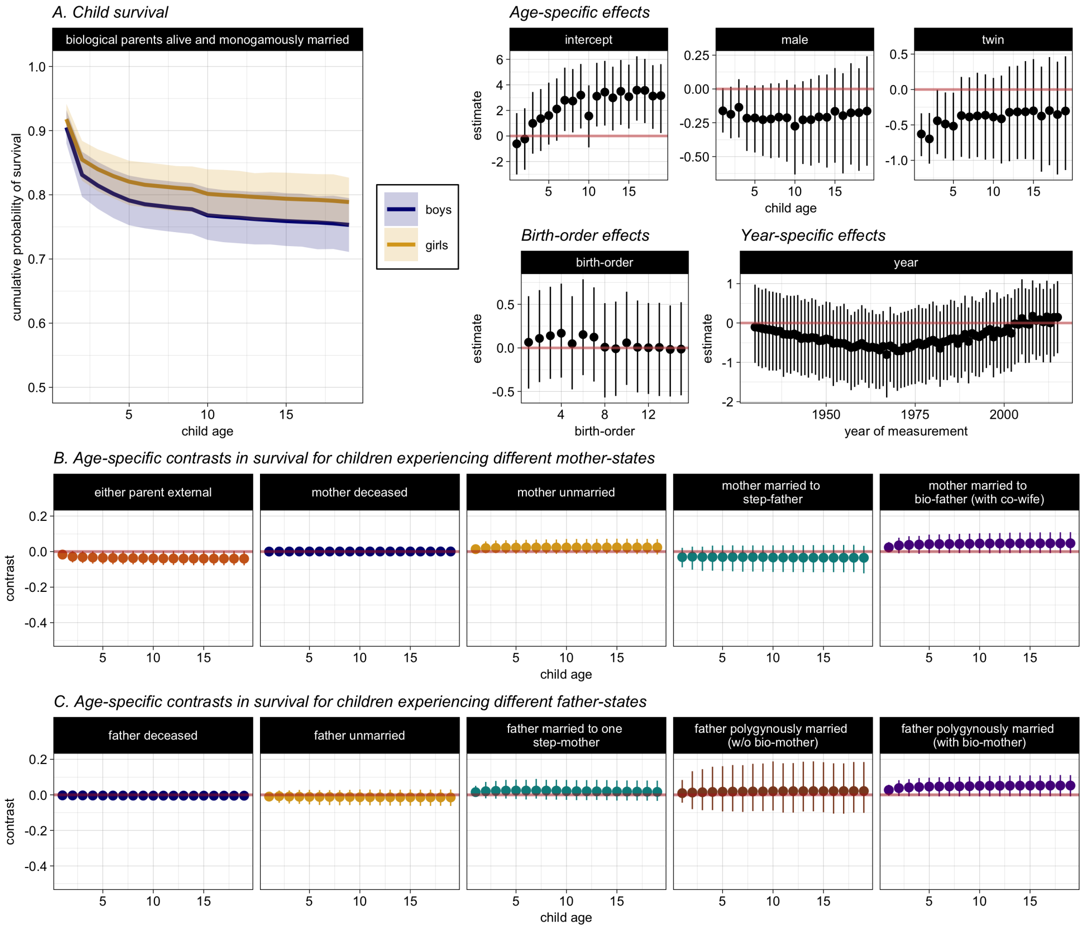

Fig. S36. Association between parent marital/vital states in year $t + 1$ and child survival in year t.

	mean	sd	5.5%	94.5%	rhat	ess_bulk
α	3.27	1.49	0.80	5.49	1.00	2464.94
γ_τ	0.97	1.00	0.05	2.91	1.00	3257.98
γ_κ	0.87	0.09	0.71	0.97	1.00	4680.39
γ_δ	0.30	0.22	0.03	0.69	1.00	1486.38
ϵ_τ	2.40	1.26	0.84	4.71	1.00	2959.37
ϵ_κ	0.92	0.06	0.82	0.99	1.00	2797.11
ϵ_δ	0.68	0.30	0.35	1.22	1.00	2730.90
β_{τ_1}	1.71	1.08	0.50	3.75	1.00	1701.21
β_{τ_2}	0.95	0.98	0.05	2.78	1.00	4555.27
β_{τ_3}	0.95	0.96	0.06	2.81	1.00	4791.03
β_{τ_4}	0.81	0.88	0.04	2.41	1.00	4591.64
β_{τ_5}	0.88	0.93	0.04	2.68	1.00	4603.13
β_{τ_6}	0.99	1.01	0.06	3.00	1.00	4998.33
β_{τ_7}	0.95	0.98	0.05	2.87	1.00	3938.22
β_{τ_8}	0.92	0.94	0.06	2.72	1.00	4339.97
β_{τ_9}	0.94	0.96	0.05	2.73	1.00	5509.40
β_{κ_1}	2.31	0.67	1.45	3.50	1.00	2108.62
β_{κ_2}	0.32	0.23	0.06	0.74	1.00	1542.56
β_{κ_3}	0.71	0.39	0.29	1.44	1.00	2121.39
β_{κ_4}	0.06	0.08	0.01	0.19	1.00	942.38
β_{κ_5}	0.02	0.02	0.00	0.06	1.00	1219.65
β_{κ_6}	0.36	0.34	0.03	0.99	1.00	1694.34
β_{κ_7}	0.62	0.58	0.03	1.70	1.00	1311.95
β_{κ_8}	0.53	0.43	0.05	1.33	1.00	1855.65
β_{κ_9}	0.26	0.18	0.05	0.60	1.00	2151.87
β_{δ_1}	0.90	0.06	0.79	0.97	1.00	1690.53
β_{δ_2}	0.87	0.08	0.72	0.98	1.00	5238.12
β_{δ_3}	0.87	0.08	0.72	0.97	1.00	5475.19
β_{δ_4}	0.85	0.09	0.68	0.97	1.00	4651.57
β_{δ_5}	0.86	0.09	0.70	0.97	1.00	5669.30
β_{δ_6}	0.86	0.09	0.70	0.97	1.00	6344.80
β_{δ_7}	0.84	0.10	0.67	0.96	1.00	5742.01
β_{δ_8}	0.86	0.09	0.69	0.97	1.00	5685.96
β_{δ_9}	0.88	0.08	0.73	0.98	1.00	5568.90
κ_σ	0.47	0.09	0.31	0.60	1.02	498.51
η_σ	0.27	0.14	0.04	0.50	1.03	275.00
π_σ	0.73	0.14	0.52	0.95	1.01	459.92

Table S25. Mean, standard deviation, 5.5% and 94.5% intervals, number of effective samples and Rhat values, for model parameters—modeling association between mother status at $t+1$ and child survival at t.

	mean	sd	5.5%	94.5%	rhat	ess_bulk
α	3.36	1.46	0.88	5.55	1.00	2140.61
γ_τ	0.96	0.95	0.05	2.72	1.00	3812.17
γ_κ	0.87	0.09	0.71	0.98	1.00	5102.91
γ_δ	0.29	0.22	0.03	0.70	1.00	1244.24
ϵ_τ	2.45	1.25	0.86	4.74	1.00	3197.26
ϵ_κ	0.93	0.06	0.82	0.99	1.00	2776.17
ϵ_δ	0.69	0.31	0.35	1.23	1.00	2321.99
β_{τ_1}	1.74	1.07	0.53	3.70	1.00	2035.99
β_{τ_2}	0.96	0.97	0.05	2.77	1.00	4141.18
β_{τ_3}	0.94	1.00	0.04	2.87	1.00	4860.00
β_{τ_4}	0.86	0.92	0.04	2.57	1.00	4957.52
β_{τ_5}	0.82	0.90	0.04	2.52	1.00	5399.93
β_{τ_6}	0.94	0.94	0.06	2.70	1.00	6062.57
β_{τ_7}	1.16	1.09	0.08	3.19	1.00	4682.91
β_{τ_8}	0.98	0.98	0.06	2.84	1.00	5696.33
β_{τ_9}	0.94	0.99	0.05	2.79	1.00	5384.14
$\beta_{\tau_{10}}$	0.91	0.94	0.04	2.70	1.00	4807.56
β_{κ_1}	2.29	0.69	1.42	3.51	1.01	1665.78
β_{κ_2}	0.31	0.22	0.06	0.72	1.00	1510.23
β_{κ_3}	0.72	0.39	0.29	1.45	1.00	2235.82
β_{κ_4}	0.02	0.02	0.00	0.06	1.00	1390.68
β_{κ_5}	0.06	0.07	0.01	0.17	1.00	1066.01
β_{κ_6}	0.38	0.38	0.02	1.10	1.00	1699.88
β_{κ_7}	0.53	0.48	0.04	1.45	1.00	1700.17
β_{κ_8}	0.73	0.73	0.05	2.16	1.00	3741.55
β_{κ_9}	0.62	0.51	0.06	1.55	1.00	1805.52
$\beta_{\kappa_{10}}$	0.28	0.19	0.07	0.63	1.00	2695.05
β_{δ_1}	0.90	0.06	0.78	0.97	1.01	1351.13
β_{δ_2}	0.87	0.08	0.71	0.98	1.00	7091.86
β_{δ_3}	0.87	0.08	0.71	0.97	1.00	5896.61
β_{δ_4}	0.86	0.09	0.70	0.97	1.00	5301.80
β_{δ_5}	0.85	0.09	0.68	0.96	1.00	5591.41
β_{δ_6}	0.85	0.09	0.69	0.97	1.00	5374.44
β_{δ_7}	0.86	0.09	0.70	0.97	1.00	6371.79
β_{δ_8}	0.86	0.09	0.69	0.97	1.00	7567.71
β_{δ_9}	0.86	0.09	0.70	0.97	1.00	6404.70
$\beta_{\delta_{10}}$	0.88	0.08	0.72	0.98	1.00	6411.21
κ_σ	0.28	0.15	0.04	0.51	1.02	259.89
η_σ	0.47	0.09	0.32	0.60	1.01	543.26
π_σ	0.75	0.13	0.53	0.96	1.01	408.39

Table S26. Mean, standard deviation, 5.5% and 94.5% intervals, number of effective samples and Rhat values, for model parameters—modeling association between father status at $t+1$ and child survival at t.

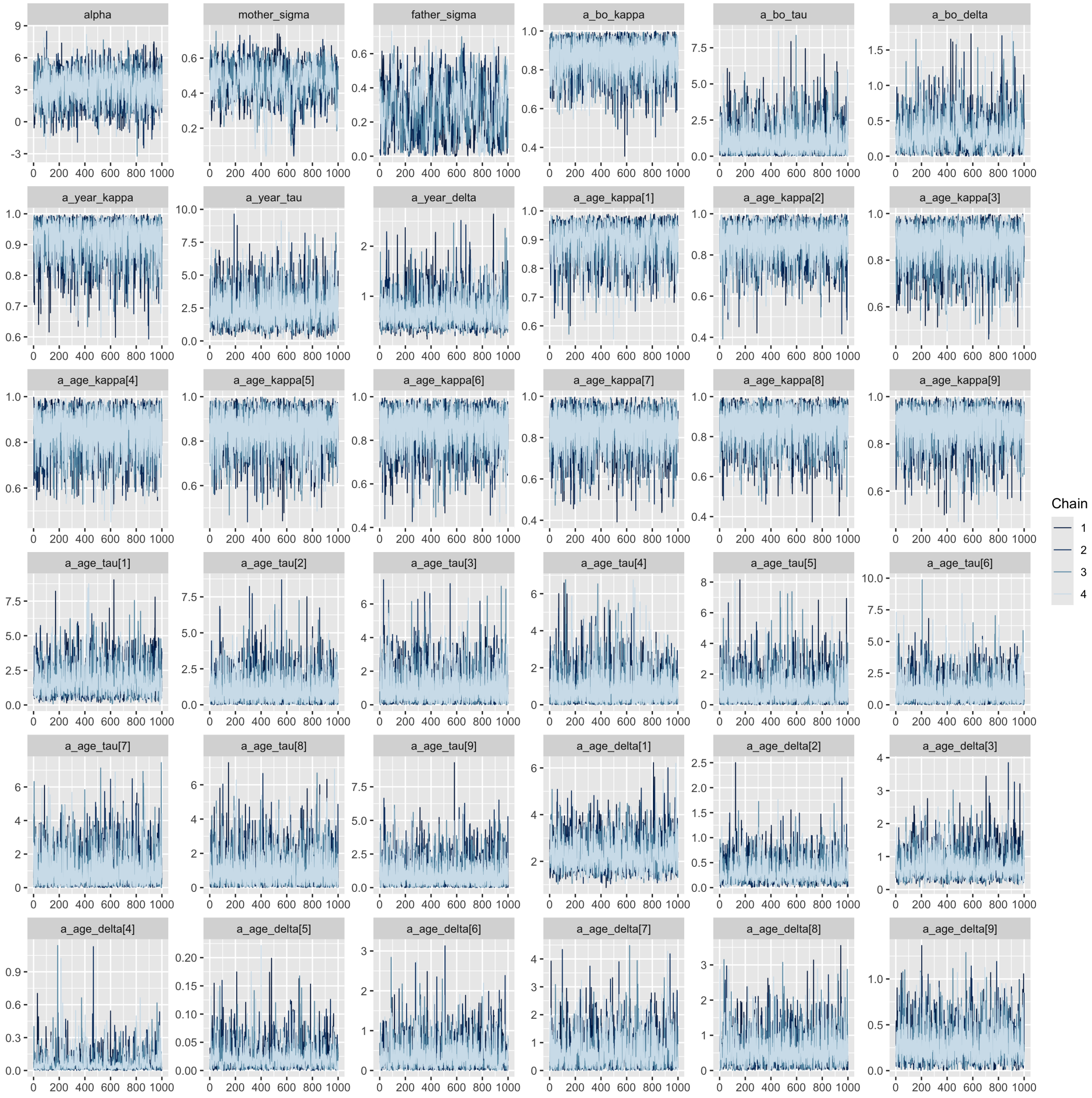

Fig. S37. Traceplots showing good mixing and convergence of four chains to the same posterior region for main model parameters—for the model of mother status at $t + 1$ and child survival at t.

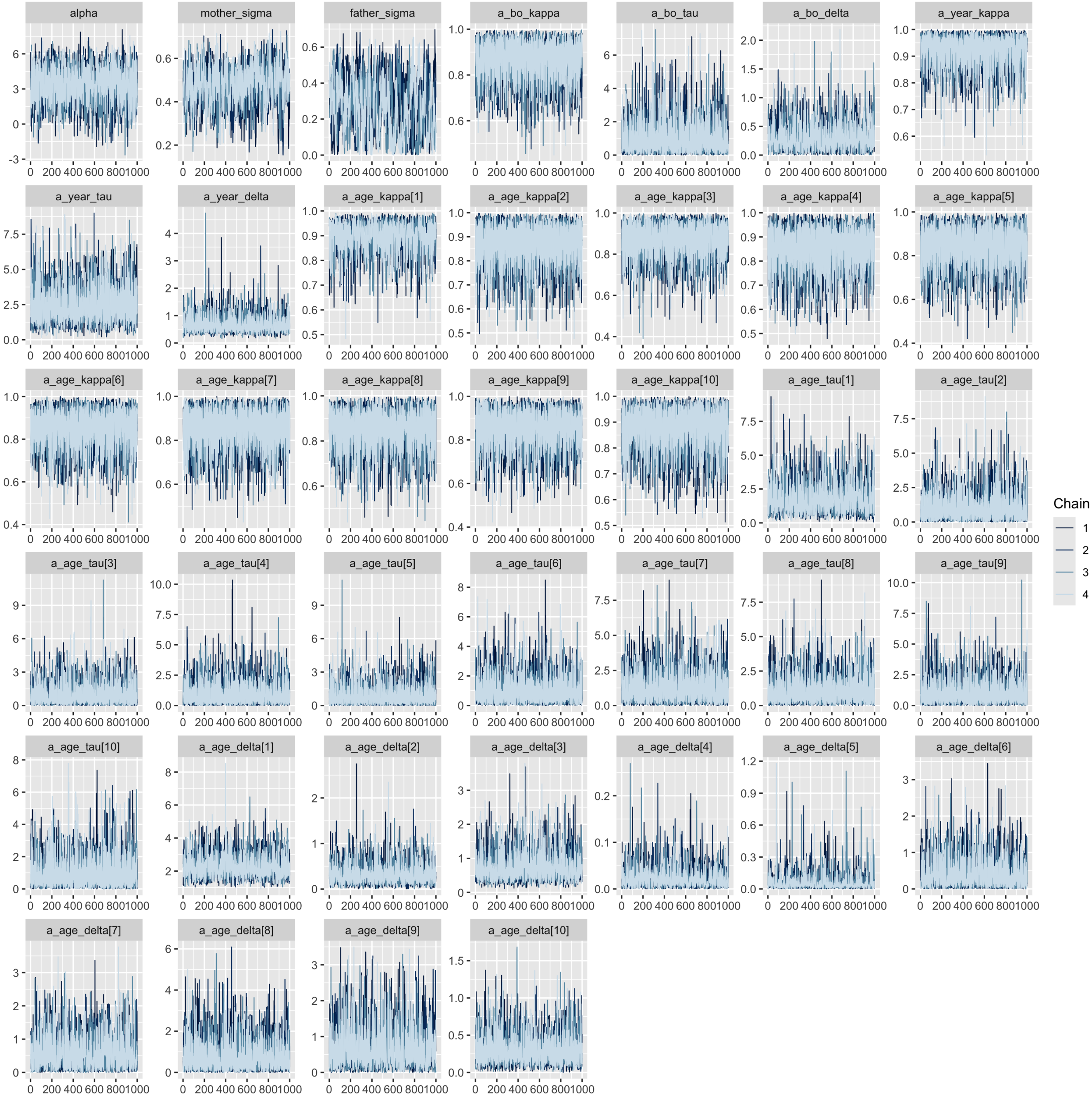

Fig. S38. Traceplots showing good mixing and convergence of four chains to the same posterior region for main model parameters—for the model of father status at $t+1$ and child survival at t.

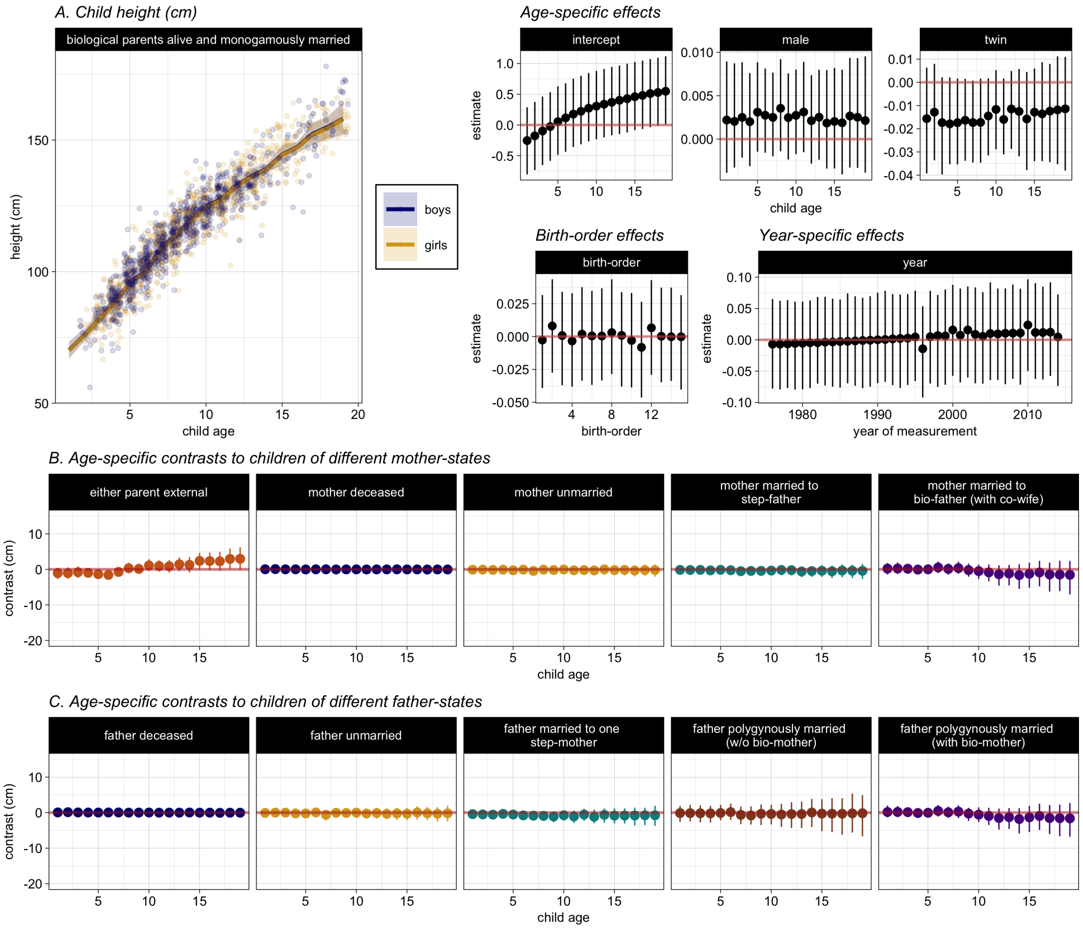

Fig. S39. Association between parent marital/vital states in year $t + 1$ and child height in year t.

	mean	sd	5.5%	94.5%	rhat	ess_bulk
α	4.50	0.37	3.89	5.01	1.00	2814.14
γ_τ	0.71	0.77	0.03	2.19	1.00	3906.91
γ_κ	0.87	0.09	0.71	0.97	1.00	3689.82
γ_δ	0.02	0.02	0.01	0.05	1.00	1627.96
ϵ_τ	0.88	0.90	0.05	2.59	1.00	3204.32
ϵ_κ	0.88	0.08	0.72	0.98	1.00	3711.35
ϵ_δ	0.04	0.03	0.02	0.09	1.00	2704.96
β_{τ_1}	2.30	0.97	0.96	4.01	1.00	2178.97
β_{τ_2}	0.95	0.99	0.05	2.82	1.00	5066.72
β_{τ_3}	0.85	0.89	0.04	2.51	1.00	4065.59
β_{τ_4}	1.06	1.00	0.09	3.02	1.00	3779.61
β_{τ_5}	1.00	0.98	0.07	2.87	1.00	3510.08
β_{τ_6}	0.91	0.96	0.04	2.80	1.00	4584.39
β_{τ_7}	0.92	0.96	0.05	2.73	1.00	4227.54
β_{τ_8}	1.14	1.16	0.07	3.26	1.00	4078.54
β_{τ_9}	1.44	1.12	0.22	3.50	1.00	3450.43
β_{κ_1}	0.43	0.19	0.22	0.79	1.00	2602.99
β_{κ_2}	0.01	0.01	0.00	0.02	1.00	2017.62
β_{κ_3}	0.02	0.02	0.01	0.06	1.00	2352.00
β_{κ_4}	0.00	0.00	0.00	0.00	1.00	528.16
β_{κ_5}	0.00	0.00	0.00	0.00	1.01	438.73
β_{κ_6}	0.01	0.01	0.00	0.03	1.00	2093.04
β_{κ_7}	0.01	0.01	0.00	0.03	1.00	2135.12
β_{κ_8}	0.02	0.02	0.00	0.06	1.00	1569.73
β_{κ_9}	0.03	0.02	0.01	0.05	1.00	2377.60
β_{δ_1}	1.00	0.00	1.00	1.00	1.00	1972.83
β_{δ_2}	0.87	0.08	0.71	0.98	1.00	4847.50
β_{δ_3}	0.88	0.08	0.73	0.98	1.00	6356.19
β_{δ_4}	0.87	0.09	0.71	0.97	1.00	3242.56
β_{δ_5}	0.86	0.09	0.69	0.97	1.00	4382.42
β_{δ_6}	0.86	0.09	0.70	0.97	1.00	5517.82
β_{δ_7}	0.87	0.09	0.70	0.97	1.00	5256.50
β_{δ_8}	0.87	0.09	0.70	0.98	1.00	5152.89
β_{δ_9}	0.89	0.08	0.75	0.98	1.00	3585.12
κ_σ	0.03	0.00	0.02	0.03	1.00	1133.21
η_σ	0.03	0.00	0.03	0.04	1.00	1233.61
π_σ	0.06	0.00	0.05	0.06	1.00	1501.50

Table S27. Mean, standard deviation, 5.5% and 94.5% intervals, number of effective samples and Rhat values, for model parameters—modeling association between mother status at $t + 1$ and child height at t.

	mean	sd	5.5%	94.5%	rhat	ess_bulk
α	4.49	0.36	3.87	4.99	1.00	1589.52
γ_{τ}	0.70	0.76	0.04	2.07	1.00	3907.99
γ_{κ}	0.87	0.08	0.72	0.97	1.00	3195.33
γ_{δ}	0.02	0.01	0.01	0.04	1.00	1250.43
ϵ_{τ}	0.87	0.88	0.05	2.58	1.00	2540.99
ϵ_{κ}	0.88	0.08	0.72	0.98	1.00	2732.97
ϵ_{δ}	0.04	0.03	0.02	0.09	1.00	1893.52
β_{τ_1}	2.34	0.99	0.98	4.05	1.00	1204.18
β_{τ_2}	0.94	0.98	0.05	2.84	1.00	4866.24
β_{τ_3}	0.86	0.93	0.04	2.60	1.00	3761.68
β_{τ_4}	1.02	1.00	0.07	2.91	1.00	3779.09
β_{τ_5}	1.10	1.00	0.10	2.96	1.00	2448.77
β_{τ_6}	0.89	0.90	0.05	2.58	1.00	4349.40
β_{τ_7}	0.93	0.97	0.05	2.80	1.00	4370.37
β_{τ_8}	0.95	1.00	0.05	2.89	1.00	4444.50
β_{τ_9}	1.09	1.09	0.07	3.14	1.00	3708.08
$\beta_{\tau_{10}}$	1.43	1.08	0.23	3.45	1.00	2208.02
β_{κ_1}	0.43	0.21	0.22	0.81	1.00	1355.38
β_{κ_2}	0.01	0.01	0.00	0.02	1.00	1829.31
β_{κ_3}	0.02	0.02	0.01	0.06	1.00	1688.49
β_{κ_4}	0.00	0.00	0.00	0.00	1.02	328.45
β_{κ_5}	0.00	0.00	0.00	0.00	1.02	349.72
β_{κ_6}	0.01	0.01	0.00	0.04	1.00	1646.11
β_{κ_7}	0.02	0.01	0.00	0.04	1.00	1211.18
β_{κ_8}	0.03	0.03	0.00	0.08	1.00	1462.34
β_{κ_9}	0.02	0.02	0.00	0.07	1.00	1212.54
$\beta_{\kappa_{10}}$	0.03	0.02	0.01	0.05	1.00	1680.74
β_{δ_1}	1.00	0.00	1.00	1.00	1.01	1128.54
β_{δ_2}	0.87	0.08	0.71	0.98	1.00	5328.33
β_{δ_3}	0.88	0.08	0.73	0.98	1.00	4977.06
β_{δ_4}	0.86	0.09	0.69	0.97	1.00	4121.41
β_{δ_5}	0.87	0.09	0.71	0.97	1.00	3076.28
β_{δ_6}	0.86	0.09	0.69	0.97	1.00	6291.72
β_{δ_7}	0.87	0.09	0.71	0.97	1.00	4894.34
β_{δ_8}	0.87	0.09	0.70	0.97	1.00	5740.25
β_{δ_9}	0.87	0.09	0.70	0.98	1.00	5853.62
$\beta_{\delta_{10}}$	0.89	0.08	0.75	0.98	1.00	2309.92
κ_{σ}	0.03	0.00	0.03	0.04	1.00	732.28
η_{σ}	0.03	0.00	0.02	0.03	1.01	738.74
π_{σ}	0.06	0.00	0.05	0.06	1.00	1418.64

Table S28. Mean, standard deviation, 5.5% and 94.5% intervals, number of effective samples and Rhat values, for model parameters—modeling association between father status at $t+1$ and child height at t.

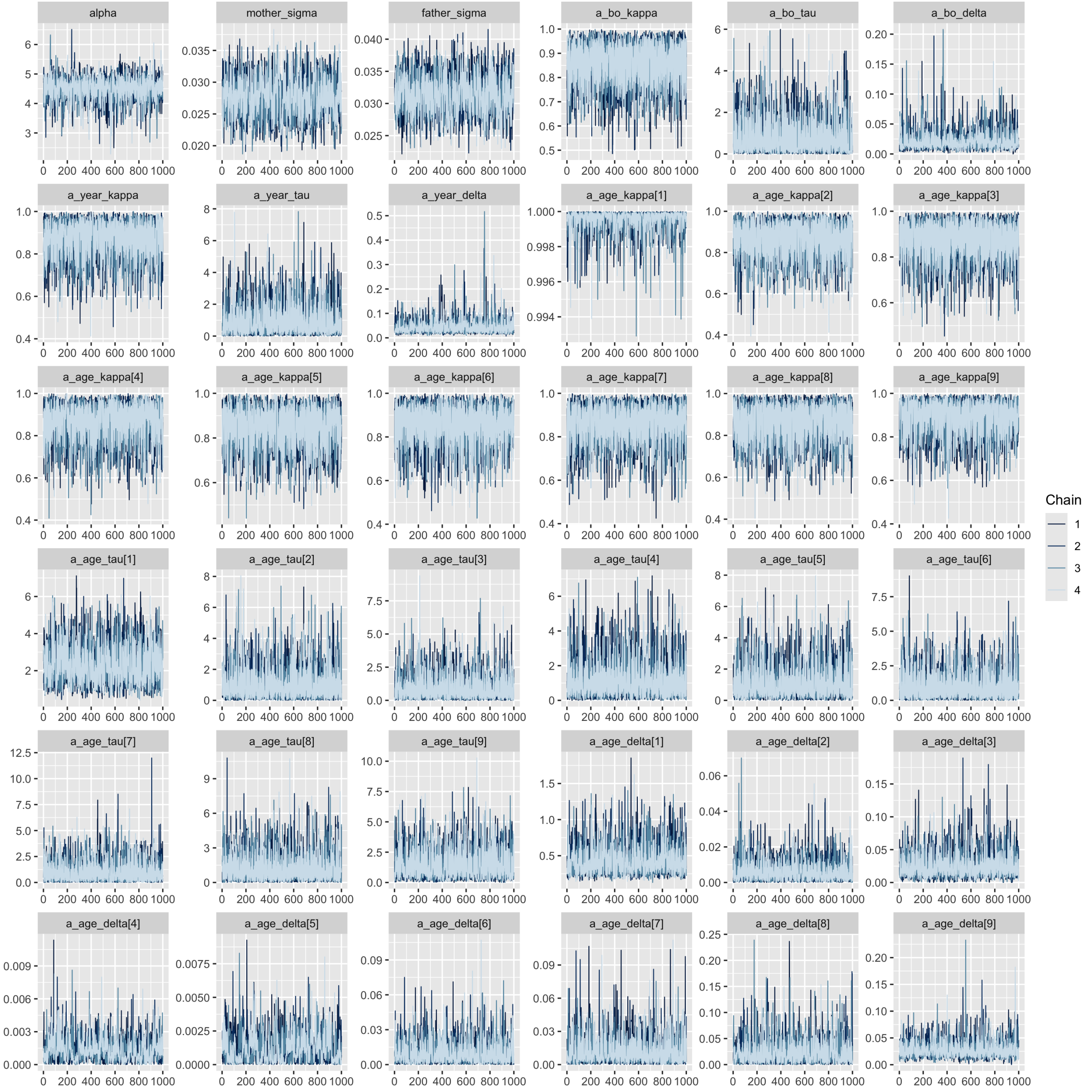

Fig. S40. Traceplots showing good mixing and convergence of four chains to the same posterior region for main model parameters—for the model of mother status at $t+1$ and child height at t.

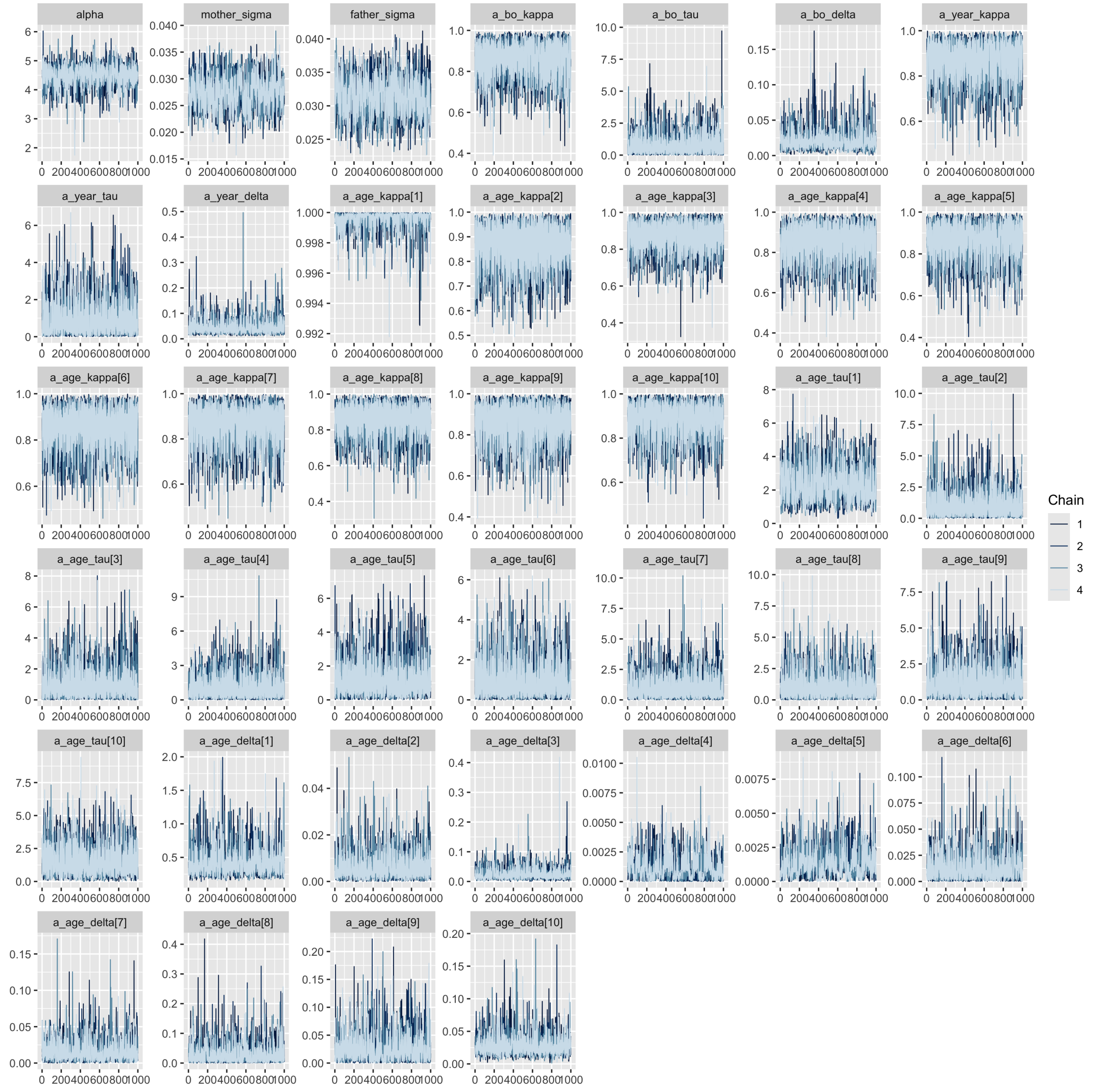

Fig. S41. Traceplots showing good mixing and convergence of four chains to the same posterior region for main model parameters—for the model of father status at $t+1$ and child height at t.

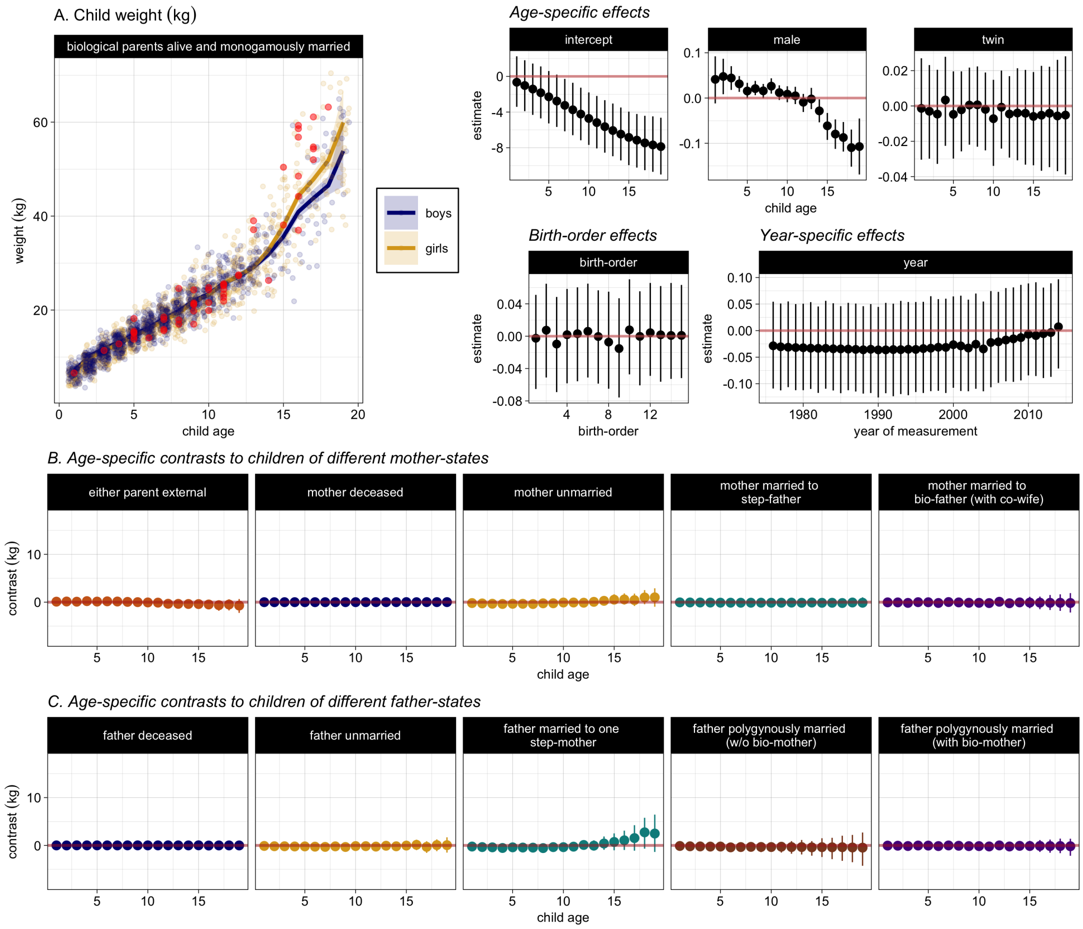

Fig. S42. Association between parent marital/vital states in year $t + 1$ and child weight in year t.

	mean	sd	5.5%	94.5%	rhat	ess_bulk
α	-1.21	1.78	-3.90	1.76	1.02	302.79
γ_τ	0.74	0.82	0.03	2.30	1.01	606.10
γ_κ	0.87	0.08	0.70	0.97	1.00	505.24
γ_δ	0.03	0.02	0.01	0.07	1.01	333.96
ϵ_τ	1.56	1.18	0.21	3.77	1.00	371.20
ϵ_κ	0.92	0.07	0.80	0.99	1.02	277.07
ϵ_δ	0.05	0.03	0.02	0.11	1.02	286.98
β_{τ_1}	1.00	0.48	0.43	1.87	1.05	149.38
β_{τ_2}	2.19	1.46	0.51	4.98	1.01	319.04
β_{τ_3}	0.93	0.96	0.05	2.74	1.00	713.05
β_{τ_4}	0.89	0.93	0.04	2.59	1.01	439.75
β_{τ_5}	0.96	0.94	0.07	2.78	1.02	363.64
β_{τ_6}	1.40	1.05	0.22	3.35	1.01	521.63
β_{τ_7}	0.96	0.97	0.04	2.83	1.01	537.01
β_{τ_8}	0.91	0.94	0.04	2.69	1.00	605.24
β_{τ_9}	1.43	1.19	0.15	3.68	1.01	525.96
$\beta_{\tau_1 0}$	0.59	0.34	0.18	1.21	1.03	151.18
β_{κ_1}	3.16	0.92	1.91	4.78	1.01	310.42
β_{κ_2}	0.09	0.04	0.05	0.17	1.01	355.30
β_{κ_3}	0.03	0.03	0.00	0.08	1.02	223.77
β_{κ_4}	0.00	0.00	0.00	0.00	1.02	105.67
β_{κ_5}	0.00	0.00	0.00	0.00	1.06	52.42
β_{κ_6}	0.04	0.02	0.01	0.08	1.02	265.83
β_{κ_7}	0.02	0.01	0.00	0.04	1.01	482.02
β_{κ_8}	0.03	0.03	0.00	0.09	1.01	373.70
β_{κ_9}	0.03	0.02	0.00	0.06	1.02	252.66
$\beta_{\kappa_1 0}$	1.20	0.40	0.74	1.93	1.01	244.22
β_{δ_1}	1.00	0.00	1.00	1.00	1.00	265.03
β_{δ_2}	0.94	0.05	0.84	0.99	1.04	244.17
β_{δ_3}	0.86	0.09	0.70	0.97	1.00	789.84
β_{δ_4}	0.84	0.09	0.68	0.96	1.00	564.60
β_{δ_5}	0.86	0.09	0.69	0.97	1.00	634.10
β_{δ_6}	0.90	0.08	0.74	0.98	1.02	358.20
β_{δ_7}	0.87	0.08	0.72	0.98	1.00	566.85
β_{δ_8}	0.87	0.09	0.70	0.97	1.01	537.76
β_{δ_9}	0.89	0.08	0.73	0.98	1.01	481.34
$\beta_{\delta_1 0}$	1.00	0.00	1.00	1.00	1.02	210.35
κ_σ	0.03	0.00	0.03	0.04	1.05	57.12
η_σ	0.05	0.00	0.04	0.06	1.01	136.61
π_σ	0.08	0.01	0.08	0.09	1.02	298.92

Table S29. Mean, standard deviation, 5.5% and 94.5% intervals, number of effective samples and Rhat values, for model parameters—modeling association between mother status at $t+1$ and child weight at t.

	mean	sd	5.5%	94.5%	rhat	ess_bulk
α	-1.03	1.81	-3.88	1.84	1.01	379.41
γ_τ	0.71	0.79	0.03	2.32	1.00	733.82
γ_κ	0.87	0.09	0.71	0.98	1.01	366.56
γ_δ	0.04	0.03	0.01	0.08	1.01	220.32
ϵ_τ	1.46	1.18	0.14	3.68	1.01	276.12
ϵ_κ	0.92	0.07	0.80	0.99	1.01	406.94
ϵ_δ	0.05	0.03	0.02	0.10	1.01	363.37
β_{τ_1}	1.04	0.50	0.44	1.94	1.01	347.27
β_{τ_2}	2.09	1.42	0.44	4.76	1.02	250.99
β_{τ_3}	0.94	0.95	0.04	2.78	1.01	653.15
β_{τ_4}	1.04	0.94	0.08	2.86	1.00	603.68
β_{τ_5}	0.88	0.87	0.04	2.51	1.01	621.44
β_{τ_6}	1.05	0.99	0.05	2.96	1.01	551.33
β_{τ_7}	1.50	1.14	0.18	3.57	1.00	476.79
β_{τ_8}	0.96	0.95	0.05	2.73	1.00	1339.48
β_{τ_9}	0.94	0.95	0.05	2.75	1.00	859.92
$\beta_{\tau_{10}}$	1.43	1.19	0.12	3.68	1.01	467.83
$\beta_{\tau_{11}}$	0.60	0.32	0.21	1.17	1.02	325.03
β_{κ_1}	3.16	0.97	1.83	4.86	1.00	351.09
β_{κ_2}	0.10	0.05	0.04	0.18	1.01	255.39
β_{κ_3}	0.03	0.03	0.00	0.09	1.01	462.64
β_{κ_4}	0.00	0.00	0.00	0.00	1.03	187.04
β_{κ_5}	0.00	0.00	0.00	0.00	1.03	282.40
β_{κ_6}	0.03	0.02	0.00	0.07	1.01	417.76
β_{κ_7}	0.07	0.05	0.02	0.14	1.02	352.47
β_{κ_8}	0.05	0.05	0.00	0.13	1.01	522.49
β_{κ_9}	0.03	0.03	0.00	0.08	1.01	545.14
$\beta_{\kappa_{10}}$	0.03	0.02	0.00	0.06	1.02	316.65
$\beta_{\kappa_{11}}$	1.17	0.37	0.73	1.86	1.01	369.25
β_{δ_1}	1.00	0.00	1.00	1.00	1.01	245.21
β_{δ_2}	0.94	0.05	0.85	0.99	1.02	215.81
β_{δ_3}	0.86	0.09	0.70	0.97	1.00	888.32
β_{δ_4}	0.87	0.09	0.71	0.98	1.00	671.63
β_{δ_5}	0.85	0.09	0.68	0.96	1.00	810.37
β_{δ_6}	0.87	0.09	0.70	0.97	1.00	701.14
β_{δ_7}	0.91	0.07	0.76	0.99	1.01	591.14
β_{δ_8}	0.87	0.09	0.71	0.97	1.00	778.86
β_{δ_9}	0.86	0.09	0.69	0.97	1.00	949.93
$\beta_{\delta_{10}}$	0.88	0.09	0.72	0.98	1.01	593.36
$\beta_{\delta_{11}}$	1.00	0.00	1.00	1.00	1.01	343.49
κ_σ	0.05	0.00	0.04	0.06	1.02	118.73
η_σ	0.03	0.01	0.02	0.04	1.03	100.02
π_σ	0.08	0.01	0.08	0.09	1.01	556.51

Table S30. Mean, standard deviation, 5.5% and 94.5% intervals, number of effective samples and Rhat values, for model parameters—modeling association between father status at $t+1$ and child weight at t.

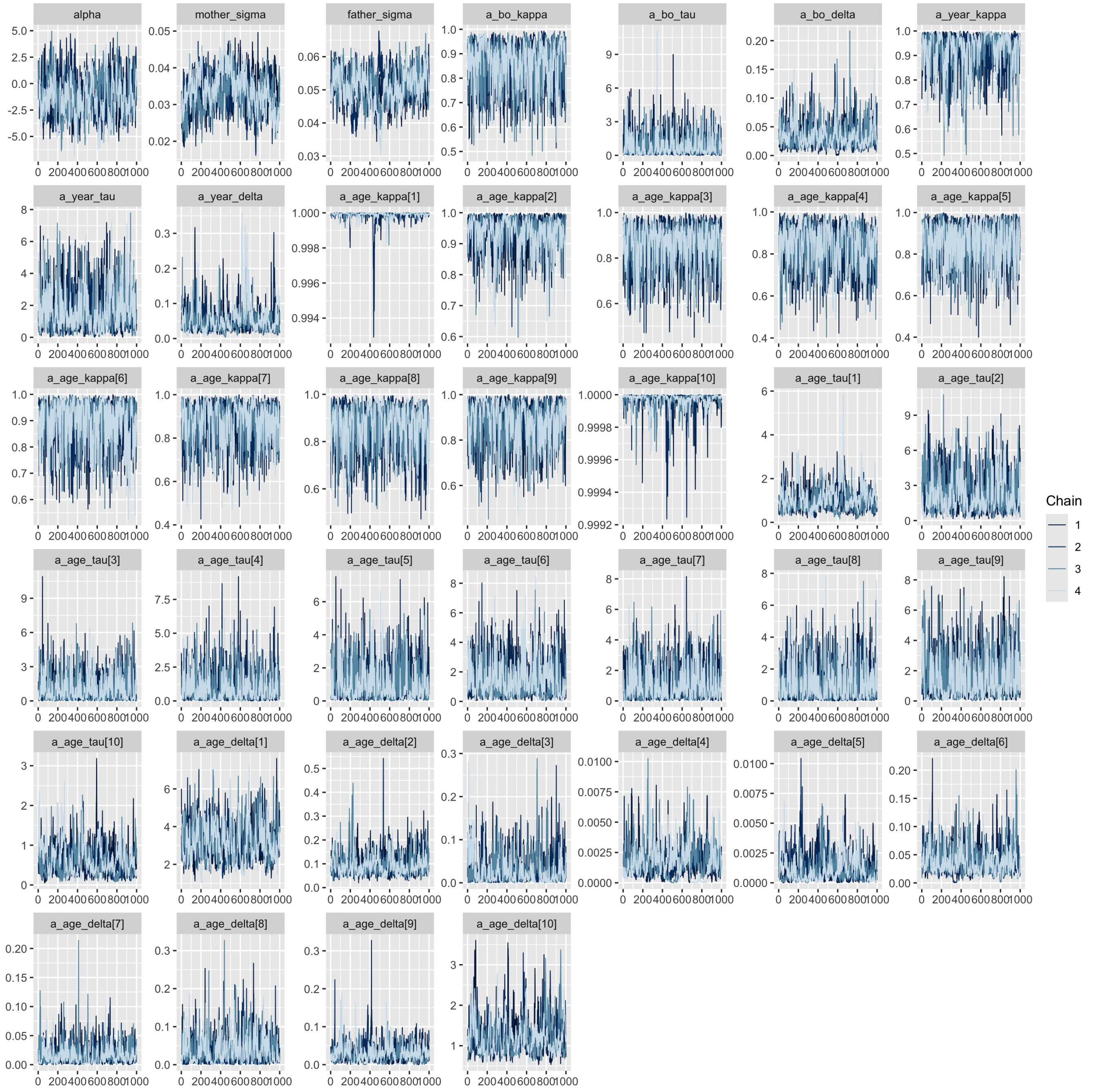

Fig. S43. Traceplots showing good mixing and convergence of four chains to the same posterior region for main model parameters—for the model of mother status at $t+1$ and child weight at t.

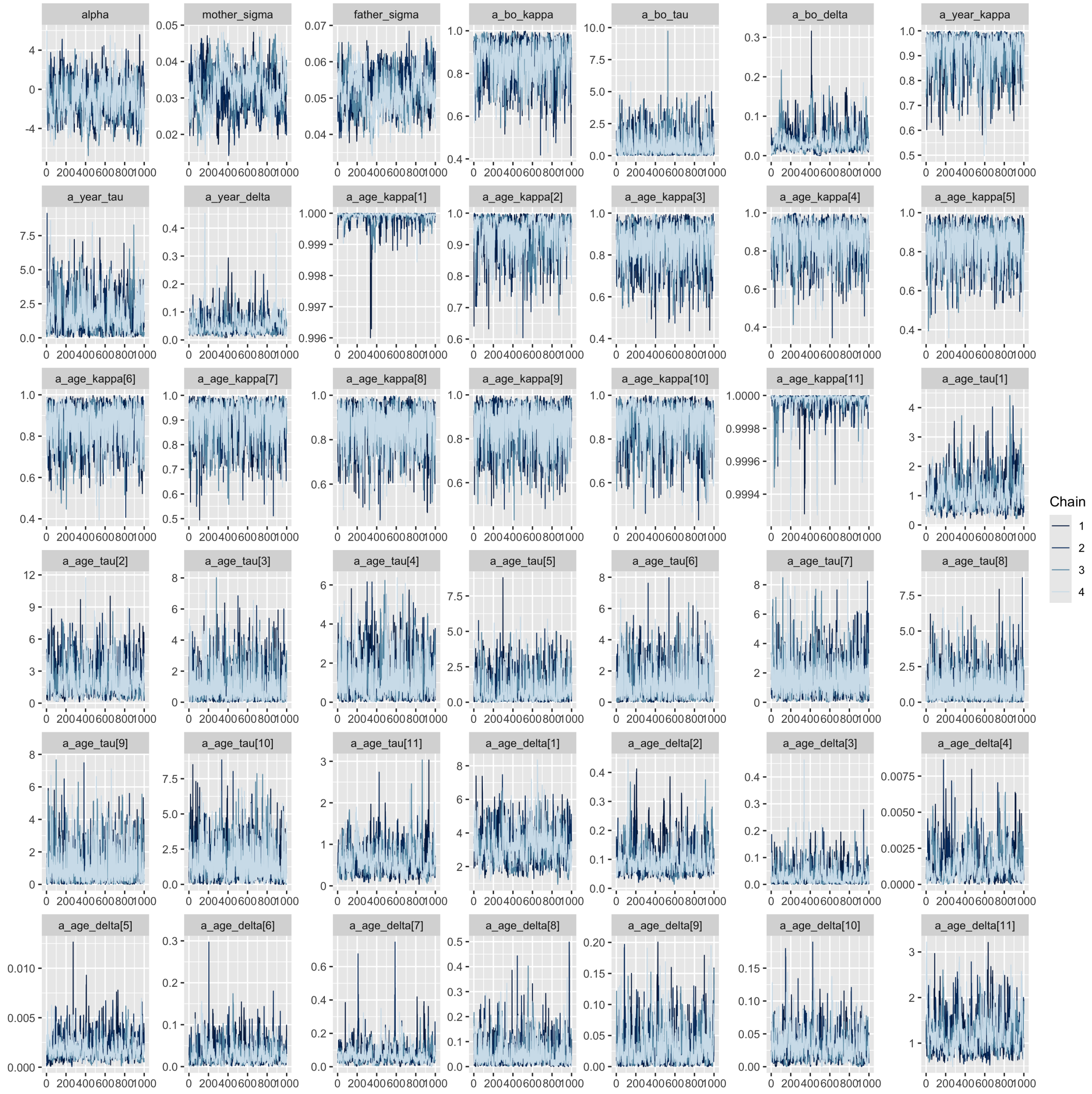

Fig. S44. Traceplots showing good mixing and convergence of four chains to the same posterior region for main model parameters—for the model of father status at $t+1$ and child weight at t.

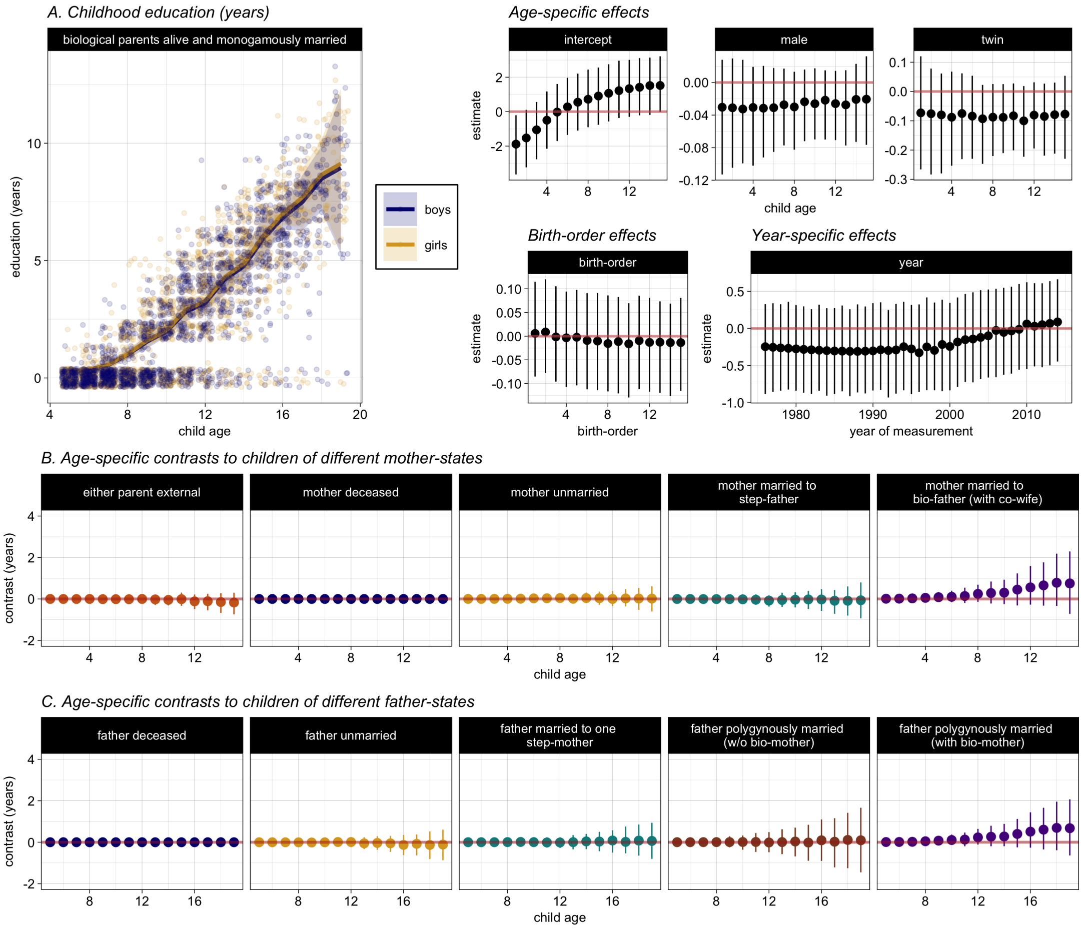

Fig. S45. Association between parent marital/vital states in year $t+1$ and child education in year t.

	mean	sd	5.5%	94.5%	rhat	ess_bulk
α	0.76	1.07	-0.91	2.47	1.00	3611.69
γ_{τ}	0.97	0.96	0.06	2.76	1.00	4803.01
γ_{κ}	0.89	0.08	0.73	0.98	1.00	4605.37
γ_{δ}	0.05	0.05	0.00	0.14	1.00	1965.09
ϵ_{τ}	1.86	1.35	0.30	4.38	1.00	1849.32
ϵ_{κ}	0.93	0.06	0.82	0.99	1.00	2386.60
ϵ_{δ}	0.38	0.21	0.18	0.73	1.00	2608.93
β_{τ_1}	2.77	1.39	1.01	5.31	1.00	1165.67
β_{τ_2}	0.95	1.00	0.04	2.88	1.00	5498.55
β_{τ_3}	0.93	0.96	0.04	2.72	1.00	4977.15
β_{τ_4}	0.98	0.95	0.07	2.80	1.00	5338.66
β_{τ_5}	0.95	0.90	0.06	2.64	1.00	4551.10
β_{τ_6}	0.97	0.97	0.06	2.83	1.00	4567.88
β_{τ_7}	0.95	0.97	0.06	2.69	1.00	4742.38
β_{τ_8}	0.94	0.94	0.05	2.78	1.00	4503.22
β_{τ_9}	0.97	0.97	0.06	2.82	1.00	5591.63
β_{κ_1}	1.55	0.57	0.86	2.61	1.00	3168.48
β_{κ_2}	0.06	0.05	0.01	0.14	1.00	1855.16
β_{κ_3}	0.15	0.12	0.02	0.37	1.00	1960.56
β_{κ_4}	0.00	0.00	0.00	0.00	1.00	2420.43
β_{κ_5}	0.00	0.00	0.00	0.00	1.00	2416.77
β_{κ_6}	0.06	0.06	0.00	0.17	1.00	2763.80
β_{κ_7}	0.09	0.09	0.00	0.25	1.00	2523.89
β_{κ_8}	0.14	0.12	0.02	0.37	1.00	1985.84
β_{κ_9}	0.06	0.06	0.00	0.16	1.00	2189.16
β_{δ_1}	0.99	0.02	0.96	1.00	1.01	586.39
β_{δ_2}	0.88	0.08	0.72	0.98	1.00	5505.56
β_{δ_3}	0.87	0.08	0.72	0.98	1.00	7374.94
β_{δ_4}	0.87	0.09	0.70	0.98	1.00	6024.45
β_{δ_5}	0.87	0.09	0.71	0.98	1.00	7211.21
β_{δ_6}	0.86	0.09	0.70	0.97	1.00	7688.21
β_{δ_7}	0.87	0.09	0.71	0.97	1.00	7301.39
β_{δ_8}	0.87	0.09	0.71	0.98	1.00	6308.98
β_{δ_9}	0.87	0.09	0.70	0.97	1.00	7464.96
κ_{σ}	0.03	0.02	0.00	0.06	1.00	1305.72
η_{σ}	0.03	0.02	0.00	0.06	1.00	1278.54

Table S31. Mean, standard deviation, 5.5% and 94.5% intervals, number of effective samples and Rhat values, for model parameters—modeling association between mother status at $t+1$ and child education at t: parameters for the model of θ, i.e., modeling the probability a child does not go to school.

	mean	sd	5.5%	94.5%	rhat	ess_bulk
α	1.69	1.78	-1.10	4.48	1.00	7658.71
γ_{τ}	1.56	1.28	0.15	3.94	1.00	3771.89
γ_{κ}	0.88	0.08	0.73	0.98	1.00	3609.17
γ_{δ}	0.84	0.40	0.36	1.56	1.00	2112.04
ϵ_{τ}	2.89	1.73	0.83	6.12	1.00	2339.96
ϵ_{κ}	0.92	0.06	0.81	0.98	1.00	2449.14
ϵ_{δ}	2.86	0.86	1.75	4.38	1.00	2948.77
β_{τ_1}	2.22	0.93	1.09	3.90	1.00	2995.64
β_{τ_2}	1.09	1.08	0.06	3.03	1.00	4721.44
β_{τ_3}	0.96	0.98	0.05	2.86	1.00	5159.17
β_{τ_4}	0.91	0.92	0.06	2.65	1.00	5386.62
β_{τ_5}	0.93	0.97	0.05	2.80	1.00	5725.00
β_{τ_6}	0.87	0.93	0.05	2.67	1.00	5583.03
β_{τ_7}	0.87	0.89	0.05	2.55	1.00	4621.56
β_{τ_8}	1.01	1.02	0.05	2.95	1.00	5221.40
β_{τ_9}	1.36	1.15	0.14	3.53	1.00	3743.76
β_{κ_1}	4.16	1.01	2.81	5.96	1.00	3278.01
β_{κ_2}	0.33	0.29	0.02	0.86	1.00	2078.57
β_{κ_3}	0.62	0.61	0.04	1.74	1.00	2100.38
β_{κ_4}	0.03	0.03	0.00	0.08	1.00	1534.32
β_{κ_5}	0.03	0.03	0.00	0.07	1.00	1602.02
β_{κ_6}	0.58	0.42	0.08	1.35	1.00	1942.81
β_{κ_7}	0.92	0.56	0.19	1.95	1.00	2143.22
β_{κ_8}	1.08	0.86	0.09	2.78	1.00	1507.08
β_{κ_9}	1.13	0.57	0.36	2.12	1.00	1910.06
β_{δ_1}	0.99	0.01	0.97	1.00	1.00	1742.36
β_{δ_2}	0.86	0.09	0.70	0.97	1.00	6078.51
β_{δ_3}	0.85	0.09	0.69	0.97	1.00	7837.94
β_{δ_4}	0.86	0.09	0.70	0.97	1.00	6556.66
β_{δ_5}	0.86	0.09	0.70	0.97	1.00	6705.28
β_{δ_6}	0.87	0.08	0.71	0.97	1.00	7141.11
β_{δ_7}	0.86	0.09	0.71	0.97	1.00	6827.11
β_{δ_8}	0.85	0.09	0.68	0.97	1.00	5199.48
β_{δ_9}	0.88	0.08	0.73	0.98	1.00	5013.81
κ_{σ}	1.05	0.29	0.55	1.47	1.02	297.41
η_{σ}	1.75	0.18	1.47	2.05	1.01	957.47

Table S32. Mean, standard deviation, 5.5% and 94.5% intervals, number of effective samples and Rhat values, for model parameters—modeling association between mother status at $t+1$ and child education at t: parameters for the model of η, i.e., modeling years of schooling, conditional on attending school.

	mean	sd	5.5%	94.5%	rhat	ess_bulk
α	0.79	1.07	-0.83	2.44	1.00	2898.62
γ_τ	0.95	0.95	0.06	2.72	1.00	4131.55
γ_κ	0.88	0.08	0.73	0.98	1.00	5659.48
γ_δ	0.05	0.05	0.00	0.14	1.00	1786.14
ϵ_τ	1.92	1.41	0.29	4.55	1.00	1731.76
ϵ_κ	0.93	0.06	0.82	0.99	1.00	2072.77
ϵ_δ	0.38	0.19	0.18	0.73	1.00	2577.61
β_{τ_1}	2.85	1.43	1.03	5.45	1.00	937.84
β_{τ_2}	0.91	0.91	0.05	2.62	1.00	5041.45
β_{τ_3}	0.92	0.94	0.05	2.69	1.00	4984.54
β_{τ_4}	0.94	0.93	0.06	2.68	1.00	4290.02
β_{τ_5}	0.96	0.98	0.04	2.92	1.00	4507.98
β_{τ_6}	0.97	0.99	0.05	2.85	1.00	4472.58
β_{τ_7}	0.96	0.94	0.05	2.76	1.00	5620.33
β_{τ_8}	0.97	0.96	0.06	2.73	1.00	4489.07
β_{τ_9}	0.94	0.97	0.05	2.76	1.00	4886.77
$\beta_{\tau_1 0}$	0.96	0.95	0.06	2.74	1.00	5239.61
β_{κ_1}	1.54	0.55	0.87	2.55	1.00	2386.55
β_{κ_2}	0.06	0.05	0.01	0.15	1.00	1980.86
β_{κ_3}	0.15	0.12	0.02	0.38	1.00	1718.62
β_{κ_4}	0.00	0.00	0.00	0.00	1.00	2669.63
β_{κ_5}	0.00	0.00	0.00	0.00	1.00	2558.81
β_{κ_6}	0.08	0.08	0.01	0.22	1.00	1745.15
β_{κ_7}	0.09	0.09	0.01	0.25	1.00	2732.03
β_{κ_8}	0.15	0.15	0.01	0.42	1.00	2806.70
β_{κ_9}	0.13	0.11	0.02	0.33	1.00	2231.59
$\beta_{\kappa_1 0}$	0.06	0.06	0.00	0.16	1.00	2036.55
β_{δ_1}	0.99	0.02	0.96	1.00	1.00	477.88
β_{δ_2}	0.88	0.08	0.72	0.98	1.00	6266.78
β_{δ_3}	0.88	0.08	0.72	0.98	1.00	5245.28
β_{δ_4}	0.87	0.08	0.71	0.97	1.00	6174.46
β_{δ_5}	0.87	0.09	0.70	0.97	1.00	6499.22
β_{δ_6}	0.86	0.09	0.70	0.97	1.00	6045.10
β_{δ_7}	0.86	0.09	0.70	0.97	1.00	6962.50
β_{δ_8}	0.86	0.09	0.70	0.97	1.00	7073.80
β_{δ_9}	0.87	0.08	0.71	0.97	1.00	6976.01
$\beta_{\delta_1 0}$	0.87	0.09	0.71	0.98	1.00	6257.30
κ_σ	0.03	0.02	0.00	0.06	1.00	1084.88
η_σ	0.03	0.02	0.00	0.06	1.00	1078.23

Table S33. Mean, standard deviation, 5.5% and 94.5% intervals, number of effective samples and Rhat values, for model parameters—modeling association between father status at $t+1$ and child education at t: parameters for the model of θ, i.e., modeling the probability a child does not go to school.

	mean	sd	5.5%	94.5%	rhat	ess_bulk
α	1.77	1.79	-1.10	4.65	1.00	6221.98
γ_τ	1.59	1.28	0.17	3.99	1.00	3800.76
γ_κ	0.88	0.08	0.73	0.98	1.00	3184.21
γ_δ	0.86	0.40	0.37	1.56	1.00	2356.71
ϵ_τ	2.87	1.68	0.83	5.95	1.00	2401.81
ϵ_κ	0.92	0.06	0.81	0.98	1.00	2354.17
ϵ_δ	2.87	0.90	1.74	4.47	1.00	3105.43
β_{τ_1}	2.21	0.94	1.10	3.96	1.00	2327.47
β_{τ_2}	1.07	1.08	0.06	3.09	1.00	4929.74
β_{τ_3}	0.97	0.99	0.06	2.78	1.00	4608.16
β_{τ_4}	0.92	0.91	0.05	2.68	1.00	4212.06
β_{τ_5}	0.92	0.94	0.05	2.74	1.00	4646.95
β_{τ_6}	0.98	0.96	0.05	2.82	1.00	4352.86
β_{τ_7}	1.04	1.05	0.05	3.06	1.00	4091.72
β_{τ_8}	1.03	1.07	0.05	3.04	1.00	4035.09
β_{τ_9}	1.05	1.01	0.06	2.92	1.00	4759.81
$\beta_{\tau_1 0}$	1.39	1.12	0.16	3.48	1.00	2998.32
β_{κ_1}	4.22	1.04	2.83	6.11	1.00	2950.70
β_{κ_2}	0.32	0.29	0.02	0.86	1.00	1950.16
β_{κ_3}	0.60	0.55	0.05	1.62	1.00	2108.88
β_{κ_4}	0.03	0.03	0.00	0.08	1.00	1466.44
β_{κ_5}	0.03	0.03	0.00	0.08	1.00	1527.37
β_{κ_6}	0.89	0.68	0.08	2.13	1.00	1269.54
β_{κ_7}	0.61	0.59	0.03	1.75	1.00	1840.26
β_{κ_8}	0.96	0.79	0.06	2.39	1.00	2259.40
β_{κ_9}	1.53	0.85	0.43	3.06	1.00	1992.41
$\beta_{\kappa_1 0}$	1.05	0.60	0.22	2.09	1.00	1533.75
β_{δ_1}	0.99	0.01	0.96	1.00	1.00	1544.74
β_{δ_2}	0.87	0.09	0.71	0.98	1.00	6974.85
β_{δ_3}	0.86	0.09	0.69	0.97	1.00	7049.87
β_{δ_4}	0.86	0.09	0.70	0.97	1.00	6307.00
β_{δ_5}	0.86	0.09	0.70	0.97	1.00	6636.54
β_{δ_6}	0.84	0.10	0.67	0.97	1.00	4606.06
β_{δ_7}	0.85	0.09	0.68	0.97	1.00	6794.61
β_{δ_8}	0.86	0.09	0.70	0.97	1.00	6559.51
β_{δ_9}	0.86	0.09	0.69	0.97	1.00	5961.35
$\beta_{\delta_1 0}$	0.88	0.08	0.74	0.98	1.00	4236.34
κ_σ	1.07	0.25	0.64	1.46	1.02	353.52
η_σ	1.76	0.18	1.47	2.05	1.00	923.05

Table S34. Mean, standard deviation, 5.5% and 94.5% intervals, number of effective samples and Rhat values, for model parameters—modeling association between father status at $t+1$ and child education at t: parameters for the model of η, i.e., modeling years of schooling, conditional on attending school.

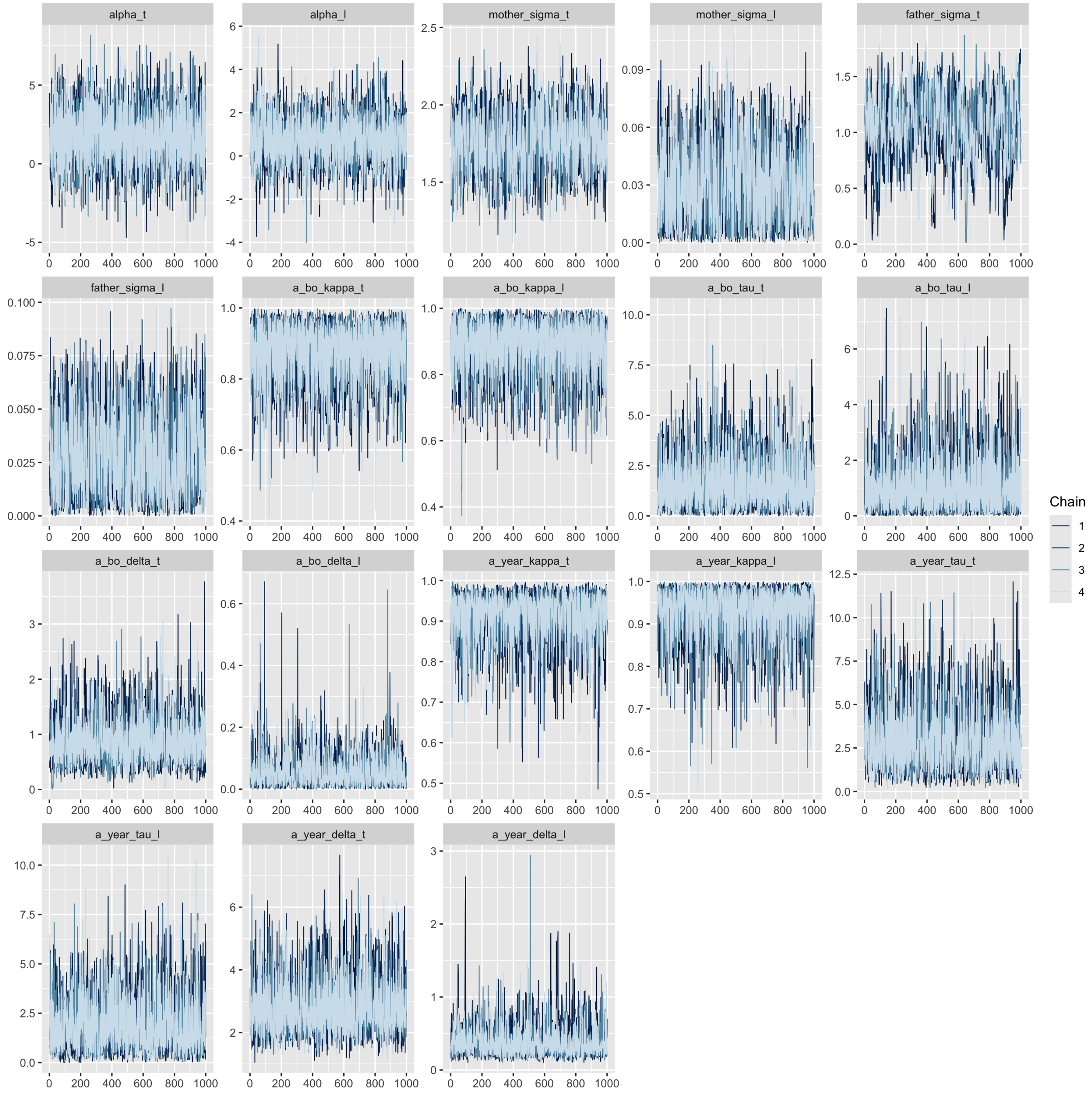

Fig. S46. Traceplots showing good mixing and convergence of four chains to the same posterior region for main model parameters—for the model of mother status at $t + 1$ and child education at t.

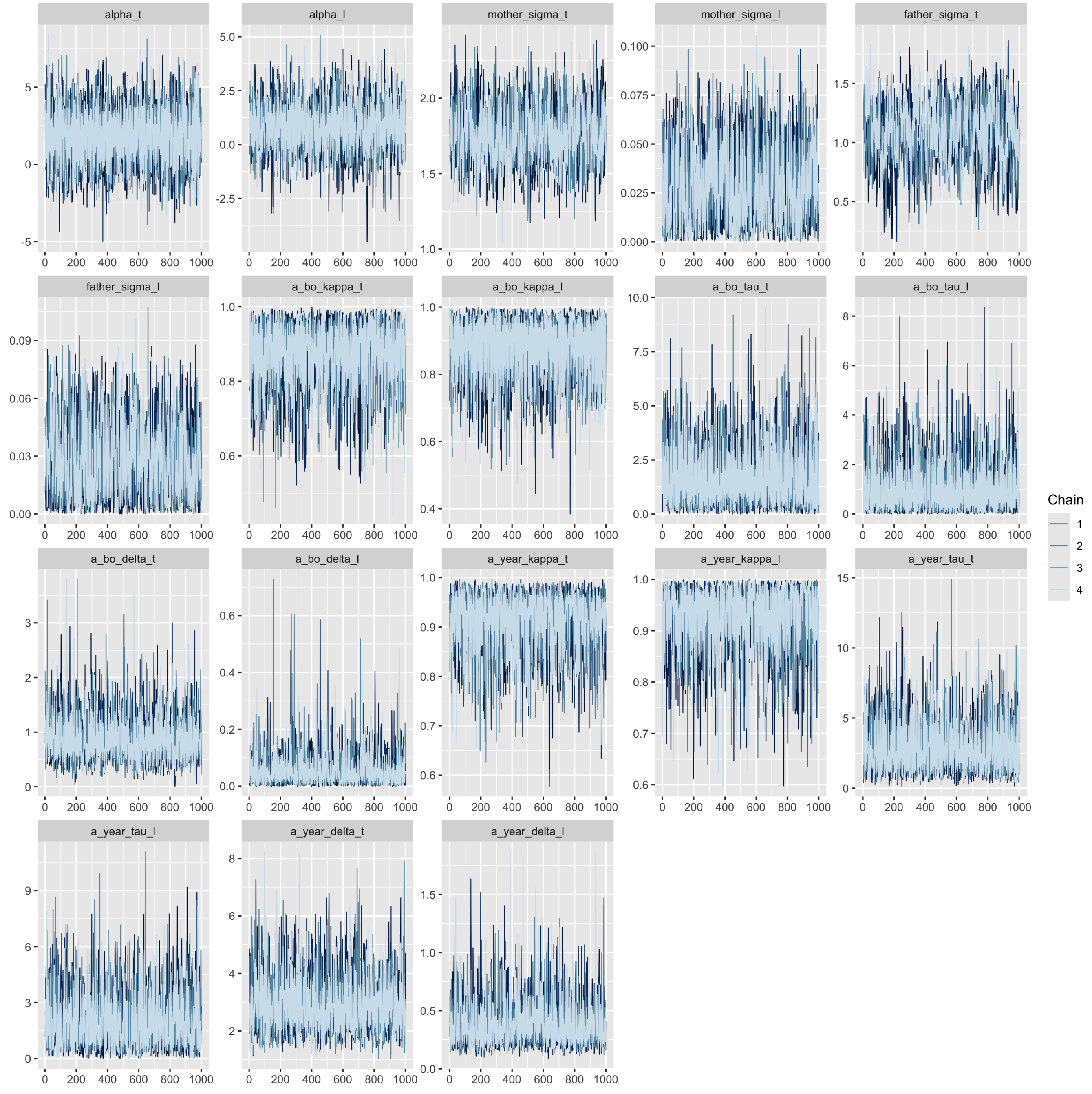

Fig. S47. Traceplots showing good mixing and convergence of four chains to the same posterior region for main model parameters—for the model of father status at $t + 1$ and child education at t.

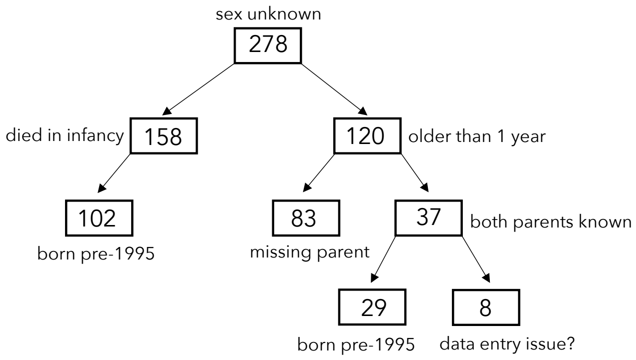

Fig. S48. Chart describing potential source of missing information on the sex of each child.

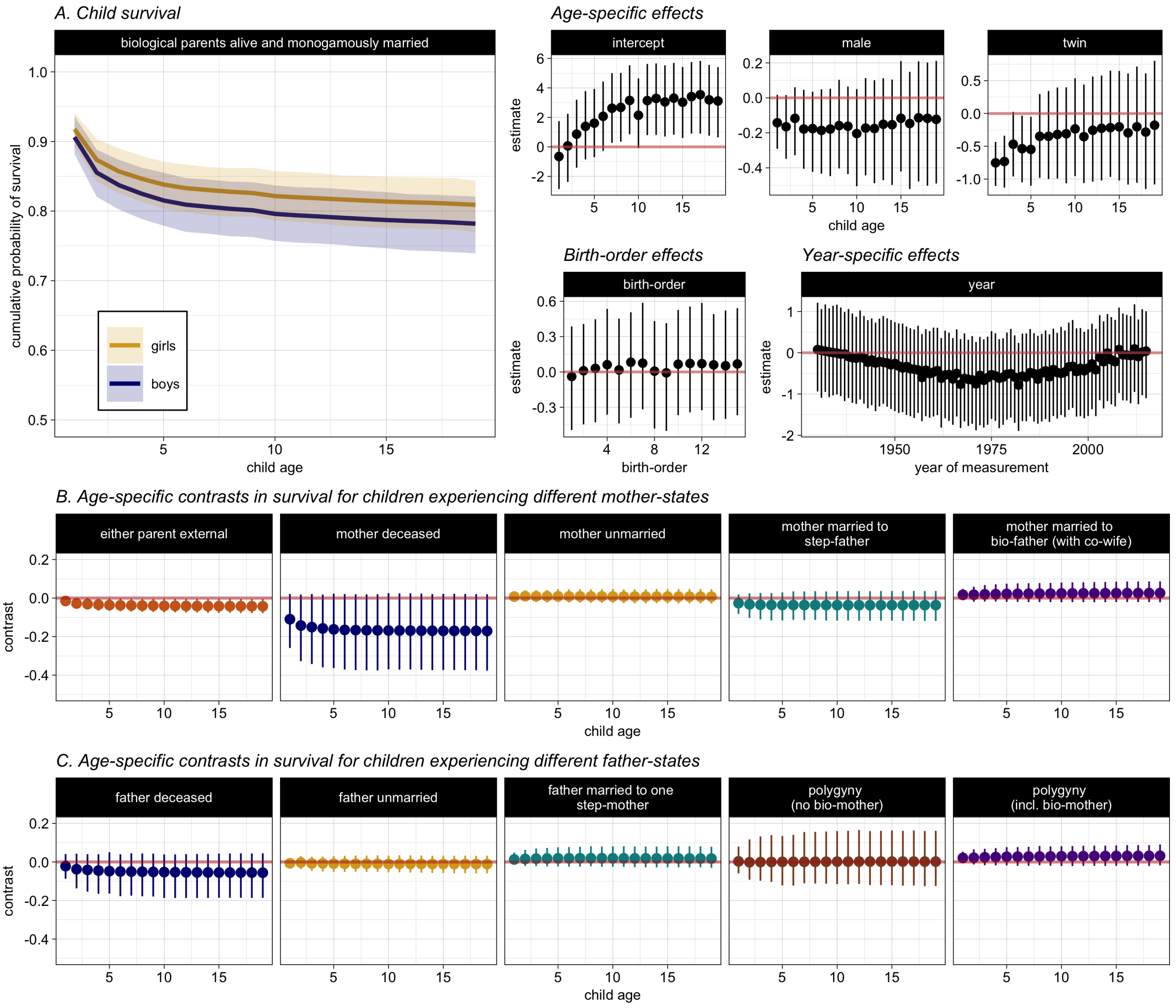

Fig. S49. Results of survival analysis, excluding records with missing information on the sex of the child.

	mean	sd	5.5%	94.5%	n_eff	Rhat4
α	3.56	1.43	1.25	5.70	2877.86	1.00
γ_τ	0.89	0.96	0.04	2.67	6656.08	1.00
γ_κ	0.87	0.08	0.72	0.98	6549.56	1.00
γ_δ	0.25	0.21	0.02	0.64	1703.66	1.00
ϵ_τ	2.42	1.24	0.85	4.62	3996.69	1.00
ϵ_κ	0.92	0.06	0.80	0.98	3116.04	1.00
ϵ_δ	0.74	0.33	0.35	1.31	2373.27	1.00
β_{τ_1}	1.97	1.18	0.64	4.13	2290.22	1.00
β_{τ_2}	0.95	0.94	0.06	2.78	7190.18	1.00
β_{τ_3}	1.08	1.08	0.06	3.16	5897.39	1.00
β_{τ_4}	0.94	0.93	0.06	2.67	6636.65	1.00
β_{τ_5}	1.01	1.02	0.05	2.95	5473.88	1.00
β_{τ_6}	1.00	1.00	0.06	2.90	6421.39	1.00
β_{τ_7}	1.00	1.02	0.05	2.99	6281.99	1.00
β_{τ_8}	0.96	0.95	0.06	2.73	6427.95	1.00
β_{τ_9}	0.91	0.95	0.05	2.66	6629.65	1.00
β_{κ_1}	2.16	0.68	1.32	3.42	2045.38	1.00
β_{κ_2}	0.28	0.21	0.05	0.67	2814.86	1.00
β_{κ_3}	0.80	0.45	0.31	1.62	2301.18	1.00
β_{κ_4}	0.53	0.47	0.04	1.43	2735.97	1.00
β_{κ_5}	0.87	0.63	0.11	1.97	2790.67	1.00
β_{κ_6}	0.32	0.32	0.02	0.92	2903.29	1.00
β_{κ_7}	0.48	0.46	0.03	1.32	2490.73	1.00
β_{κ_8}	0.53	0.54	0.03	1.50	2820.63	1.00
β_{κ_9}	0.34	0.23	0.08	0.76	2694.57	1.00
β_{δ_1}	0.93	0.05	0.84	0.99	1471.64	1.00
β_{δ_2}	0.87	0.09	0.71	0.98	7556.03	1.00
β_{δ_3}	0.87	0.09	0.70	0.98	5738.87	1.00
β_{δ_4}	0.86	0.09	0.69	0.97	6377.58	1.00
β_{δ_5}	0.86	0.09	0.70	0.97	7037.37	1.00
β_{δ_6}	0.86	0.09	0.69	0.97	7738.34	1.00
β_{δ_7}	0.86	0.09	0.69	0.97	7076.37	1.00
β_{δ_8}	0.86	0.09	0.69	0.97	6471.29	1.00
β_{δ_9}	0.87	0.09	0.71	0.98	8167.76	1.00
κ_σ	0.35	0.13	0.09	0.54	275.60	1.02
η_σ	0.27	0.15	0.03	0.51	312.95	1.01
π_σ	0.62	0.15	0.38	0.86	456.44	1.01

Table S35. Mean, standard deviation, 5.5% and 94.5% intervals, number of effective samples and Rhat values, for model parameters—modeling association between mother marital status and child survival, excluding data for children of unknown sex.

	mean	sd	5.5%	94.5%	n_eff	Rhat4
α	3.56	1.47	1.16	5.73	1675.90	1.00
γ_τ	0.89	0.90	0.05	2.53	5159.90	1.00
γ_κ	0.87	0.09	0.71	0.97	5581.26	1.00
γ_δ	0.24	0.21	0.02	0.63	1479.34	1.00
ϵ_τ	2.38	1.22	0.84	4.52	3220.54	1.00
ϵ_κ	0.92	0.06	0.80	0.98	2628.01	1.00
ϵ_δ	0.75	0.34	0.36	1.34	1810.68	1.00
β_{τ_1}	1.97	1.19	0.61	4.21	1626.93	1.00
β_{τ_2}	0.96	0.96	0.05	2.73	5218.48	1.00
β_{τ_3}	1.10	1.07	0.07	3.09	5633.97	1.00
β_{τ_4}	1.02	1.01	0.07	2.97	5852.30	1.00
β_{τ_5}	0.98	0.97	0.06	2.84	6128.80	1.00
β_{τ_6}	0.94	0.95	0.06	2.76	5687.37	1.00
β_{τ_7}	1.01	1.00	0.05	2.84	5405.03	1.00
β_{τ_8}	0.98	0.98	0.06	2.88	6048.14	1.00
β_{τ_9}	0.94	0.93	0.05	2.78	5671.04	1.00
$\beta_{\tau_{10}}$	0.91	0.95	0.04	2.80	5653.14	1.00
β_{κ_1}	2.15	0.65	1.31	3.34	1549.12	1.01
β_{κ_2}	0.28	0.22	0.05	0.68	1988.37	1.00
β_{κ_3}	0.82	0.45	0.34	1.65	2028.68	1.00
β_{κ_4}	0.83	0.62	0.09	1.93	2283.14	1.00
β_{κ_5}	0.55	0.47	0.04	1.47	2939.95	1.00
β_{κ_6}	0.50	0.49	0.03	1.43	1617.90	1.00
β_{κ_7}	0.43	0.42	0.03	1.23	2605.09	1.00
β_{κ_8}	0.72	0.72	0.05	2.06	4789.08	1.00
β_{κ_9}	0.60	0.54	0.05	1.58	1905.87	1.00
$\beta_{\kappa_{10}}$	0.32	0.22	0.07	0.73	1957.73	1.00
β_{δ_1}	0.93	0.05	0.83	0.99	947.92	1.00
β_{δ_2}	0.87	0.08	0.71	0.98	5724.43	1.00
β_{δ_3}	0.87	0.09	0.70	0.97	5362.43	1.00
β_{δ_4}	0.86	0.09	0.69	0.97	5470.20	1.00
β_{δ_5}	0.86	0.09	0.70	0.97	7128.08	1.00
β_{δ_6}	0.85	0.10	0.67	0.97	4783.27	1.00
β_{δ_7}	0.86	0.09	0.69	0.97	5303.78	1.00
β_{δ_8}	0.86	0.09	0.69	0.97	6434.48	1.00
β_{δ_9}	0.85	0.09	0.70	0.97	4920.05	1.00
$\beta_{\delta_{10}}$	0.87	0.08	0.72	0.98	5705.63	1.00
κ_σ	0.29	0.15	0.05	0.52	197.87	1.02
η_σ	0.33	0.14	0.07	0.53	170.54	1.01
π_σ	0.62	0.15	0.37	0.87	474.94	1.01

Table S36. Mean, standard deviation, 5.5% and 94.5% intervals, number of effective samples and Rhat values, for model parameters—modeling association between father marital status and child survival, excluding data for children of unknown sex.

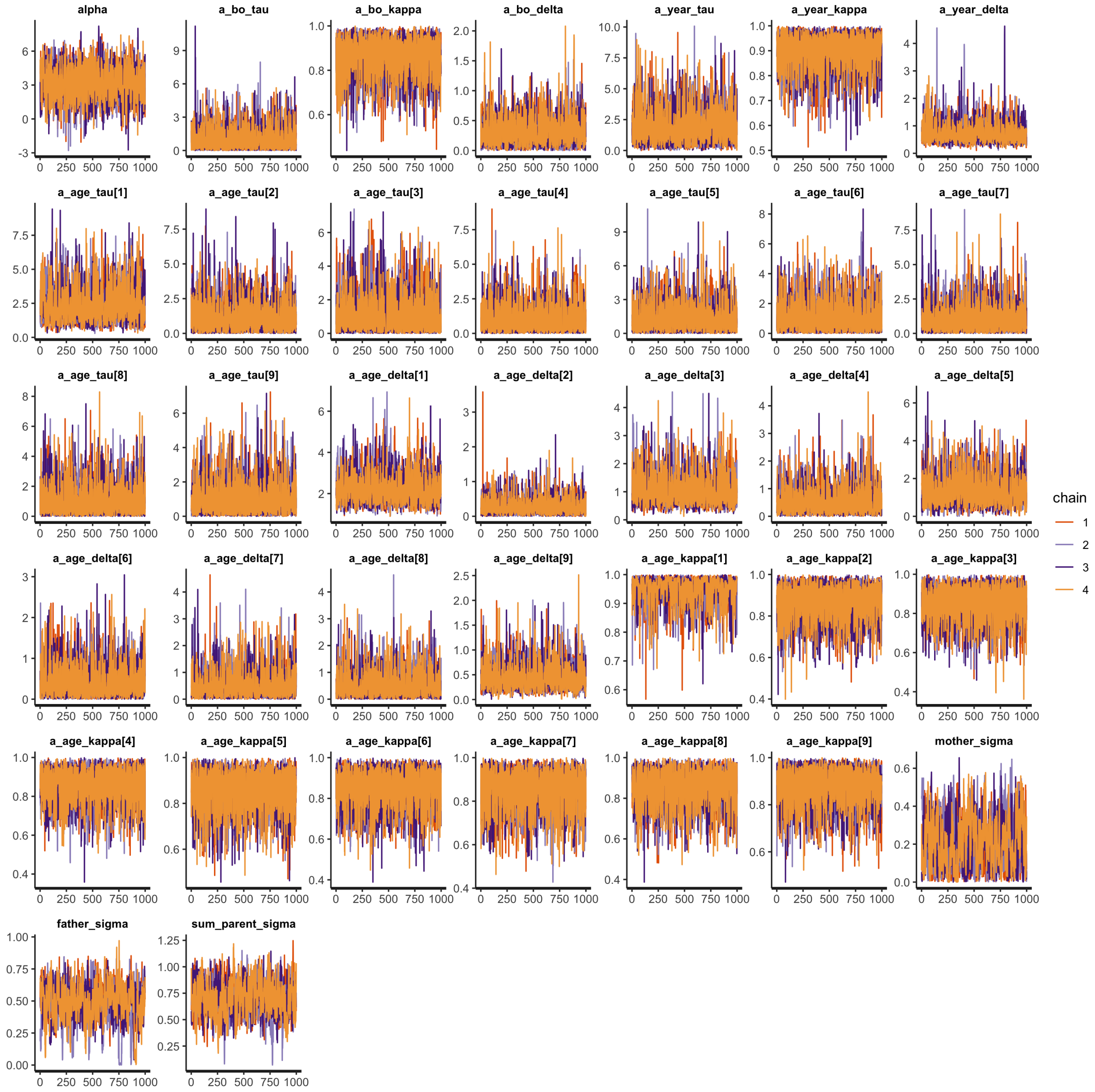

Fig. S50. Traceplots showing good mixing and convergence of four chains each for main model parameters—modeling association between mother marital status and child survival, excluding data for children of unknown sex.

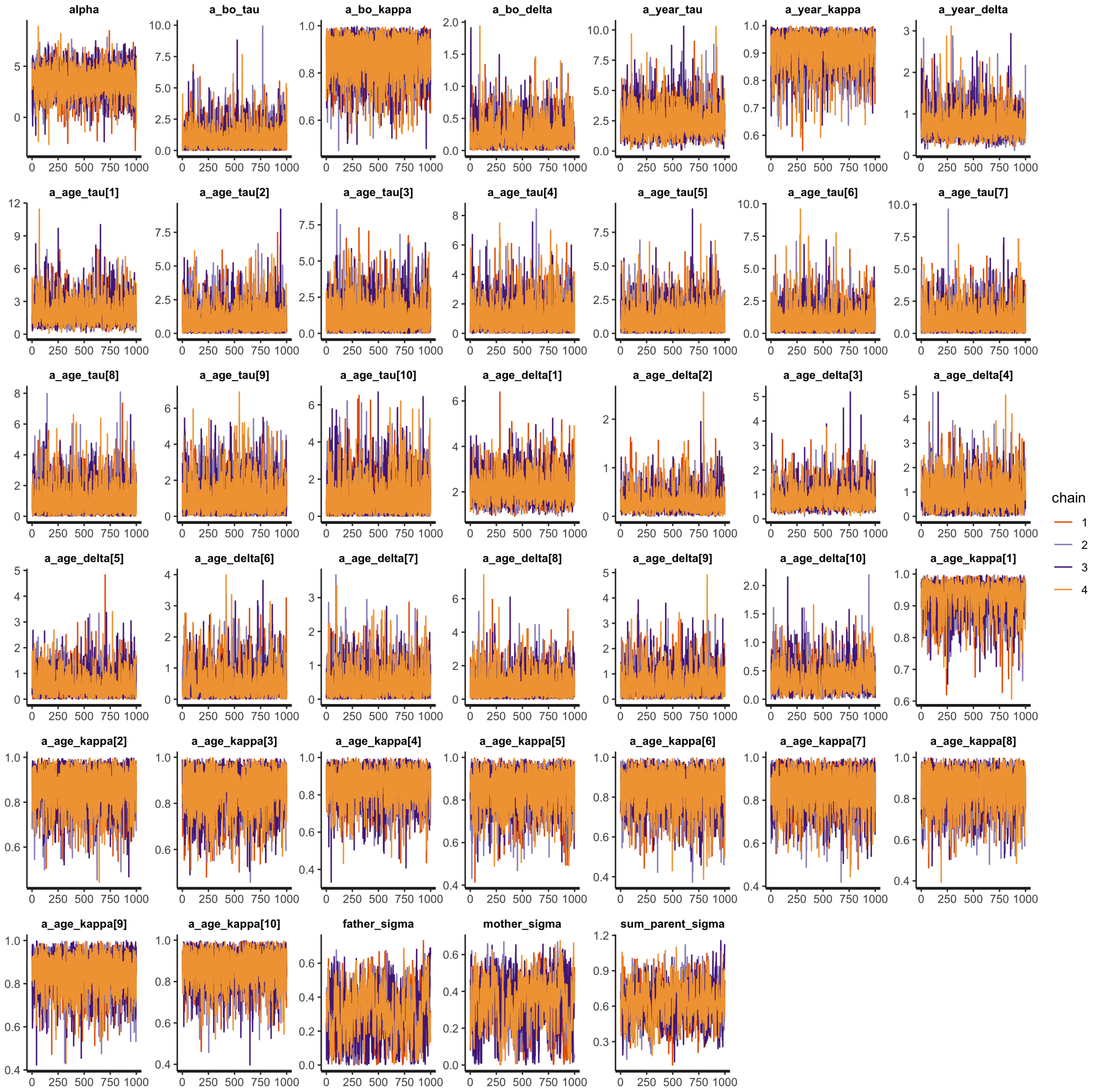

Fig. S51. Traceplots showing good mixing and convergence of four chains each for main model parameters—modeling association between father marital status and child survival, excluding data for children of unknown sex.

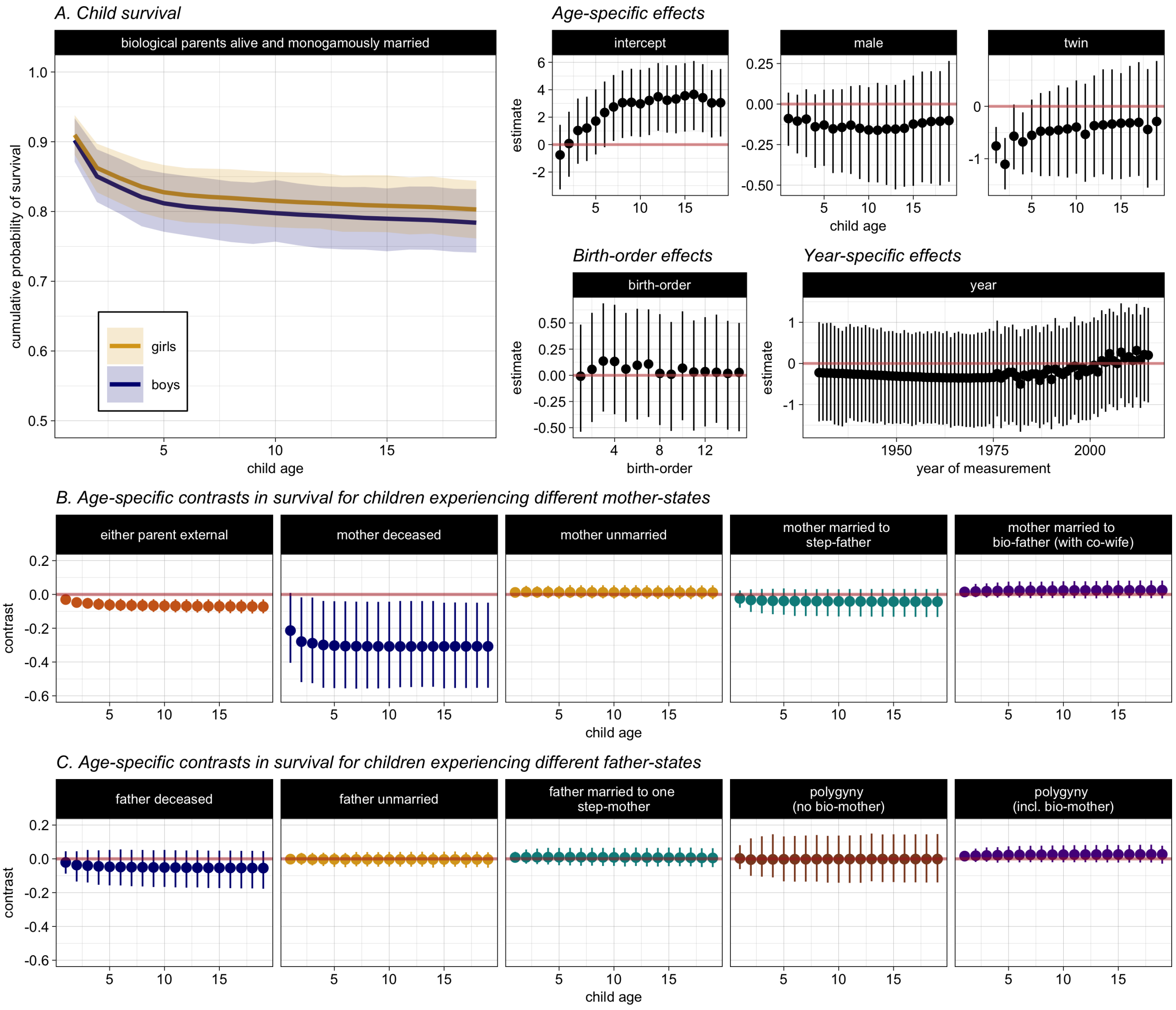

Fig. S52. Results of analysis of survival outcomes, excluding data for children born pre-1976.

	mean	sd	5.5%	94.5%	n_eff	Rhat4
α	3.42	1.47	0.97	5.51	258.86	1.01
γ_τ	0.93	1.00	0.04	2.88	556.45	1.01
γ_κ	0.87	0.08	0.71	0.97	546.73	1.01
γ_δ	0.28	0.22	0.03	0.67	259.32	1.01
ϵ_τ	1.76	1.36	0.27	4.29	230.00	1.04
ϵ_κ	0.90	0.07	0.76	0.98	306.18	1.03
ϵ_δ	0.73	0.33	0.33	1.33	182.03	1.05
β_{τ_1}	2.23	1.19	0.83	4.46	262.58	1.00
β_{τ_2}	0.99	1.03	0.03	2.94	506.50	1.00
β_{τ_3}	1.01	1.06	0.05	2.98	544.83	1.00
β_{τ_4}	1.21	1.24	0.07	3.32	473.38	1.01
β_{τ_5}	0.98	0.98	0.05	2.80	939.58	1.01
β_{τ_6}	0.92	0.93	0.06	2.67	817.36	1.00
β_{τ_7}	1.08	1.02	0.06	3.03	675.00	1.00
β_{τ_8}	1.02	0.97	0.06	2.81	815.81	1.00
β_{τ_9}	0.97	0.95	0.05	2.74	747.33	1.00
$\beta_{\tau_{10}}$	0.95	0.92	0.06	2.72	479.46	1.00
β_{κ_1}	2.16	0.64	1.28	3.30	270.86	1.00
β_{κ_2}	0.26	0.23	0.02	0.68	239.95	1.01
β_{κ_3}	1.07	0.55	0.47	2.11	206.05	1.02
β_{κ_4}	1.23	0.70	0.36	2.48	203.32	1.01
β_{κ_5}	0.55	0.47	0.04	1.46	386.12	1.01
β_{κ_6}	0.48	0.50	0.02	1.42	248.60	1.01
β_{κ_7}	0.44	0.43	0.02	1.27	424.56	1.02
β_{κ_8}	0.75	0.74	0.04	2.19	640.92	1.00
β_{κ_9}	0.48	0.45	0.03	1.32	290.27	1.01
$\beta_{\kappa_{10}}$	0.46	0.24	0.18	0.91	358.31	1.00
β_{δ_1}	0.94	0.05	0.85	0.99	197.41	1.01
β_{δ_2}	0.87	0.08	0.72	0.97	636.13	1.01
β_{δ_3}	0.86	0.08	0.70	0.97	442.75	1.00
β_{δ_4}	0.86	0.08	0.71	0.97	514.27	1.01
β_{δ_5}	0.86	0.09	0.69	0.97	511.10	1.00
β_{δ_6}	0.84	0.10	0.66	0.97	390.67	1.01
β_{δ_7}	0.87	0.09	0.71	0.97	521.80	1.01
β_{δ_8}	0.86	0.09	0.69	0.97	467.63	1.00
β_{δ_9}	0.86	0.09	0.69	0.97	526.18	1.01
$\beta_{\delta_{10}}$	0.89	0.08	0.74	0.98	438.86	1.01
κ_σ	0.44	0.16	0.06	0.65	25.76	1.14
η_σ	0.25	0.13	0.05	0.47	81.30	1.07
π_σ	0.69	0.16	0.41	0.94	51.85	1.07

Table S37. Mean, standard deviation, 5.5% and 94.5% intervals, number of effective samples and Rhat values, for model parameters—modeling association between mother marital status and child survival, excluding data for children born before the year 1976.

	mean	sd	5.5%	94.5%	n_eff	Rhat4
α	3.29	1.48	0.82	5.53	2426.22	1.00
γ_τ	0.93	1.00	0.04	2.79	5357.26	1.00
γ_κ	0.87	0.09	0.71	0.98	5407.79	1.00
γ_δ	0.29	0.23	0.03	0.69	1671.28	1.00
ϵ_τ	1.77	1.27	0.32	4.15	3373.10	1.00
ϵ_κ	0.91	0.06	0.80	0.99	4257.00	1.00
ϵ_δ	0.70	0.33	0.32	1.26	2745.40	1.00
β_{τ_1}	2.28	1.26	0.81	4.64	2293.03	1.00
β_{τ_2}	1.01	1.04	0.06	2.92	6082.42	1.00
β_{τ_3}	1.00	1.02	0.06	2.90	6955.94	1.00
β_{τ_4}	1.22	1.14	0.07	3.45	5462.88	1.00
β_{τ_5}	0.97	0.98	0.05	2.87	5613.45	1.00
β_{τ_6}	0.94	0.94	0.05	2.70	6283.23	1.00
β_{τ_7}	1.08	1.05	0.07	3.07	6523.64	1.00
β_{τ_8}	1.01	1.03	0.05	2.92	5951.01	1.00
β_{τ_9}	1.00	0.99	0.07	2.85	7598.38	1.00
$\beta_{\tau_{10}}$	0.87	0.88	0.05	2.50	6268.20	1.00
β_{κ_1}	2.30	0.73	1.38	3.57	2002.12	1.00
β_{κ_2}	0.25	0.23	0.02	0.68	2472.90	1.00
β_{κ_3}	0.99	0.52	0.40	1.94	2515.03	1.00
β_{κ_4}	1.30	0.78	0.35	2.70	2760.56	1.00
β_{κ_5}	0.58	0.49	0.04	1.47	3422.90	1.00
β_{κ_6}	0.51	0.52	0.03	1.53	1750.72	1.00
β_{κ_7}	0.49	0.49	0.03	1.41	2253.75	1.00
β_{κ_8}	0.72	0.73	0.04	2.06	4809.19	1.00
β_{κ_9}	0.51	0.50	0.04	1.44	2998.78	1.00
$\beta_{\kappa_{10}}$	0.51	0.28	0.21	1.02	2360.98	1.00
β_{δ_1}	0.95	0.04	0.87	0.99	1281.03	1.00
β_{δ_2}	0.87	0.08	0.72	0.97	6449.48	1.00
β_{δ_3}	0.86	0.09	0.69	0.97	5767.71	1.00
β_{δ_4}	0.86	0.09	0.70	0.97	6951.42	1.00
β_{δ_5}	0.86	0.09	0.70	0.97	6197.71	1.00
β_{δ_6}	0.85	0.10	0.67	0.97	7052.35	1.00
β_{δ_7}	0.86	0.09	0.69	0.97	6826.78	1.00
β_{δ_8}	0.86	0.09	0.69	0.97	7872.97	1.00
β_{δ_9}	0.86	0.09	0.69	0.97	7203.78	1.00
$\beta_{\delta_{10}}$	0.89	0.08	0.75	0.98	5570.64	1.00
κ_σ	0.37	0.18	0.05	0.64	306.05	1.01
η_σ	0.33	0.18	0.04	0.59	269.31	1.01
π_σ	0.70	0.16	0.43	0.96	823.86	1.00

Table S38. Mean, standard deviation, 5.5% and 94.5% intervals, number of effective samples and Rhat values, for model parameters—modeling association between father marital status and child survival, excluding data for children born before the year 1976.

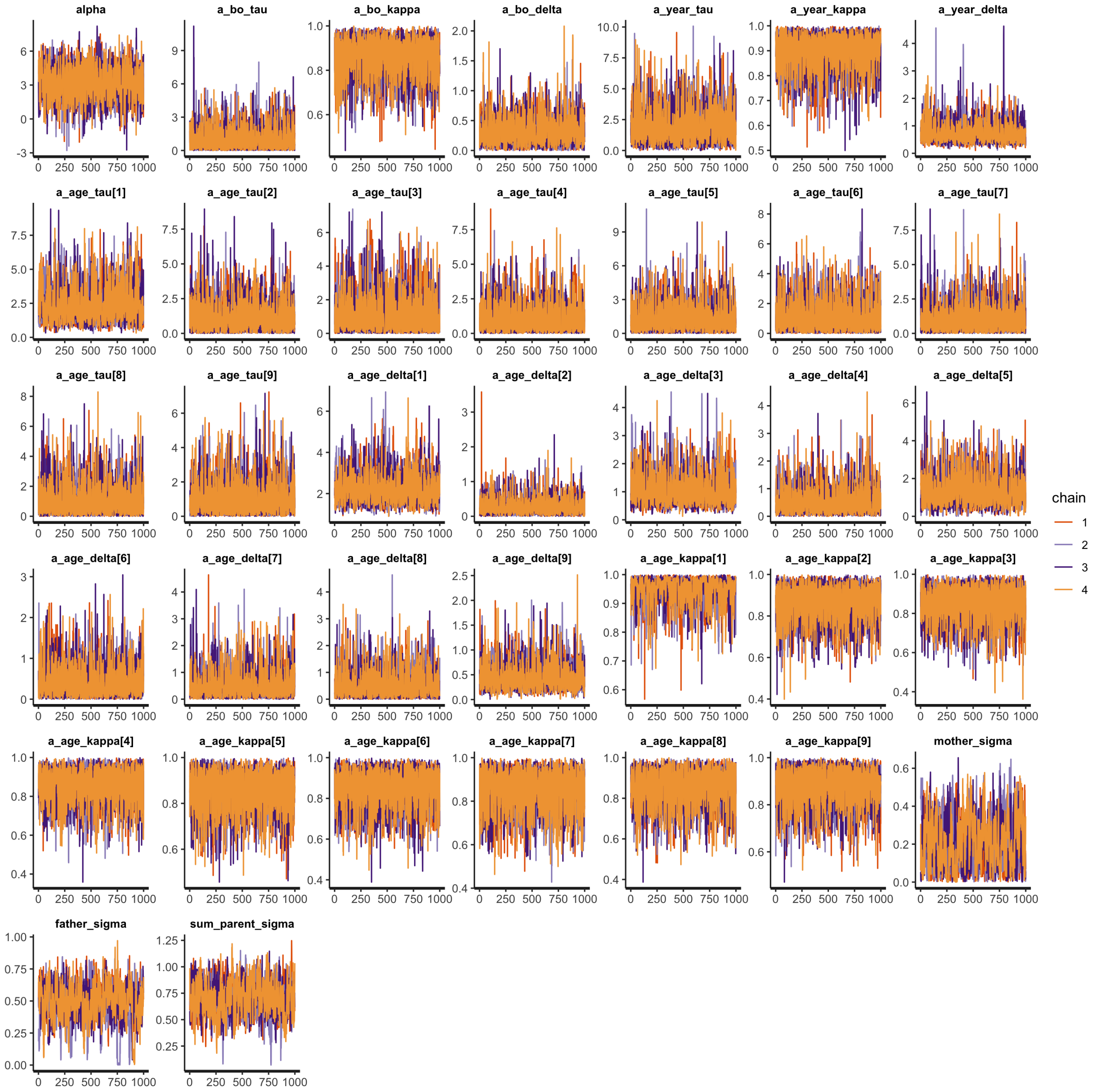

Fig. S53. Traceplots showing good mixing and convergence of four chains each for main model parameters—modeling association between mother marital status and child survival, excluding data for children born before the year 1976.

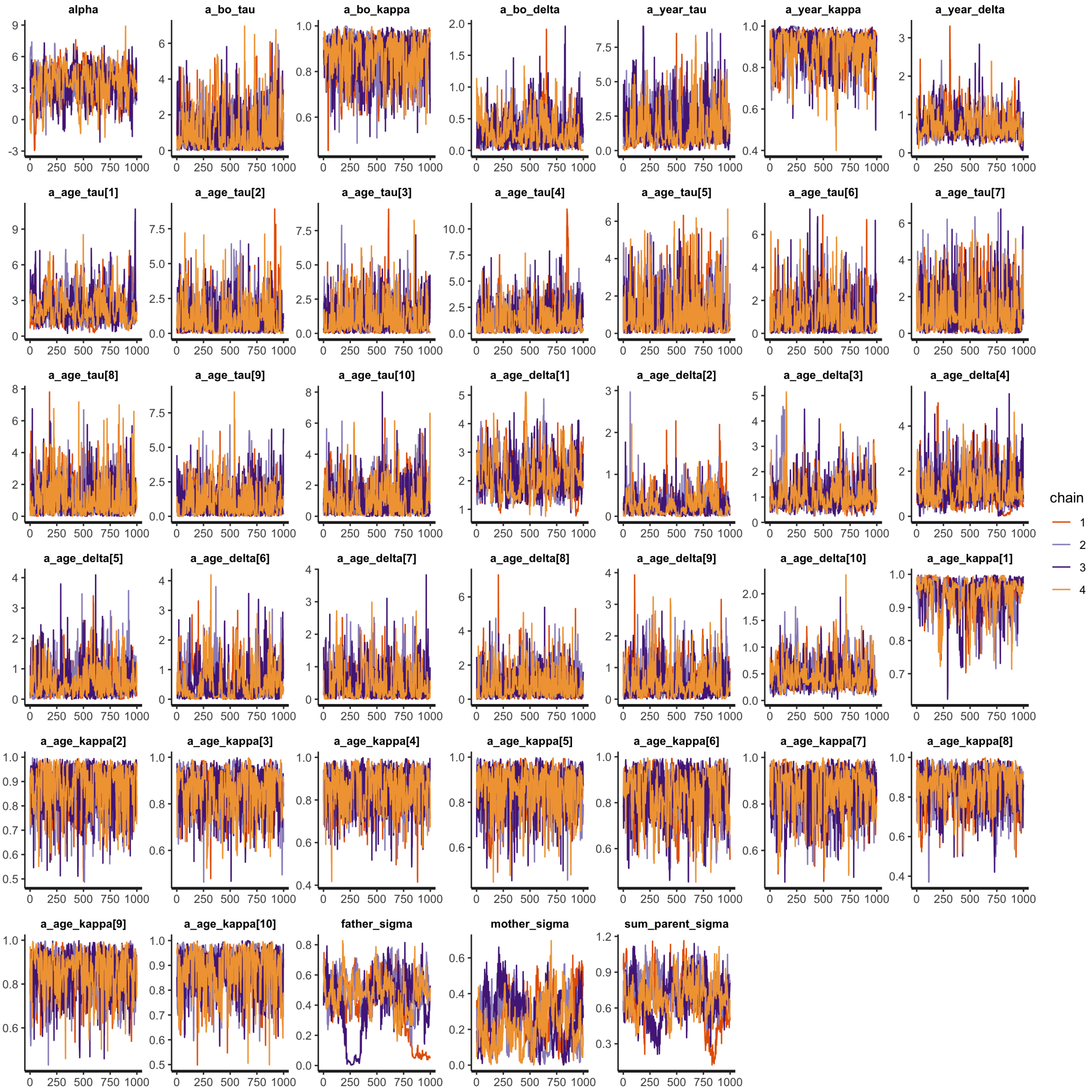

Fig. S54. Traceplots showing good mixing and convergence of four chains each for main model parameters—modeling association between father marital status and child survival, excluding data for children born before the year 1976.

Table S39. Age-specific parameter sets for which we simulate outcome data, for our power analysis.

	effect of interest
1	male
2	twin
3	mother deceased
4	father deceased
5	father unmarried
6	father married to stepmother (monogamy)
7	father married to stepmother (polygyny)
8	father married to biological mother (polygyny)
9	parent external

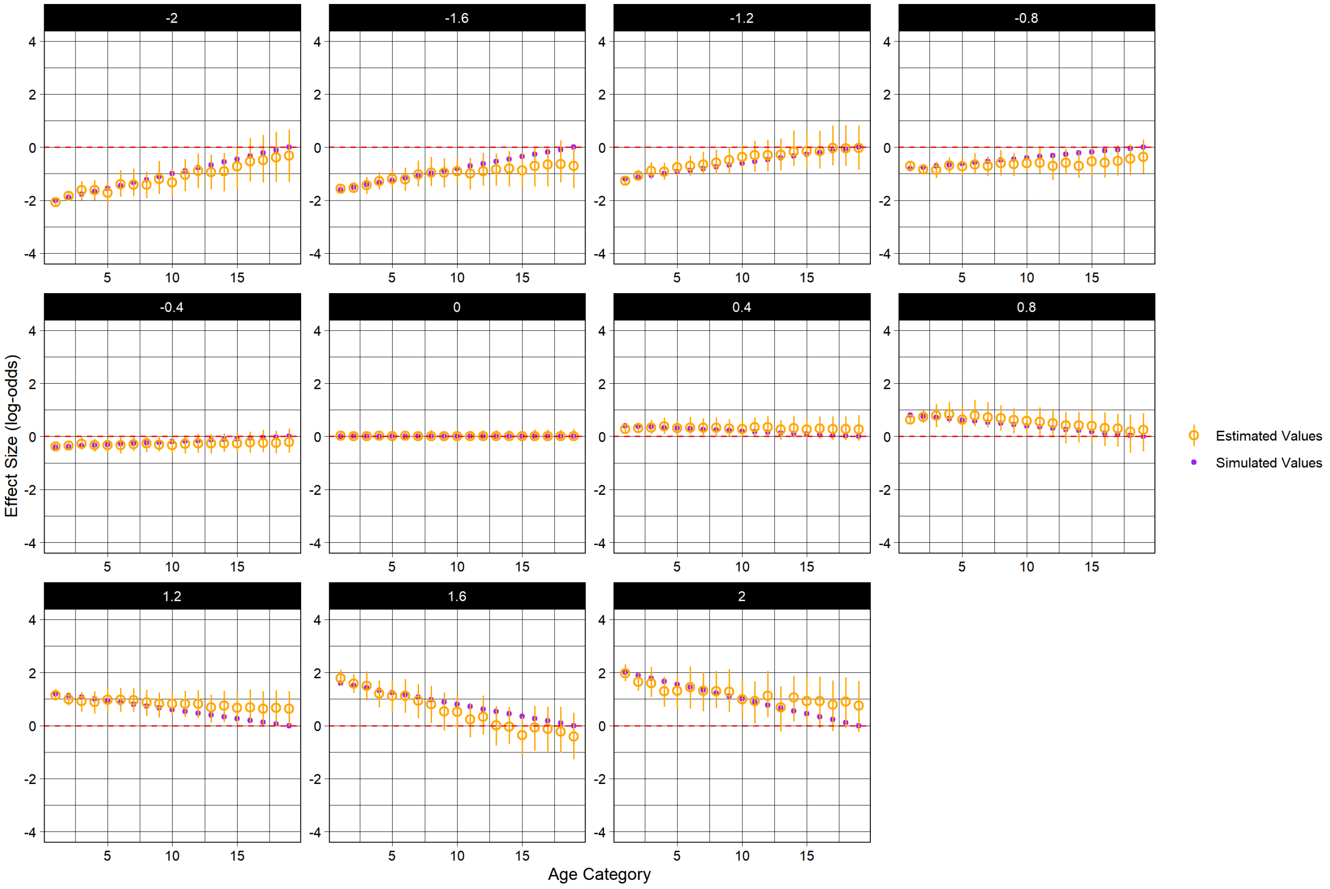

Fig. S55. Survival power analysis for the “male” effect. Each panel shows the simulated (purple) and estimated (yellow) effect sizes at each age, for different ranges of simulated parameter values.

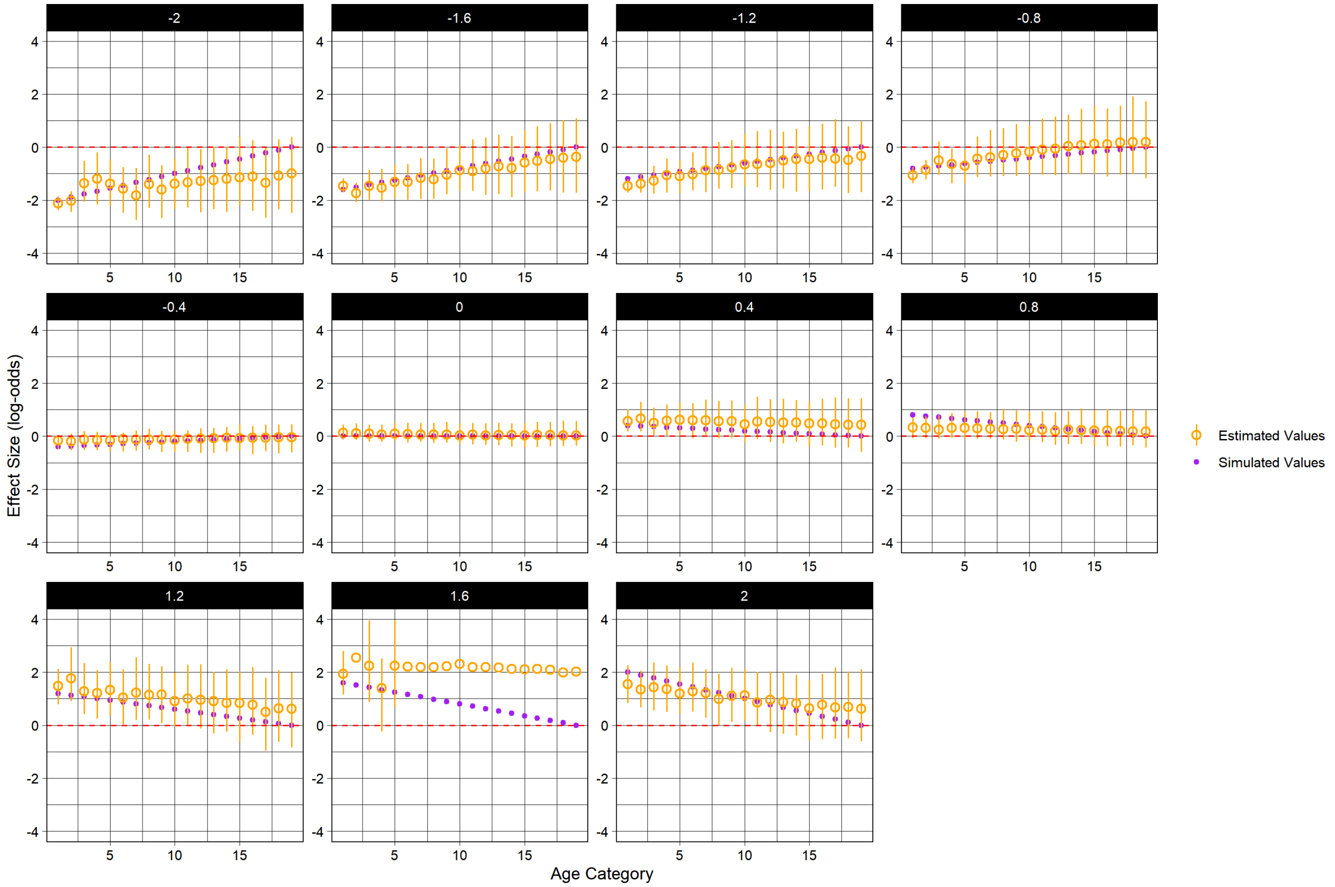

Fig. S56. Survival power analysis for the "twin" effect. Each panel shows the simulated (purple) and estimated (yellow) effect sizes at each age, for different ranges of simulated parameter values.

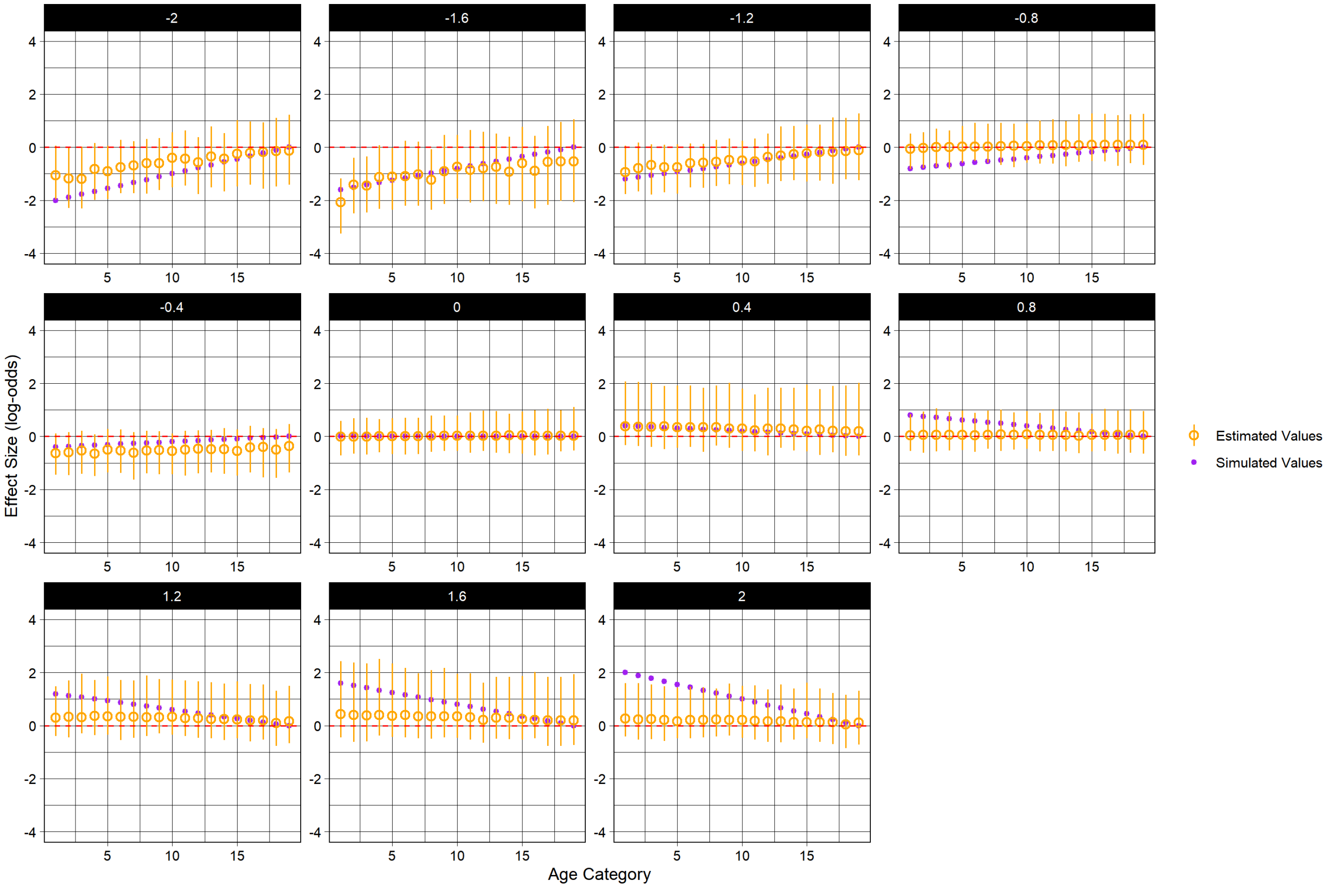

Fig. S57. Survival power analysis for the “mother deceased” effect. Each panel shows the simulated (purple) and estimated (yellow) effect sizes at each age, for different ranges of simulated parameter values.

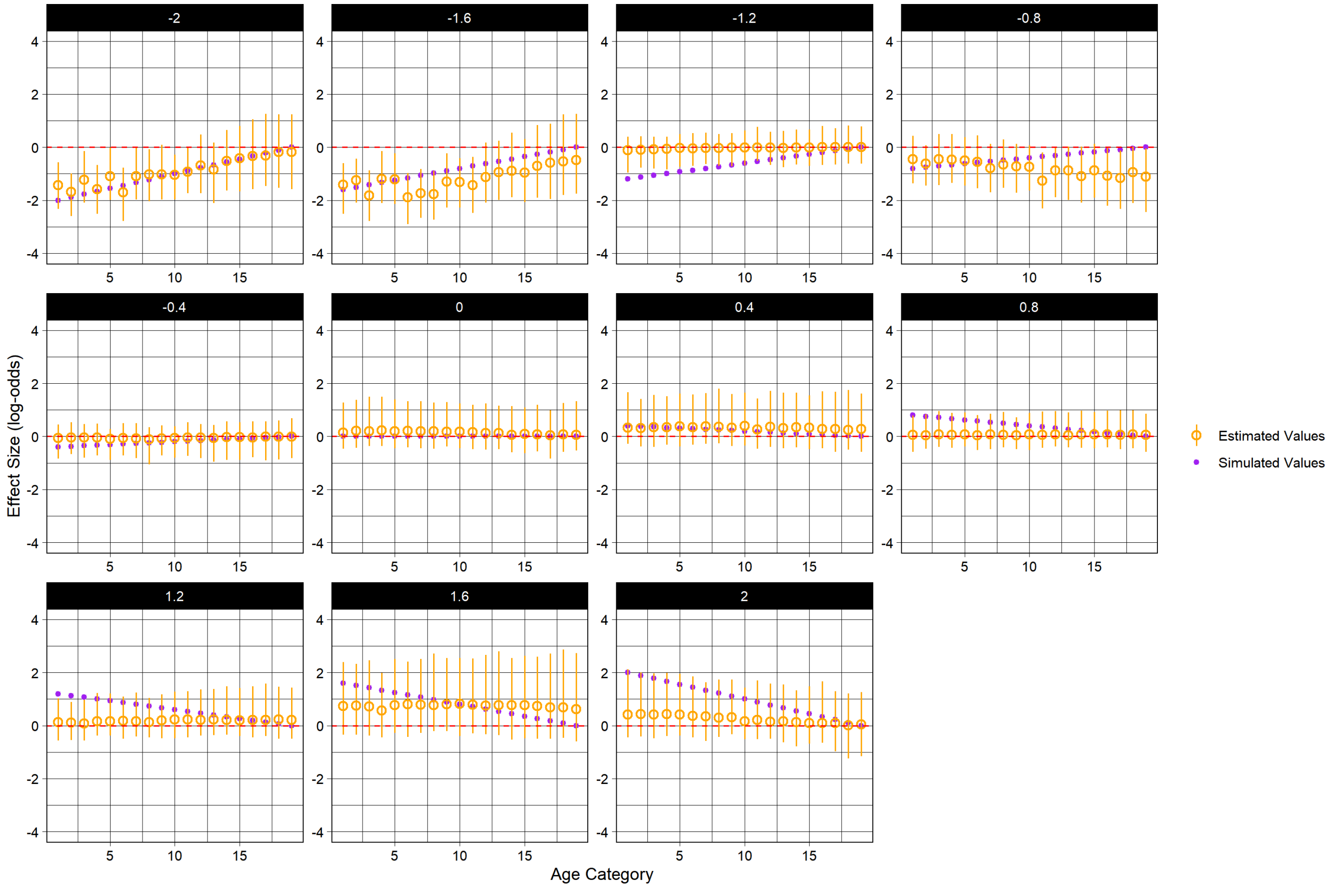

Fig. S58. Survival power analysis for the “father deceased” effect. Each panel shows the simulated (purple) and estimated (yellow) effect sizes at each age, for different ranges of simulated parameter values.

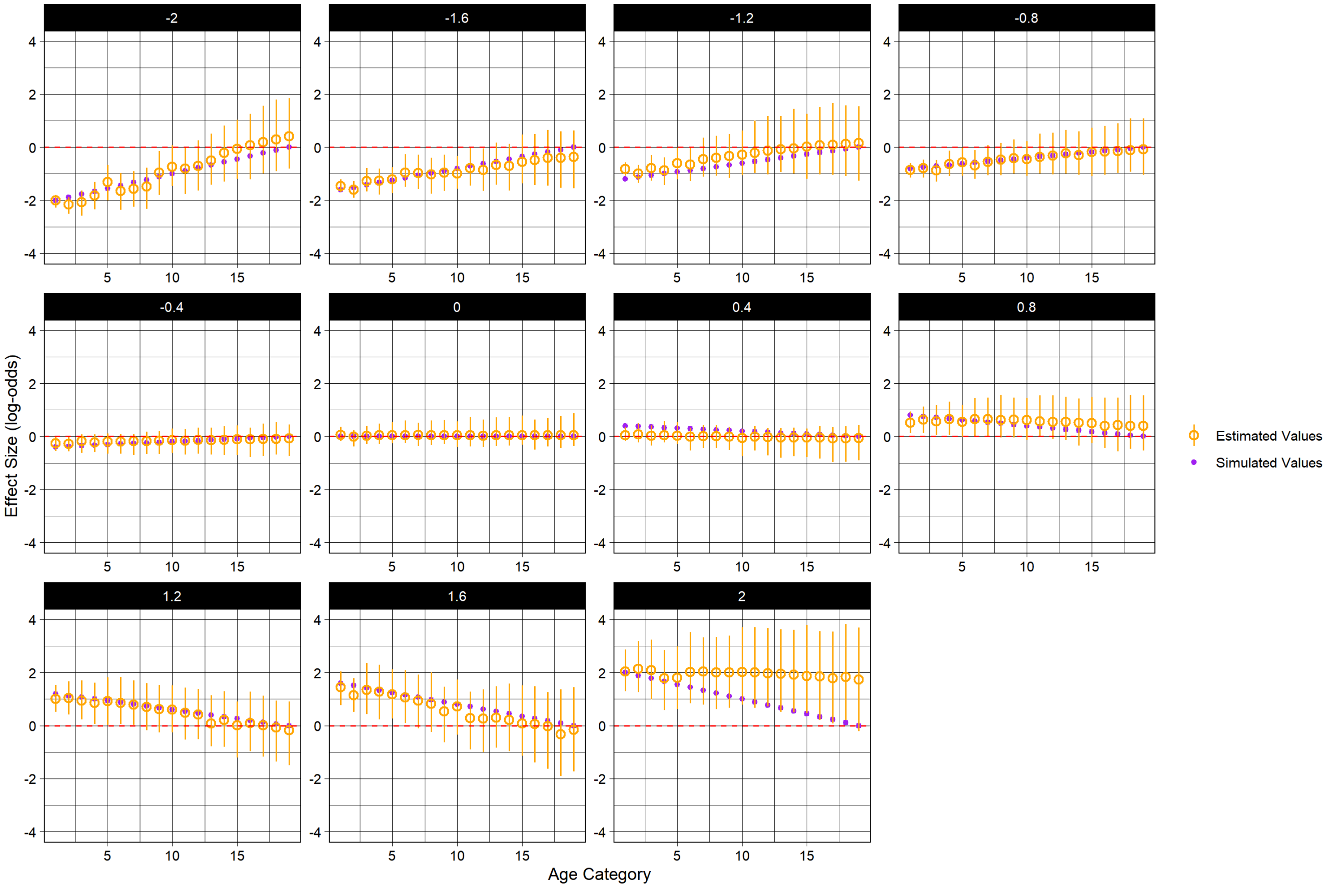

Fig. S59. Survival power analysis for the “father unmarried” effect. Each panel shows the simulated (purple) and estimated (yellow) effect sizes at each age, for different ranges of simulated parameter values.

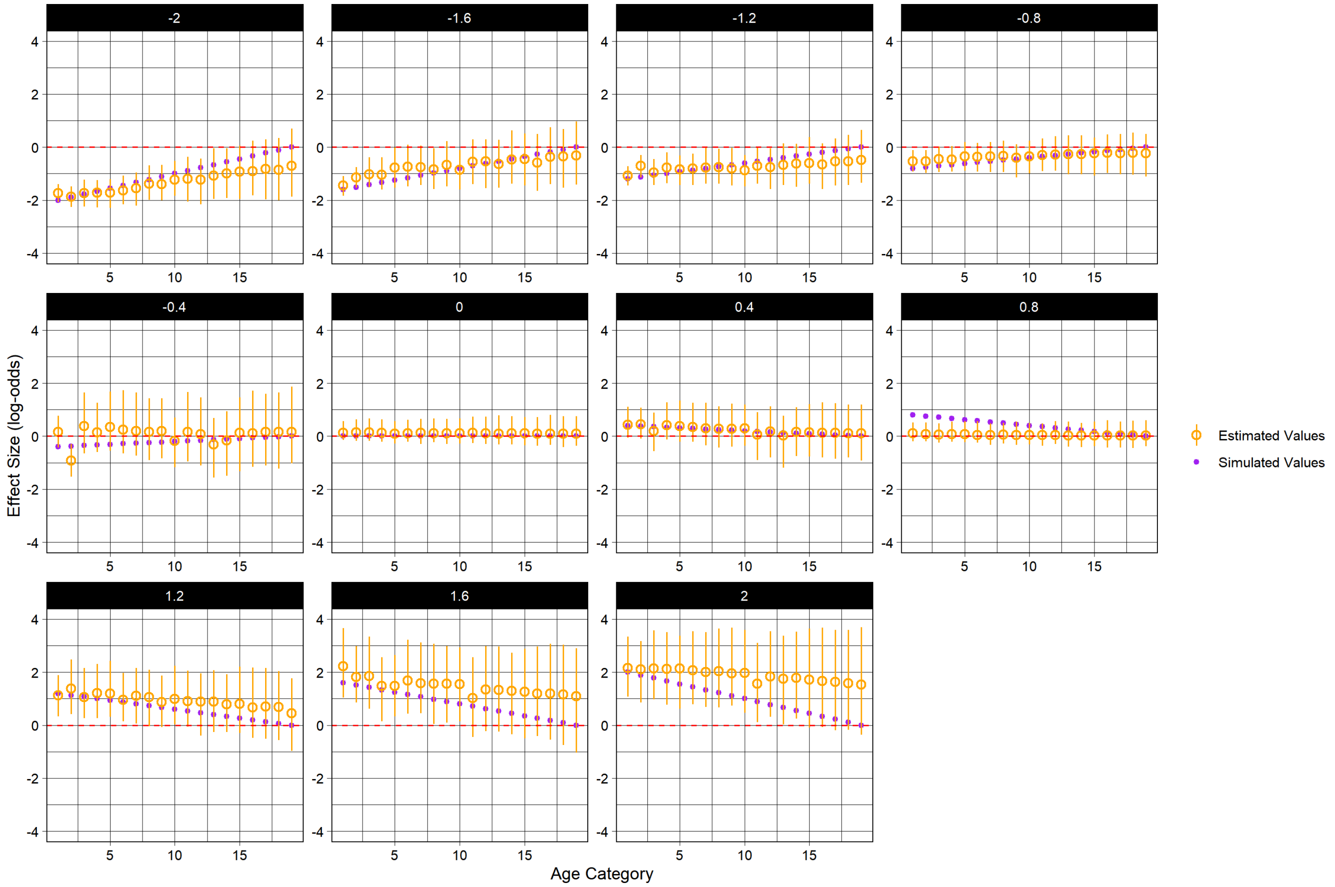

Fig. S60. Survival power analysis for the "father married to stepmother (monogamy)" effect. Each panel shows the simulated (purple) and estimated (yellow) effect sizes at each age, for different ranges of simulated parameter values.

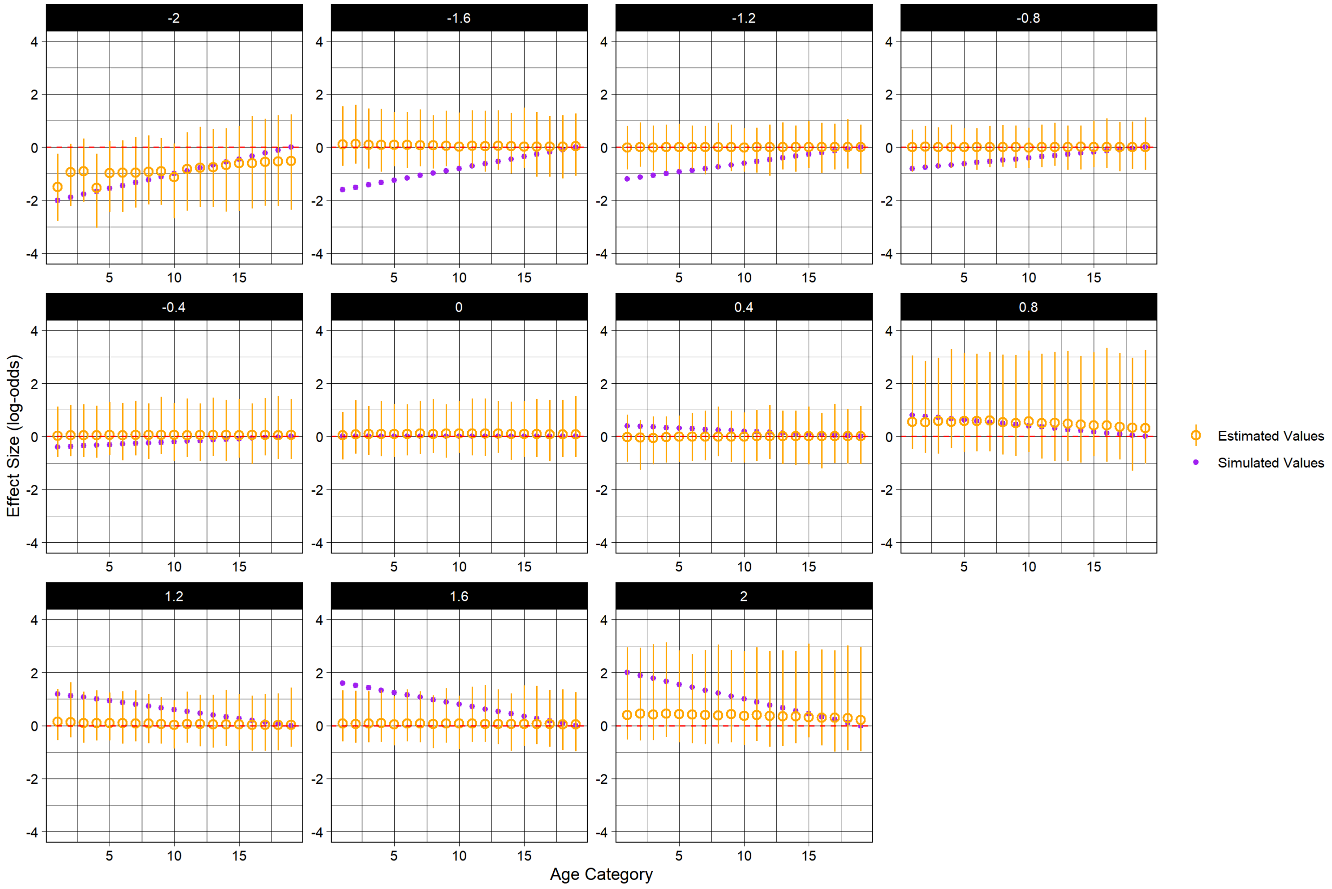

Fig. S61. Survival power analysis for the "father married to stepmother (polygyny)" effect. Each panel shows the simulated (purple) and estimated (yellow) effect sizes at each age, for different ranges of simulated parameter values.

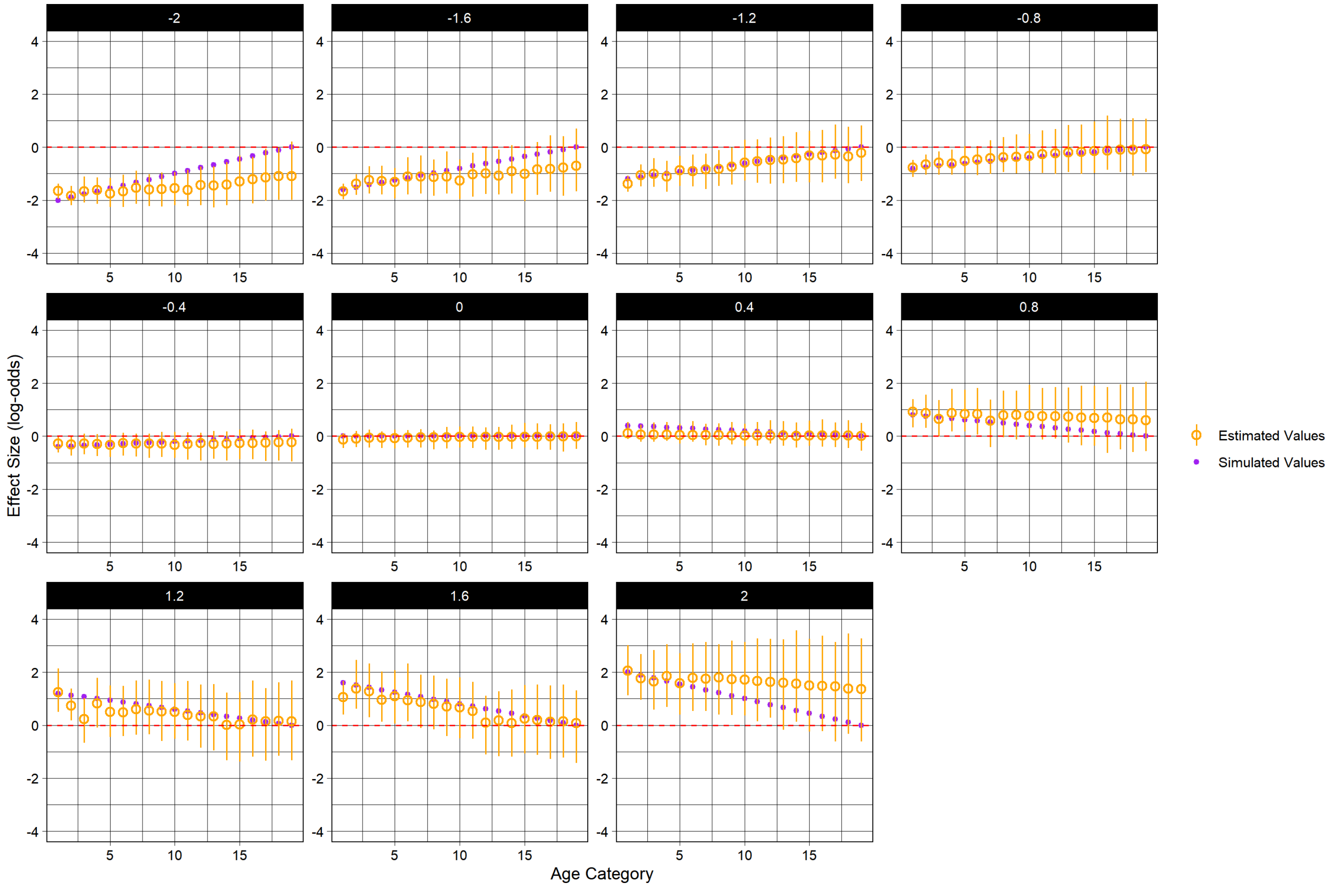

Fig. S62. Survival power analysis for the "father married to biological (polygyny)" effect. Each panel shows the simulated (purple) and estimated (yellow) effect sizes at each age, for different ranges of simulated parameter values.

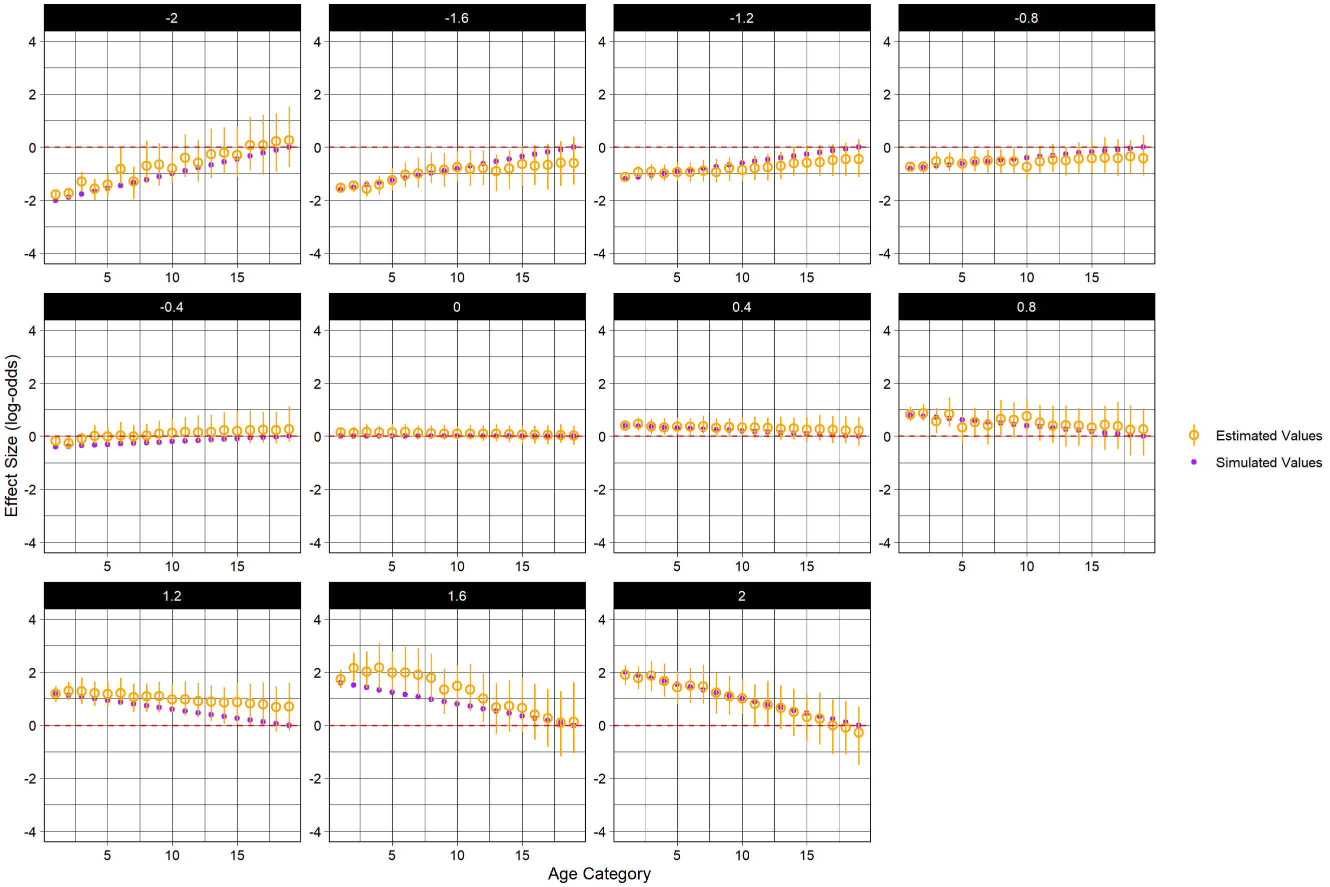

Fig. S63. Survival power analysis for the “either parent external” effect. Each panel shows the simulated (purple) and estimated (yellow) effect sizes at each age, for different ranges of simulated parameter values.

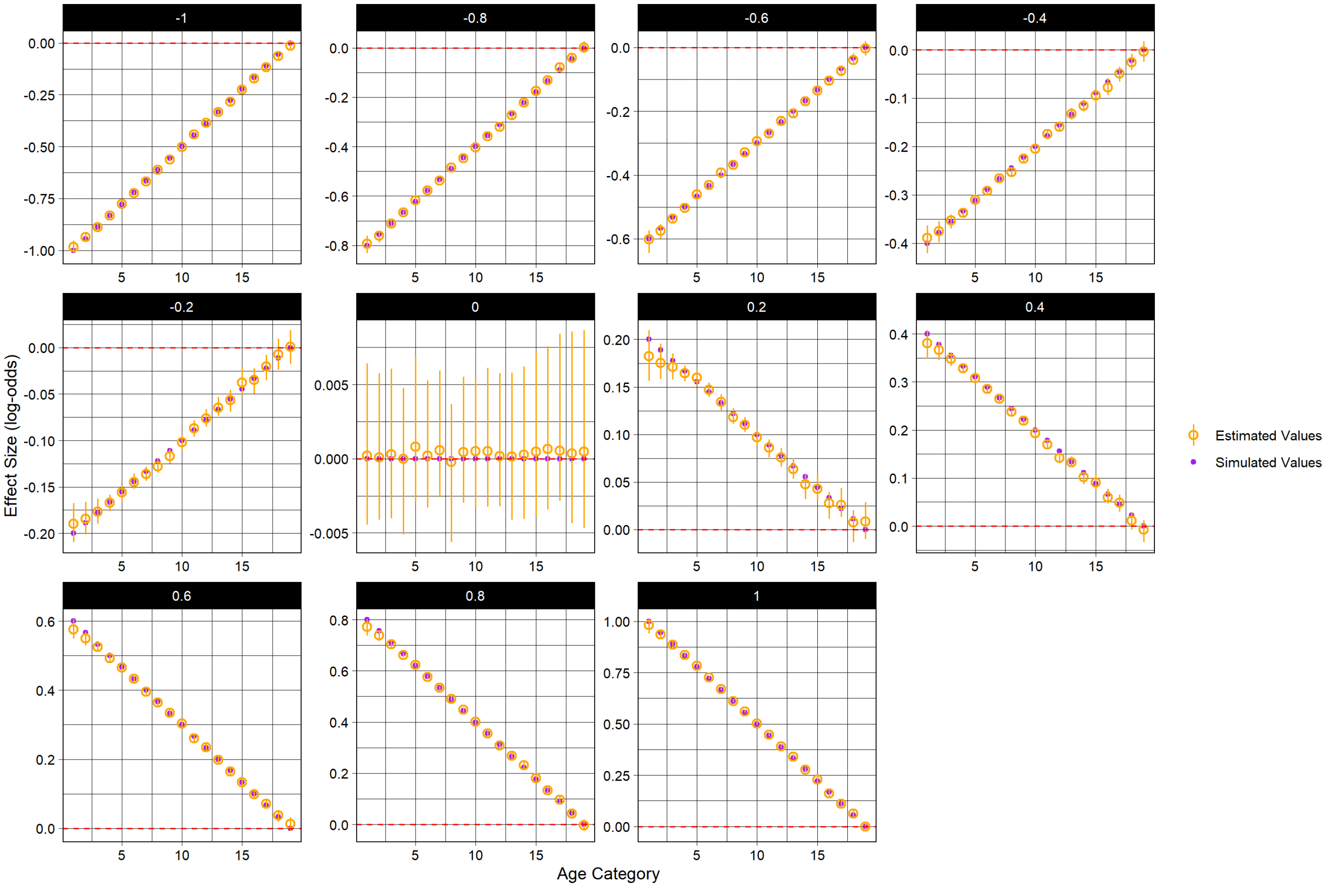

Fig. S64. Height power analysis for the "male" effect. Each panel shows the simulated (purple) and estimated (yellow) effect sizes at each age, for different ranges of simulated parameter values.

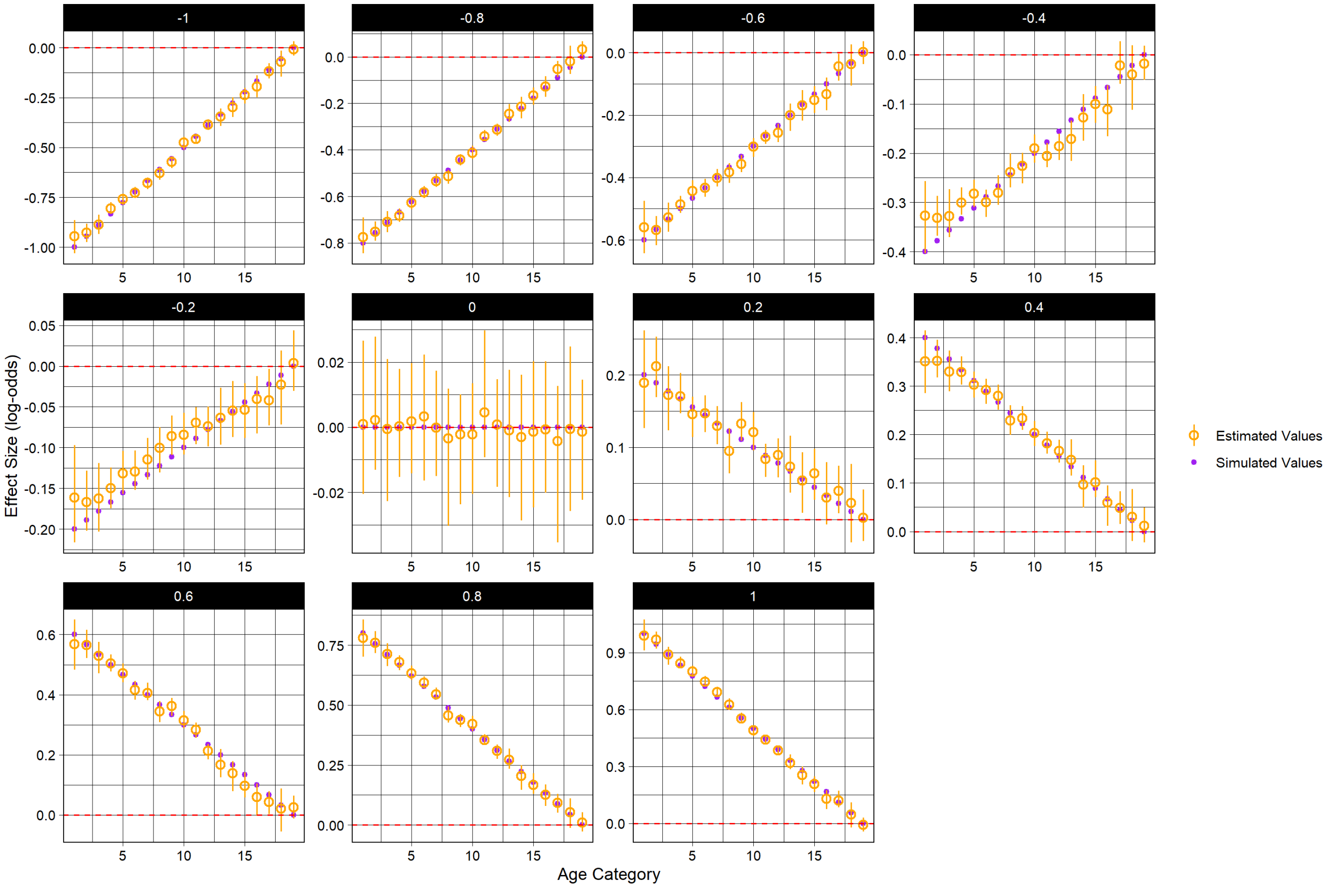

Fig. S65. Height power analysis for the "twin" effect. Each panel shows the simulated (purple) and estimated (yellow) effect sizes at each age, for different ranges of simulated parameter values.

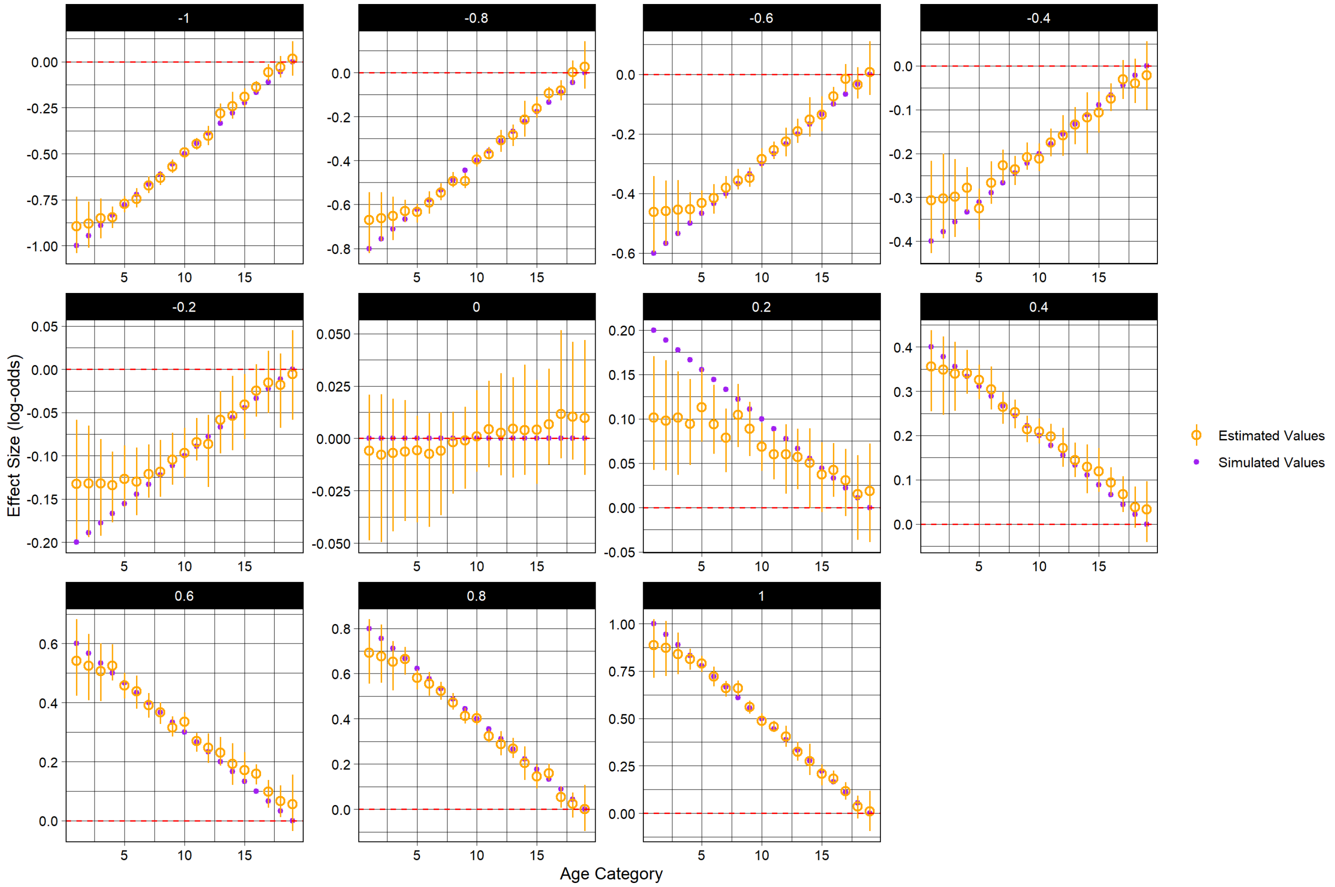

Fig. S66. Height power analysis for the “mother deceased” effect. Each panel shows the simulated (purple) and estimated (yellow) effect sizes at each age, for different ranges of simulated parameter values.

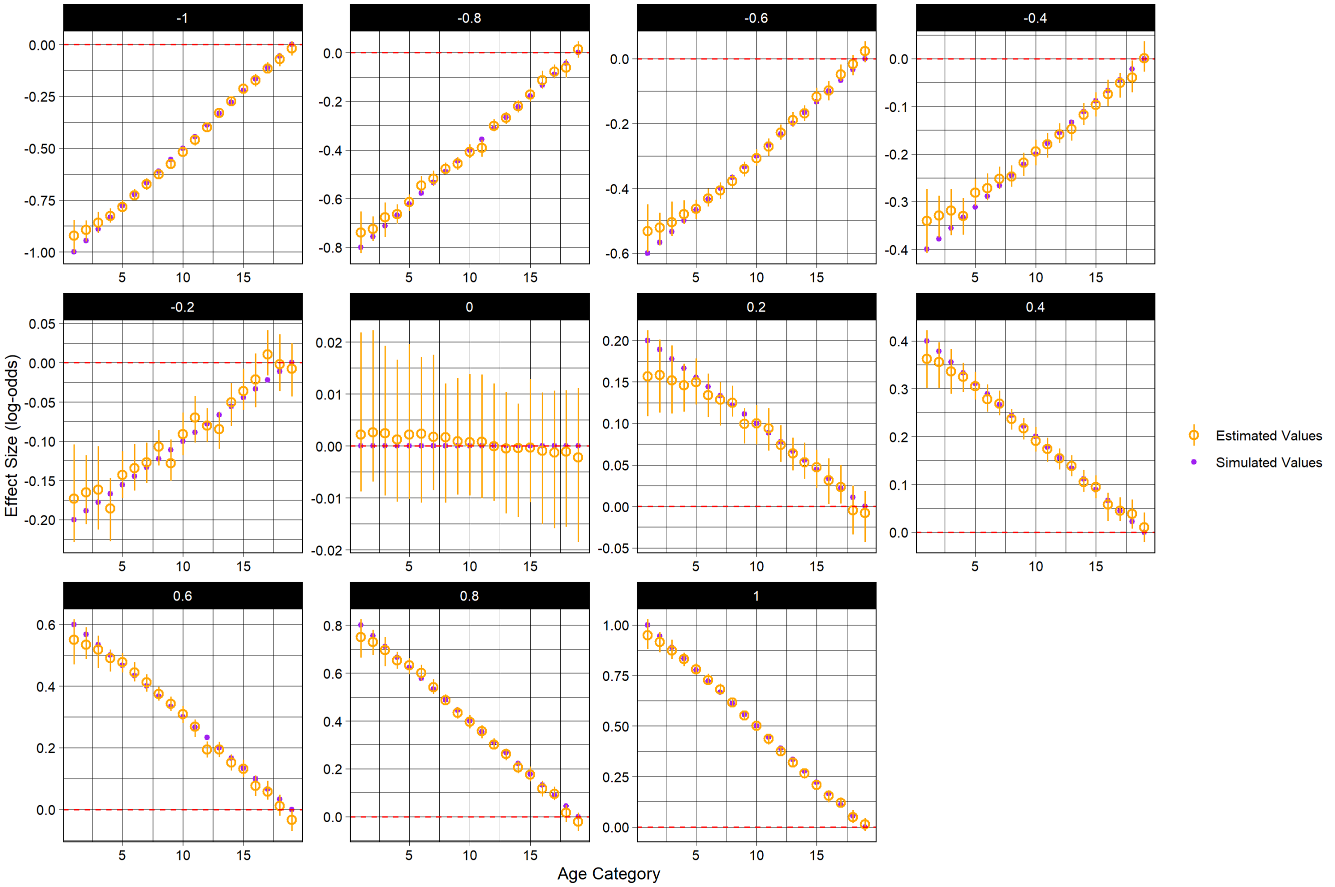

Fig. S67. Height power analysis for the "father deceased" effect. Each panel shows the simulated (purple) and estimated (yellow) effect sizes at each age, for different ranges of simulated parameter values.

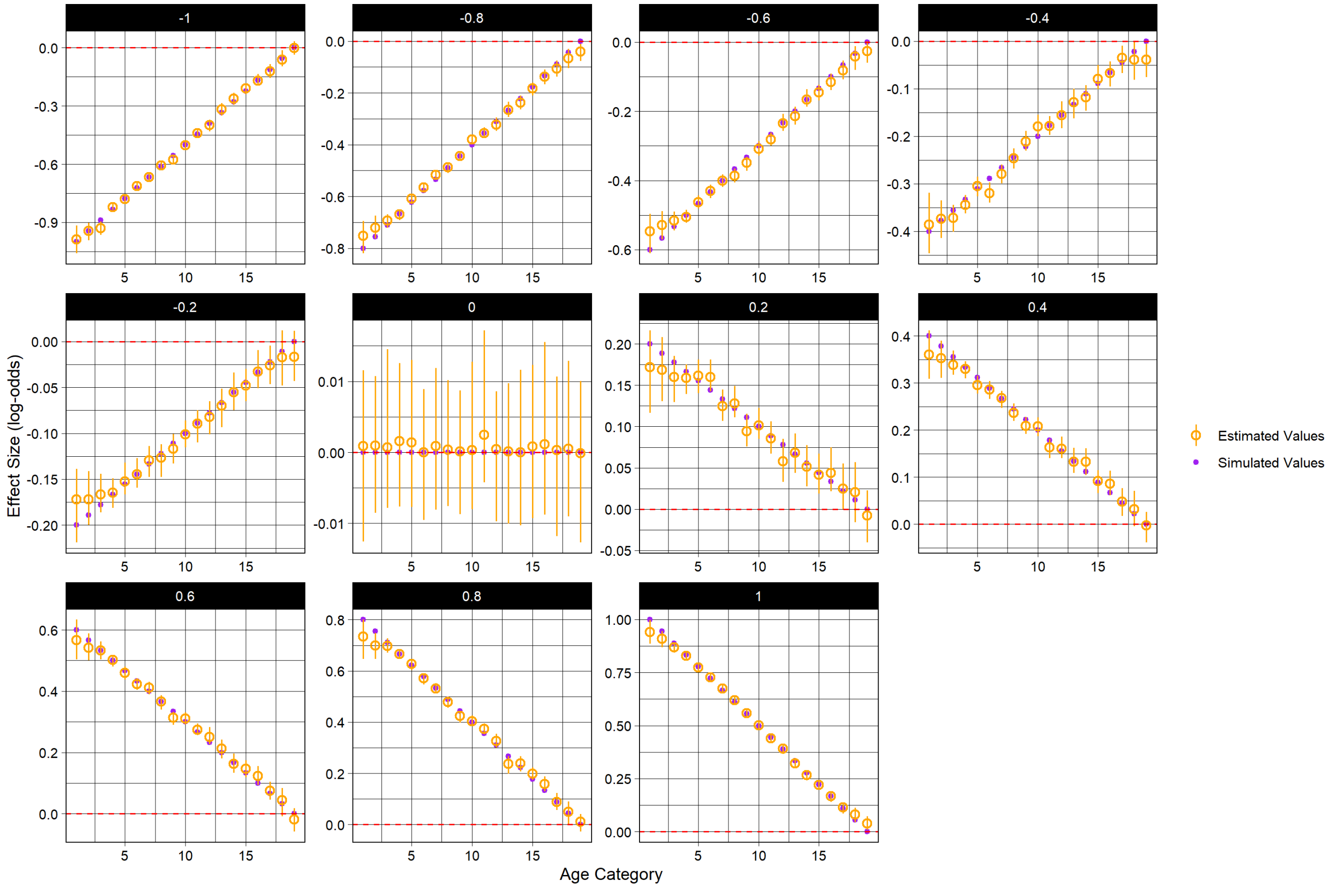

Fig. S68. Height power analysis for the “father unmarried” effect. Each panel shows the simulated (purple) and estimated (yellow) effect sizes at each age, for different ranges of simulated parameter values.

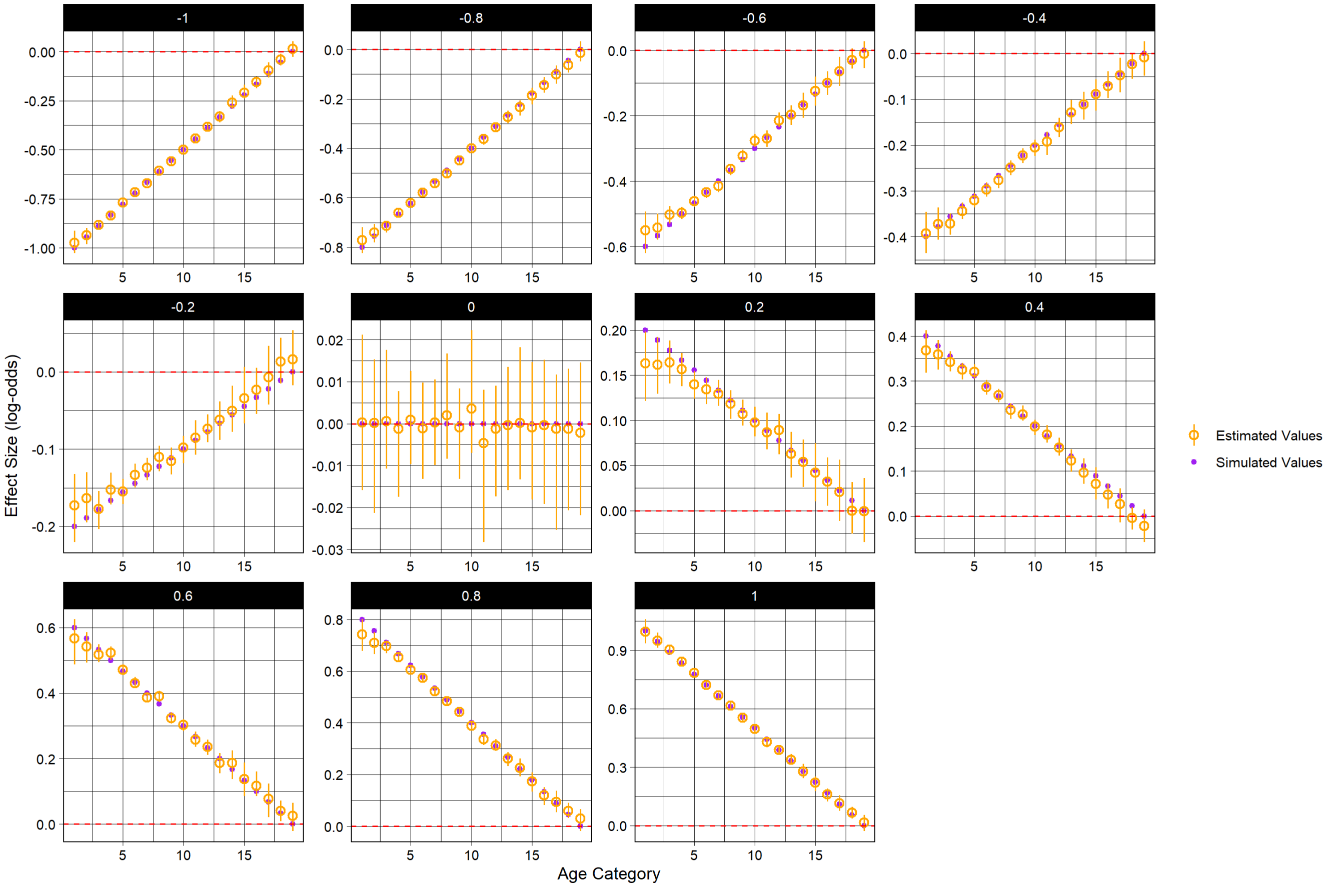

Fig. S69. Height power analysis for the “father married to stepmother (monogamy)” effect. Each panel shows the simulated (purple) and estimated (yellow) effect sizes at each age, for different ranges of simulated parameter values.

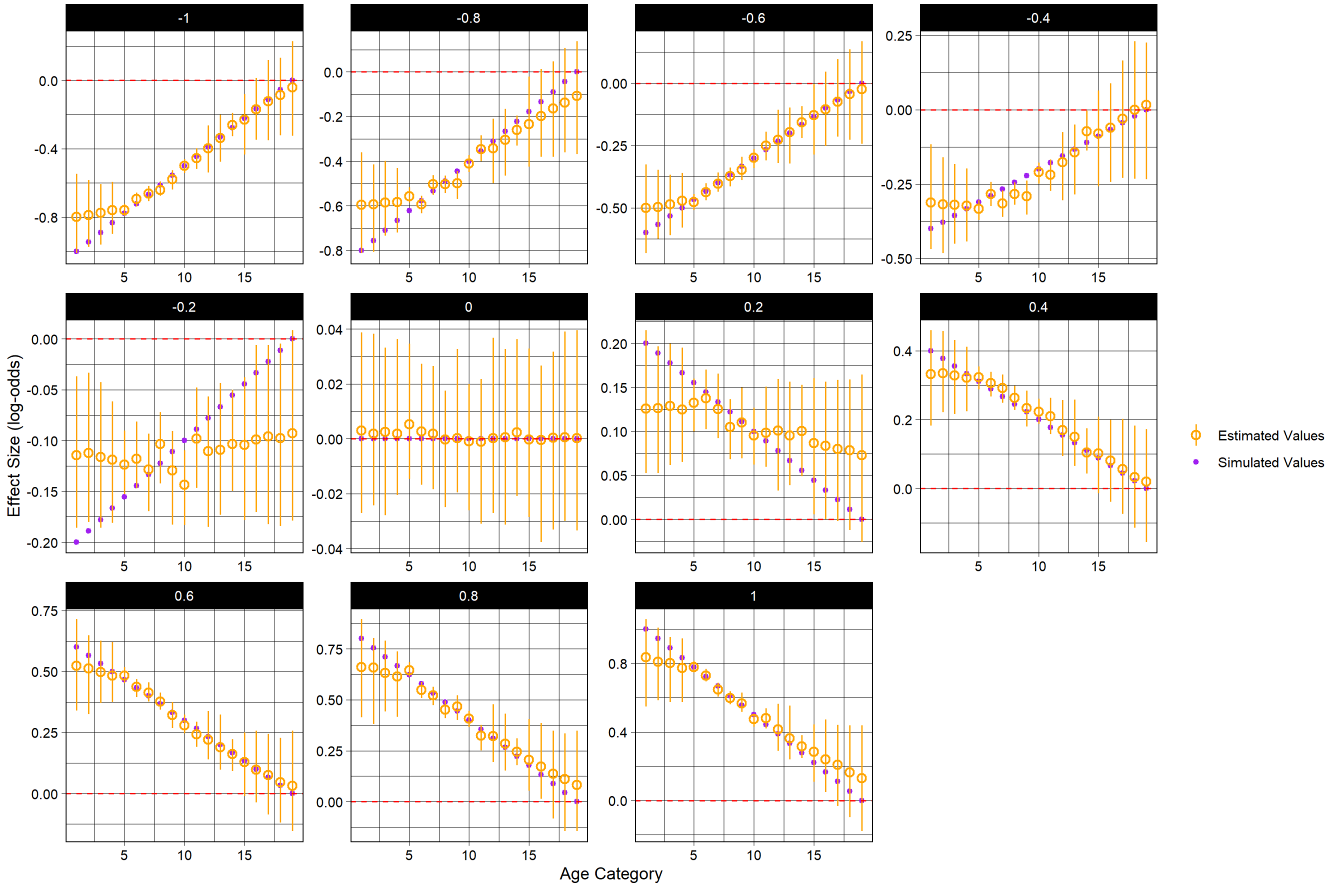

Fig. S70. Height power analysis for the "father married to stepmother (polygyny)" effect. Each panel shows the simulated (purple) and estimated (yellow) effect sizes at each age, for different ranges of simulated parameter values.

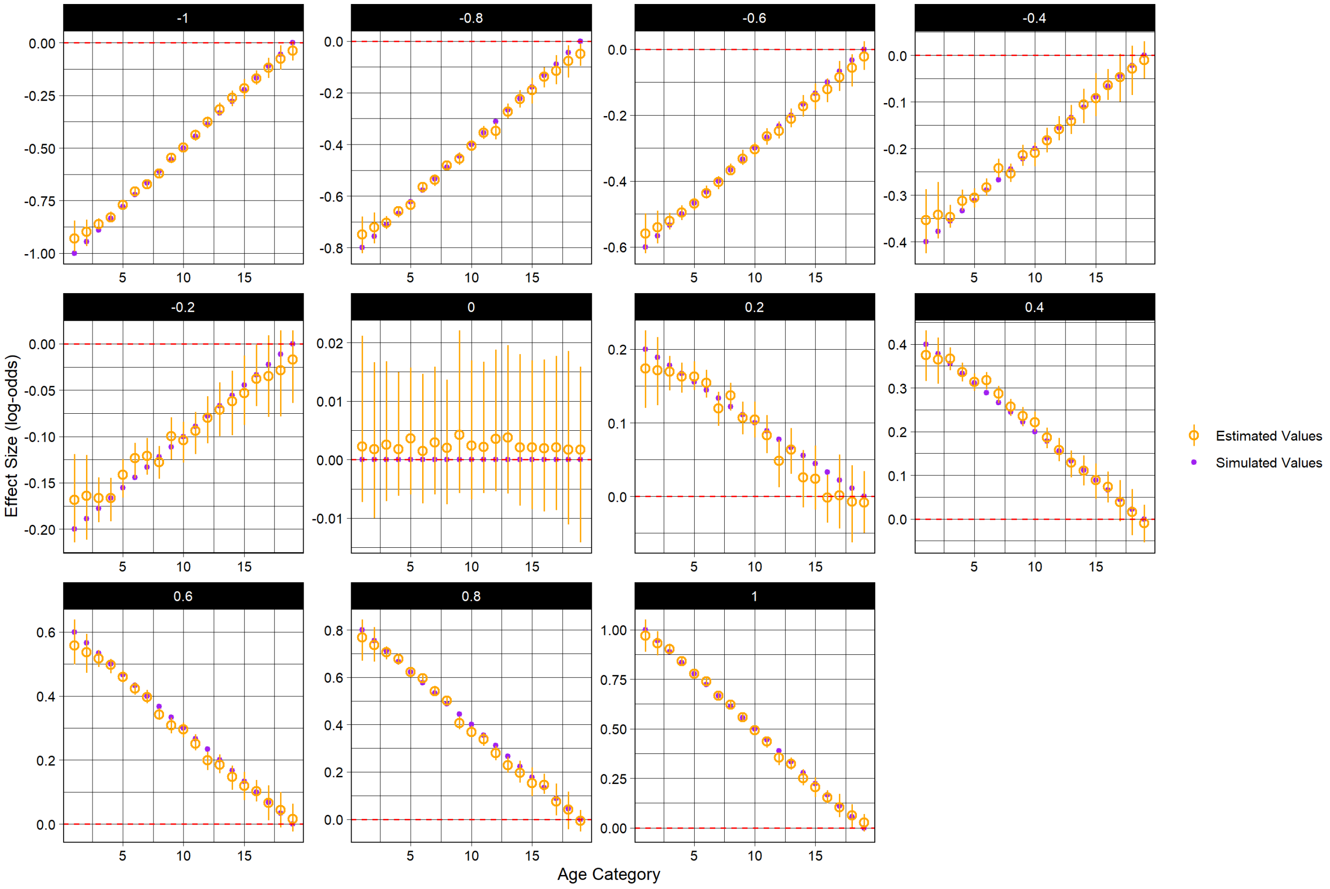

Fig. S71. Height power analysis for the “father married to biological (polygyny)” effect. Each panel shows the simulated (purple) and estimated (yellow) effect sizes at each age, for different ranges of simulated parameter values.

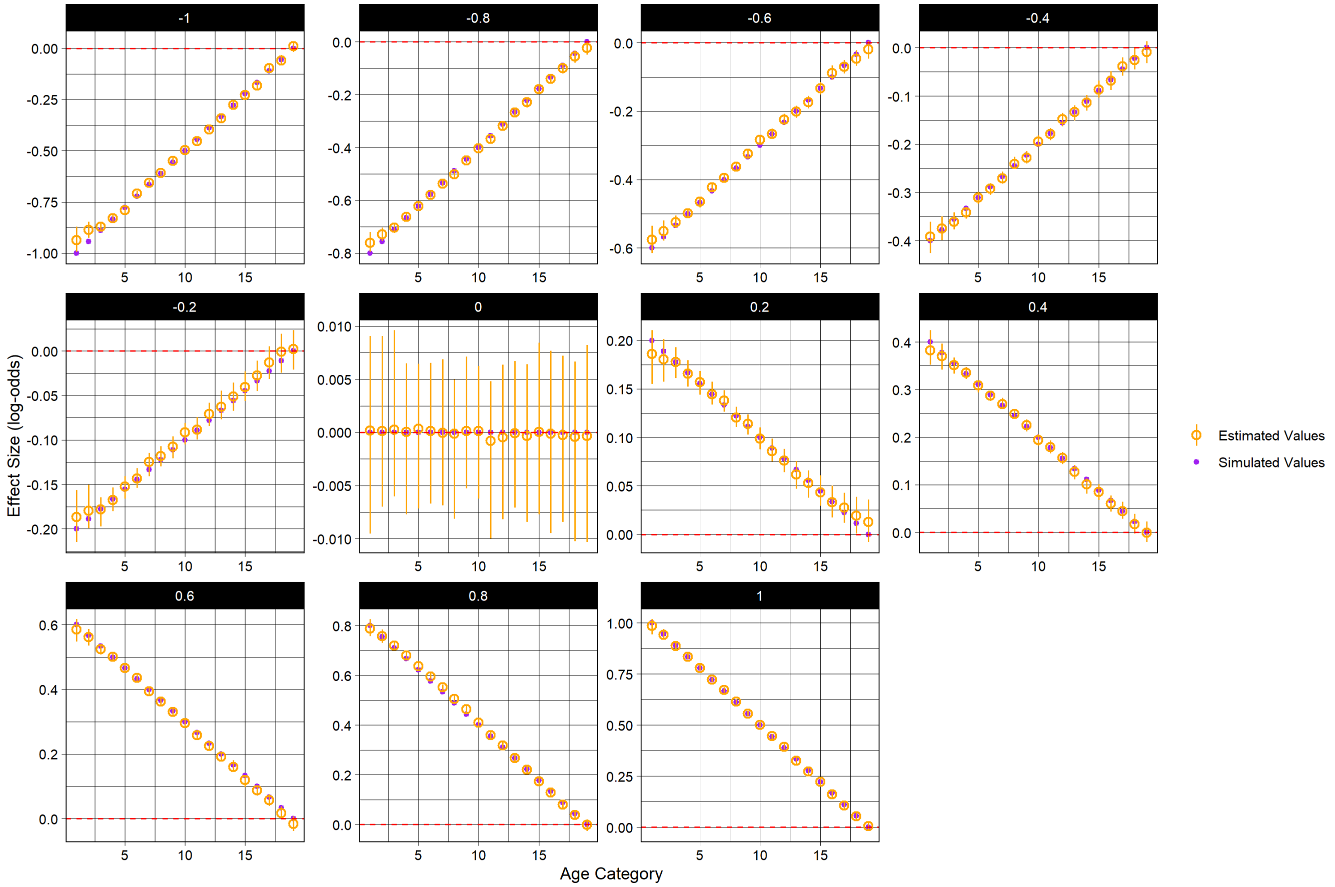

Fig. S72. Height power analysis for the “either parent external” effect. Each panel shows the simulated (purple) and estimated (yellow) effect sizes at each age, for different ranges of simulated parameter values.

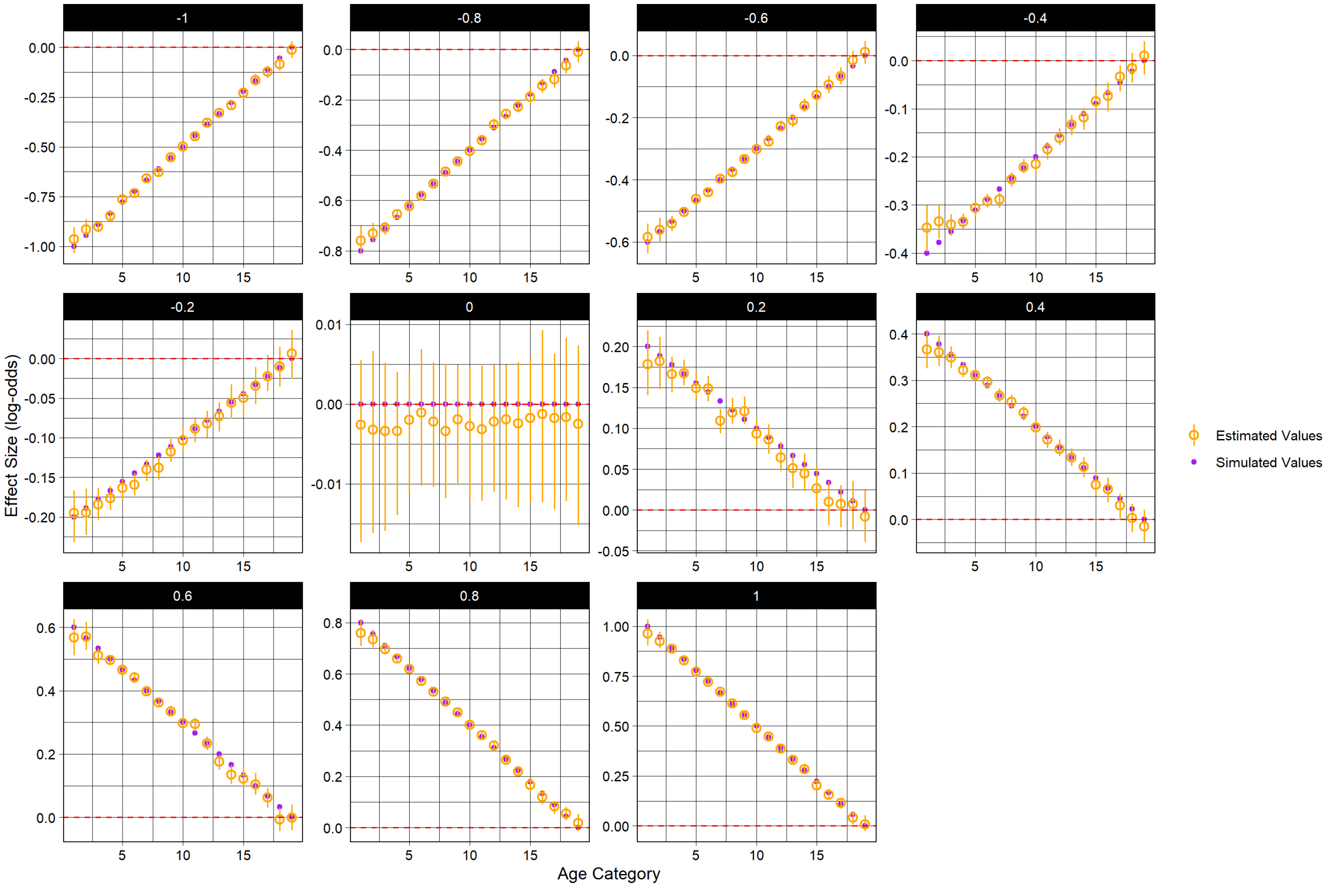

Fig. S73. Weight power analysis for the “male” effect. Each panel shows the simulated (purple) and estimated (yellow) effect sizes at each age, for different ranges of simulated parameter values.

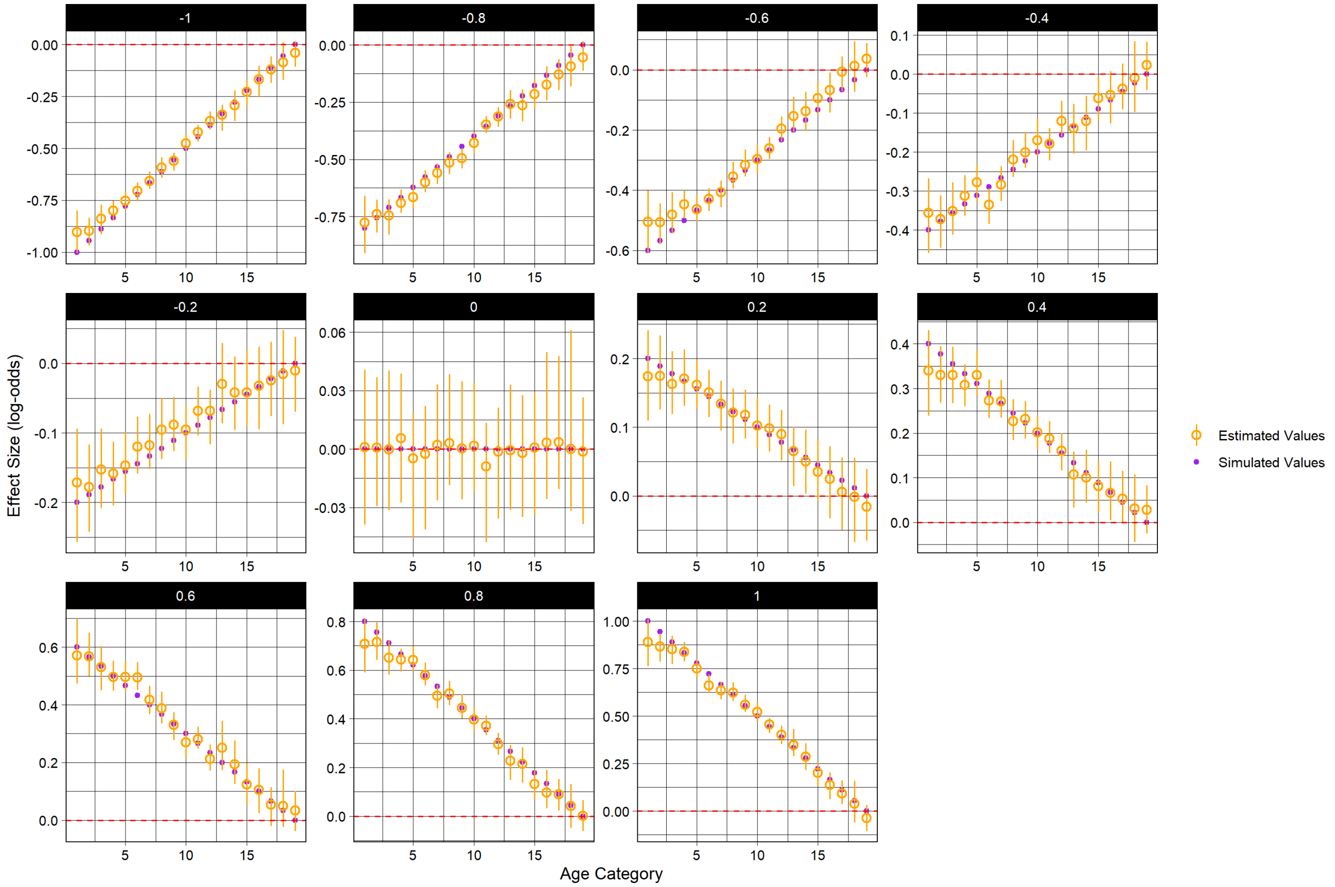

Fig. S74. Weight power analysis for the “twin” effect. Each panel shows the simulated (purple) and estimated (yellow) effect sizes at each age, for different ranges of simulated parameter values.

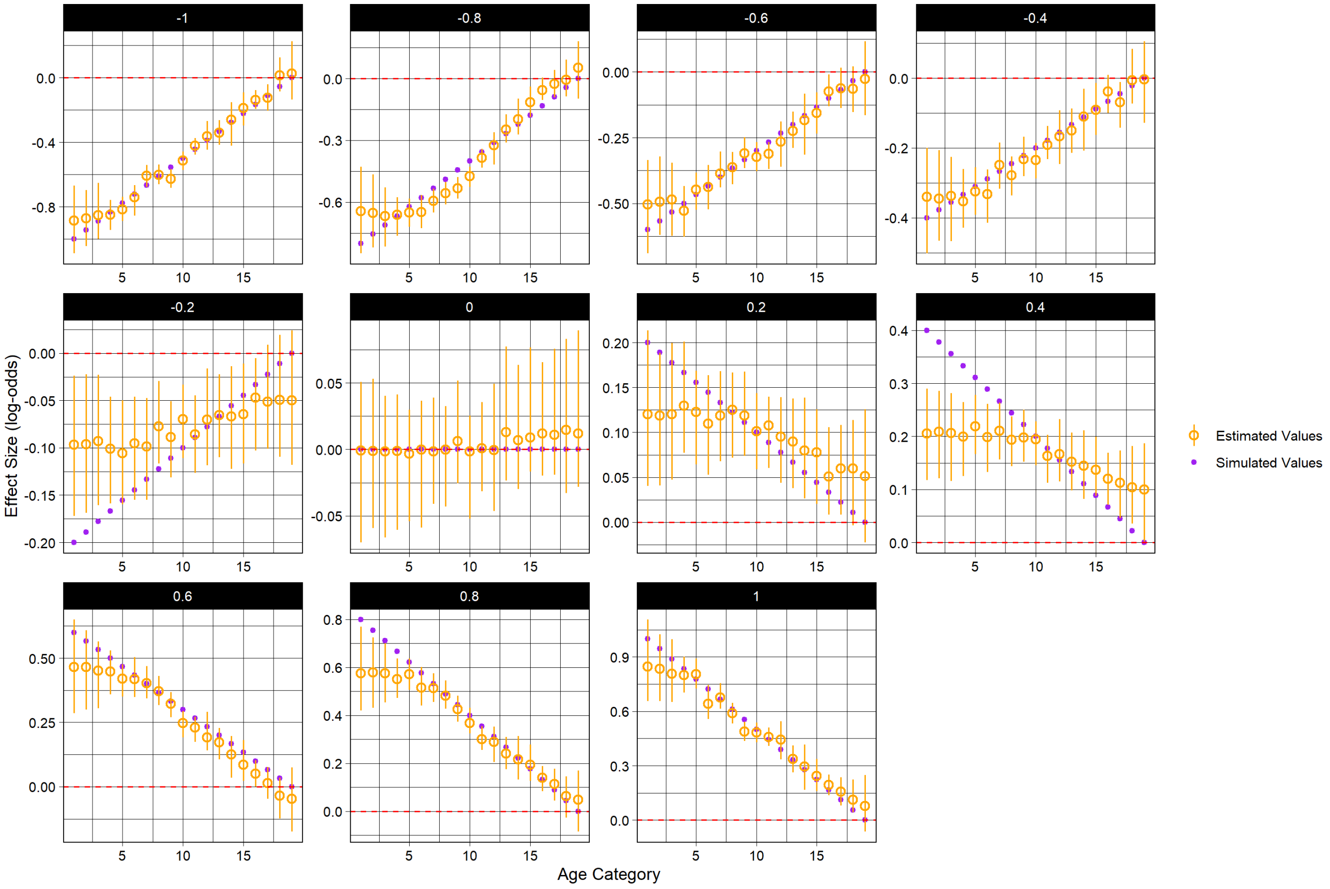

Fig. S75. Weight power analysis for the “mother deceased” effect. Each panel shows the simulated (purple) and estimated (yellow) effect sizes at each age, for different ranges of simulated parameter values.

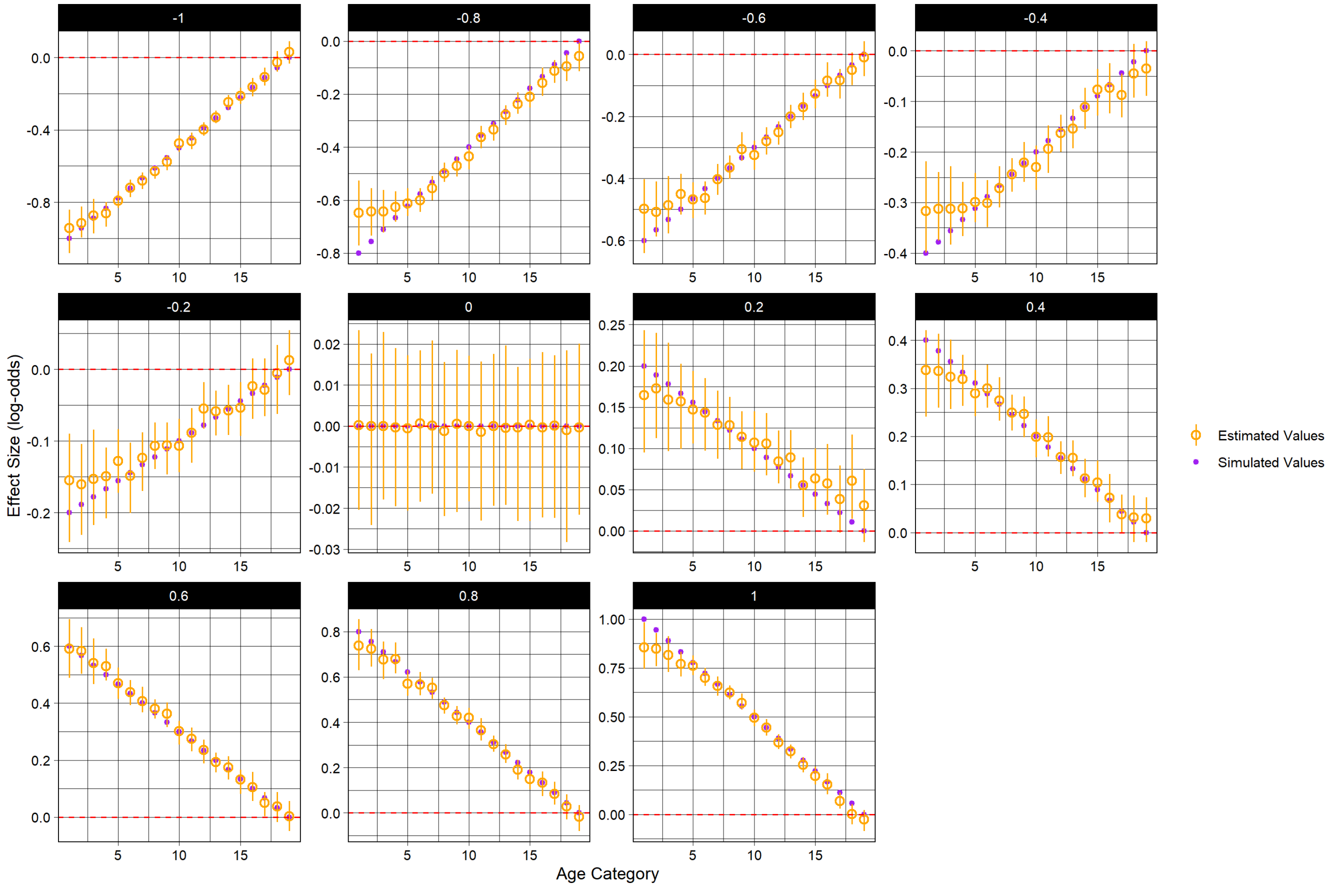

Fig. S76. Weight power analysis for the “father deceased” effect. Each panel shows the simulated (purple) and estimated (yellow) effect sizes at each age, for different ranges of simulated parameter values.

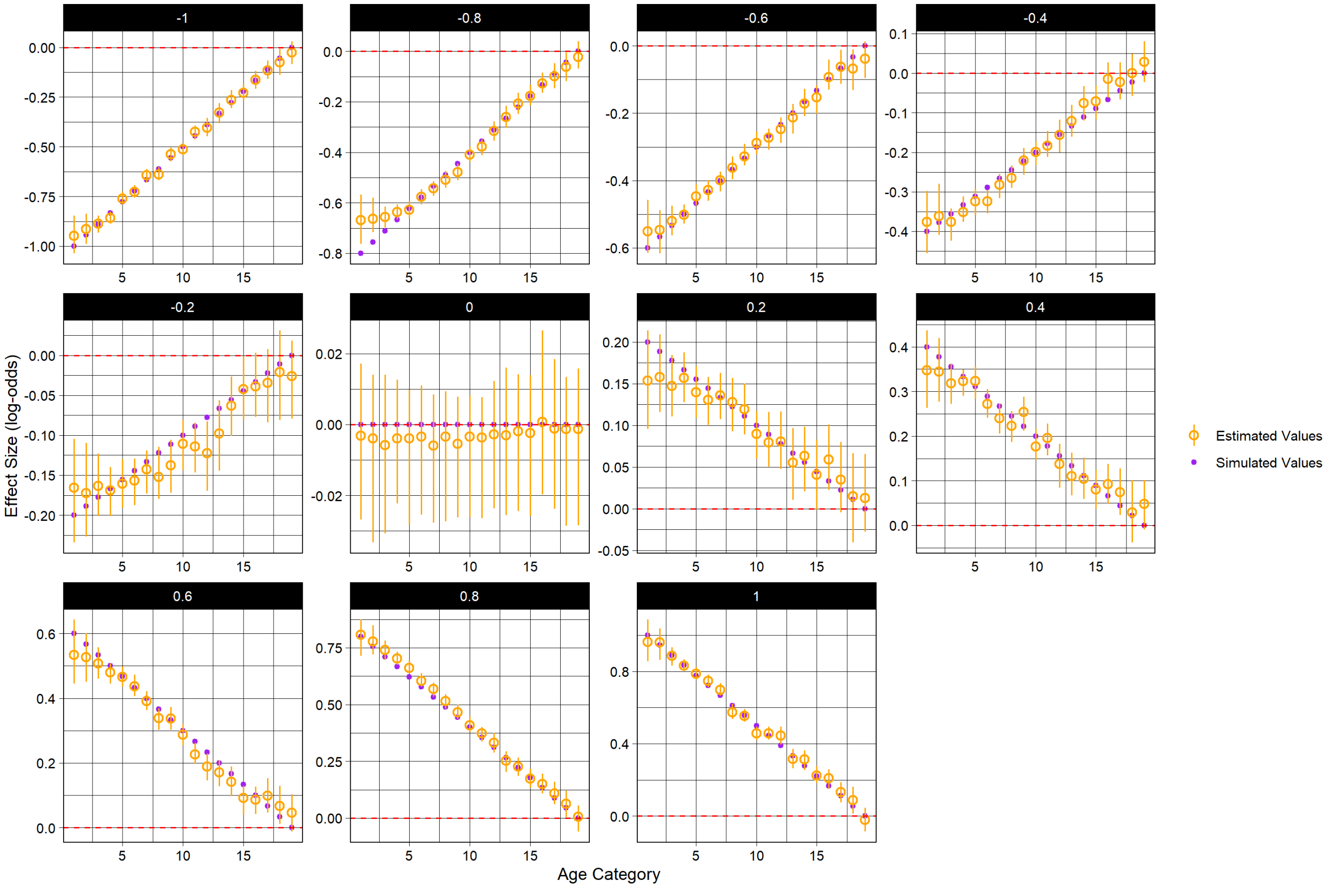

Fig. S77. Weight power analysis for the “father unmarried” effect. Each panel shows the simulated (purple) and estimated (yellow) effect sizes at each age, for different ranges of simulated parameter values.

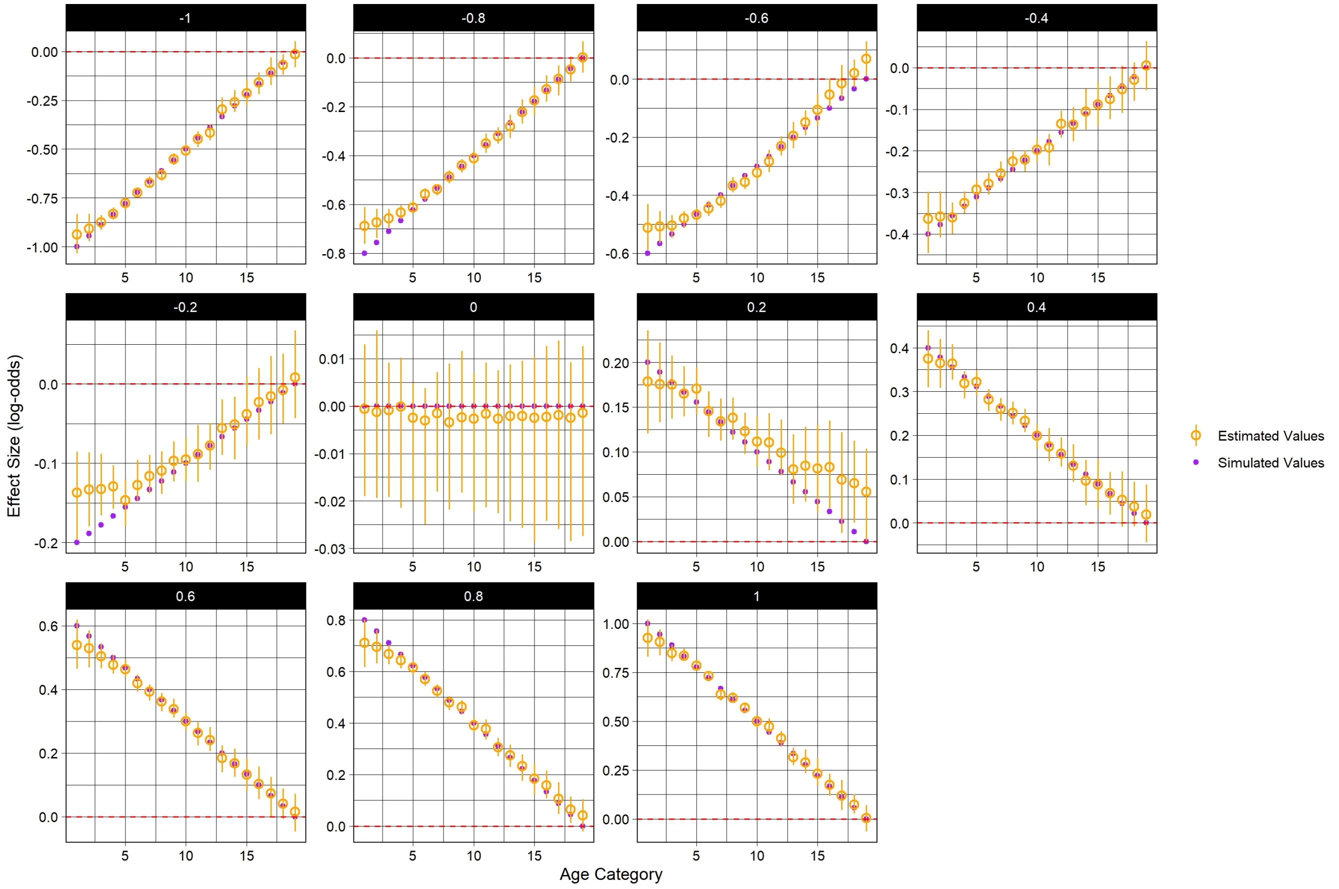

Fig. S78. Weight power analysis for the "father married to stepmother (monogamy)" effect. Each panel shows the simulated (purple) and estimated (yellow) effect sizes at each age, for different ranges of simulated parameter values.

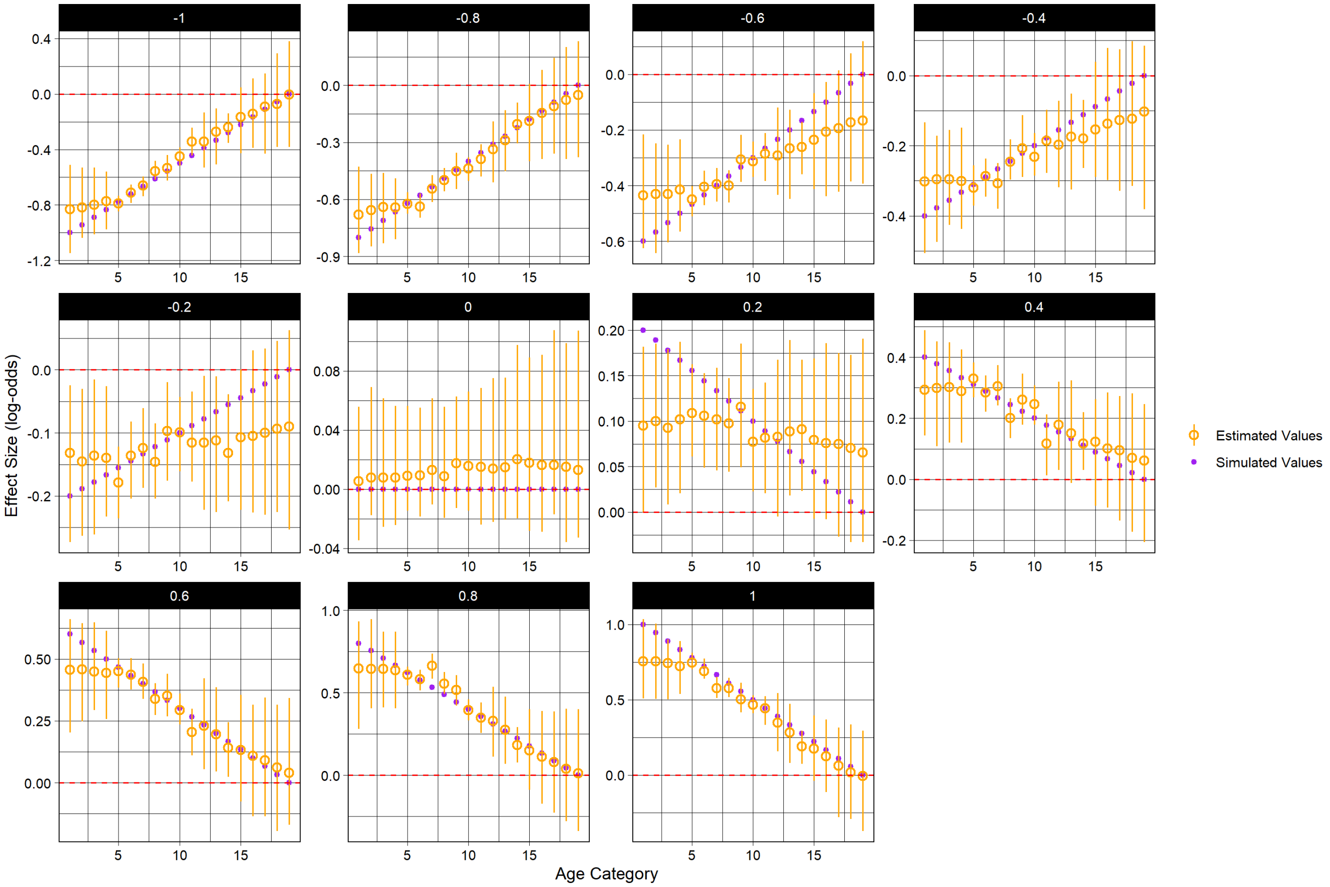

Fig. S79. Weight power analysis for the "father married to stepmother (polygyny)" effect. Each panel shows the simulated (purple) and estimated (yellow) effect sizes at each age, for different ranges of simulated parameter values.

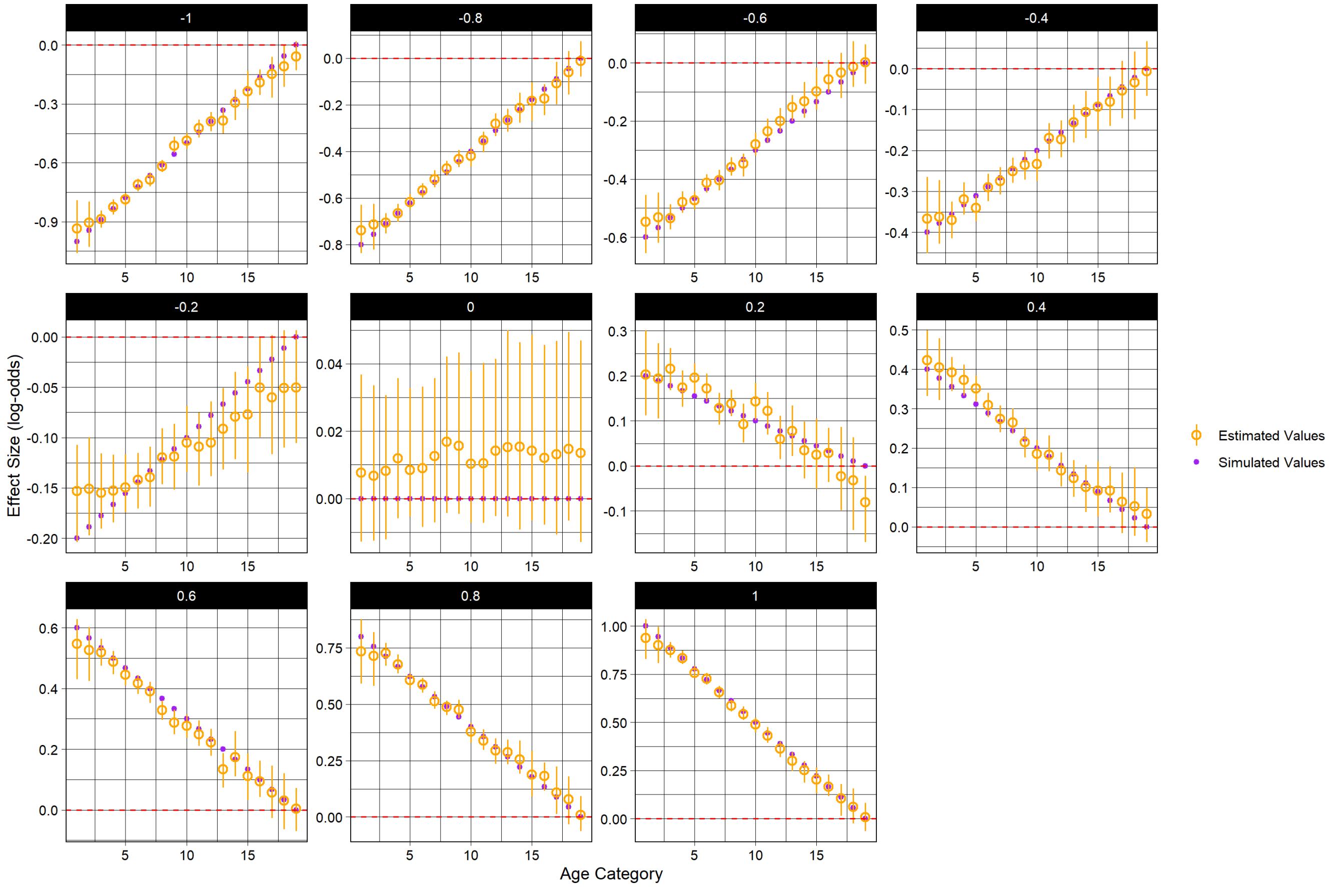

Fig. S80. Weight power analysis for the "father married to biological (polygyny)" effect. Each panel shows the simulated (purple) and estimated (yellow) effect sizes at each age, for different ranges of simulated parameter values.

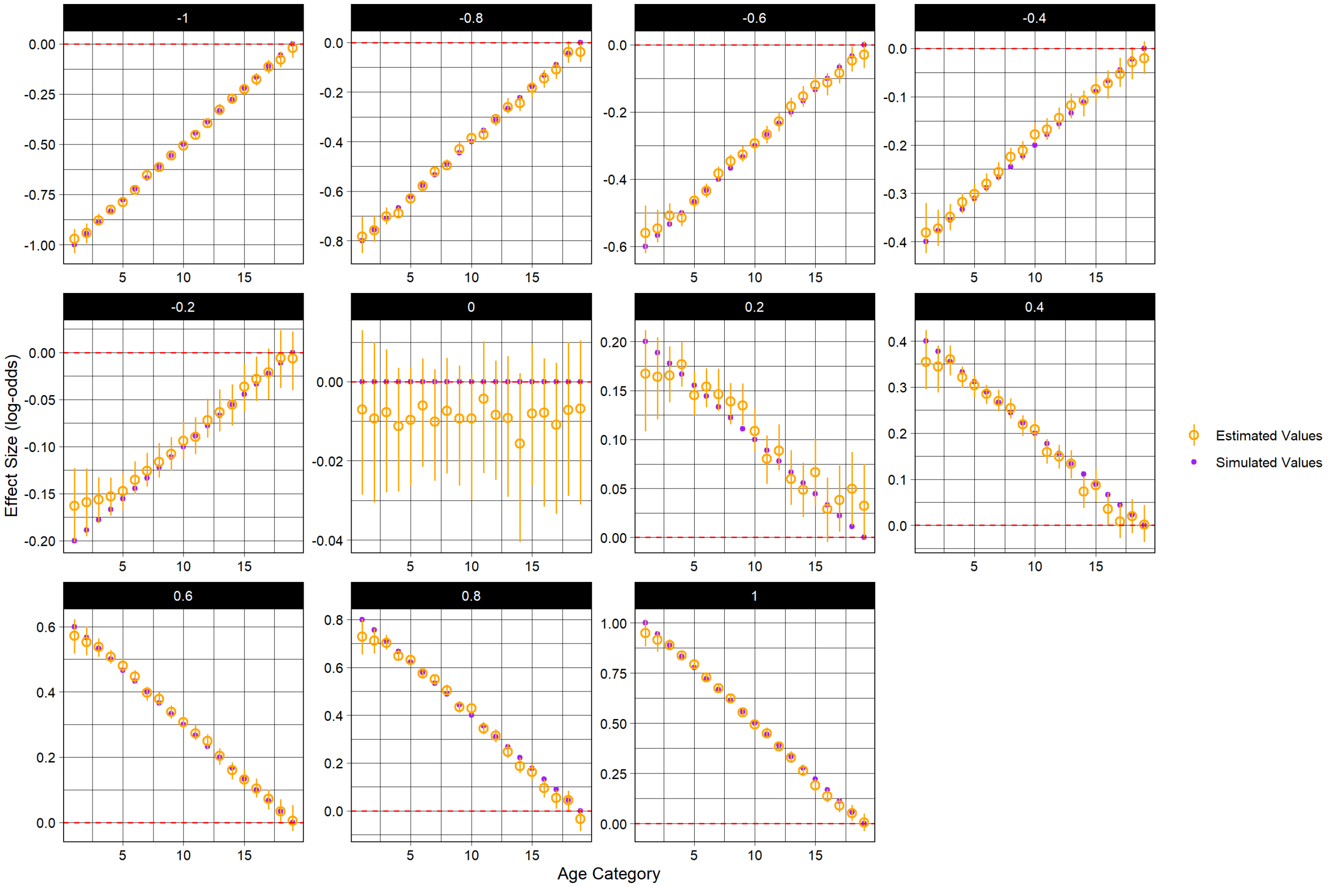

Fig. S81. Weight power analysis for the "either parent external" effect. Each panel shows the simulated (purple) and estimated (yellow) effect sizes at each age, for different ranges of simulated parameter values.

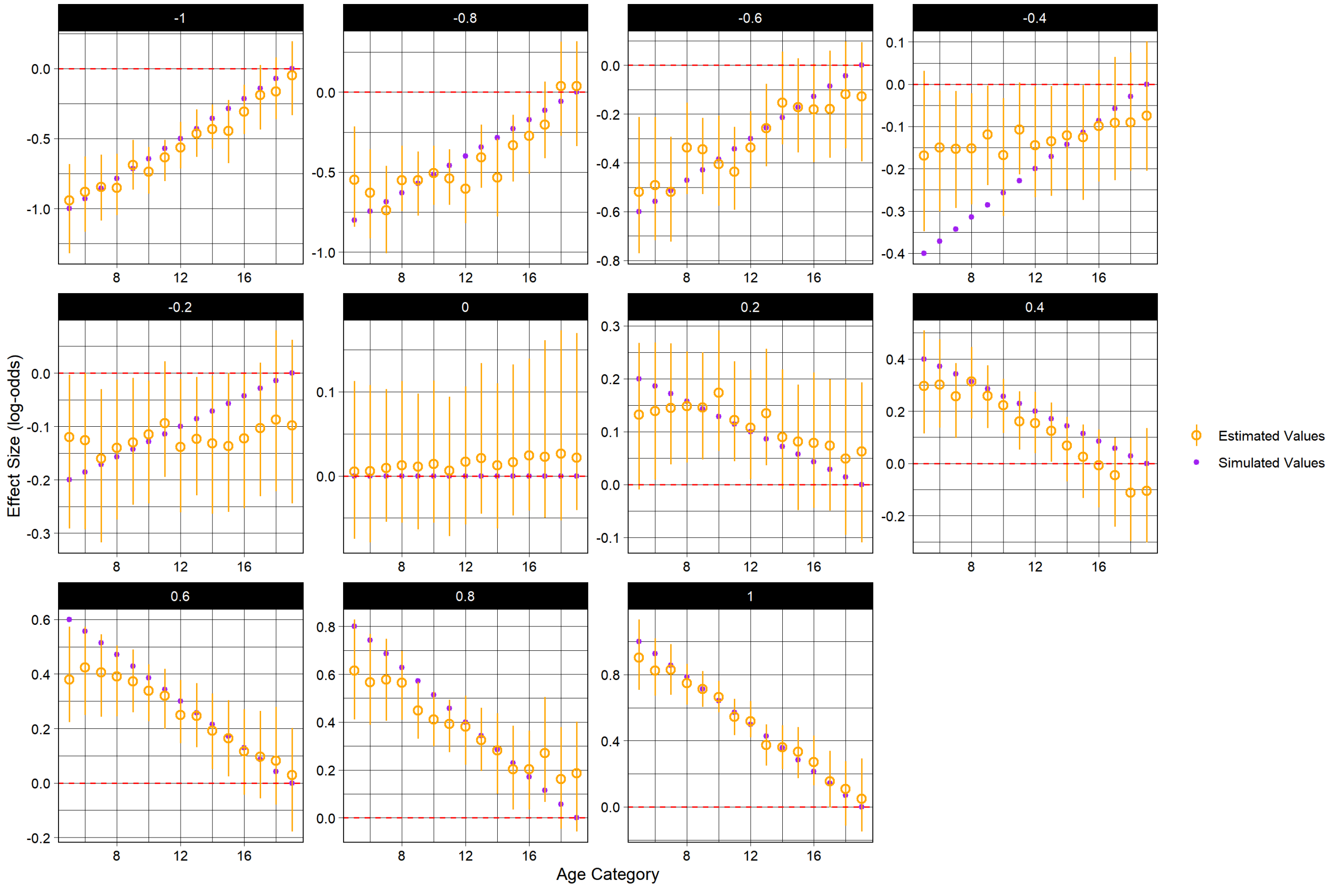

Fig. S82. Education power analysis for the "male" effect. Each panel shows the simulated (purple) and estimated (yellow) effect sizes at each age, for different ranges of simulated parameter values.

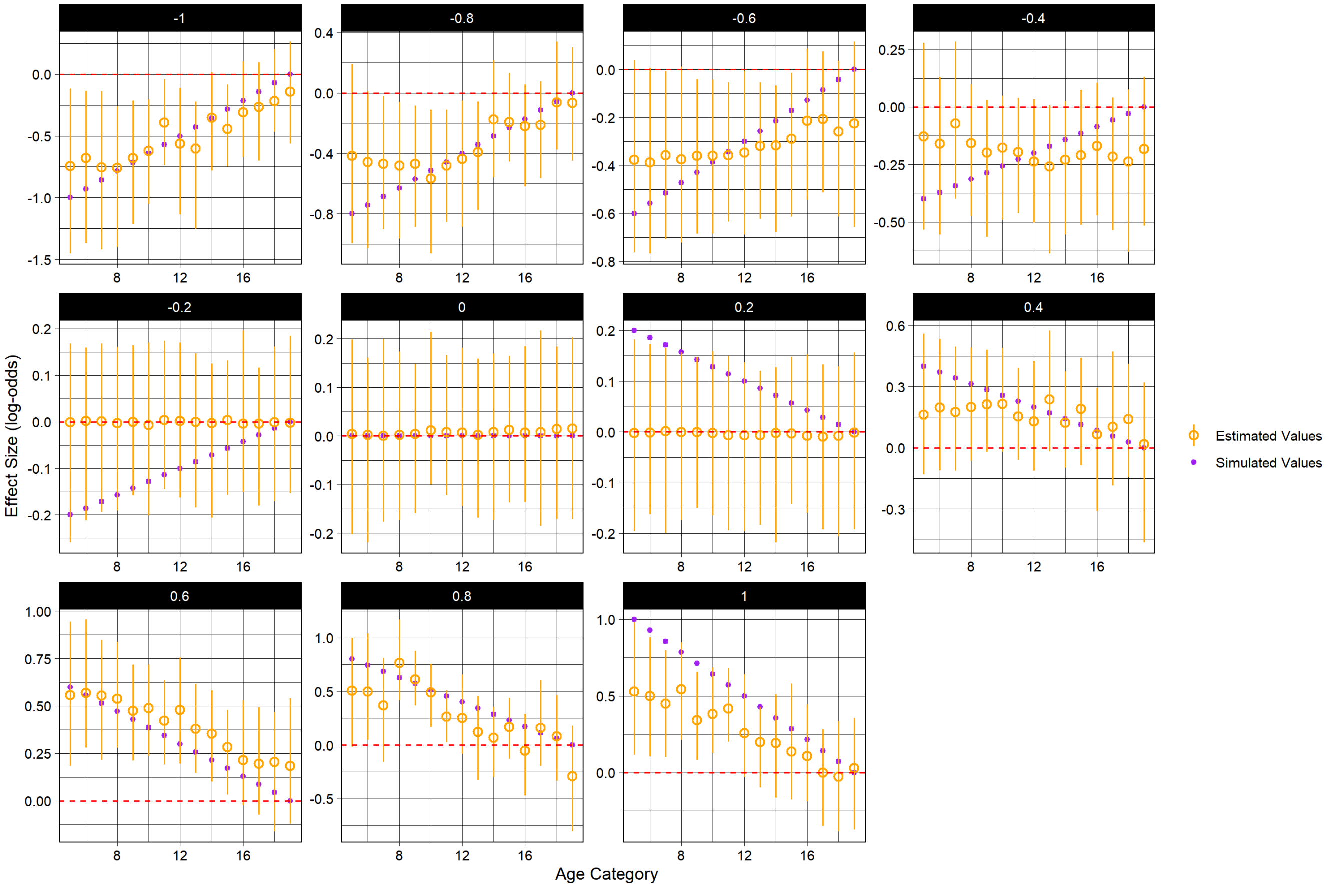

Fig. S83. Education power analysis for the "twin" effect. Each panel shows the simulated (purple) and estimated (yellow) effect sizes at each age, for different ranges of simulated parameter values.

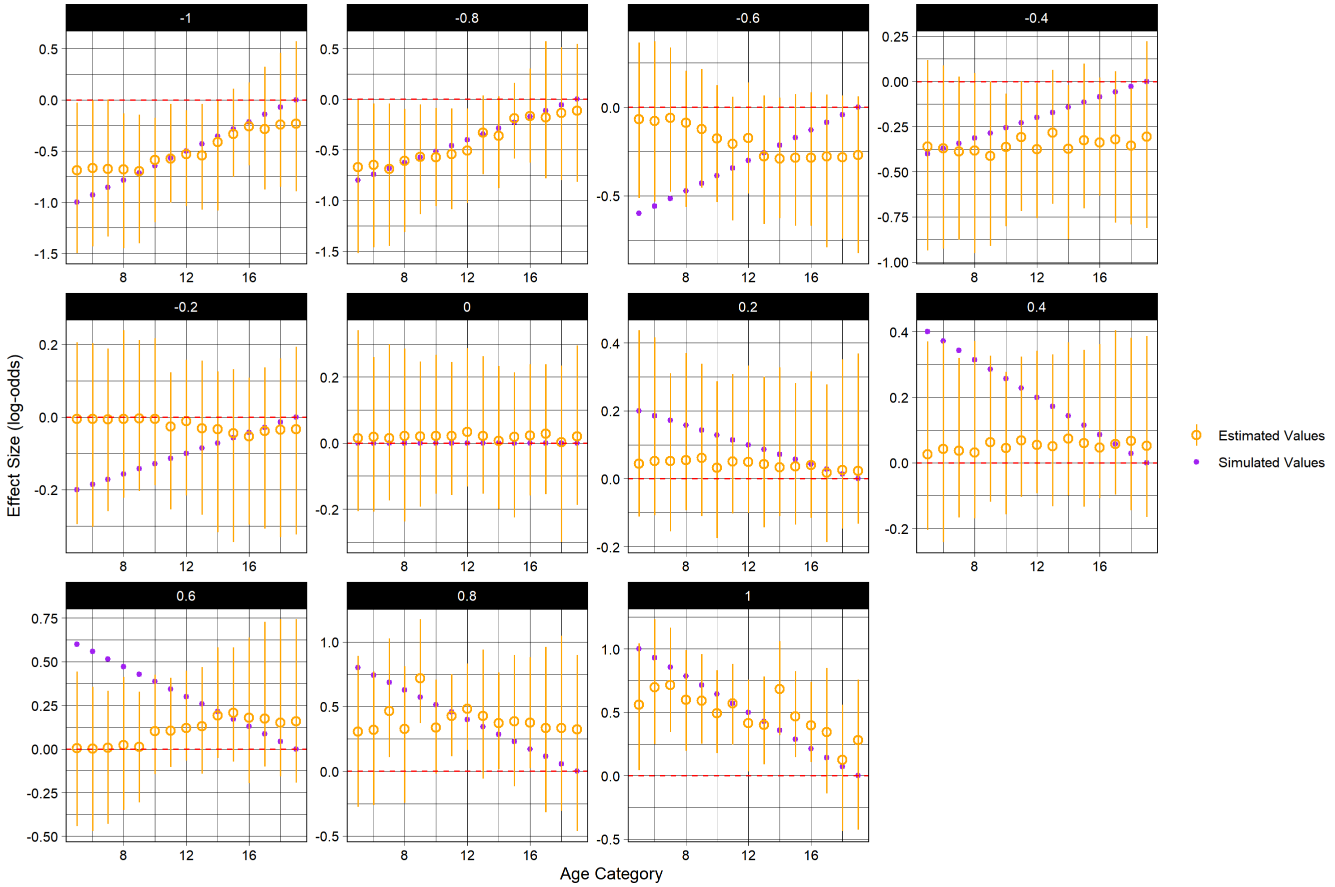

Fig. S84. Education power analysis for the “mother deceased” effect. Each panel shows the simulated (purple) and estimated (yellow) effect sizes at each age, for different ranges of simulated parameter values.

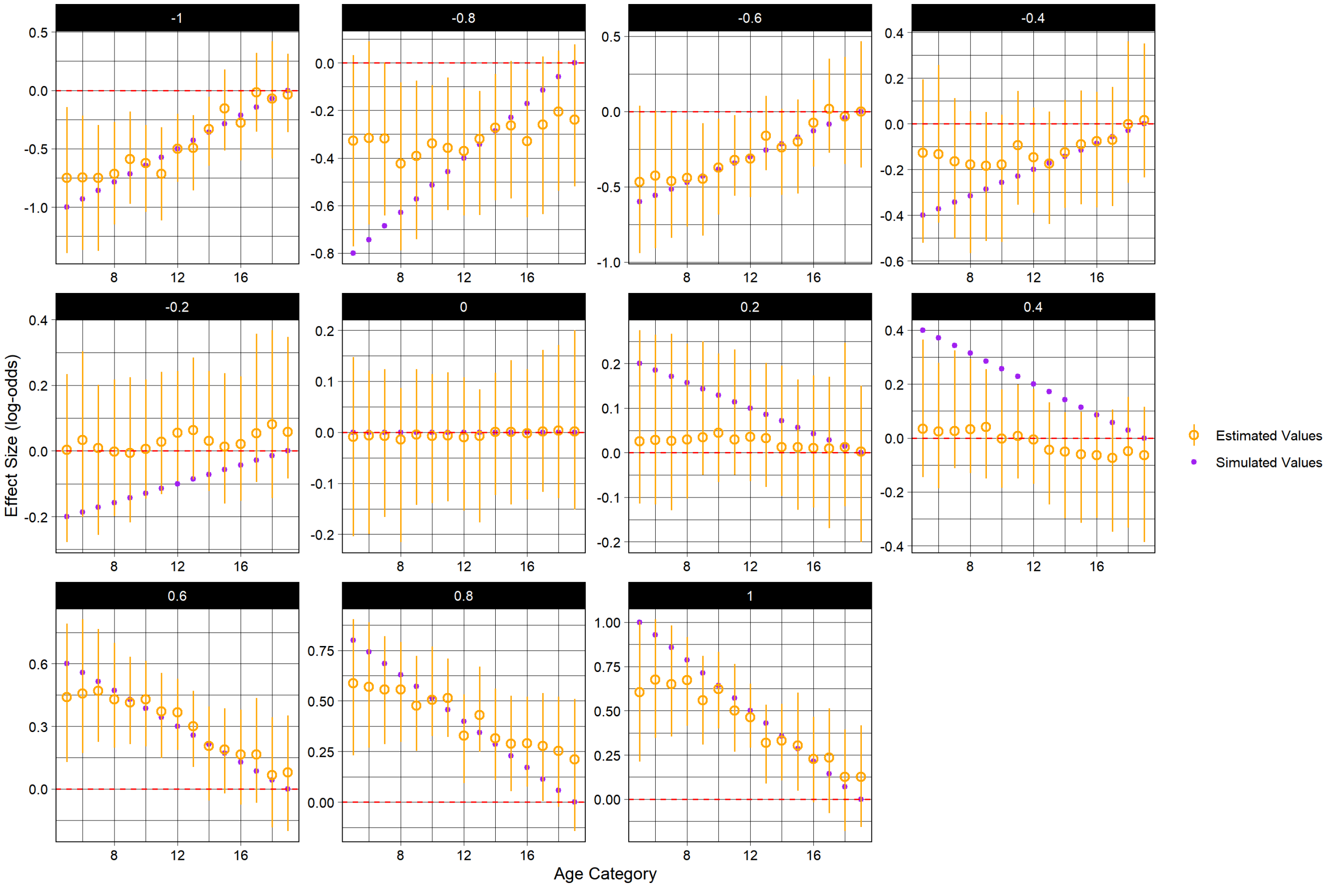

Fig. S85. Education power analysis for the "father deceased" effect. Each panel shows the simulated (purple) and estimated (yellow) effect sizes at each age, for different ranges of simulated parameter values.

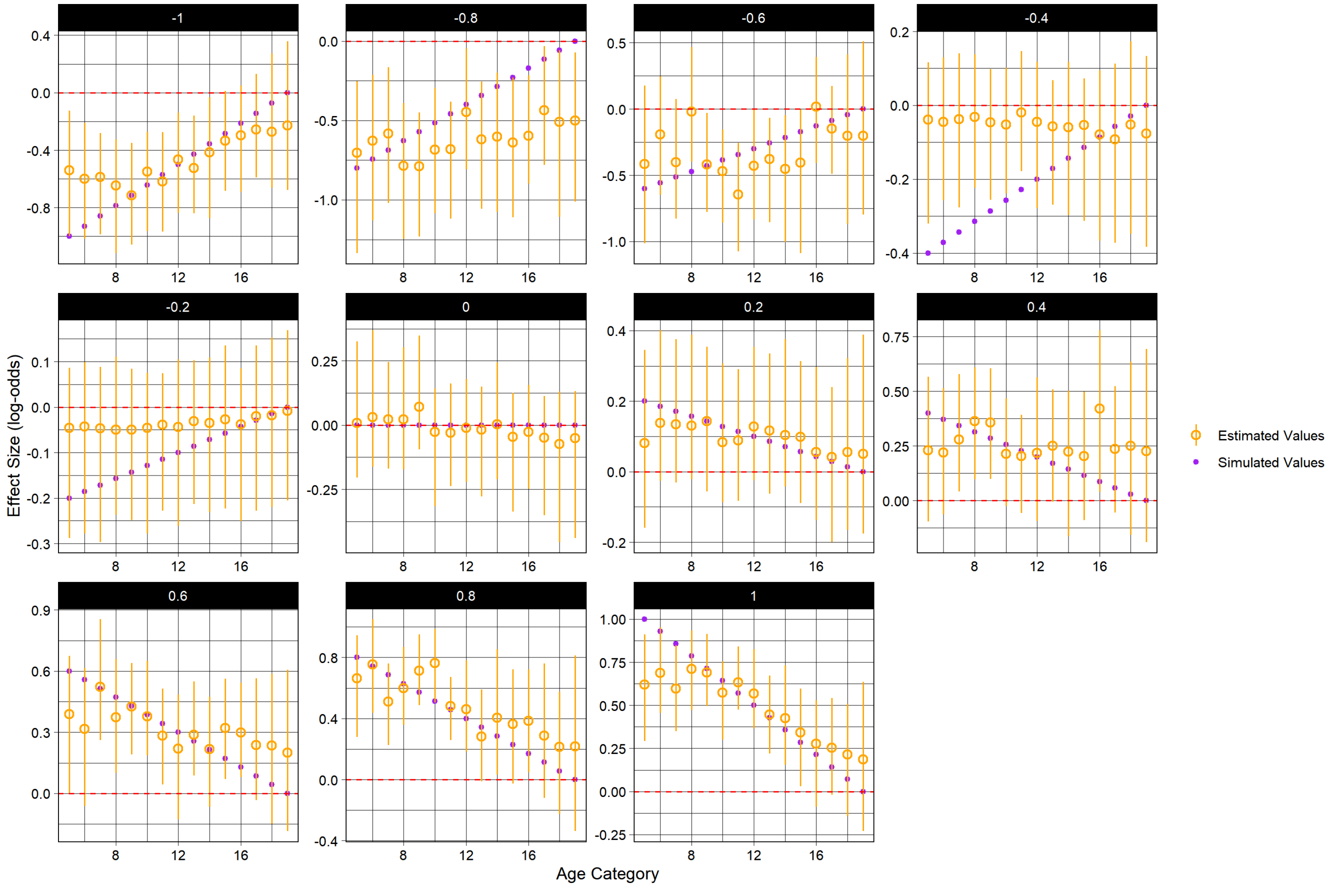

Fig. S86. Education power analysis for the “father unmarried” effect. Each panel shows the simulated (purple) and estimated (yellow) effect sizes at each age, for different ranges of simulated parameter values.

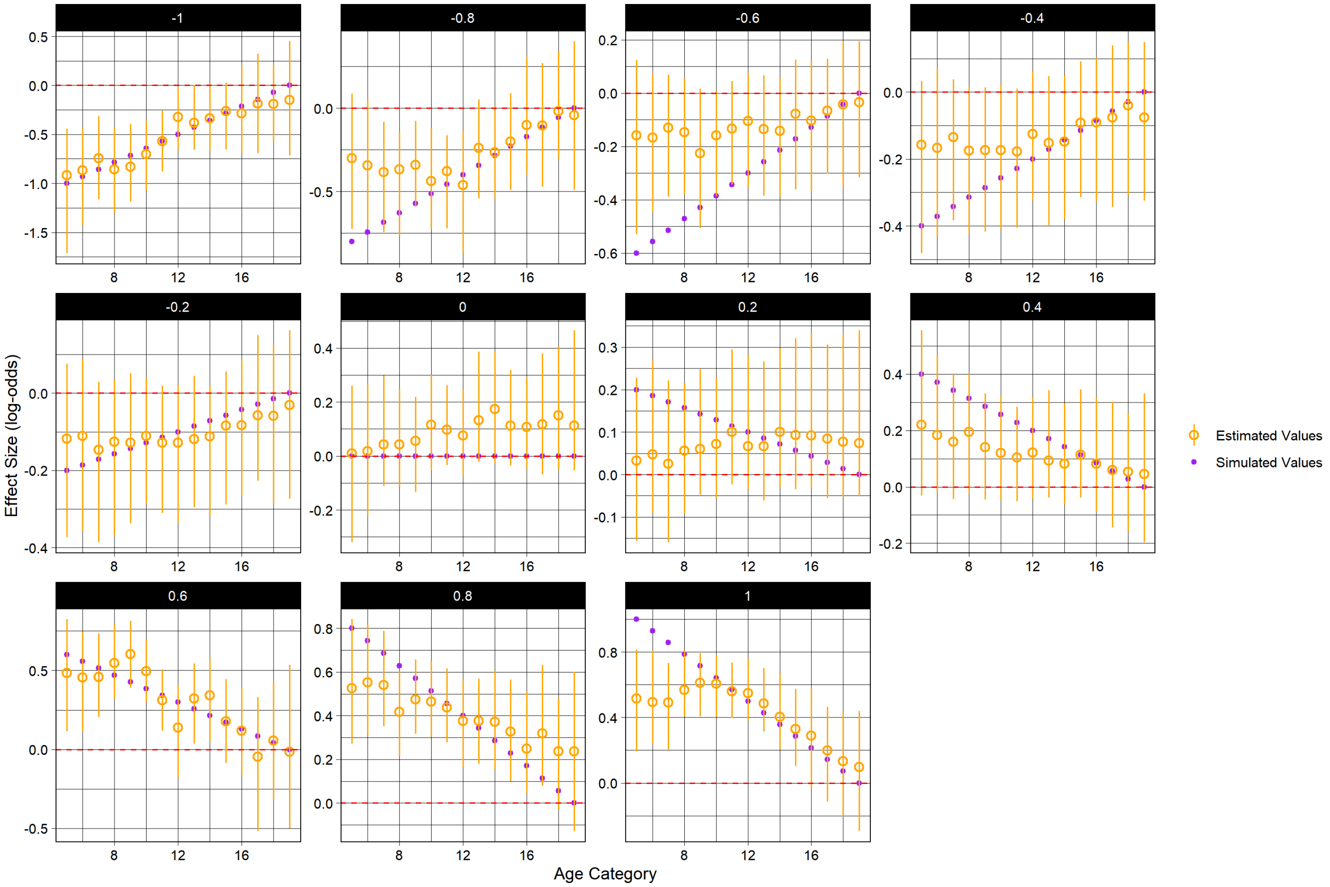

Fig. S87. Education power analysis for the “father married to stepmother (monogamy)” effect. Each panel shows the simulated (purple) and estimated (yellow) effect sizes at each age, for different ranges of simulated parameter values.

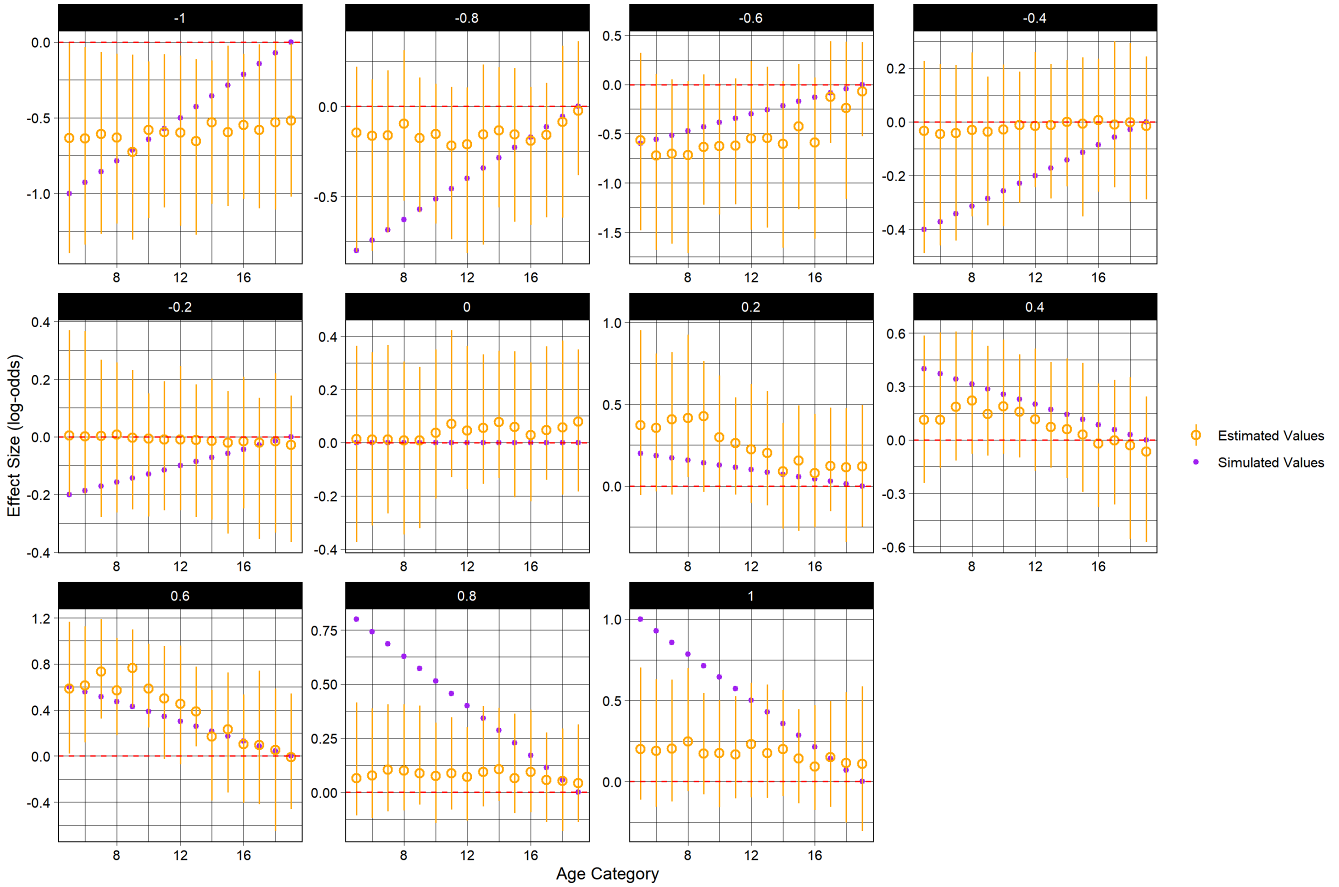

Fig. S88. Education power analysis for the “father married to stepmother (polygyny)” effect. Each panel shows the simulated (purple) and estimated (yellow) effect sizes at each age, for different ranges of simulated parameter values.

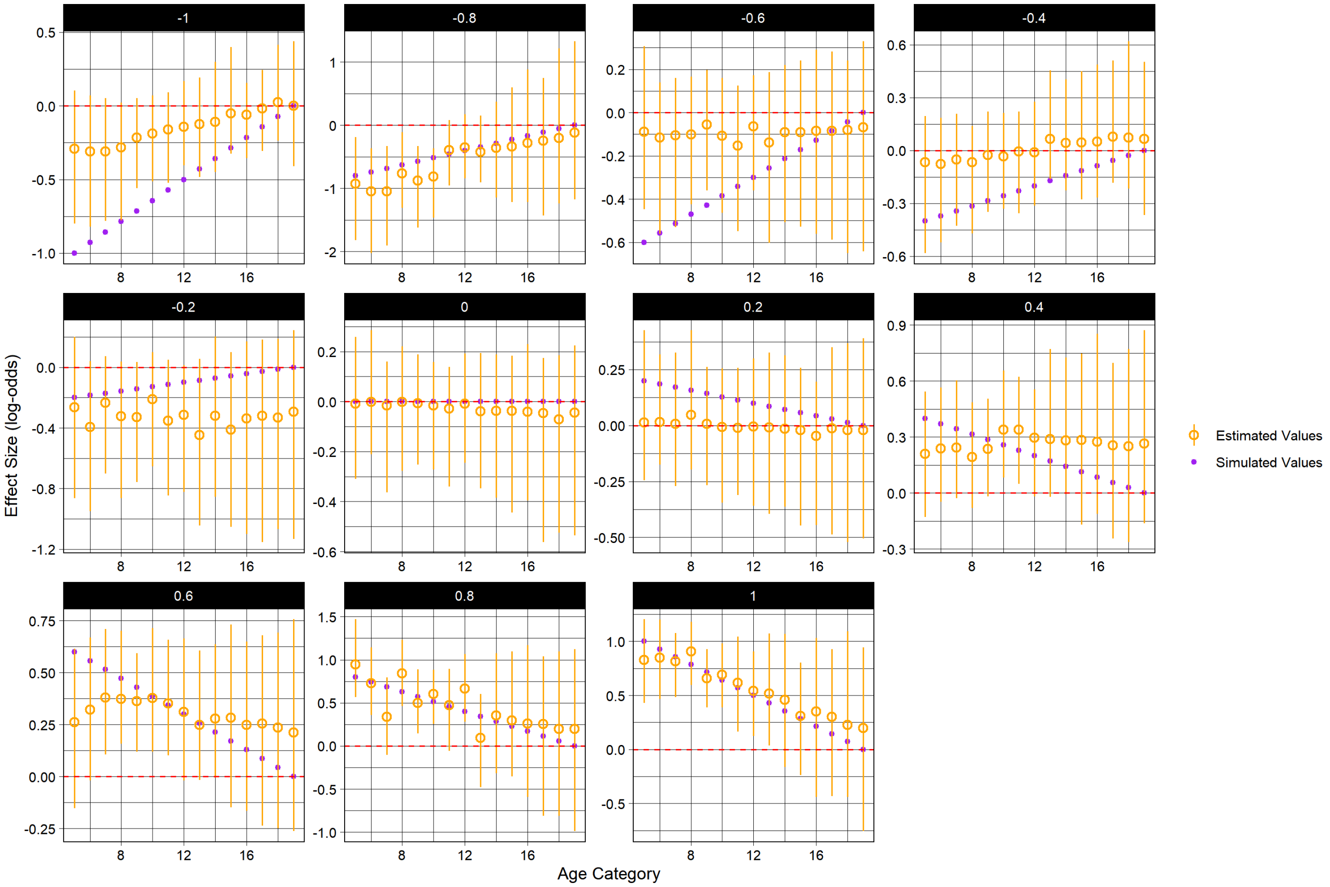

Fig. S89. Education power analysis for the “father married to biological (polygyny)” effect. Each panel shows the simulated (purple) and estimated (yellow) effect sizes at each age, for different ranges of simulated parameter values.

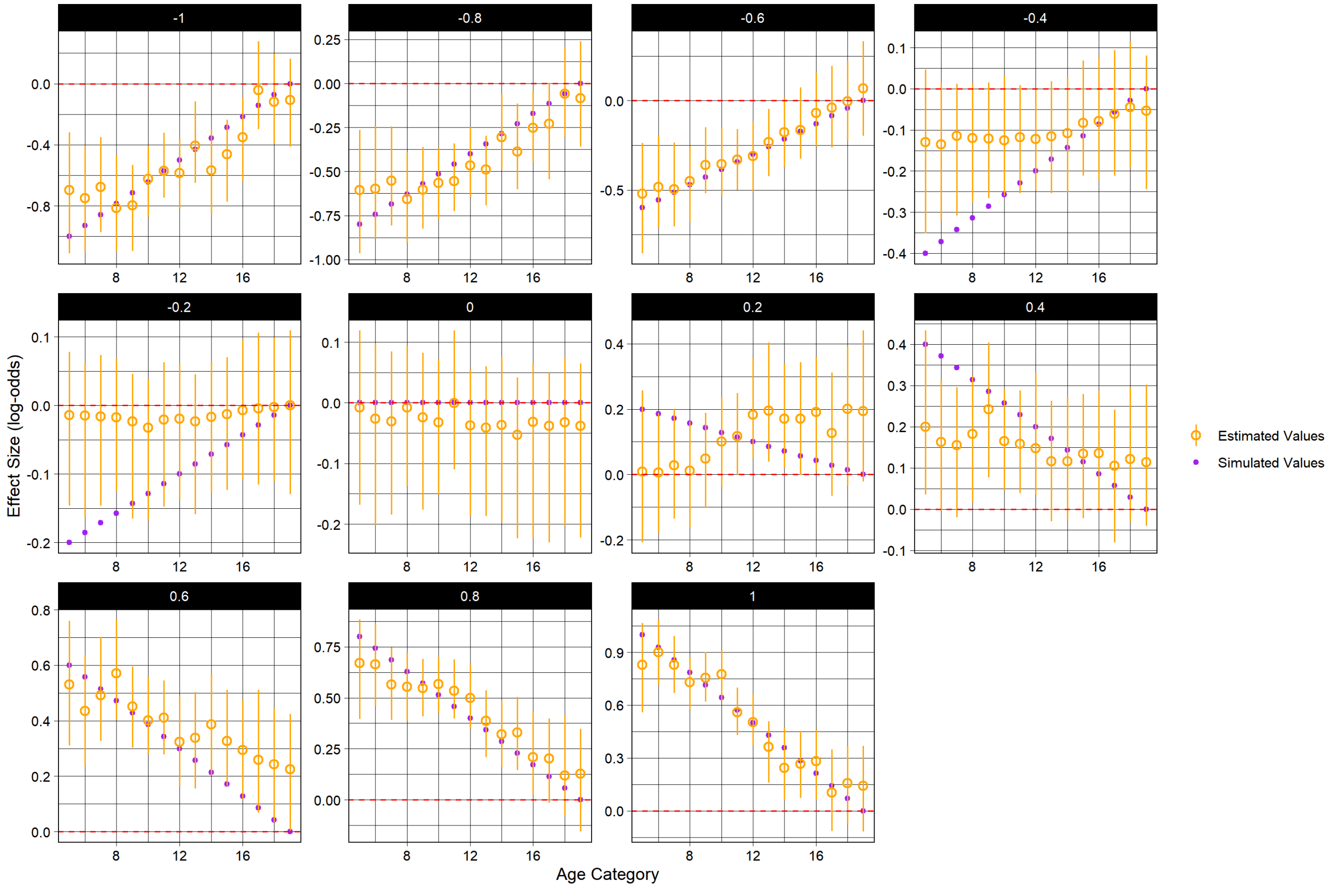

Fig. S90. Education power analysis for the “either parent external” effect. Each panel shows the simulated (purple) and estimated (yellow) effect sizes at each age, for different ranges of simulated parameter values.
